# Supplementary material for: Enantioselective synthesis of chiral quinohelicenes through sequential organocatalyzed Povarov reaction and oxidative aromatization
Source: Nat Commun. 2023 Jun 8;14:3380. doi: 10.1038/s41467-023-39134-9 (PMC10250546; doi:10.1038/s41467-023-39134-9)
Supplement: Supplementary file 1 — Supplementary Information [file 41467_2023_39134_MOESM1_ESM.pdf]

Supplementary Information

for

Enantioselective Synthesis of Chiral Quinohelicenes through Sequential Organocatalyzed Povarov Reaction and Oxidative Aromatization

Chengwen Li[1], Ying-Bo Shao[1], Xi Gao[1], Zhiyuan Ren[1], Chenhao Guo[2], Meng Li[2] and Xin Li*[,1,3]

[1]State Key Laboratory of Elemento-Organic Chemistry, College of Chemistry, Nankai University, Tianjin 300071, China.

[2]CAS Key Laboratory of Molecular Recognition and Function, Institute of Chemistry, Chinese Academy of Sciences, Beijing 100190, China.

[3]Haihe Laboratory of Sustainable Chemical Transformations, Tianjin 300192, China.

*email xin_li@nankai.edu.cn (X. Li)

Table of Contents

1. Supplementary Methods 3

1.1. General information 3

1.2. Optimization of conditions 3

1.3. General procedure for the synthesis of the amine substrates 5

1.4. General procedure for the synthesis and characterization of **4**, **5** and **6** 15

1.5. Crystal structure data of **5a** and **5al** 41

1.6. DFT calculations 43

1.7. Barriers to enantiomerization of experiments 110

1.8. Electrochemical and Photophysical properties 111

1.9. NMR spectra 118

2. Supplementary References 227

1. Supplementary Methods

1.1. General information

Commercially available materials purchased was used as received. ^{1}H **NMR** were recorded on a Bruker Avance (400 MHz) spectrometer, and reported as δ in units of parts per million (ppm) relative to tetramethylsilane (δ 0.00), and splitting patterns are designated as singlet (s), doublet (d), triplet (t), quartet (q), dd (doublet of doublets), m (multiplets). ^{13}C **NMR** were reported on a Bruker Avance (101 MHz) spectrometer, and reported as δ in units of parts per million (ppm) relative to the signal of chloroform-d (δ 77.16 triplet). ^{19}F **NMR** were reported on a Bruker Avance (376 MHz) spectrometer. Mass spectra were obtained using electrospray ionization (ESI) mass spectrometer. The electrochemical measurements were carried out using a Zahner electrochemical analyzer. UV-vis absorption spectra were performed on an Analytikjena Specord 210 Plus UV-vis spectrophotometer. Photoluminescence (PL) spectra, photoluminescence quantum yields (PLQYs) and were recorded on an Edinburgh FSL1000 Spectrofluorometer. Circular dichroism (CD) spectra were collected on MOS-450 circular dichroism spectrometer at 297 K. Circularly polarized luminescence (CPL) measurements were performed using a JASCO CPL-300 at 297 K.

1.2. Optimization of conditions

Supplementary Table 1. Optimization of the Conditions[a,d]

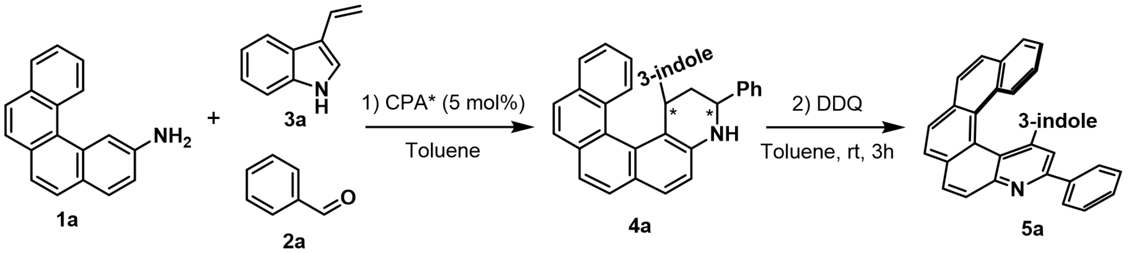

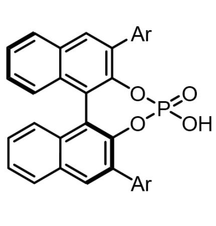

(*S*)-**A1**: Ar = 2,4,6-*i*Pr$_3$C$_6$H$_2$
(*S*)-**A2**: Ar = 1-naphthyl
(*S*)-**A3**: Ar = 4-PhC$_6$H$_4$
(*S*)-**A4**: Ar = 2-naphthyl
(S)-**A5**: Ar = 9-anthryl
(*S*)-**A6**: Ar = 3,5-(CF$_3$)$_2$C$_6$H$_2$
(*S*)-**A7**: Ar = Triphenylsilyl
(*S*)-**A8**: Ar = 4-(2-naphthyl)C$_6$H$_4$
(*S*)-**A9**: Ar = 4-*t*BuC$_6$H$_4$

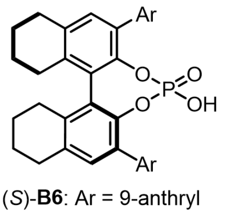

(*S*)-**B6**: Ar = 9-anthryl

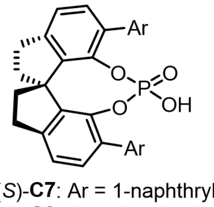

(*S*)-**C7**: Ar = 1-naphthryl
(*S*)-**C8**: Ar = 9-anthryl

Entry[a]	CPA	**4a**, yield (%)[b]	**4a**, ee(%)[c]	**5a**, yield (%)[b]	**5a**, ee(%)[c]	cp(%)[d]
1	(*S*)-**A1**	36	92	86	90	98
2	(*S*)-**A2**	69	96	89	92	96
3	(*S*)-**A3**	72	80	87	75	94
4	(*S*)-**A4**	71	72	85	68	94
5	(*S*)-**A5**	75	99	87	94	95

6	(*S*)-**A6**	49	86	84	82	95
7	(*S*)-**A7**	58	82	85	77	94
8	(*S*)-**A8**	62	91	89	85	93
9	(*S*)-**A9**	47	89	88	86	97
10	(*S*)-**B6**	63	82	86	78	95
11	(*S*)-**C7**	51	-89	89	-85	96
12	(*S*)-**C8**	74	-92	87	-87	94

Reaction conditions： [a]**1a** (0.05 mmol) and **2a** (0.2 mmol) in toluene (1.5 mL) at 110 °C for 12 h, then added **3a** (0.1 mmol) and CPA* (0.0025 mmol) at rt for 12 h. purified by silica gel column chromatography to get **4a**, all dr values of **4a** were > 20:1. [b]Yields are of the isolated. [c]The ee values were determined by high-performance liquid chromatography (HPLC) analysis with a chiral stationary phase. [d]**4a** and 1,2-Dichloro-4,5-Dicyanobenzoquinone (DDQ 3 equivale) in Toluene (2 mL) at rt for 3 h, Conversion percentage (cp) = ee_{4a} /ee_{5a} × 100%.

Supplementary Table 2. Optimization of the oxidation Conditions

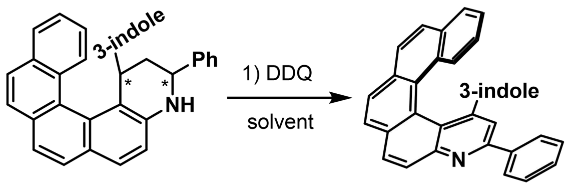

4a **5a**

Entry[a]	solvent	**5a**, yield (%)[b]	**5a**, ee(%)[c]	cp(%)[d]
1	Toluene	89	94	95
2	CH_3CN	81	95	96
3	DCM	91	99	100
4	EA	86	97	98
5	THF	92	91	92
6[e]	DCM	18	54	54
7[f]	DCM	16	46	46
8[g]	DCM	trace	---	---

Reaction conditions: [a]**4a** (0.05 mmol with 99% ee), DDQ (0.15 mmol), solvent (2 mL) at rt for 3 h. [b]Yields are of the isolated. [c]The ee values were determined by high-performance liquid chromatography (HPLC) analysis with a chiral stationary phase. [d]Conversion percentage (cp) = ee_{4a} /ee_{5a} × 100%. [e]DDQ (0.15 mmol) was replaced by MnO_2 (0.5 mmol). [f]DDQ (0.15 mmol) was replaced by Pyridinium chlorochromate (0.15 mmol). [g]DDQ (0.15 mmol) was replaced by Ag_2O (0.25 mmol).

1.3. General procedure for the synthesis of the amine substrates

1.3.1. General procedure for the synthesis of 1a-1f

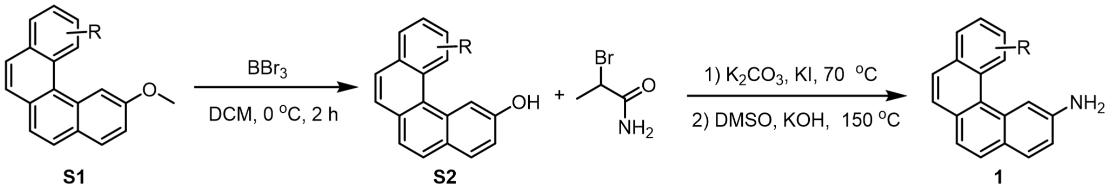

To a solution of **S1** (This compound was prepared according to the literature procedure[1]) (2 mmol) in DCM (20 mL) was added BBr_3 (3 mmol) dropwise at 0 °C. The reaction mixture was stirred at 0 °C for 2 h, then quenched with water (20 mL) extracted with DCM (3× 20 mL), the combined organic layers were dried over Na_2SO_4, and concentrated under reduced pressure to afford **S2**, which was used directly for next step without further purification.

To a slution of DMSO (15 mL) were added **S2** (2 mmol), K_2CO_3 (6 mmol), KI (0.2 mmol) and amide (6 mmol) at 70 °C until **S2** react completely, then added KOH (8 mmol) at 150 °C for 4 h. The reaction was quenched with water (200 mL) and the aqueous layer was extracted with ethyl acetate. The combined organic layers were washed with brine, dried over anhydrous Na_2SO_4, concentrated in vacuo. The crude product was purified by silica column chromatography (Petroleum ether: Ethyl acetate = 10:1) to afford **1a-1f**. This procedure was adopted from the literature.[2, 3]

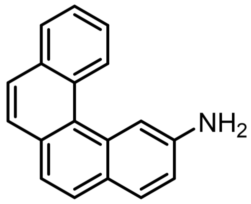

Enzo[c]phenanthren-2-amine(**1a**)

Brown solid, 86% yield form **S1**

^{1}H NMR (400 MHz, Chloroform-*d*) δ 9.15 (d, J = 8.6 Hz, 1H), 8.38 (s, 1H), 8.00 (dd, J = 8.2, 3.1 Hz, 1H), 7.87 – 7.81 (m, 2H), 7.79 (d, J = 2.9 Hz, 1H), 7.77 (d, J = 3.3 Hz, 1H), 7.68 – 7.62(m, 1H), 7.61 (d, J = 4.1 Hz, 1H), 7.60 – 7.58 (m, 1H), 7.05 (d, J = 8.6 Hz, 1H), 3.97 (s, 2H).

^{13}C NMR (101 MHz, Chloroform-*d*) δ 145.03, 133.29, 131.93, 131.74, 130.74, 129.94, 128.56, 127.43, 127.38, 127.30, 127.10, 126.03, 125.84, 125.44, 123.44, 116.67, 110.78.

HRMS (ESI) m/z: $[M+H]^+$ Calcd for $C_{18}H_{13}N^+$ 244.1121; Found 244.1110.

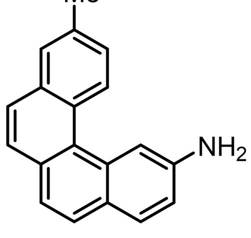

10-methylbenzo[c]phenanthren-2-amine(**1b**)

Brown solid, 90% yield form **S1**

^{1}H NMR (400 MHz, Chloroform-*d*) *δ* 9.05 (d, *J* = 8.7 Hz, 1H), 8.37 (s, 1H), 7.83 (d, *J* = 8.4 Hz, 1H), 7.80 – 7.72 (m, *J* = 8.7 Hz, 4H), 7.59 (d, *J* = 8.4 Hz, 1H), 7.49 (d, *J* = 8.7 Hz, 1H), 7.05 (d, *J* = 8.5 Hz, 1H), 3.93 (s, 2H), 2.61 (s, 3H).

^{13}C NMR (101 MHz, Chloroform-*d*) *δ* 144.90, 135.12, 133.55, 131.89, 131.32, 129.90, 128.74, 127.97, 127.76, 127.45, 127.26, 127.13, 126.97, 126.08, 123.52, 116.62, 110.84, 21.38.

HRMS (ESI) m/z: $[M+H]^+$ Calcd for $C_{19}H_{15}N^+$ 258.1277; Found 258.1269.

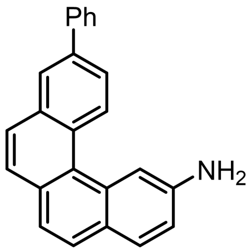

10-phenylbenzo[c]phenanthren-2-amine(**1c**)

Brown solid, 85% yield form **S1**

^{1}H NMR (400 MHz, Chloroform-*d*) *δ* 9.20 (d, *J* = 8.8 Hz, 1H), 8.38 (s, 1H), 8.19 (s, 1H), 7.92 – 7.86 (m, 2H), 7.86 – 7.72 (m, 5H), 7.59 (d, *J* = 8.4 Hz, 1H), 7.52 (t, *J* = 7.5 Hz, 2H), 7.40 (t, *J* = 7.4 Hz, 1H), 7.07 (d, *J* = 8.5 Hz, 1H), 4.00 (s, 2H).

^{13}C NMR (101 MHz, Chloroform-*d*) *δ* 145.08, 140.74, 137.89, 133.64, 131.94, 131.83, 129.99, 129.92, 128.95, 127.91, 127.54, 127.49, 127.45, 127.43, 127.35, 126.35, 125.94, 125.07, 123.46, 116.73, 110.73.

HRMS (ESI) m/z: $[M+H]^+$ Calcd for $C_{24}H_{17}N^+$ 320.1434; Found 320.1423.

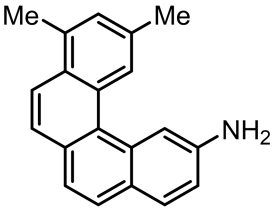

9,10-dimethylbenzo[c]phenanthren-2-amine(**1d**)

Brown solid, 89% yield form **S1**

^{1}H NMR (400 MHz, Chloroform-*d*) *δ* 8.79 (s, 1H), 8.34 (s, 1H), 8.01 (d, *J* = 8.8 Hz, 1H), 7.82 (d, *J* = 8.5 Hz, 1H), 7.75 (d, *J* = 1.6 Hz, 1H), 7.73 (d, *J* = 1.6 Hz, 1H), 7.58 (d, *J* = 8.5 Hz, 1H), 7.29 (s, 1H), 7.04 (d, *J* = 8.4 Hz, 1H), 3.95 (s, 2H), 2.79 (s, 3H), 2.59 (s, 3H).

^{13}C NMR (101 MHz, Chloroform-*d*) *δ* 144.71, 134.79, 134.31, 132.10, 131.45, 131.01, 130.21, 129.82, 128.58, 127.45, 127.18, 126.16, 125.88, 125.29, 123.37, 122.91, 116.43, 111.35, 22.24, 20.14.

HRMS (ESI) m/z: $[M+H]^+$ Calcd for $C_{20}H_{17}N^+$ 272.1434; Found 272.1424.

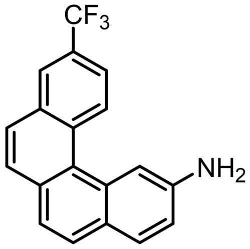

10-(trifluoromethyl)benzo[c]phenanthren-2-amine(**1e**)

Brown solid, 88% yield form **S1**

^{1}H NMR (400 MHz, Chloroform-*d*) *δ* 9.20 (d, *J* = 8.9 Hz, 1H), 8.25 (s, 2H), 7.92 – 7.71 (m, 5H), 7.58 (d, *J* = 8.5 Hz, 1H), 7.08 (dd, *J* = 8.5, 2.2 Hz, 1H), 4.15 – 3.88 (m, 2H).

^{13}C NMR (101 MHz, Chloroform-*d*) *δ* 145.49, 132.76, 132.43, 132.27, 131.80, 130.14, 128.56, 128.45, 128.08, 127.44, 127.26, 126.81, 125.83, 125.54, 123.14, 121.42, 116.99, 110.27.

^{19}F NMR (376 MHz, $CDCl_3$) δ -61.91.

HRMS (ESI) m/z: $[M+H]^+$ Calcd for $C_{19}H_{12}F_3N^+$ 312.0995; Found 312.0983.

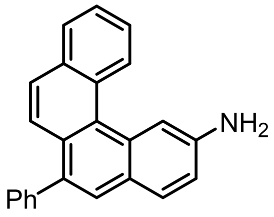

6-phenylbenzo[c]phenanthren-2-amine(**1f**)

Brown solid, 93% yield form **S1**

^{1}H NMR (400 MHz, Chloroform-*d*) *δ* 9.14 (d, *J* = 8.4 Hz, 1H), 8.35 (s, 1H), 7.95 (d, *J* = 7.8 Hz, 1H), 7.86 – 7.77 (m, 2H), 7.74 (d, *J* = 8.9 Hz, 1H), 7.70 (s, 1H), 7.63 (t, *J* = 7.6 Hz, 1H), 7.58 (t, *J* = 7.4 Hz, 1H), 7.54 –7.40(m, 5H), 7.08 (d, *J* = 8.5 Hz, 1H), 4.05 (s, 2H).

^{13}C NMR (101 MHz, Chloroform-*d*) *δ* 144.80, 141.27, 135.21, 132.94, 131.33, 130.62, 130.47, 130.16, 129.93, 128.28, 128.21, 128.06, 127.91, 127.10, 126.78, 125.73, 125.66, 124.80, 117.08, 110.91.

HRMS (ESI) m/z: $[M+H]^+$ Calcd for $C_{24}H_{17}N^+$ 320.1434; Found 320.1424.

1.3.2. General procedure for the synthesis of 1g,1h and 1i

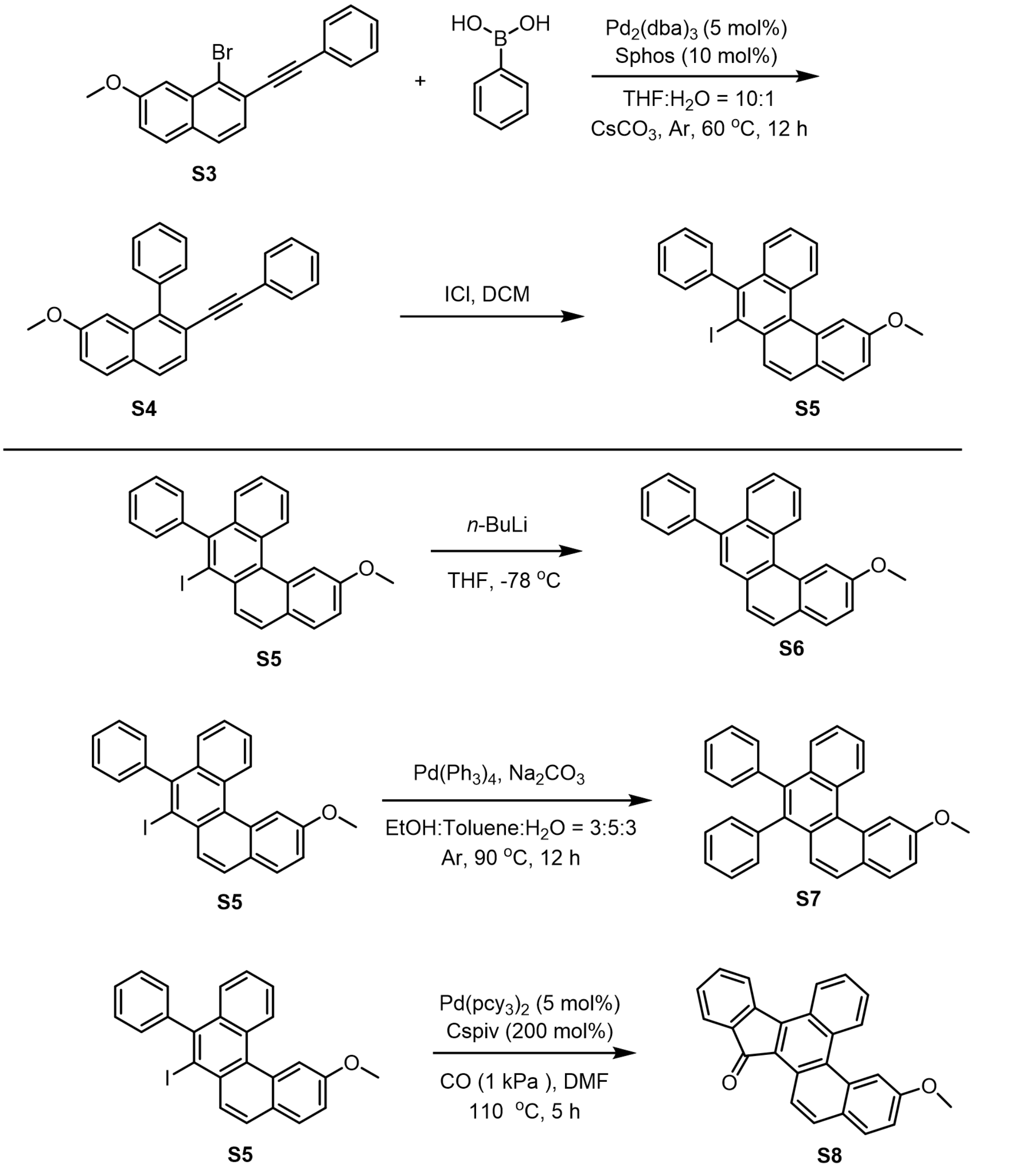

The synthesis of **S4**. Add $CsCO_3$ (1.46 g, 4.5 mmol), THF (30 mL) and H_2O (3 mL) to a flask containing corresponding **S3** (This compound was prepared according to the literature procedure.[4]) (3.0 mmol), boronic acid (4.5 mmol) and under inert atmosphere. Degas the mixture for 10 min. Then add $Pd_2(dba)_3$ (137 mg, 0.15 mmol) and DPEphos (123 mg, 0.3 mmol) to the reaction mixture. Heat the mixture at 60 °C for 12 h. Filter the mixture over a pad of silica (toluene). Distill the volatiles under

vacuum. Purify the crude product by column chromatography on silica gel column (Petroleum ether: Ethyl acetate = 20:1) to afford **S4**.

The synthesis of **S5**. Add **S4** (2.3 mmol) and DCM (20mL) to a flask, then added ICl (452mg, 2.8mmol in 4mL DCM) after the suspension of **S4** cooled to -78 °C. The reaction mixture was stirred at -78 °C for 1 h, then diluted with DCM (50 mL), washed with 50 mL of saturated aqueous $Na_2S_2O_3$. The organic layers were dried over Na_2SO_4 and evaporated under reduced pressure. Column chromatography of the crude product on silica gel (Petroleum ether: Ethyl acetate = 20:1) provided product **S5**.

The synthesis of **S6**.[5] n-BuLi 2.5 M (1.5 mmol, 0.6 mL) was added dropwise to a solution of the corresponding alkyne (1 mmol) in anhydrous THF (10 mL) at -78 °C under inert atmosphere. The reaction mixture was stirred at -78 °C for 1 h, then quenched with water (20 mL) extracted with EA (3× 10 mL), the combined organic layers were dried over Na_2SO_4, and concentrated under reduced pressure. Column chromatography of the residue on silica gel (Petroleum ether: Ethyl acetate = 20:1) provided product **S6**.

The synthesis of **S7**.[1] Add 2 M aqueous Na_2CO_3 (3 mL, 2 mmol, 2 equivalents), toluene (5 mL) and **S5** (1 mmol) to a mixture of $Pd(PPh_3)_4$ (115 mg, 0.1 mmol, 10 mol%), arylboronic acid (1.2 mmol) in ethanol (3 mL). Heat the mixture at 90 °C for 12 hours. After complete conversion of aryl halide (monitored by TLC analysis), cool down the mixture to room temperature. Extract the mixture with ethyl acetate (20mL × 3). Wash the combined organic layer with saturated aqueous NaCl (20 mL × 2). Dry the combined organic layer over anhydrous Na_2SO_4. Remove the solvent under reduced pressure. Purify the crude product by silica gel chromatography (Petroleum ether: Ethyl acetate = 20:1) to afford **S7**.

The synthesis of **S8**.[6, 7] Add $Pd(PCy_3)_2$ (0.05 mmol) and anhydrous cesium pivalate (2 mmol) to a solution of the **S5** (1 mmol) in DMF (10 mL) under CO (1 kPa). The reaction mixture was stirred at 110 °C for 5 h, then quenched with water (50 mL) extracted with ethyl acetate (3 × 20 mL), the combined organic layers were dried over Na_2SO_4, and concentrated under reduced pressure. Column chromatography of the crude product on silica gel (Petroleum ether: Ethyl acetate = 10:1) provided product **S8**.
1g,1h and 1i were synthesized using the same procedure with **1a** from **S6**, **S7** and **S8** respectively.

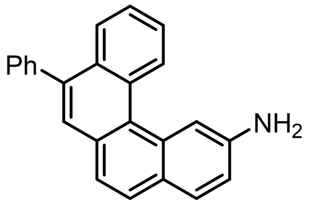

8-phenylbenzo[c]phenanthren-2-amine(**1g**)
Brown solid, 76% yield form **S6**
^{1}H NMR (400 MHz, Chloroform-*d*) *δ* 9.19 (d, *J* = 8.6 Hz, 1H), 8.37 (s, 1H), 8.01 (d, *J* = 8.3 Hz, 1H), 7.83 (d, *J* = 8.5 Hz, 1H), 7.77 (d, *J* = 8.4 Hz, 1H), 7.72 (s, 1H), 7.64 (t, *J* = 7.6 Hz, 1H), 7.63 – 7.55 (m, 3H), 7.56 – 7.42 (m, 4H), 7.06 (d, *J* = 9.4 Hz, 1H), 3.99 (s, 2H).
^{13}C NMR (101 MHz, Chloroform-*d*) *δ* 145.11, 140.73, 138.86, 131.98, 131.66, 131.12, 131.06, 130.09, 129.98, 128.38, 127.73, 127.67, 127.49, 127.35, 126.70, 125.64, 125.60, 125.48, 123.37, 116.64, 110.77.

HRMS (ESI) m/z: $[M+H]^+$ Calcd for $C_{24}H_{17}N^+$ 320.1434; Found 320.1424.

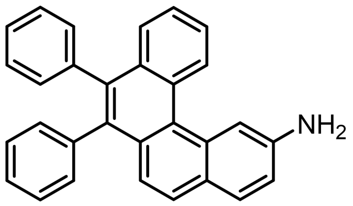

7,8-diphenylbenzo[c]phenanthren-2-amine(**1h**)

Brown solid, 59% yield form **S7**

^{1}H NMR (400 MHz, Chloroform-*d*) δ 9.17 (d, *J* = 8.4 Hz, 1H), 8.39 (s, 1H), 7.80 (d, *J* = 8.5 Hz, 1H), 7.66 (d, *J* = 8.5 Hz, 1H), 7.64 – 7.60 (m, 2H), 7.47 (t, *J* = 7.6 Hz, 1H), 7.30 – 7.12 (m, 11H), 7.07 (dd, *J* = 8.5, 2.2 Hz, 1H), 4.00 (s, 2H).

^{13}C NMR (101 MHz, Chloroform-*d*) δ 144.97, 139.91, 139.63, 137.33, 132.55, 131.49, 131.20, 131.13, 130.89, 130.14, 129.68, 127.56, 127.40, 127.11, 127.04, 126.45, 126.43, 126.12, 125.57, 125.34, 121.96, 116.93, 111.24.

HRMS (ESI) m/z: $[M+H]^+$ Calcd for $C_{30}H_{21}N^+$ 396.1747; Found 396.1738.

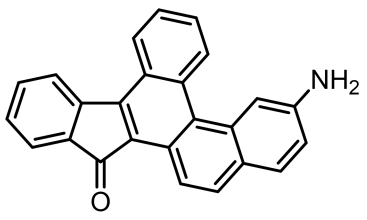

5-amino-15H-benzo[c]indeno[2,1-a]phenanthren-15-one(**1i**)

Red solid, 71% yield form **S8**

^{1}H NMR (400 MHz, DMSO-*d*$_6$) δ 9.13 (d, *J* = 8.6 Hz, 1H), 8.84 (d, *J* = 8.2 Hz, 1H), 8.74 (d, *J* = 8.7 Hz, 1H), 8.31 (d, *J* = 6.6 Hz, 1H), 8.07 (s, 1H), 7.93 – 7.82 (m, 2H), 7.84 – 7.76 (m, 2H), 7.69 – 7.58 (m, 2H), 7.52 – 7.34 (m, 1H), 7.09 (d, *J* = 8.6 Hz, 1H), 5.73 (s, 2H).

^{13}C NMR (101 MHz, DMSO-*d*$_6$) δ 195.77, 148.95, 143.73, 143.08, 135.31, 134.72, 134.67, 131.61, 130.25, 130.21, 129.77, 129.49, 128.84, 127.61, 127.55, 127.42, 127.25, 125.88, 125.77, 125.36, 124.43, 123.81, 117.69, 116.09, 108.76.

HRMS (ESI) m/z: $[M+H]^+$ Calcd for $C_{25}H_{15}NO^+$ 346.1227; Found 246.1219.

1.3.3. General procedure for the synthesis of 1j – 1l

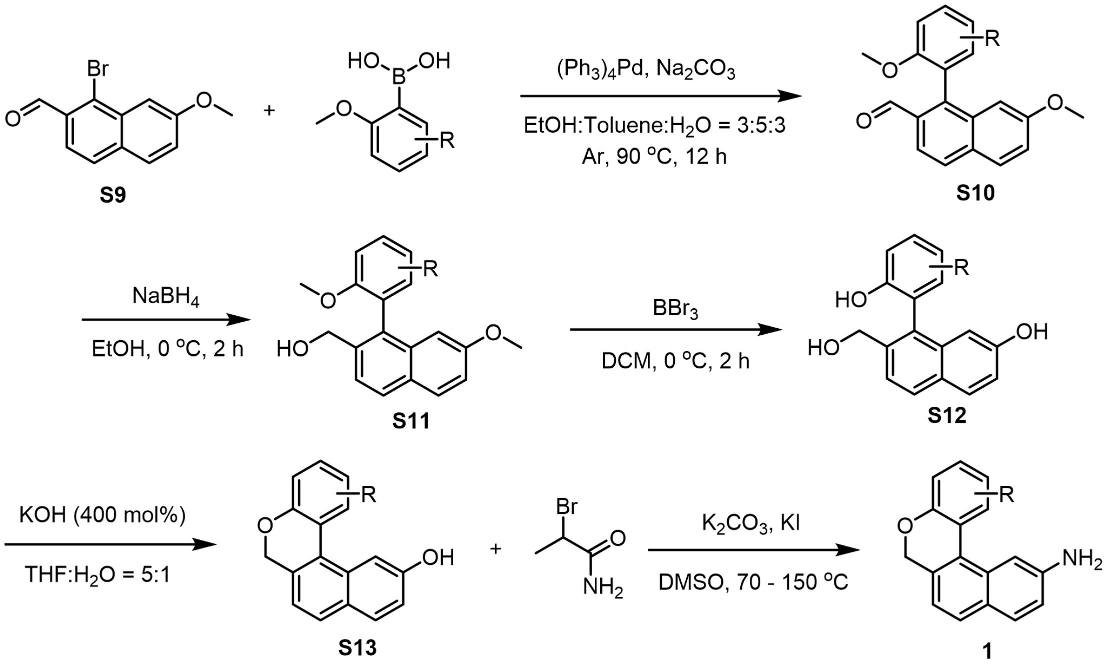

S10 was synthesized using the same reaction condition with **S7** from corresponding **S9** and arylboronic acid.

The synthesis of **S11**. $NaBH_4$ (114 mg, 3 mmol) was added to a solution of the corresponding **S10** (2 mmol) in EtOH (20 mL) at 0 °C for 2 h. Then remove solvent under reduced pressure. The reaction mixture was redissolved in DCM (40 mL), quenched with water (20 mL × 3). The organic layers were dried over Na_2SO_4, and concentrated under reduced pressure to afford **S11**, which was used directly for next step without further purification.

The synthesis of **S12**. To a solution of **S11** (2 mmol) in DCM (20 mL) was added BBr_3 (5 mmol) dropwise at 0 °C. The reaction mixture was stirred at 0 °C for 2 h, then quenched with water (20 mL) extracted with DCM (3× 20 mL), the combined organic layers were dried over Na_2SO_4, and concentrated under reduced pressure to afford **S12**, which was used directly for next step without further purification.

The synthesis of **S13**. To a solution **S12** (2 mmol) in THF/H_2O = 5/1(20 mL + 4 mL) was added KOH (8 mmol, 448 mg) at 60 °C for 4h. Then the reaction was cooled to room temperature, quenched with water (20 mL) extracted with ethyl acetate (3× 20 mL), the combined organic layers were dried over Na_2SO_4,

and concentrated under reduced pressure to afford **S13**, which was used directly for next step without further purification.

1j,1k and 1l were synthesized using the same procedure with **1a** from corresponding **S13** respectively.

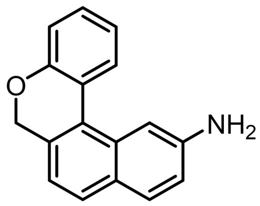

6H-naphtho[2,1-c]chromen-11-amine(**1j**)

Gray solid, 85% yield form **S13**

^{1}H NMR (400 MHz, Chloroform-*d*) *δ* 8.16 (d, *J* = 7.7 Hz, 1H), 7.84 (s, 1H), 7.77 (d, *J* = 8.7 Hz, 1H), 7.71 (d, *J* = 8.1 Hz, 1H), 7.37 (t, *J* = 7.7 Hz, 1H), 7.25 (d, *J* = 8.1 Hz, 2H), 7.12 (d, *J* = 8.1 Hz,1H), 7.02 (d, *J* = 8.7 Hz, 1H), 5.16 (s, 2H), 3.94 (s, 2H).

^{13}C NMR (101 MHz, Chloroform-*d*) *δ* 156.49, 145.38, 133.47, 130.74, 130.42, 128.96, 128.27, 128.12, 127.52, 124.76, 124.58, 121.67, 119.08, 117.67, 117.58, 106.17, 70.17.

HRMS (ESI) m/z: $[M+H]^+$ Calcd for $C_{17}H_{13}NO^+$ 248.1070; Found 248.1060.

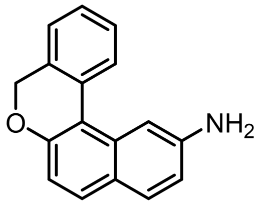

5H-dibenzo[c,f]chromen-11-amine(**1k**)

Gray solid, 89% yield form **S13**

^{1}H NMR (400 MHz, Chloroform-*d*) *δ* 8.00 (t, *J* = 6.4 Hz, 1H), 7.65 (s, 1H), 7.59 (d, *J* = 8.6 Hz, 1H), 7.53 (dd, *J* = 9.1, 4.1 Hz, 1H), 7.35 (d, *J* = 7.7 Hz, 1H), 7.30 – 7.19 (m, 2H), 6.93 (t, *J* = 8.3 Hz, 1H), 6.80 (d, *J* = 8.6 Hz, 1H), 4.94 (s, 2H), 3.83 (s, 2H).

^{13}C NMR (101 MHz, Chloroform-*d*) *δ* 154.92, 145.54, 132.79, 131.96, 130.72, 130.34, 130.13, 128.01, 126.61, 125.60, 125.21, 124.97, 115.73, 114.51, 106.32, 69.26.

HRMS (ESI) m/z: $[M+H]^+$ Calcd for $C_{17}H_{13}NO^+$ 248.1070; Found 248.1062.

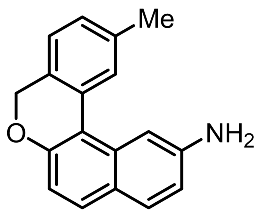

2-methyl-5H-dibenzo[c,f]chromen-11-amine(**1l**)

Gray solid, 78% yield form **S13**

^{1}H NMR (400 MHz, Chloroform-*d*) *δ* 7.87 (s, 1H), 7.72 (s, 1H), 7.65 (d, *J* = 8.6 Hz, 1H), 7.58 (d, *J* = 8.7 Hz, 1H), 7.20 (d, *J* = 7.6 Hz, 1H), 7.13 (d, *J* = 7.6 Hz, 1H), 6.99 (d, *J* = 8.8 Hz, 1H), 6.86 (dd, *J* = 8.6, 2.2 Hz,

1H), 4.98 (s, 2H), 3.91 (s, 2H), 2.44 (s, 3H).

^{13}C NMR (101 MHz, Chloroform-*d*) *δ* 154.97, 145.41, 137.61, 131.96, 130.61, 130.33, 129.99, 129.96, 127.36, 126.18, 125.12, 124.98, 115.95, 115.67, 114.60, 106.44, 69.14, 21.83.

HRMS (ESI) m/z: [M+H]$^+$ Calcd for $C_{18}H_{15}NO^+$ 262.1227; Found 262.1219.

1.3.4. The procedure for the synthesis of 1m and 1n

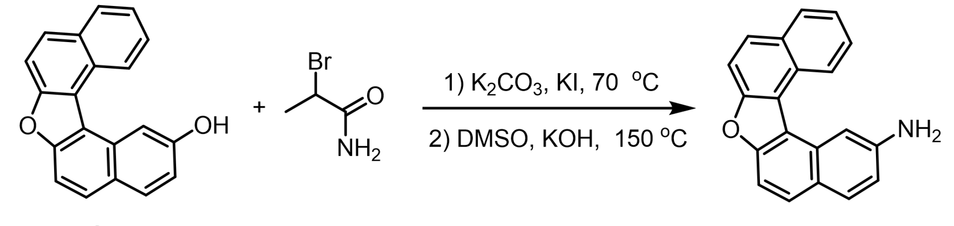

S14 **1m**

S14 was prepared according to the literature procedure[8]. **1m** was synthesized using the same procedure with **1a** from corresponding **S14**.

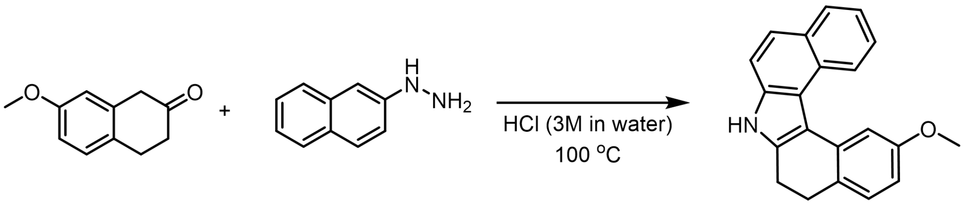

S15

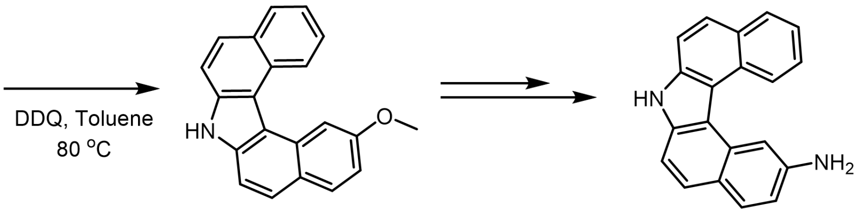

S16 **1n**

The synthesis of **S12**.[9] 17 mL of hydrochloric acid (36%) and 50 mL water were mixed. The mixture was heated to reflux, naphthylhydrazine (4.42 g, 28mmol) was added, and the contents of the flask were rapidly heated to reflux with stirring. 7-methoxy-3,4-dihydronaphthalen-2(1H)-one (20 mmol, 3.52 g) was then added dropwise within 1 h, and the mixture was continuously heated to reflux for 1 h. After cooling, the precipitated solid was recovered by filtration, washed with water and aqueous ethanol (75%, v/v), and dried at room temperature for 24 h to afford **S15**, which was used directly for next step without further purification.

The synthesis of **S16**.[10] To a solution of **S15** (15 mmol) in toluene (30 mL) added DDQ (1,2-Dichloro-4,5-Dicyanobenzoquinone, 30mmol, 6.8 g) at 80 °C for 6 h. Then the mixture was diluted with hexanes (50 mL), filtered, and concentrated under reduced pressure. Column chromatography of the residue on silica gel (Petroleum ether: Ethyl acetate = 15:1) provided product **S16.**

1n were synthesized using the same procedure with **1a** from corresponding **S16**.

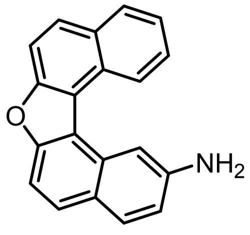

Dinaphtho[2,1-b:1',2'-d]furan-2-amine(1m)

Yellow solid, 80% yield form **S14**

^{1}H NMR (400 MHz, Chloroform-*d*) *δ* 9.15 (d, *J* = 8.6 Hz, 1H), 8.31 (s, 1H), 8.09 (d, *J* = 8.1 Hz, 1H), 7.95 (d, *J* = 8.9 Hz, 1H), 7.89 (d, *J* = 8.6 Hz, 1H), 7.85 (d, *J* = 3.1 Hz, 1H), 7.82 (d, *J* = 3.0 Hz, 1H), 7.73 (t, *J* = 7.8 Hz, 1H), 7.68 – 7.55 (m, 2H), 7.04 (dd, *J* = 8.6, 2.3 Hz, 1H), 4.13 (s, 2H).

^{13}C NMR (101 MHz, Chloroform-*d*) *δ* 155.25, 154.03, 144.84, 131.24, 130.91, 130.36, 129.49, 128.57, 128.38, 127.72, 125.78, 125.73, 125.24, 124.16, 119.67, 117.87, 115.79, 112.70, 109.07, 108.03.

HRMS (ESI) m/z: $[M+H]^+$ Calcd for $C_{20}H_{13}NO^+$ 284.1070; Found 284.1062.

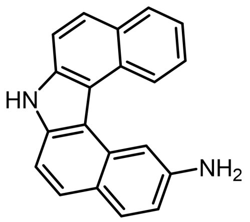

7H-dibenzo[c,g]carbazol-2-amine(1n)

Gray solid, 18% yield form **S16**

^{1}H NMR (400 MHz, DMSO-*d$_6$*) *δ* 12.03 (s, 1H), 9.10 (d, *J* = 8.5 Hz, 1H), 8.12 (s, 1H), 8.05 (d, *J* = 8.0 Hz, 1H), 7.85 (d, *J* = 8.7 Hz, 1H), 7.78 – 7.72 (m, 2H), 7.69 – 7.61 (m, 2H), 7.48 (t, *J* = 7.4 Hz, 1H), 7.40 (d, *J* = 8.5 Hz, 1H), 6.89 (dd, *J* = 8.6, 2.1 Hz, 1H), 5.57 (s, 2H).

^{13}C NMR (101 MHz, DMSO-*d$_6$*) *δ* 147.32, 137.79, 136.54, 131.34, 130.67, 129.75, 129.49, 129.10, 127.04, 125.88, 125.59, 125.39, 122.87, 122.42, 117.14, 115.40, 114.55, 113.83, 108.73, 106.18.

HRMS (ESI) m/z: $[M+H]^+$ Calcd for $C_{20}H_{14}N_2^+$ 283.1230; Found 283.1220.

1.4. General procedure for the synthesis and characterization of 4, 5 and 6

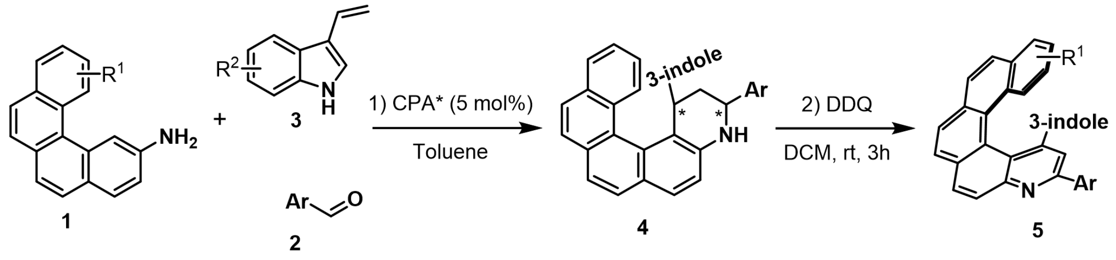

To a solution of **1** (0.1 mmol) in toluene (2 mL) was added **2** (0.4 mmol). The reaction was stirred at 110 °C for 12 h then cooled to room temperature, after that **3** (0.2 mmol) and (*S*)-**A5** (0.005 mmol) were added and allowed to proceed for another 12 h. Column chromatography of the crude product on silica gel (Petroleum ether: Ethyl acetate = 10:1) provided product **4**.

Add DDQ (0.3 mmol) to a suspension of **4** in DCM (3 mL) at room temperature for 3 h. Then diluted with ethyl acetate (20 mL), washed with 25 mL of saturated aqueous $Na_2S_2O_3$. The organic layers were dried over Na_2SO_4, and concentrated under reduced pressure. Column chromatography of the crude product on silica gel (Petroleum ether: Ethyl acetate = 10:1) provided product **5**.

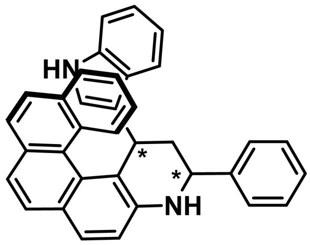

1-(1H-indol-3-yl)-3-phenyl-2,3-dihydro-1H-4l2-phenanthro[4,3-f]quinoline (**4a**)

Yellow solid, $[\alpha]_D^{24}$= - 115.2 (c = 0.1, acetone).

^{1}H NMR (400 MHz, DMSO-*d*$_6$) *δ* 9.84 (s, 1H), 8.51 (d, *J* = 8.2 Hz, 1H), 7.89 (d, *J* = 7.9 Hz, 1H), 7.81 – 7.70 (m, 2H), 7.66 – 7.55 (m, 3H), 7.52 (d, *J* = 7.6 Hz, 2H), 7.45 (d, *J* = 8.5 Hz, 1H), 7.41 – 7.26 (m, 4H), 7.21 (d, *J* = 8.1 Hz, 1H), 6.80 (d, *J* = 8.0 Hz, 1H), 6.71 (s, 1H), 6.61 (t, *J* = 7.5 Hz, 1H), 6.34 (t, *J* = 7.5 Hz, 1H), 5.96 (d, *J* = 8.0 Hz, 1H), 5.34 (t, *J* = 8.8 Hz, 1H), 5.10 (d, *J* = 2.3 Hz, 1H), 4.80 (dd, *J* = 11.2 Hz, 1H), 2.31 – 2.22 (m, 1H), 2.00 (q, *J* = 11.3 Hz, 1H).

^{13}C NMR (101 MHz, DMSO-*d*$_6$) *δ* 147.22, 144.10, 135.91, 131.24, 131.16, 130.78, 130.22, 128.85, 127.95, 127.75, 127.67, 127.51, 127.45, 127.30, 127.24, 126.51, 126.24, 126.02, 125.11, 125.07, 121.93, 121.48, 120.08, 118.71, 118.34, 118.16, 117.67, 117.55, 110.90, 55.99, 43.39, 36.54.

HRMS (ESI) m/z: $[M+Na]^+$ Calcd for $C_{35}H_{26}N_2Na^+$ 479.1988; Found 479.1951.

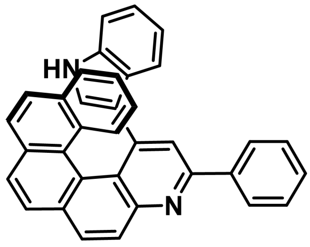

1-(1H-indol-3-yl)-3-phenylphenanthro[4,3-f]quinoline (**5a**)

Yellow solid, 31.5 mg, 67% yield with 99% ee. $[\alpha]_D^{24}$= 1832.5 (c = 0.1, acetone).

HPLC separation Chiralpak AD-H, i-PrOH / hexane = 1 / 3, 1.0 mL/min, 210 nm; tr (minor) = 5.7 min, tr(major) = 6.7 min

^{1}H NMR (400 MHz, DMSO-*d6*) *δ* 10.68 (s, 1H), 8.44 (d, *J* = 7.6 Hz, 2H), 8.31 (d, *J* = 8.4 Hz, 1H), 8.22 (d, *J* = 8.4 Hz, 1H), 8.12 – 8.02 (m, 2H), 7.94 – 7.85 (m, 2H), 7.62 (t, *J* = 7.2 Hz, 2H), 7.56 – 7.46 (m, 3H), 7.42 (d, *J* = 8.8Hz, 1H), 7.21 (t, *J* = 7.2 Hz, 1H), 7.13 (t, *J* = 7.6 Hz, 1H), 6.93 (d, *J* = 8.0 Hz, 1H), 6.74 (t, *J* = 7.6 Hz, 1H), 6.52 – 6.23 (m, 2H), 5.96 (s, 1H).

^{13}C NMR (101 MHz, DMSO-*d6*) *δ* 154.90, 148.58, 143.80, 138.46, 135.17, 131.68, 130.27, 130.22, 130.19, 130.10, 129.48, 128.86, 128.22, 127.51, 127.46, 127.13, 126.95, 126.81, 126.27, 125.50, 124.90, 124.81, 124.67, 124.47, 124.19, 123.68, 123.02, 120.61, 118.46, 118.32, 117.99, 116.38, 110.45.

HRMS (ESI) m/z: $[M+H]^+$ Calcd for $C_{35}H_{23}N_2^+$ 471.1856; Found 471.1855.

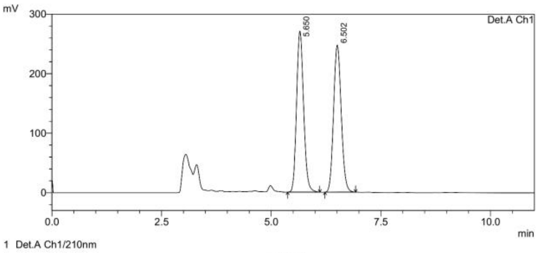

PeakTable

Detector A Ch1 210nm

Peak#	Ret. Time	Area	Height	Area %	Height %
1	5.650	3026279	270468	50.140	52.275
2	6.502	3009411	246927	49.860	47.725
Total		6035690	517395	100.000	100.000

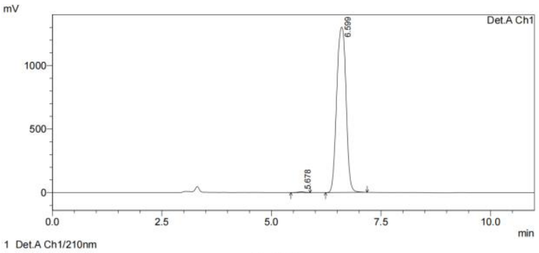

PeakTable

Detector A Ch1 210nm

Peak#	Ret. Time	Area	Height	Area %	Height %
1	5.678	72422	6972	0.374	0.534
2	6.599	19306548	1299710	99.626	99.466
Total		19378970	1306682	100.000	100.000

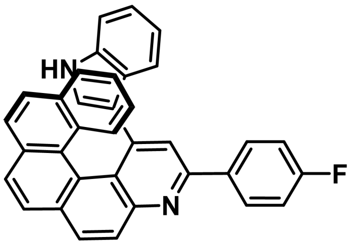

3-(4-fluorophenyl)-1-(1H-indol-3-yl)phenanthro[4,3-f]quinoline (**5b**)

Yellow solid, 28.8 mg, 59% yield with 97% ee. $[\alpha]_D^{24}$= - 1751.6 (c = 0.1, acetone).

HPLC separation Chiralpak AD-H, i-PrOH / hexane = 1 / 9, 1.0 mL/min, 210 nm; tr (minor) = 16.4 min, tr(major) = 19.5 min

^{1}H NMR (400 MHz, DMSO-*d6*) *δ* 10.69 (s, 1H), 8.67 – 8.43 (m, 2H), 8.30 (d, *J* = 8.7 Hz, 1H), 8.19 (d, *J* = 8.7 Hz, 1H), 8.12 – 8.01 (m, 2H), 7.92 – 7.85 (m, 2H), 7.55 – 7.41 (m, 5H), 7.21 (t, *J* = 7.3 Hz, 1H), 7.13 (t, *J* = 7.8 Hz, 1H), 6.93 (d, *J* = 8.1 Hz, 1H), 6.73 (t, *J* = 7.5 Hz, 1H), 6.43 – 6.36 (m, 2H), 5.93 (s, 1H).

^{13}C NMR (101 MHz, DMSO-*d6*) *δ* 163.64(d, *J* = 246.7 Hz), 154.41, 149.09, 144.37, 135.82, 135.5(d, *J* = 2.8 Hz), 132.17, 130.77, 130.73, 130.69, 130.61, 129.8 (d, *J* = 8.5 Hz), 128.72, 128.01, 127.94, 127.45, 127.31, 126.75, 125.99, 125.39, 125.33, 125.16, 124.9 (d, *J* = 6.7 Hz), 124.11, 123.55, 121.08, 118.93, 118.65, 118.49, 116.91, 116.2 (d, *J* = 21.5 Hz), 110.98.

^{19}F NMR (376 MHz, DMSO-*d6*) *δ* -112.45.

HRMS (ESI) m/z: $[M+H]^+$ Calcd for $C_{35}H_{22}FN_2^+$ 489.1762; Found 489.1763.

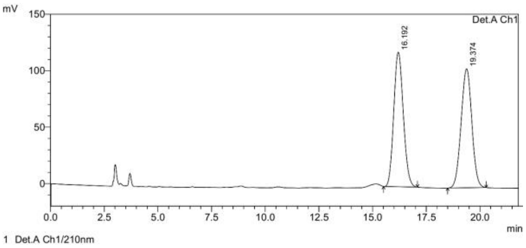

PeakTable

Detector A Ch1 210nm

Peak#	Ret. Time	Area	Height	Area %	Height %
1	16.192	3686009	118661	50.169	52.981
2	19.374	3661206	105307	49.831	47.019
Total		7347215	223968	100.000	100.000

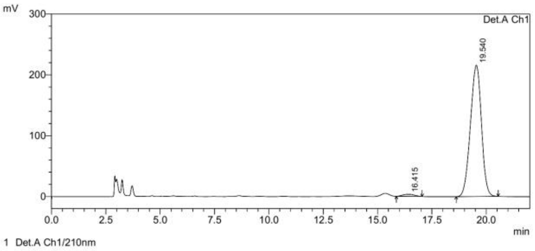

PeakTable

Detector A Ch1 210nm

Peak#	Ret. Time	Area	Height	Area %	Height %
1	16.415	128559	3416	1.659	1.560
2	19.540	7620846	215602	98.341	98.440
Total		7749404	219018	100.000	100.000

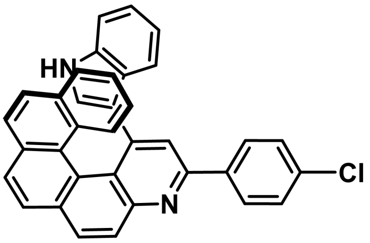

3-(4-chlorophenyl)-1-(1H-indol-3-yl)phenanthro[4,3-f]quinoline (**5c**)

Yellow solid, 23.7 mg, 47% yield with 96% ee. $[\alpha]_D^{24}$= - 1571.3 (c = 0.1, acetone).

HPLC separation Chiralpak AD-H, i-PrOH / hexane = 1 / 9, 1.0 mL/min, 210 nm; tr (minor) = 17.1 min, tr(major) = 22.8 min

^{1}H NMR (400 MHz, DMSO-d_6) δ 10.69 (s, 1H), 8.48 (d, J = 8.2 Hz, 2H), 8.31 (d, J = 8.7 Hz, 1H), 8.19 (d, J = 8.7 Hz, 1H), 8.09 (d, J = 8.0 Hz, 1H), 8.04 (d, J = 8.4 Hz, 1H), 7.99 – 7.85 (m, 2H), 7.67 (d, J = 8.2 Hz, 2H), 7.53 (d, J = 8.0 Hz, 1H), 7.48 (d, J = 8.5 Hz, 1H), 7.42 (d, J = 8.6 Hz, 1H), 7.22 (t, J = 7.5 Hz, 1H), 7.14 (t, J = 7.8 Hz, 1H), 6.92 (d, J = 8.1 Hz, 1H), 6.73 (t, J = 7.5 Hz, 1H), 6.52 – 6.29 (m, 2H), 5.91 (s, 1H).

^{13}C NMR (101 MHz, DMSO-d_6) δ 153.64, 148.58, 143.96, 137.34, 135.33, 134.28, 131.74, 130.29, 130.26, 130.21, 130.17, 128.86, 128.21, 127.53, 126.96, 126.83, 126.26, 125.52, 124.91, 124.80, 124.66, 124.47, 123.82, 123.02, 120.59, 118.45, 118.23, 117.96, 116.39, 110.49.

HRMS (ESI) m/z: $[M+H]^+$ Calcd for $C_{35}H_{22}ClN_2^+$ 505.1466; Found 505.1471.

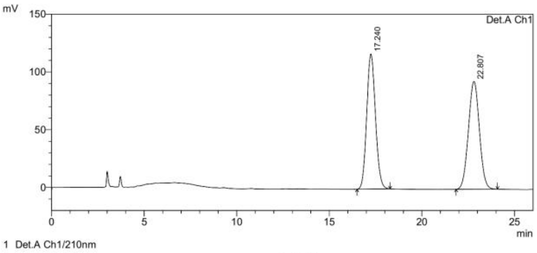

PeakTable

Detector A Ch1 210nm

Peak#	Ret. Time	Area	Height	Area %	Height %
1	17.240	3839073	116749	49.931	55.593
2	22.807	3849630	93258	50.069	44.407
Total		7688704	210007	100.000	100.000

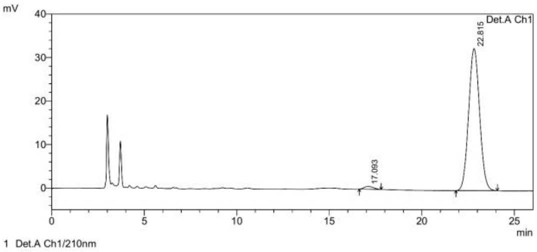

PeakTable

Detector A Ch1 210nm

Peak#	Ret. Time	Area	Height	Area %	Height %
1	17.093	22633	641	1.655	1.924
2	22.815	1345200	32658	98.345	98.076
Total		1367833	33299	100.000	100.000

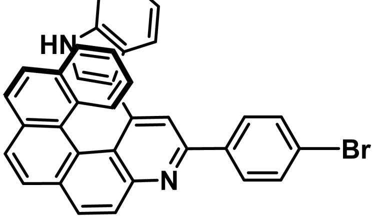

3-(4-bromophenyl)-1-(1H-indol-3-yl)phenanthro[4,3-f]quinoline (**5d**)

Yellow solid, 28.5 mg, 50% yield with 94% ee. $[\alpha]_D^{24}$= - 1104.6 (c = 0.1, acetone).

HPLC separation Chiralpak AD-H, i-PrOH / hexane = 1 / 9, 1.0 mL/min, 210 nm; tr (minor) = 18.3 min, tr(major) = 24.5 min

^{1}H NMR (400 MHz, DMSO-*d_6*) *δ* 10.71 (s, 1H), 8.41 (d, *J* = 8.1 Hz, 2H), 8.31 (d, *J* = 8.5 Hz, 1H), 8.20 (d, *J* = 8.7 Hz, 1H), 8.09 (d, *J* = 7.9 Hz, 1H), 8.04 (d, *J* = 8.3 Hz, 1H), 7.93 – 7.87 (m, 2H), 7.80 (d, *J* = 8.1 Hz, 2H), 7.53 (d, *J* = 7.9 Hz, 1H), 7.48 (d, *J* = 8.2 Hz, 1H), 7.42 (d, *J* = 8.8 Hz, 1H), 7.23 (s, 1H), 7.14 (t, *J* = 7.7 Hz, 1H), 6.93 (d, *J* = 8.0 Hz, 1H), 6.74 (t, *J* = 7.4 Hz, 1H), 6.43 – 6.38 (m, 2H), 5.91 (s, 1H).

^{13}C NMR (101 MHz, DMSO-*d_6*) *δ* 154.21, 149.10, 144.47, 138.19, 135.83, 132.28, 132.24, 130.79, 130.76, 130.72, 130.67, 129.63, 128.72, 128.03, 127.46, 127.34, 126.77, 126.02, 125.41, 125.30, 125.16, 124.98, 124.36, 123.60, 123.51, 121.10, 118.95, 118.70, 118.47, 116.90, 110.99.

HRMS (ESI) m/z: $[M+H]^+$ Calcd for $C_{35}H_{22}BrN_2^+$ 549.0961; Found 549.0963.

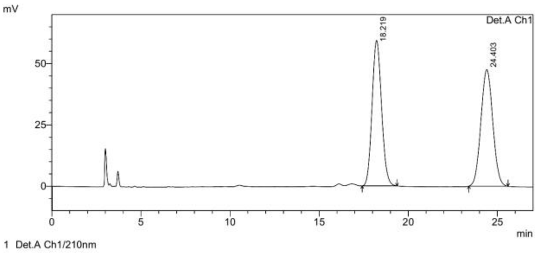

PeakTable

Detector A Ch1 210nm

Peak#	Ret. Time	Area	Height	Area %	Height %
1	18.219	2200896	59298	49.955	55.666
2	24.403	2204861	47226	50.045	44.334
Total		4405757	106524	100.000	100.000

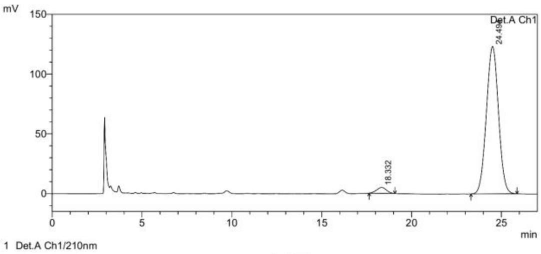

PeakTable

Detector A Ch1 210nm

Peak#	Ret. Time	Area	Height	Area %	Height %
1	18.332	186789	4911	3.133	3.835
2	24.494	5775298	123131	96.867	96.165
Total		5962088	128042	100.000	100.000

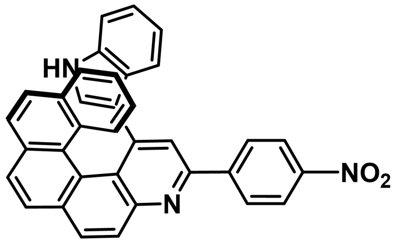

1-(1H-indol-3-yl)-3-(4-nitrophenyl)phenanthro[4,3-f]quinoline (**5e**)

Yellow solid, 34.5 mg, 67% yield with 96% ee. $[\alpha]_D^{24}$= - 1787.2 (c = 0.1, acetone).

HPLC separation Chiralpak AD-H, i-PrOH / hexane = 1 / 2, 1.0 mL/min, 210 nm; tr (minor) = 7.6 min, tr(major) = 8.2 min

^{1}H NMR (400 MHz, DMSO-*d_6*) *δ* 10.74 (s, 1H), 8.71 (d, *J* = 8.4 Hz, 2H), 8.43 (d, *J* = 8.4 Hz, 2H), 8.34 (d, *J* = 8.7 Hz, 1H), 8.22 (d, *J* = 8.7 Hz, 1H), 8.11 (d, *J* = 8.1 Hz, 1H), 8.07 – 7.99 (m, 2H), 7.92 (d, *J* = 8.1 Hz, 1H), 7.54 (d, *J* = 7.9 Hz, 1H), 7.48 (d, *J* = 8.6 Hz, 1H), 7.42 (d, *J* = 8.5 Hz, 1H), 7.24 (t, *J* = 7.4 Hz, 1H), 7.15 (t, *J* = 7.6 Hz, 1H), 6.93 (d, *J* = 8.1 Hz, 1H), 6.74 (t, *J* = 7.5 Hz, 1H), 6.47 (s, 1H), 6.37 (t, *J* = 7.6 Hz, 1H), 5.89 (s, 1H).

^{13}C NMR (101 MHz, DMSO-*d_6*) *δ* 152.96, 149.19, 148.35, 145.06, 144.71, 135.86, 132.52, 131.05, 130.84, 130.60, 128.73, 128.38, 128.08, 127.49, 127.42, 126.78, 126.13, 125.48, 125.19, 124.99, 124.82, 124.49, 123.43, 121.16, 119.54, 119.02, 118.41, 116.84, 111.02.

HRMS (ESI) m/z: $[M+H]^+$ Calcd for $C_{35}H_{22}N_3O_2^+$ 516.1707; Found 516.1711.

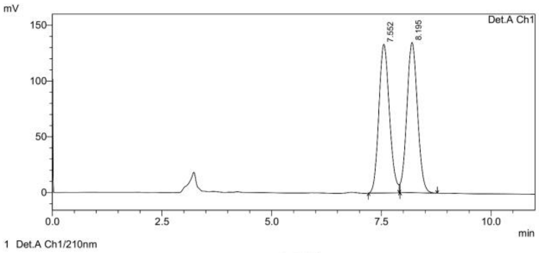

PeakTable

Detector A Ch1 210nm

Peak#	Ret. Time	Area	Height	Area %	Height %
1	7.552	2238014	133294	49.558	49.759
2	8.195	2277949	134585	50.442	50.241
Total		4515963	267880	100.000	100.000

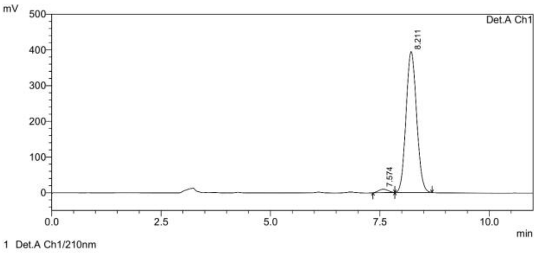

PeakTable

Detector A Ch1 210nm

Peak#	Ret. Time	Area	Height	Area %	Height %
1	7.574	138434	9397	2.048	2.333
2	8.211	6620709	393484	97.952	97.667
Total		6759143	402882	100.000	100.000

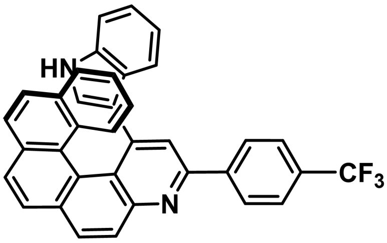

1-(1H-indol-3-yl)-3-(4-(trifluoromethyl)phenyl)phenanthro[4,3-f]quinoline (**5f**)

Yellow solid, 36.0 mg, 65% yield with 96% ee. $[\alpha]_D^{24}$= - 967.3 (c = 0.1, acetone).

HPLC separation Chiralpak AD-H, i-PrOH / hexane = 1 / 9, 1.0 mL/min, 210 nm; tr (minor) = 11.5 min, tr(major) = 16.8 min

^{1}H NMR (400 MHz, DMSO-d_6) δ 10.71 (s, 1H), 8.57 (d, J = 8.4 Hz, 2H), 8.32 (d, J = 8.6 Hz, 1H), 8.20 (d, J = 8.7 Hz, 1H), 8.10 (d, J = 8.1 Hz, 1H), 8.04 (d, J = 8.4 Hz, 1H), 7.94 – 7.87 (m, 2H), 7.60 (d, J = 8.3 Hz, 2H), 7.56 – 7.45 (m, 2H), 7.42 (d, J = 8.5 Hz, 1H), 7.22 (t, J = 7.4 Hz, 1H), 7.13 (t, J = 7.7 Hz, 1H), 6.94 (d, J = 8.1 Hz, 1H), 6.74 (t, J = 7.5 Hz, 1H), 6.46 – 6.31 (m, 2H), 5.95 (s, 1H).

^{13}C NMR (101 MHz, DMSO-d_6) δ 153.98, 149.73, 149.12, 144.45, 138.24, 135.84, 132.27, 130.80, 130.75, 130.72, 130.66, 129.60, 128.71, 128.07, 128.03, 127.45, 127.34, 126.76, 126.02, 125.40, 125.27, 125.16, 124.97, 124.31, 123.52, 121.72, 121.11, 120.63(q, J = 255.2 Hz), 118.96, 118.80, 118.50, 116.87, 111.00.

^{19}F NMR (376 MHz, DMSO-d_6) δ -56.59.

HRMS (ESI) m/z: $[M+H]^+$ Calcd for $C_{36}H_{22}F_3N_2^+$ 539.1730; Found 539.1734.

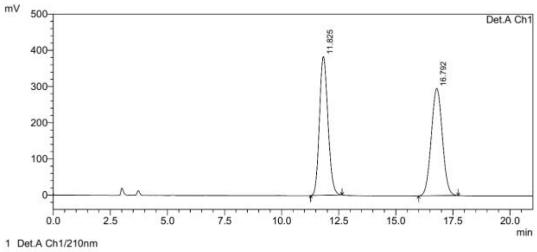

PeakTable

Detector A Ch1 210nm

Peak#	Ret. Time	Area	Height	Area %	Height %
1	11.825	9907332	383279	49.765	56.450
2	16.792	10000971	295698	50.235	43.550
Total		19908303	678977	100.000	100.000

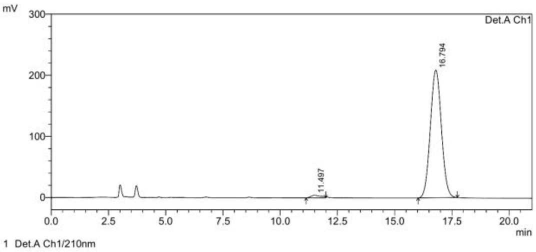

PeakTable

Detector A Ch1 210nm

Peak#	Ret. Time	Area	Height	Area %	Height %
1	11.497	130462	4205	1.823	1.974
2	16.794	7025913	208815	98.177	98.026
Total		7156375	213019	100.000	100.000

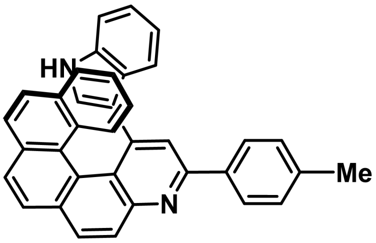

1-(1H-indol-3-yl)-3-(p-tolyl)phenanthro[4,3-f]quinoline (**5g**)

Yellow solid, 19.4 mg, 40% yield with 97% ee. $[\alpha]_D^{24}$= - 1152.7 (c = 0.1, acetone).

HPLC separation Chiralpak AD-H, i-PrOH / hexane = 1 / 9, 1.0 mL/min, 210 nm; tr (minor) = 15.6 min, tr(major) = 30.8 min

^{1}H NMR (400 MHz, DMSO-*d_6*) *δ* 10.66 (s, 1H), 8.34 (d, *J* = 7.9 Hz, 2H), 8.29 (d, *J* = 8.6 Hz, 1H), 8.18 (d, *J* = 8.6 Hz, 1H), 8.08 (d, *J* = 8.2 Hz, 1H), 8.04 (d, *J* = 8.3 Hz, 1H), 7.88 (d, *J* = 8.1 Hz, 1H), 7.83 (s, 1H), 7.56 – 7.32 (m, 5H), 7.21 (t, *J* = 7.4 Hz, 1H), 7.13 (t, *J* = 7.8 Hz, 1H), 6.92 (d, *J* = 7.9 Hz, 1H), 6.73 (t, *J* = 7.5 Hz, 1H), 6.44 – 6.30 (m, 2H), 5.94 (s, 1H), 2.43 (s, 3H).

^{13}C NMR (101 MHz, DMSO-*d_6*) *δ* 155.47, 149.15, 144.17, 139.56, 136.28, 135.83, 132.10, 130.73, 130.47, 129.96, 128.80, 128.01, 127.84, 127.51, 127.46, 127.28, 126.76, 125.96, 125.37, 125.17, 125.00, 124.76, 124.08, 123.60, 121.07, 118.93, 118.62, 118.53, 116.97, 110.99, 21.44.

HRMS (ESI) m/z: $[M+H]^+$ Calcd for $C_{36}H_{25}N_2^+$ 485.2012; Found 485.2017.

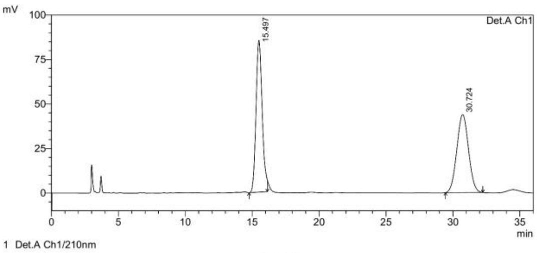

PeakTable

Detector A Ch1 210nm

Peak#	Ret. Time	Area	Height	Area %	Height %
1	15.497	2735815	85281	50.688	66.072
2	30.724	2661564	43792	49.312	33.928
Total		5397379	129073	100.000	100.000

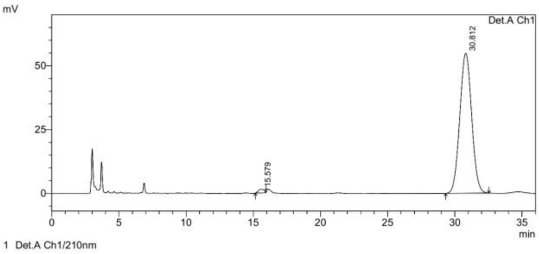

PeakTable

Detector A Ch1 210nm

Peak#	Ret. Time	Area	Height	Area %	Height %
1	15.579	47637	1449	1.392	2.575
2	30.812	3374080	54829	98.608	97.425
Total		3421717	56278	100.000	100.000

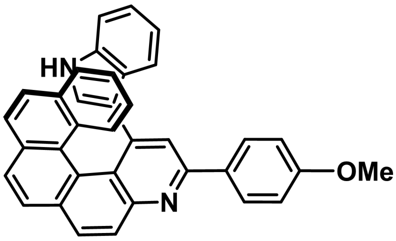

1-(1H-indol-3-yl)-3-(4-methoxyphenyl)phenanthro[4,3-f]quinoline (**5h**)

Yellow solid, 33.5 mg, 67% yield with 90% ee. $[\alpha]_D^{24}$= - 871.7 (c = 0.1, acetone).

HPLC separation Chiralpak AD-H, i-PrOH / hexane = 1 / 9, 1.0 mL/min, 210 nm; tr (minor) = 25.4 min, tr(major) = 41.0 min

^{1}H NMR (400 MHz, DMSO-*d_6*) *δ* 10.64 (s, 1H), 8.65 – 8.32 (m, 2H), 8.28 (d, *J* = 8.7 Hz, 1H), 8.16 (d, *J* = 9.2 Hz, 1H), 8.08 (d, *J* = 8.2 Hz, 1H), 8.04 (d, *J* = 8.1 Hz, 1H), 7.87 (d, *J* = 8.3 Hz, 1H), 7.80 (s, 1H), 7.55 – 7.45 (m, 2H), 7.41 (d, *J* = 8.7 Hz, 1H), 7.24 – 7.09 (m, 4H), 6.91 (d, *J* = 8.0 Hz, 1H), 6.73 (t, *J* = 7.4 Hz, 1H), 6.47 – 6.26 (m, 2H), 5.95 (s, 1H), 3.88 (s, 3H).

^{13}C NMR (101 MHz, DMSO-*d_6*) *δ* 161.04, 155.24, 149.10, 144.08, 135.79, 131.99, 131.48, 130.74, 130.68, 130.41, 129.04, 128.72, 127.96, 127.72, 127.43, 127.25, 126.76, 125.93, 125.41, 125.36, 125.16, 124.98, 124.72, 123.79, 123.60, 121.05, 118.89, 118.51, 118.28, 116.95, 114.73, 113.70, 110.97, 55.79.

HRMS (ESI) m/z: $[M+H]^+$ Calcd for $C_{36}H_{25}N_2O^+$ 501.1961; Found 501.1966.

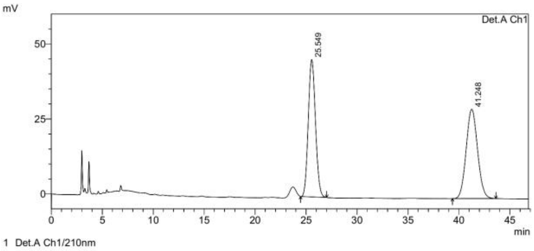

PeakTable

Detector A Ch1 210nm

Peak#	Ret. Time	Area	Height	Area %	Height %
1	25.549	2252759	45789	49.363	60.591
2	41.248	2310925	29782	50.637	39.409
Total		4563684	75571	100.000	100.000

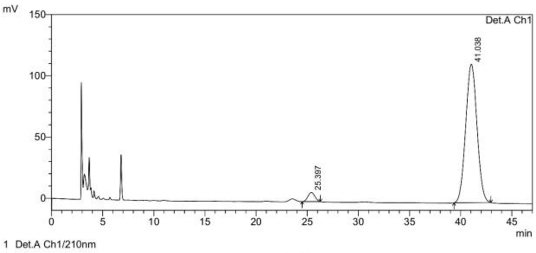

PeakTable

Detector A Ch1 210nm

Peak#	Ret. Time	Area	Height	Area %	Height %
1	25.397	351553	7481	3.884	6.210
2	41.038	8698875	112992	96.116	93.790
Total		9050428	120473	100.000	100.000

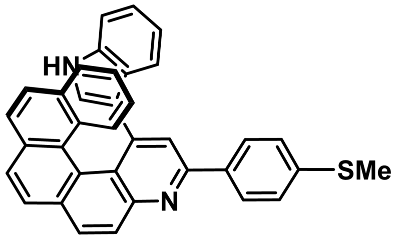

1-(1H-indol-3-yl)-3-(4-(methylthio)phenyl)phenanthro[4,3-f]quinoline (5i)

Yellow solid, 24.3 mg, 47% yield with 98% ee. $[\alpha]_D^{24}$= - 928.4 (c = 0.1, acetone).

HPLC separation Chiralpak AD-H, i-PrOH / hexane = 1 / 9, 1.0 mL/min, 210 nm; tr (minor) = 26.1 min, tr(major) = 41.3 min

^{1}H NMR (400 MHz, DMSO-*d$_6$*) *δ* 10.67 (s, 1H), 8.39 (d, *J* = 8.1 Hz, 2H), 8.29 (d, *J* = 8.7 Hz, 1H), 8.18 (d, *J* = 8.6 Hz, 1H), 8.08 (d, *J* = 8.2 Hz, 1H), 8.04 (d, *J* = 8.5 Hz, 1H), 7.88 (d, *J* = 8.1 Hz, 1H), 7.84 (s, 1H), 7.54 – 7.45 (m, 4H), 7.41 (d, *J* = 8.5 Hz, 1H), 7.21 (t, *J* = 7.4 Hz, 1H), 7.13 (t, *J* = 7.6 Hz, 1H), 6.92 (d, *J* = 8.1 Hz, 1H), 6.73 (t, *J* = 7.6 Hz, 1H), 6.40 – 6.36 (m, 2H), 5.95 (s, 1H), 2.58 (s, 3H).

^{13}C NMR (101 MHz, DMSO-*d$_6$*) *δ* 154.92, 149.13, 144.21, 140.66, 135.81, 135.42, 132.11, 130.76, 130.71, 130.53, 128.74, 127.99, 127.87, 127.44, 127.28, 126.76, 126.35, 125.97, 125.36, 125.16, 124.98, 124.80, 124.10, 123.57, 121.07, 118.92, 118.47, 116.92, 110.99, 14.92.

HRMS (ESI) m/z: $[M+H]^+$ Calcd for $C_{36}H_{25}N_2S^+$ 517.1733; Found 517.1737.

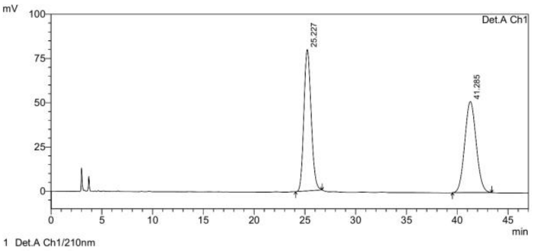

PeakTable

Detector A Ch1 210nm

Peak#	Ret. Time	Area	Height	Area %	Height %
1	25.227	3961359	79835	49.617	60.825
2	41.285	4022527	51418	50.383	39.175
Total		7983887	131253	100.000	100.000

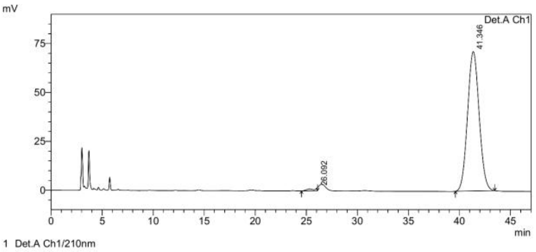

PeakTable

Detector A Ch1 210nm

Peak#	Ret. Time	Area	Height	Area %	Height %
1	26.092	61300	1573	1.077	2.159
2	41.346	5629665	71277	98.923	97.841
Total		5690965	72850	100.000	100.000

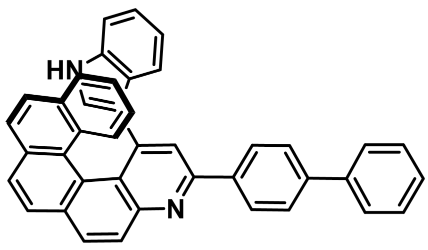

3-([1,1'-biphenyl]-4-yl)-1-(1H-indol-3-yl)phenanthro[4,3-f]quinoline (5j)

Yellow solid, 28.9 mg, 53% yield with 98% ee. $[\alpha]_D^{24}$= - 1473.9 (c = 0.1, acetone).

HPLC separation Chiralpak AD-H, i-PrOH / hexane = 1 / 3, 1.0 mL/min, 210 nm; tr (minor) = 9.8 min, tr(major) = 11.8 min

^{1}H NMR (400 MHz, DMSO-*d$_6$*) *δ* 10.72 (s, 1H), 8.55 (d, *J* = 8.2 Hz, 2H), 8.33 (d, *J* = 8.7 Hz, 1H), 8.23 (d, *J* = 8.6 Hz, 1H), 8.11 (d, *J* = 8.1 Hz, 1H), 8.07 (d, *J* = 8.3 Hz, 1H), 7.96 – 7.87 (m, 4H), 7.82 (d, *J* = 7.5 Hz, 2H), 7.57 – 7.48 (m, 4H), 7.46 – 7.41 (m, 2H), 7.23 (t, *J* = 7.4 Hz, 1H), 7.15 (t, *J* = 7.6 Hz, 1H), 6.94 (d, *J* = 8.0 Hz, 1H), 6.74 (t, *J* = 7.5 Hz, 1H), 6.45 – 6.38 (m, 2H), 5.97 (s, 1H)

^{13}C NMR (101 MHz, DMSO-*d$_6$*) *δ* 154.97, 149.19, 144.26, 141.50, 140.01, 138.01, 135.84, 132.20, 130.79, 130.73, 130.60, 129.52, 128.80, 128.27, 128.18, 128.02, 127.95, 127.57, 127.46, 127.31, 127.20, 126.78, 126.00, 125.40, 125.35, 125.18, 124.99, 124.88, 124.23, 123.58, 121.10, 118.95, 118.75, 118.51, 116.94, 111.01.

HRMS (ESI) m/z: $[M+H]^+$ Calcd for $C_{41}H_{27}N_2^+$ 547.2169; Found 547.2173.

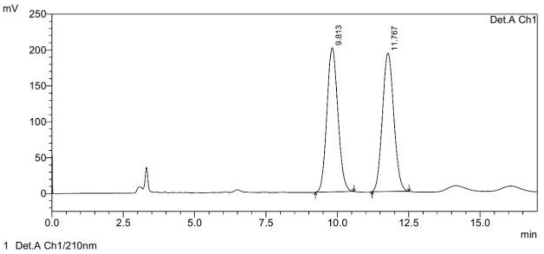

PeakTable

Detector A Ch1 210nm

Peak#	Ret. Time	Area	Height	Area %	Height %
1	9.813	5390460	199963	50.173	50.933
2	11.767	5353182	192640	49.827	49.067
Total		10743642	392603	100.000	100.000

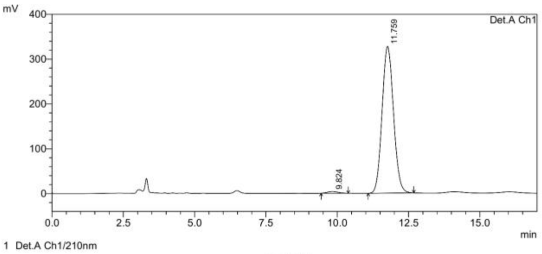

PeakTable

Detector A Ch1 210nm

Peak#	Ret. Time	Area	Height	Area %	Height %
1	9.824	96779	3762	1.049	1.138
2	11.759	9130375	326779	98.951	98.862
Total		9227154	330541	100.000	100.000

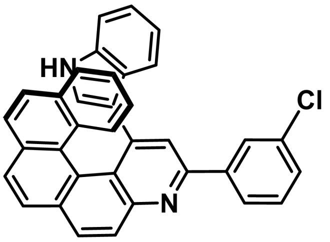

3-(3-chlorophenyl)-1-(1H-indol-3-yl)phenanthro[4,3-f]quinoline (**5k**)

Yellow solid, 30.2 mg, 60% yield with 99% ee. $[\alpha]_D^{24}$= - 961.8 (c = 0.1, acetone).

HPLC separation Chiralpak AD-H, i-PrOH / hexane = 1 / 9, 1.0 mL/min, 210 nm; tr (minor) = 12.2 min, tr(major) = 15.9 min

^{1}H NMR (400 MHz, DMSO-*d$_6$*) *δ* 10.75 (s, 1H), 8.53 (s, 1H), 8.41 (d, *J* = 7.4 Hz, 1H), 8.33 (d, *J* = 8.6 Hz, 1H), 8.23 (d, *J* = 8.6 Hz, 1H), 8.10 (d, *J* = 8.1 Hz, 1H), 8.04 (d, *J* = 8.4 Hz, 1H), 7.94 (s, 1H), 7.90 (d, *J* = 8.2 Hz, 1H), 7.70 – 7.58 (m, 2H), 7.55 (d, *J* = 7.9 Hz, 1H), 7.47 (d, *J* = 8.6 Hz, 1H), 7.41 (d, *J* = 8.5 Hz, 1H), 7.25 (t, *J* = 7.5 Hz, 1H), 7.15 (t, *J* = 7.6 Hz, 1H), 6.93 (d, *J* = 8.0 Hz, 1H), 6.73 (t, *J* = 7.5 Hz, 1H), 6.52 (s, 1H), 6.36 (t, *J* = 7.8 Hz, 1H), 5.83 (s, 1H).

^{13}C NMR (101 MHz, DMSO-*d$_6$*) *δ* 153.73, 149.02, 144.59, 141.15, 135.84, 134.36, 132.27, 131.21,130.81, 130.79, 130.63, 129.68, 128.68, 128.11, 128.04, 127.48, 127.34, 127.23, 126.75, 126.24, 126.05, 125.40, 125.28, 125.21, 125.15, 124.97, 124.49, 123.48, 121.07, 119.00, 118.93, 118.40, 116.92, 110.98.

HRMS (ESI) m/z: $[M+H]^+$ Calcd for $C_{35}H_{22}ClN_2^+$ 505.1466; Found 505.1471.

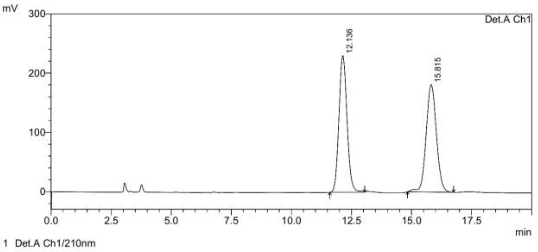

PeakTable

Detector A Ch1 210nm

Peak#	Ret. Time	Area	Height	Area %	Height %
1	12.136	5414863	230585	50.036	56.078
2	15.815	5407010	180600	49.964	43.922
Total		10821874	411184	100.000	100.000

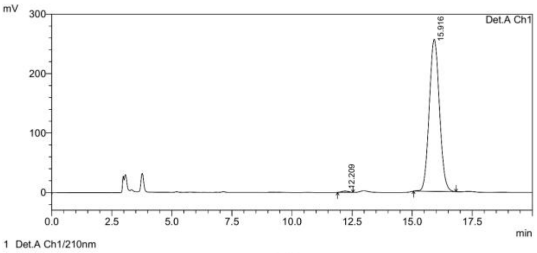

PeakTable

Detector A Ch1 210nm

Peak#	Ret. Time	Area	Height	Area %	Height %
1	12.209	41865	2100	0.550	0.815
2	15.916	7568542	255448	99.450	99.185
Total		7610407	257548	100.000	100.000

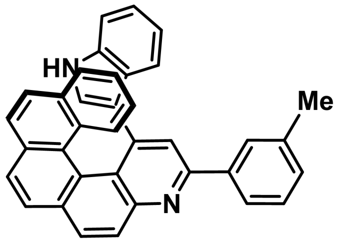

1-(1H-indol-3-yl)-3-(m-tolyl)phenanthro[4,3-f]quinoline (**5l**)

Yellow solid, 24.7 mg, 51% yield with 97% ee. $[\alpha]_D^{24}$= - 685.0 (c = 0.1, acetone).

HPLC separation Chiralpak AD-H, i-PrOH / hexane = 1 / 9, 1.0 mL/min, 210 nm; tr (minor) = 12.6 min, tr(major) = 15.8 min

^{1}H NMR (400 MHz, DMSO-*d$_6$*) *δ* 10.66 (s, 1H), 8.34 (d, *J* = 7.9 Hz, 2H), 8.29 (d, *J* = 8.6 Hz, 1H), 8.18 (d, *J* = 8.6 Hz, 1H), 8.08 (d, *J* = 8.2 Hz, 1H), 8.04 (d, *J* = 8.3 Hz, 1H), 7.88 (d, *J* = 8.1 Hz, 1H), 7.83 (s, 1H), 7.56 – 7.32 (m, 5H), 7.21 (t, *J* = 7.4 Hz, 1H), 7.13 (t, *J* = 7.8 Hz, 1H), 6.92 (d, *J* = 7.9 Hz, 1H), 6.73 (t, *J* = 7.5 Hz, 1H), 6.44 – 6.30 (m, 2H), 5.94 (s, 1H), 2.43 (s, 3H).

^{13}C NMR (101 MHz, DMSO-*d$_6$*) *δ* 155.47, 149.15, 144.17, 139.56, 136.28, 135.83, 132.10, 130.73, 130.47, 129.96, 128.80, 128.01, 127.84, 127.51, 127.46, 127.28, 126.76, 125.96, 125.37, 125.17, 125.00, 124.76, 124.08, 123.60, 121.07, 118.93, 118.62, 118.53, 116.97, 110.99, 21.44.

HRMS (ESI) m/z: $[M+H]^+$ Calcd for $C_{36}H_{25}N_2^+$ 485.2012; Found 485.2017.

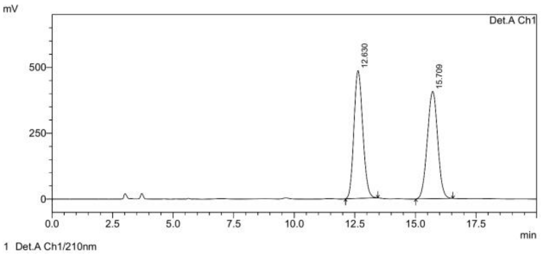

PeakTable

Detector A Ch1 210nm

Peak#	Ret. Time	Area	Height	Area %	Height %
1	12.630	12326204	484448	50.398	54.380
2	15.709	12131314	406401	49.602	45.620
Total		24457518	890849	100.000	100.000

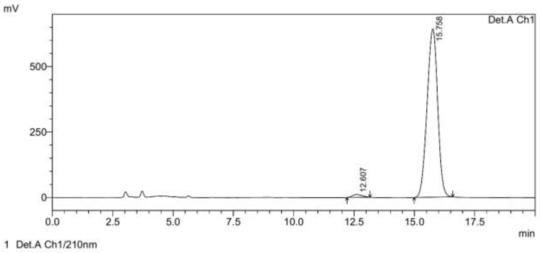

PeakTable

Detector A Ch1 210nm

Peak#	Ret. Time	Area	Height	Area %	Height %
1	12.607	315034	11044	1.581	1.695
2	15.758	19612812	640610	98.419	98.305
Total		19927846	651654	100.000	100.000

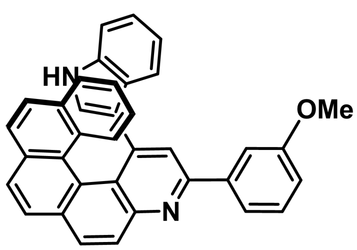

1-(1H-indol-3-yl)-3-(3-methoxyphenyl)phenanthro[4,3-f]quinoline (**5m**)

Yellow solid, 19.0 mg, 37% yield with 96% ee. $[\alpha]_D^{24}$= - 1708.4 (c = 0.1, acetone).

HPLC separation Chiralpak AD-H, i-PrOH / hexane = 1 / 3, 1.0 mL/min, 210 nm; tr (minor) = 5.6 min, tr(major) = 7.3 min

^{1}H NMR (400 MHz, DMSO-*d₆*) *δ* 10.70 (s, 1H), 8.31 (d, *J* = 8.7 Hz, 1H), 8.21 (d, *J* = 8.6 Hz, 1H), 8.10 (d, *J* = 8.1 Hz, 1H), 8.04 (d, *J* = 8.4 Hz, 1H), 8.03 – 7.97 (m, 2H), 7.90 (d, *J* = 8.2 Hz, 1H), 7.88 (s, 1H), 7.57 – 7.49 (m, 2H), 7.48 (d, *J* = 8.5 Hz, 1H), 7.42 (d, *J* = 8.5 Hz, 1H), 7.23 (t, *J* = 7.5 Hz, 1H), 7.19 – 7.09 (m, 2H), 6.93 (d, *J* = 8.1 Hz, 1H), 6.73 (t, *J* = 7.5 Hz, 1H), 6.43 (s, 1H), 6.38 (t, *J* = 9.0 Hz, 1H), 5.90 (s, 1H), 3.93 (s, 3H).

^{13}C NMR (101 MHz, DMSO-d_6) *δ* 160.30, 155.18, 149.05, 144.22, 140.54, 135.82, 132.18, 130.78, 130.72, 130.68, 130.56, 130.44, 128.80, 128.01, 127.96, 127.46, 127.29, 126.75, 126.00, 125.38, 125.31, 125.16, 124.96, 124.91, 124.27, 123.56, 121.07, 120.05, 118.98, 118.92, 118.42, 116.93, 115.60, 112.87, 110.98, 55.76.

HRMS (ESI) m/z: $[M+H]^+$ Calcd for $C_{36}H_{25}N_2O^+$ 501.1961; Found 501.1967.

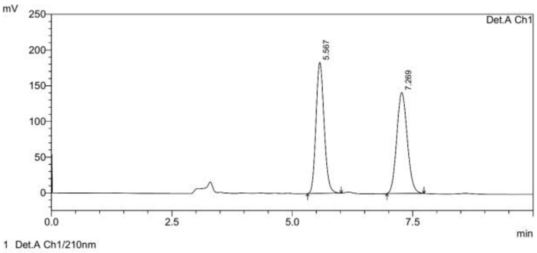

PeakTable

Detector A Ch1 210nm

Peak#	Ret. Time	Area	Height	Area %	Height %
1	5.567	2146107	183370	49.938	56.518
2	7.269	2151453	141075	50.062	43.482
Total		4297561	324445	100.000	100.000

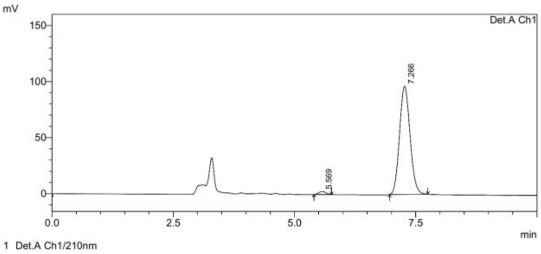

PeakTable

Detector A Ch1 210nm

Peak#	Ret. Time	Area	Height	Area %	Height %
1	5.569	28360	2677	1.906	2.703
2	7.266	1459773	96338	98.094	97.297
Total		1488133	99015	100.000	100.000

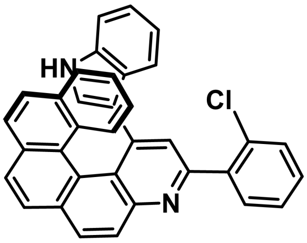

3-(2-chlorophenyl)-1-(1H-indol-3-yl)phenanthro[4,3-f]quinoline (5n)

Yellow solid, 16.1 mg, 32% yield with 84% ee. $[\alpha]_D^{24}$= - 1053.4 (c = 0.1, acetone).

HPLC separation Chiralpak AD-H, i-PrOH / hexane = 1 / 3, 1.0 mL/min, 210 nm; tr (minor) = 9.5 min, tr(major) = 6.8 min

^{1}H NMR (400 MHz, DMSO-d_6) *δ* 10.65 (s, 1H), 8.32 (d, *J* = 8.6 Hz, 1H), 8.16 (d, *J* = 8.6 Hz, 1H), 8.12 (d, *J* = 8.2 Hz, 1H), 8.07 (d, *J* = 8.1 Hz, 1H), 8.00 (d, *J* = 6.9 Hz, 1H), 7.94 (d, *J* = 8.1 Hz, 1H), 7.70 (d, *J* = 7.2 Hz, 1H), 7.63 – 7.53 (m, 4H), 7.47 (t, *J* = 9.8 Hz, 2H), 7.21 – 7.07 (m, 2H), 6.92 (d, *J* = 8.0 Hz, 1H), 6.75 (t, *J* = 7.5 Hz, 1H), 6.46 (t, *J* = 7.7 Hz, 1H), 6.35 – 6.16 (m, 2H).

^{13}C NMR (101 MHz, DMSO-d_6) *δ* 155.70, 148.75, 142.91, 139.32, 135.81, 132.39, 132.30, 132.03, 130.87, 130.80, 130.64, 130.54, 128.51, 128.19, 128.00, 127.97, 127.43, 126.81, 126.12, 125.38, 125.25, 125.06, 124.94, 124.65, 123.65, 123.50, 122.69, 121.24, 119.05, 118.55, 116.38, 111.13.

HRMS (ESI) m/z: $[M+H]^+$ Calcd for $C_{35}H_{22}ClN_2^+$ 505.1466; Found 505.1469.

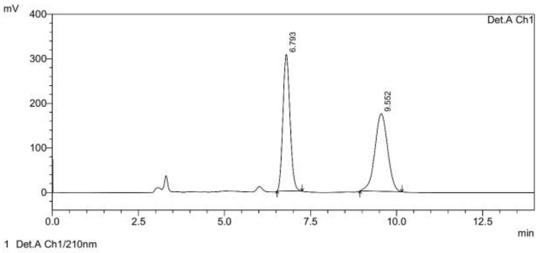

PeakTable

Detector A Ch1 210nm

Peak#	Ret. Time	Area	Height	Area %	Height %
1	6.793	4374580	305355	49.474	63.773
2	9.552	4467544	173457	50.526	36.227
Total		8842125	478812	100.000	100.000

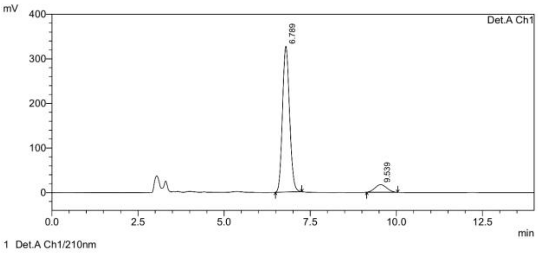

PeakTable

Detector A Ch1 210nm

Peak#	Ret. Time	Area	Height	Area %	Height %
1	6.789	4664747	326528	91.981	95.069
2	9.539	406687	16937	8.019	4.931
Total		5071433	343465	100.000	100.000

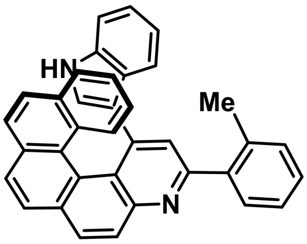

1-(1H-indol-3-yl)-3-(o-tolyl)phenanthro[4,3-f]quinoline (**5o**)

Yellow solid, 18.8 mg, 39% yield with 94% ee. $[\alpha]_D^{24}$= - 1541.7 (c = 0.1, acetone).

HPLC separation Chiralpak AD-H, i-PrOH / hexane = 1 / 9, 1.0 mL/min, 210 nm; tr (minor) = 19.6 min, tr(major) = 14.3 min

^{1}H NMR (400 MHz, DMSO-*d$_6$*) *δ* 10.66 (s, 1H), 8.30 (d, *J* = 8.6 Hz, 1H), 8.16 (d, *J* = 8.3 Hz, 1H), 8.08 (d, *J* = 10.1 Hz, 2H), 7.91 (d, *J* = 7.8 Hz, 1H), 7.79 (s, 1H), 7.55 – 7.35 (m, 7H), 7.27 – 7.08 (m, 2H), 6.92 (d, *J* = 8.0 Hz, 1H), 6.74 (t, *J* = 7.5 Hz, 1H), 6.40 (t, *J* = 7.6 Hz, 1H), 6.29 (s, 1H), 6.02 (s, 1H), 2.63 (s, 3H).

^{13}C NMR (101 MHz, DMSO-*d$_6$*) *δ* 158.65, 148.62, 143.18, 140.44, 136.29, 135.83, 132.17, 131.30, 130.79, 130.67,130.65, 130.35, 130.22, 128.95, 128.60, 127.97, 127.93, 127.44, 127.31, 126.76, 126.46, 126.04, 125.30, 125.20, 125.01, 124.74, 123.52, 123.25, 122.30, 121.12, 118.94, 118.50, 116.62, 111.05, 20.96.

HRMS (ESI) m/z: $[M+H]^+$ Calcd for $C_{36}H_{25}N_2^+$ 485.2012; Found 485.2016.

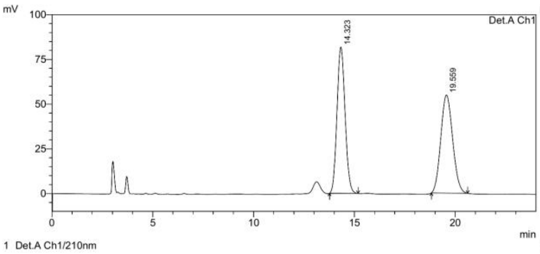

PeakTable

Detector A Ch1 210nm

Peak#	Ret. Time	Area	Height	Area %	Height %
1	14.323	2280311	81596	50.421	59.818
2	19.559	2242197	54812	49.579	40.182
Total		4522508	136409	100.000	100.000

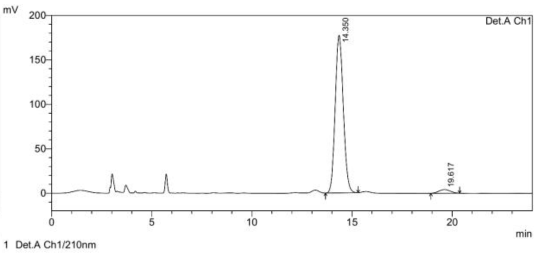

PeakTable

Detector A Ch1 210nm

Peak#	Ret. Time	Area	Height	Area %	Height %
1	14.350	4996995	176946	96.813	97.705
2	19.617	164488	4157	3.187	2.295
Total		5161483	181103	100.000	100.000

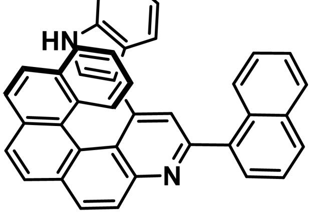

1-(1H-indol-3-yl)-3-(naphthalen-1-yl)phenanthro[4,3-f]quinoline (**5p**)

Yellow solid, 28.6 mg, 55% yield with 92% ee. $[\alpha]_D^{24}$= - 764.2 (c = 0.1, acetone).

HPLC separation Chiralpak AD-H, i-PrOH / hexane = 1 / 9, 1.0 mL/min, 210 nm; tr (minor) = 37.6 min, tr(major) = 18.2 min

^{1}H NMR (400 MHz, DMSO-d_6) δ 10.65 (s, 1H), 8.53 (d, J = 8.2 Hz, 1H), 8.35 (d, J = 8.6 Hz, 1H), 8.20 (d, J = 8.6 Hz, 1H), 8.18 – 8.07 (m, 4H), 7.99 (d, J = 7.1 Hz, 1H), 7.94 (d, J = 8.1 Hz, 1H), 7.72 (t, J = 7.6 Hz, 1H), 7.70 – 7.59 (m, 3H), 7.57 – 7.50 (m, 2H), 7.45 (d, J = 8.6 Hz, 1H), 7.25 – 7.19 (m, 2H), 6.92 (d, J = 8.1 Hz, 1H), 6.73 (t, J = 7.5 Hz, 1H), 6.40 (t, J = 7.4 Hz, 1H), 6.30 (s, 1H), 6.06 (s, 1H).

^{13}C NMR (101 MHz, DMSO-d_6) δ 157.95, 148.91, 143.56, 138.40, 135.85, 134.09, 132.30, 131.34, 130.81, 130.72, 130.58, 129.46, 128.94, 128.67, 128.37, 128.07, 128.02, 127.47, 127.38, 127.24, 126.82, 126.57, 126.10, 126.05, 125.44, 125.23, 125.20, 125.03, 124.75, 123.55, 123.52, 123.09, 121.16, 118.99, 118.51, 116.59, 111.08.

HRMS (ESI) m/z: [M+H]$^+$ Calcd for $C_{39}H_{25}N_2^+$ 521.2012; Found 521.2018.

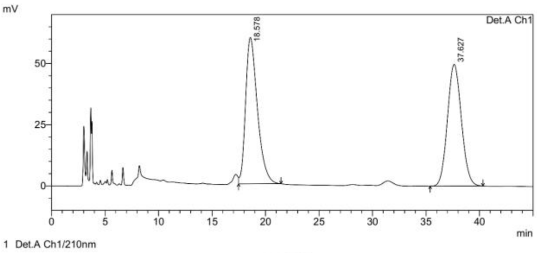

1 Det.A Ch1/210nm

PeakTable

Detector A Ch1 210nm

Peak#	Ret. Time	Area	Height	Area %	Height %
1	18.578	4592204	59750	50.446	54.609
2	37.627	4510930	49664	49.554	45.391
Total		9103133	109414	100.000	100.000

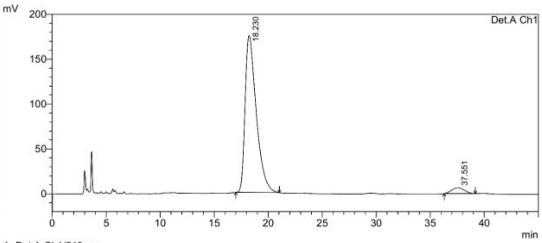

1 Det.A Ch1/210nm

PeakTable

Detector A Ch1 210nm

Peak#	Ret. Time	Area	Height	Area %	Height %
1	18.230	12538493	174759	95.933	96.401
2	37.551	531554	6525	4.067	3.599
Total		13070047	181284	100.000	100.000

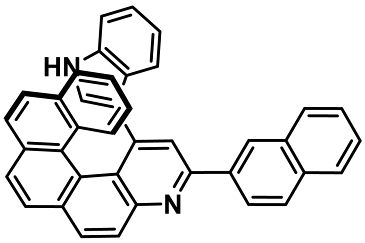

1-(1H-indol-3-yl)-3-(naphthalen-2-yl)phenanthro[4,3-f]quinoline (**5q**)

Yellow solid, 24.0 mg, 46% yield with 98% ee. $[\alpha]_D^{24}$= - 1570.4 (c = 0.1, acetone).

HPLC separation Chiralpak AD-H, i-PrOH / hexane = 1 / 9, 1.0 mL/min, 210 nm; tr (minor) = 19.7 min, tr(major) = 29.0 min

^{1}H NMR (400 MHz, DMSO-d_6) δ 10.71 (s, 1H), 9.00 (s, 1H), 8.66 (d, J = 8.6 Hz, 1H), 8.34 (d, J = 8.6 Hz, 1H), 8.27 (d, J = 9.2 Hz, 1H), 8.20 – 8.00 (m, 6H), 7.90 (d, J = 8.1 Hz, 1H), 7.63 – 7.57 (m, 2H), 7.54 (d, J = 8.0 Hz, 1H), 7.48 (d, J = 8.5 Hz, 1H), 7.42 (d, J = 8.6 Hz, 1H), 7.25 (t, J = 7.4 Hz, 1H), 7.16 (t, J = 7.6 Hz, 1H), 6.94 (d, J = 8.1 Hz, 1H), 6.74 (t, J = 7.6 Hz, 1H), 6.48 (s, 1H), 6.38 (t, J = 7.7 Hz, 1H), 5.91 (s, 1H).

^{13}C NMR (101 MHz, DMSO-d_6) δ 155.33, 149.23, 144.33, 136.46, 135.84, 133.99, 133.68, 132.20, 130.81, 130.76, 130.72, 130.62, 129.26, 128.82, 128.12, 128.06, 127.97, 127.49, 127.36, 127.30, 127.05, 127.01, 126.77, 126.02, 125.41, 125.26, 125.16, 125.03, 124.93, 124.31, 123.58, 121.08, 119.23, 118.94, 118.49, 117.06, 110.98.

HRMS (ESI) m/z: [M+H]$^+$ Calcd for $C_{39}H_{25}N_2^+$ 521.2012; Found 521.2016.

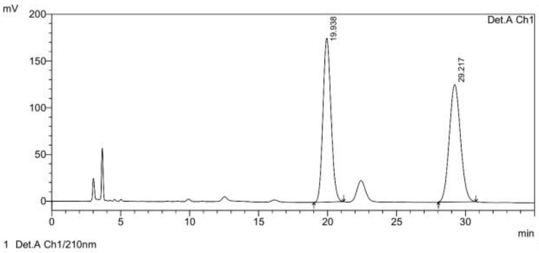

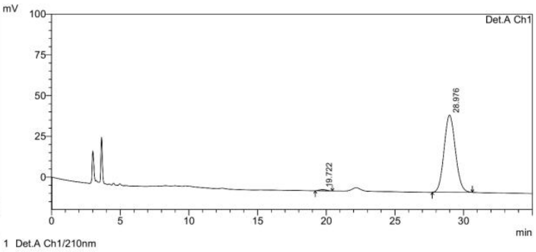

PeakTable

Detector A Ch1 210nm

Peak#	Ret. Time	Area	Height	Area %	Height %
1	19.938	7098927	175064	49.986	58.285
2	29.217	7103030	125294	50.014	41.715
Total		14201958	300358	100.000	100.000

PeakTable

Detector A Ch1 210nm

Peak#	Ret. Time	Area	Height	Area %	Height %
1	19.722	26710	729	0.974	1.517
2	28.976	2714610	47313	99.026	98.483
Total		2741320	48041	100.000	100.000

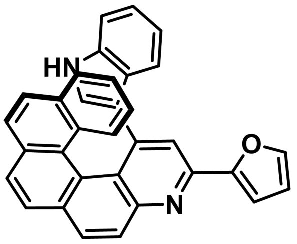

3-(furan-2-yl)-1-(1H-indol-3-yl)phenanthro[4,3-f]quinoline (**5r**)

Yellow solid, 23.0 mg, 50% yield with 95% ee. $[\alpha]_D^{24}$= - 967.3 (c = 0.1, acetone).

HPLC separation Chiralpak AD-H, i-PrOH / hexane = 1 / 3, 1.0 mL/min, 210 nm; tr (minor) = 4.5 min, tr(major) = 6.0 min

^{1}H NMR (400 MHz, DMSO-*d*$_6$) *δ* 10.68 (s, 1H), 8.29 (d, *J* = 8.6 Hz, 1H), 8.13 (d, *J* = 8.6 Hz, 1H), 8.08 (d, *J* = 8.1 Hz, 1H), 8.04 (d, *J* = 8.1 Hz, 1H), 7.99 (s, 1H), 7.89 (d, *J* = 8.1 Hz, 1H), 7.73 (s, 1H), 7.54 – 7.35 (m, 4H), 7.23 – 7.10 (m, 2H), 6.93 (d, *J* = 8.1 Hz, 1H), 6.79 (s, 1H), 6.74 (d, *J* = 7.7 Hz, 1H), 6.43 (t, *J* = 7.4 Hz, 1H), 6.28 (s, 1H), 6.04 (s, 1H).

^{13}C NMR (101 MHz, DMSO-*d*$_6$) *δ* 151.42, 148.95, 145.26, 144.11, 135.78, 132.01, 130.74, 130.71, 129.69, 129.01, 128.16, 127.89, 127.84, 127.47, 127.30, 127.05, 126.74, 125.98, 125.41, 125.13, 124.96, 124.83, 124.19, 123.49, 121.10, 118.94, 118.45, 117.70, 116.78, 114.01, 112.33, 110.97.

HRMS (ESI) m/z: $[M+H]^+$ Calcd for $C_{33}H_{21}N_2O^+$ 461.1648; Found 461.1649.

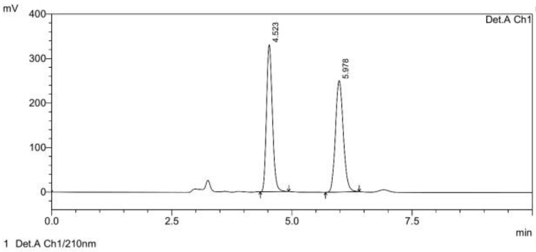

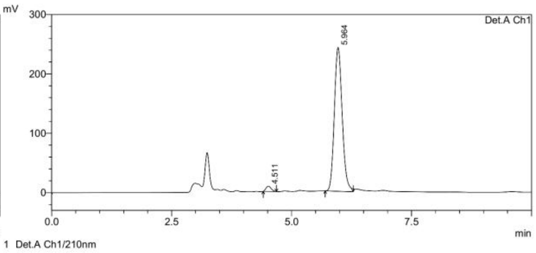

PeakTable

Detector A Ch1 210nm

Peak#	Ret. Time	Area	Height	Area %	Height %
1	4.523	2814433	330651	49.841	56.867
2	5.978	2832337	250800	50.159	43.133
Total		5646769	581452	100.000	100.000

PeakTable

Detector A Ch1 210nm

Peak#	Ret. Time	Area	Height	Area %	Height %
1	4.511	66466	8813	2.344	3.520
2	5.964	2768736	241564	97.656	96.480
Total		2835202	250377	100.000	100.000

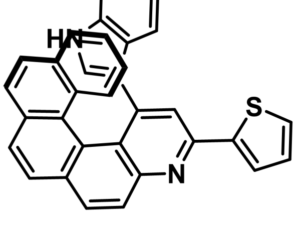

1-(1H-indol-3-yl)-3-(thiophen-2-yl)phenanthro[4,3-f]quinoline (**5s**)

Yellow solid, 19.0 mg, 40% yield with 98% ee. $[\alpha]_D^{24}$= - 1701.4 (c = 0.1, acetone).

HPLC separation Chiralpak AD-H, i-PrOH / hexane = 1 / 9, 1.0 mL/min, 210 nm; tr (minor) = 10.3 min, tr(major) = 21.3 min

^{1}H NMR (400 MHz, DMSO-d_6) *δ* 10.70 (s, 1H), 8.28 (d, *J* = 8.7 Hz, 1H), 8.11 (d, *J* = 8.7 Hz, 1H), 8.11 – 8.01 (m, 3H), 7.87 (d, *J* = 8.2 Hz, 1H), 7.84 (s, 1H), 7.78 (d, *J* = 5.0 Hz, 1H), 7.52 (d, *J* = 7.9 Hz, 1H), 7.46 (d, *J* = 8.6 Hz, 1H), 7.41 (d, *J* = 8.6 Hz, 1H), 7.32 – 7.24 (m, 1H), 7.23 (t, *J* = 7.5 Hz, 1H), 7.15 (t, *J* = 7.6 Hz, 1H), 6.92 (d, *J* = 8.1 Hz, 1H), 6.73 (t, *J* = 7.5 Hz, 1H), 6.39 – 6.35 (m, 2H), 5.88 (s, 1H).

^{13}C NMR (101 MHz, DMSO-d_6) *δ* 151.42, 148.95, 145.26, 144.11, 135.78, 132.01, 130.74, 130.71, 129.69, 129.01, 128.16, 127.89, 127.84, 127.47, 127.30, 127.05, 126.74, 125.98, 125.41, 125.13, 124.96, 124.83, 124.19, 123.49, 121.10, 118.94, 118.45, 117.70, 110.97.

HRMS (ESI) m/z: $[M+H]^+$ Calcd for $C_{33}H_{21}N_2S^+$ 477.1420; Found 477.1424.

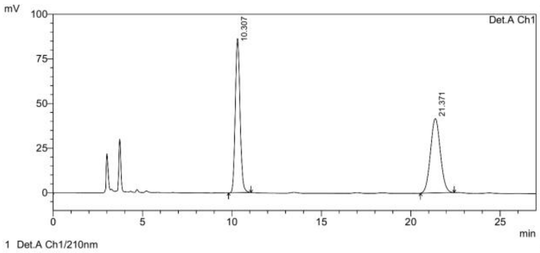

PeakTable

Detector A Ch1 210nm

Peak#	Ret. Time	Area	Height	Area %	Height %
1	10.307	1603356	86391	50.018	67.513
2	21.371	1602221	41571	49.982	32.487
Total		3205577	127962	100.000	100.000

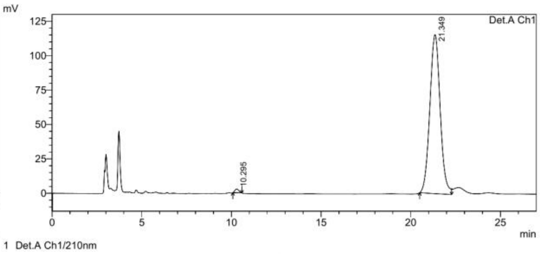

PeakTable

Detector A Ch1 210nm

Peak#	Ret. Time	Area	Height	Area %	Height %
1	10.295	42084	2728	0.900	2.311
2	21.349	4635359	115311	99.100	97.689
Total		4677443	118039	100.000	100.000

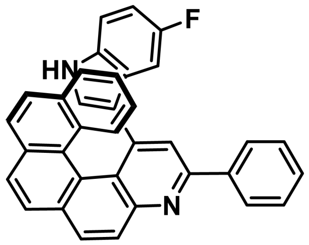

1-(5-fluoro-1H-indol-3-yl)-3-phenylphenanthro[4,3-f]quinoline (**5t**)

Yellow solid, 22.4 mg, 46% yield with 99% ee. $[\alpha]_D^{24}$= - 1173.3 (c = 0.1, acetone).

HPLC separation Chiralpak AD-H, i-PrOH / hexane = 1 / 3, 1.0 mL/min, 210 nm; tr (minor) = 4.9 min, tr(major) = 6.7 min

^{1}H NMR (400 MHz, DMSO-d_6) *δ* 10.84 (s, 1H), 8.46 (d, *J* = 7.3 Hz, 2H), 8.34 (d, *J* = 8.7 Hz, 1H), 8.22 (d, *J* = 8.8 Hz, 1H), 8.14 (d, *J* = 8.3 Hz, 1H), 8.01 (d, *J* = 5.5 Hz, 1H), 7.95 (d, *J* = 8.1 Hz, 1H), 7.87 (d, *J* = 3.1 Hz, 1H), 7.68 – 7.59 (m, 2H), 7.58 – 7.52 (m, 3H), 7.44 (d, *J* = 8.3 Hz, 1H), 7.27 (s, 1H), 7.19 – 7.15 (m, 1H), 7.06 – 6.86 (m, 1H), 6.57 (t, *J* = 9.4 Hz, 2H), 5.43 (s, 1H).

^{13}C NMR (101 MHz, DMSO-d_6) *δ* 156.69 (d, *J* = 231.1 Hz), 155.51, 149.12, 143.60, 138.94, 132.45, 132.23, 130.82, 130.76, 130.63, 130.60, 130.00, 129.34, 128.89, 127.97, 127.89, 127.66, 127.53, 127.36, 126.93, 126.83, 126.01, 125.48, 125.16, 125.12, 124.94, 123.93 (d, *J* = 10.0 Hz),123.91, 118.72, 117.14, 111.91 (d, *J* = 9.8 Hz), 109.18 (d, *J* = 25.8 Hz), 103.31 (d, *J* = 23.7 Hz).

^{19}F NMR (376 MHz, DMSO-d_6) *δ* -125.00.

HRMS (ESI) m/z: $[M+H]^+$ Calcd for $C_{35}H_{22}FN_2^+$ 489.1762; Found 489.1766.

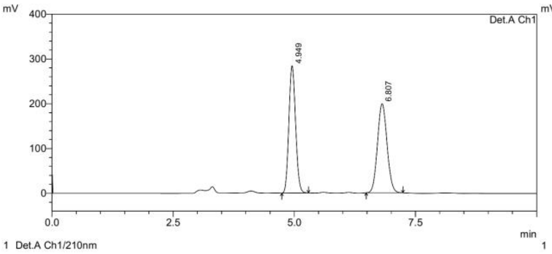

PeakTable

Detector A Ch1 210nm

Peak#	Ret. Time	Area	Height	Area %	Height %
1	4.949	2738233	284602	50.015	58.933
2	6.807	2736595	198325	49.985	41.067
Total		5474828	482927	100.000	100.000

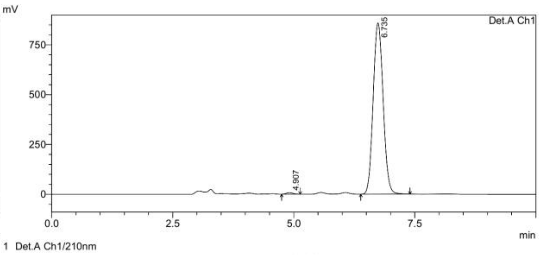

PeakTable

Detector A Ch1 210nm

Peak#	Ret. Time	Area	Height	Area %	Height %
1	4.907	77144	7933	0.622	0.917
2	6.735	12322734	857032	99.378	99.083
Total		12399877	864965	100.000	100.000

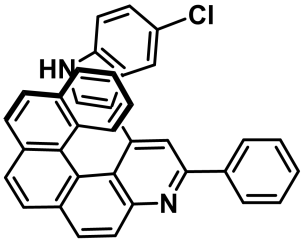

1-(5-chloro-1H-indol-3-yl)-3-phenylphenanthro[4,3-f]quinoline (**5u**)

Yellow solid, 37.9 mg, 75% yield with 98% ee. $[\alpha]_D^{24}$= - 1784.4 (c = 0.1, acetone).

HPLC separation Chiralpak AD-H, i-PrOH / hexane = 1 / 3, 1.0 mL/min, 210 nm; tr (minor) = 4.7 min, tr(major) = 5.4 min

^{1}H NMR (400 MHz, DMSO-d_6) δ 10.96 (s, 1H),8.49 (d, J = 7.6 Hz, 2H), 8.34 (d, J = 8.7 Hz, 1H), 8.25 (d, J = 8.6 Hz, 1H), 8.14 (d, J = 8.2 Hz, 1H), 8.02 (d, J = 8.4 Hz, 1H), 7.95 (d, J = 8.1 Hz, 1H), 7.91 (s, 1H), 7.63 (t, J = 7.5 Hz, 2H), 7.59 – 7.47 (m, 3H), 7.41 (d, J = 8.6 Hz, 1H), 7.30 (t, J = 7.4 Hz, 1H), 7.17 (t, J = 7.7 Hz, 1H), 6.96 (d, J = 8.5 Hz, 1H), 6.75 (d, J = 8.6 Hz, 1H), 6.66 (s, 1H), 5.57 (s, 1H).

^{13}C NMR (101 MHz, DMSO-d_6) δ 155.50, 149.13, 143.31, 138.95, 134.25, 132.24, 130.88, 130.80, 130.60, 130.53, 129.98, 129.33, 128.90, 128.05, 127.88, 127.67, 127.52, 127.30, 126.79, 126.70, 126.01, 125.44, 125.15, 125.09, 125.02, 124.60, 123.99, 123.51, 120.97, 118.95, 118.11, 116.87, 112.35.

HRMS (ESI) m/z: $[M+H]^+$ Calcd for $C_{35}H_{22}ClN_2^+$ 505.1466; Found 505.1474.

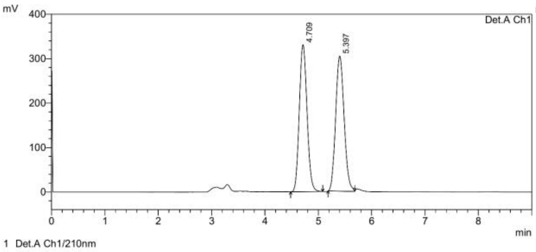

PeakTable

Detector A Ch1 210nm

Peak#	Ret. Time	Area	Height	Area %	Height %
1	4.709	3329863	330710	49.705	52.067
2	5.397	3369394	304454	50.295	47.933
Total		6699257	635164	100.000	100.000

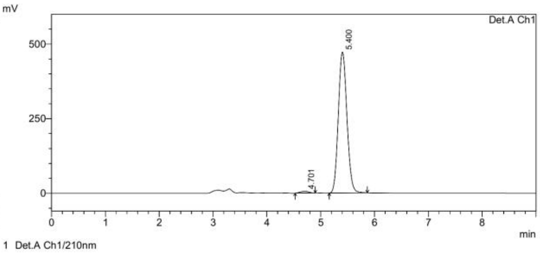

PeakTable

Detector A Ch1 210nm

Peak#	Ret. Time	Area	Height	Area %	Height %
1	4.701	62761	5846	1.179	1.223
2	5.400	5260450	472277	98.821	98.777
Total		5323212	478124	100.000	100.000

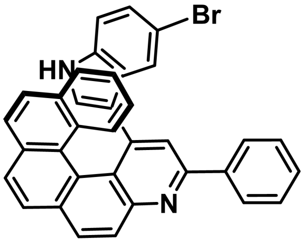

1-(5-bromo-1H-indol-3-yl)-3-phenylphenanthro[4,3-f]quinoline (**5v**)

Yellow solid, 24.1 mg, 44% yield with 98% ee. $[\alpha]_D^{24}$= - 2209.6 (c = 0.1, acetone).

HPLC separation Chiralpak AD-H, i-PrOH / hexane = 1 / 3, 1.0 mL/min, 210 nm; tr (minor) = 4.8 min, tr(major) = 5.3 min

^{1}H NMR (400 MHz, DMSO-d_6) δ 10.95 (s, 1H), 8.47 (d, J = 7.6 Hz, 2H), 8.34 (d, J = 8.7 Hz, 1H), 8.23 (d, J = 8.6 Hz, 1H), 8.14 (d, J = 8.1 Hz, 1H), 7.99 (d, J = 8.4 Hz, 1H), 7.95 (d, J = 8.1 Hz, 1H), 7.89 (s, 1H), 7.62 (t, J = 7.4 Hz, 2H), 7.58 – 7.52 (m, 2H), 7.49 (d, J = 8.6 Hz, 1H), 7.39 (d, J = 8.6 Hz, 1H), 7.30 (t, J = 7.5 Hz, 1H), 7.16 (t, J = 7.6 Hz, 1H), 6.89 (d, J = 8.5 Hz, 1H), 6.83 (d, J = 8.6 Hz, 1H), 6.65 (s, 1H), 5.61 (s, 1H).

^{13}C NMR (101 MHz, DMSO-d_6) δ 155.47, 149.10, 143.26, 138.92, 134.46, 132.22, 130.89, 130.82, 130.61, 130.48, 130.00, 129.33, 128.88, 128.04, 127.94, 127.66, 127.52, 127.28, 126.69, 126.64, 126.03, 125.42, 125.20, 125.12, 125.09, 125.01, 123.96, 123.46, 121.19, 119.00, 116.87, 112.78, 111.60.

HRMS (ESI) m/z: $[M+H]^+$ Calcd for $C_{35}H_{22}BrN_2^+$ 549.0961; Found 549.0962.

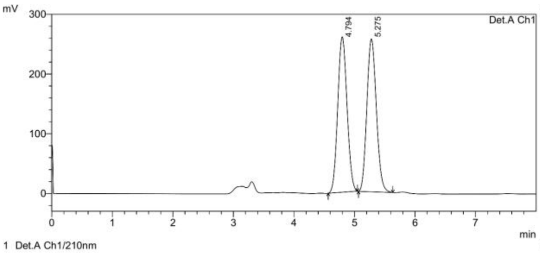

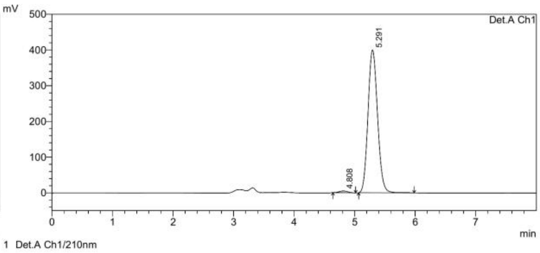

PeakTable

Detector A Ch1 210nm

Peak#	Ret. Time	Area	Height	Area %	Height %
1	4.794	2781360	260065	49.914	50.400
2	5.275	2790962	255935	50.086	49.600
Total		5572322	516000	100.000	100.000

PeakTable

Detector A Ch1 210nm

Peak#	Ret. Time	Area	Height	Area %	Height %
1	4.808	48315	4935	1.091	1.219
2	5.291	4381962	399943	98.909	98.781
Total		4430277	404878	100.000	100.000

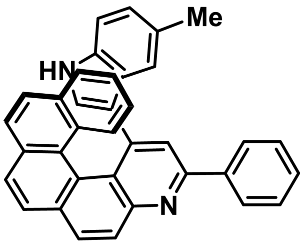

1-(5-methyl-1H-indol-3-yl)-3-phenylphenanthro[4,3-f]quinoline (**5w**)

Yellow solid, 10.2 mg, 21% yield with 99% ee. $[\alpha]_D^{24}$= - 1641.7 (c = 0.1, acetone).

HPLC separation Chiralpak OD-H, i-PrOH / hexane = 1 / 9, 1.0 mL/min, 210 nm; tr (minor) = 17.2 min, tr(major) = 14.3 min

^{1}H NMR (400 MHz, DMSO-d_6) δ 10.59 (s, 1H), 8.45 (d, J = 7.1 Hz, 2H), 8.32 (d, J = 8.7 Hz, 1H), 8.20 (d, J = 8.6 Hz, 1H), 8.12 (d, J = 8.1 Hz, 1H), 8.02 (d, J = 8.4 Hz, 1H), 7.91 (d, J = 8.1 Hz, 1H), 7.87 (s, 1H), 7.62 (t, J = 7.5 Hz, 2H), 7.54 (t, J = 7.5 Hz, 2H), 7.45 (d, J = 8.5 Hz, 1H), 7.36 (d, J = 8.6 Hz, 1H), 7.26 (t, J = 7.5 Hz, 1H), 7.14 (t, J = 7.6 Hz, 1H), 6.78 (d, J = 8.2 Hz, 1H), 6.58 – 6.46 (m, 2H), 5.37 (s, 1H), 1.90 (s, 3H).

^{13}C NMR (101 MHz, DMSO-d_6) δ 155.27, 149.07, 144.41, 139.01, 134.20, 132.14, 130.85, 130.82, 130.56, 129.93, 129.33, 128.68, 128.16, 127.63, 127.58, 127.45, 127.25, 126.90, 126.52, 125.90, 125.59, 125.25, 125.01, 124.91, 124.18, 123.70, 122.54, 118.78, 118.66, 116.70, 110.41, 21.34.

HRMS (ESI) m/z: $[M+H]^+$ Calcd for $C_{36}H_{25}N_2^+$ 485.2012; Found 485.2013.

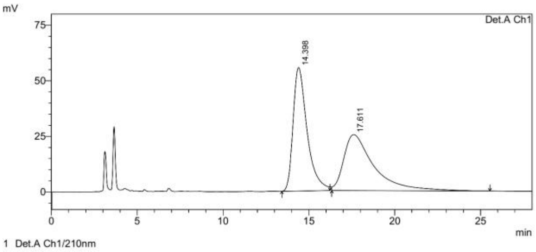

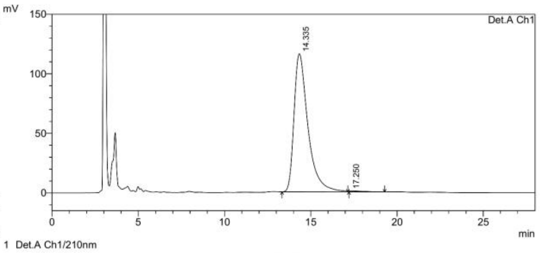

PeakTable

Detector A Ch1 210nm

Peak#	Ret. Time	Area	Height	Area %	Height %
1	14.398	3154371	55543	50.484	68.800
2	17.611	3093939	25188	49.516	31.200
Total		6248310	80731	100.000	100.000

PeakTable

Detector A Ch1 210nm

Peak#	Ret. Time	Area	Height	Area %	Height %
1	14.335	6512755	115977	99.322	99.358
2	17.250	44465	750	0.678	0.642
Total		6557220	116727	100.000	100.000

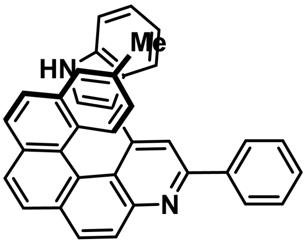

1-(1H-indol-3-yl)-12-methyl-3-phenylphenanthro[4,3-f]quinoline (**5ab**)

Yellow solid, 30.0 mg, 62% yield with 99% ee. $[\alpha]_D^{24}$= - 1692.7 (c = 0.1, acetone).

HPLC separation Chiralpak AD-H, i-PrOH / hexane = 1 / 9, 1.0 mL/min, 210 nm; tr (minor) = 11.3 min, tr(major) = 21.0 min

^{1}H NMR (400 MHz, DMSO-d_6) δ 10.64 (s, 1H), 8.43 (d, J = 7.4 Hz, 2H), 8.29 (d, J = 8.7 Hz, 1H), 8.18 (d, J = 8.6 Hz, 1H), 8.06 (d, J = 8.1 Hz, 1H), 7.96 – 7.84 (m, 3H), 7.63 (t, J = 7.5 Hz, 2H), 7.56 (d, J = 7.1 Hz, 1H), 7.51 (d, J = 8.6 Hz, 1H), 7.37 (d, J = 8.6 Hz, 1H), 7.21 (s, 1H), 6.99 – 6.92 (m, 2H), 6.78 (t, J = 7.6 Hz, 1H), 6.55 (s, 1H), 6.36 (s, 1H), 6.24 (s, 1H), 2.25 (s, 3H).

^{13}C NMR (101 MHz, DMSO-d_6) δ 155.34, 149.15, 144.12, 139.03, 135.86, 135.24, 132.28, 130.94, 130.58, 130.30, 129.96, 129.37, 128.99, 128.69, 127.96, 127.62, 127.59, 127.37, 127.07, 126.49, 126.36, 125.20, 125.16, 124.85,124.37, 124.36, 123.70, 121.07, 121.04, 118.86, 118.84, 118.52, 111.12, 21.33.

HRMS (ESI) m/z: $[M+H]^+$ Calcd for $C_{36}H_{25}N_2^+$ 485.2012; Found 485.2016.

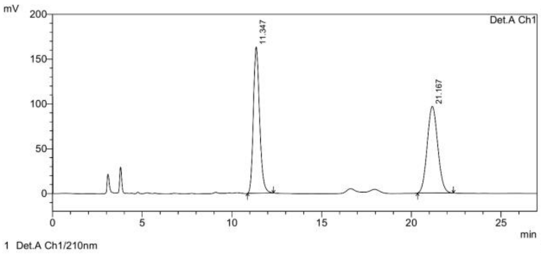

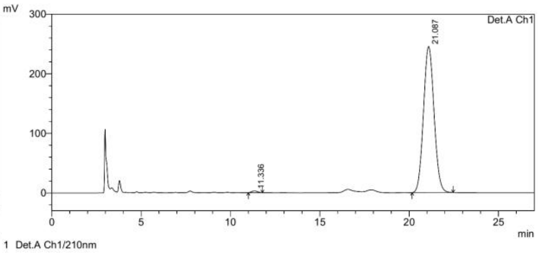

PeakTable

Detector A Ch1 210nm

Peak#	Ret. Time	Area	Height	Area %	Height %
1	11.347	3928888	163056	49.849	62.791
2	21.167	3952725	96623	50.151	37.209
Total		7881613	259679	100.000	100.000

PeakTable

Detector A Ch1 210nm

Peak#	Ret. Time	Area	Height	Area %	Height %
1	11.336	65581	2892	0.647	1.166
2	21.087	10072064	245090	99.353	98.834
Total		10137644	247982	100.000	100.000

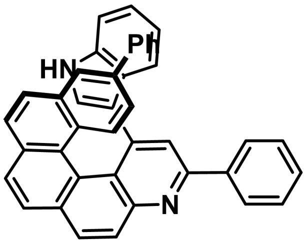

1-(1H-indol-3-yl)-3,12-diphenylphenanthro[4,3-f]quinoline (**5ac**)

Yellow solid, 28.9 mg, 53% yield with 99% ee. $[\alpha]_D^{24}$= - 1923.5 (c = 0.1, acetone).

HPLC separation Chiralpak AD-H, i-PrOH / hexane = 1 / 3, 1.0 mL/min, 210 nm; tr (minor) = 5.8 min, tr(major) = 13.8 min

^{1}H NMR (400 MHz, DMSO-d_6) δ 10.69 (s, 1H), 8.46 (d, J = 7.7 Hz, 2H), 8.31 (d, J = 8.7 Hz, 1H), 8.21 (d, J = 8.6 Hz, 1H), 8.11 (d, J = 8.8 Hz, 1H), 8.10 (d, J = 8.0 Hz, 1H), 7.92 (t, J = 4.1 Hz, 2H), 7.77 (s, 1H), 7.71 (d, J = 7.6 Hz, 2H), 7.62 (t, J = 7.5 Hz, 2H), 7.58 – 7.41 (m, 6H), 7.37 (t, J = 7.3 Hz, 1H), 6.93 (d, J = 8.1 Hz, 1H), 6.69 (t, J = 7.5 Hz, 1H), 6.51 – 6.28 (m, 2H), 6.21 (s, 1H).

^{13}C NMR (101 MHz, DMSO-d_6) δ 155.52, 149.21, 144.21, 140.44, 139.07, 137.73, 135.82, 132.30, 131.18, 130.78, 130.56, 130.02, 129.97, 129.35, 129.16,128.86, 128.85, 127.94, 127.85, 127.73, 127.67, 127.47, 126.84, 125.67, 125.58, 125.33, 124.85, 124.40, 124.17, 123.60, 121.04, 119.07, 118.87, 118.60, 118.58, 116.72, 111.04.

HRMS (ESI) m/z: [M+H]$^+$ Calcd for $C_{41}H_{27}N_2^+$ 547.2169; Found 547.2174.

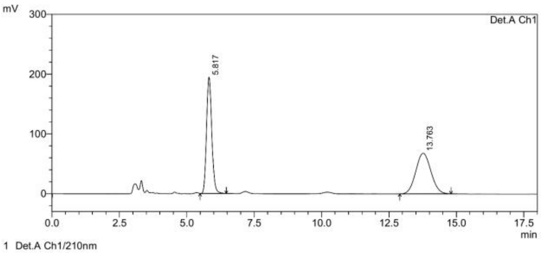

PeakTable

Detector A Ch1 210nm

Peak#	Ret. Time	Area	Height	Area %	Height %
1	5.817	2639594	194075	49.802	74.086
2	13.763	2660630	67884	50.198	25.914
Total		5300224	261959	100.000	100.000

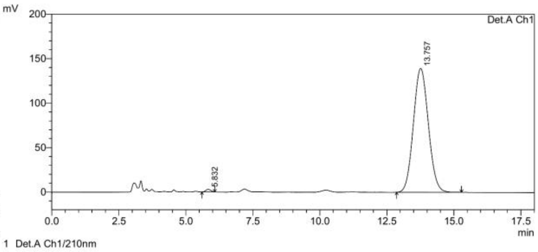

PeakTable

Detector A Ch1 210nm

Peak#	Ret. Time	Area	Height	Area %	Height %
1	5.832	38530	2977	0.706	2.095
2	13.757	5422802	139124	99.294	97.905
Total		5461333	142102	100.000	100.000

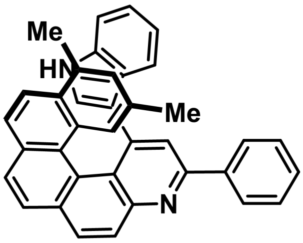

1-(1H-indol-3-yl)-11,13-dimethyl-3-phenylphenanthro[4,3-f]quinoline (**5ad**)

Yellow solid, 24.9 mg, 50% yield with 91% ee. $[\alpha]_D^{24}$= - 1886.8 (c = 0.1, acetone).

HPLC separation Chiralpak AD-H, i-PrOH / hexane = 1 / 9, 1.0 mL/min, 210 nm; tr (minor) = 9.8 min, tr(major) = 12.2 min

^{1}H NMR (400 MHz, DMSO-d_6) δ 10.64 (s, 1H), 8.42 (d, J = 7.6 Hz, 2H), 8.27 (d, J = 8.6 Hz, 1H), 8.17 (d, J = 8.6 Hz, 1H), 8.04 (d, J = 8.2 Hz, 1H), 7.91 – 7.85 (m, 2H), 7.68 (s, 1H), 7.62 (t, J = 7.5 Hz, 2H), 7.59 – 7.40 (m, 3H), 6.97 (d, J = 8.0 Hz, 1H), 6.81 (t, J = 7.5 Hz, 1H), 6.76 (s, 1H), 6.53 (t, J = 7.5 Hz, 1H), 6.34 (s, 1H), 6.22 (s, 1H), 2.36 (s, 3H), 2.16 (s, 3H).

^{13}C NMR (101 MHz, DMSO-d_6) δ 155.04, 148.37, 143.63, 138.76, 135.39, 133.34, 132.42, 131.59, 130.30, 130.01, 129.88, 129.42, 128.91, 128.18, 128.02, 127.41, 127.27, 127.22, 127.09, 126.06, 124.88, 123.85, 123.69, 123.61, 123.36, 122.87, 122.70, 120.63, 118.47, 117.95, 117.74, 115.91, 110.47, 21.35, 19.11.

HRMS (ESI) m/z: [M+H]$^+$ Calcd for $C_{37}H_{27}N_2^+$ 499.2169; Found 499.2173.

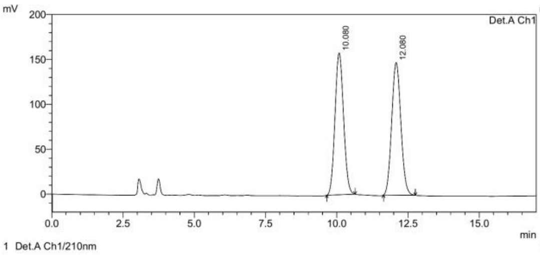

PeakTable

Detector A Ch1 210nm

Peak#	Ret. Time	Area	Height	Area %	Height %
1	10.080	3302848	158117	49.890	51.696
2	12.080	3317414	147744	50.110	48.304
Total		6620262	305860	100.000	100.000

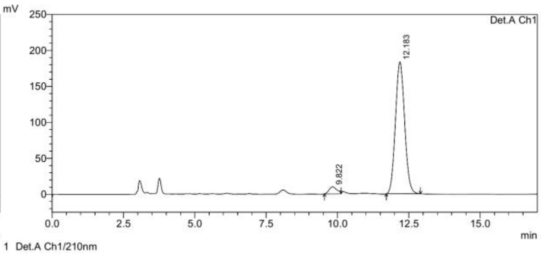

PeakTable

Detector A Ch1 210nm

Peak#	Ret. Time	Area	Height	Area %	Height %
1	9.822	194234	9731	4.506	5.043
2	12.183	4116479	183217	95.494	94.957
Total		4310713	192948	100.000	100.000

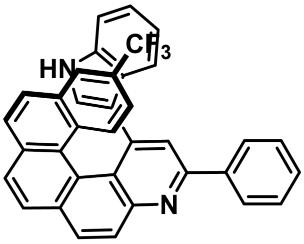

1-(1H-indol-3-yl)-3-phenyl-12-(trifluoromethyl)phenanthro[4,3-f]quinoline (**5ae**)

Yellow solid, 25.8 mg, 48% yield with 99% ee. $[\alpha]_D^{24}$= - 1852.9 (c = 0.1, acetone).

HPLC separation Chiralpak AD-H, i-PrOH / hexane = 1 / 3, 1.0 mL/min, 210 nm; tr (minor) = 4.3 min, tr(major) = 5.3 min

^{1}H NMR (400 MHz, DMSO-*d*$_6$) *δ* 10.74 (s, 1H), 8.46 (d, *J* = 7.6 Hz, 2H), 8.34 (d, *J* = 8.7 Hz, 1H), 8.27 – 8.14 (m, 3H), 7.98 (d, *J* = 8.2 Hz, 1H), 7.96 – 7.89 (m, 2H), 7.71 – 7.50 (m, 6H), 7.38 (d, *J* = 8.9 Hz, 1H), 6.96 (d, *J* = 8.1 Hz, 1H), 6.78 (t, *J* = 7.6 Hz, 1H), 6.49 (s, 1H), 6.27 (s, 1H).

^{13}C NMR (101 MHz, DMSO) *δ* 155.83, 149.38, 143.95, 138.96, 135.84, 132.54, 132.46, 131.44, 130.50, 130.03, 129.85, 129.33, 129.11, 128.13, 127.89, 127.70, 127.33, 127.31, 126.76, 126.31, 126.21, 125.24, 124.85, 123.87, 123.37, 122.18(q, *J* = 256.0 Hz), 121.26, 120.96, 119.35, 118.95, 118.50, 116.55, 111.14.

^{19}F NMR (376 MHz, DMSO-*d*$_6$) *δ* -60.33.

HRMS (ESI) m/z: $[M+H]^+$ Calcd for $C_{36}H_{22}F_3N_2^+$ 539.1730; Found 539.1733.

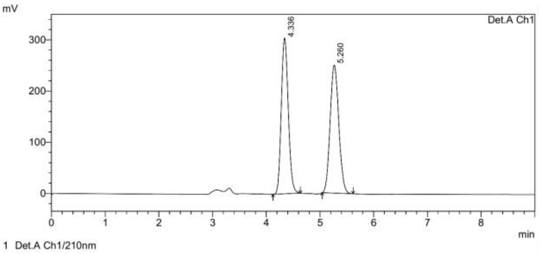

PeakTable

Detector A Ch1 210nm

Peak#	Ret. Time	Area	Height	Area %	Height %
1	4.336	2744715	304475	49.468	54.940
2	5.260	2803719	249724	50.532	45.060
Total		5548433	554198	100.000	100.000

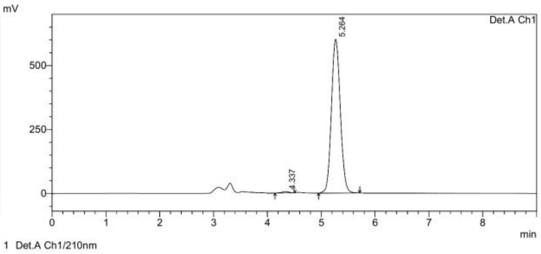

PeakTable

Detector A Ch1 210nm

Peak#	Ret. Time	Area	Height	Area %	Height %
1	4.337	44593	4959	0.643	0.820
2	5.264	6890017	599494	99.357	99.180
Total		6934610	604453	100.000	100.000

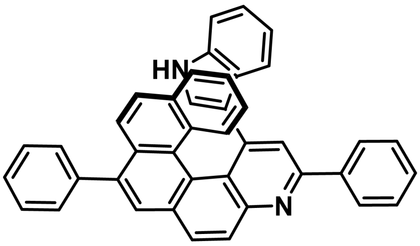

1-(1H-indol-3-yl)-3,8-diphenylphenanthro[4,3-f]quinoline (**5af**)

Yellow solid, 32.2 mg, 59% yield with 86% ee. $[\alpha]_D^{24}$= - 956.6 (c = 0.1, acetone).

HPLC separation Chiralpak AD-H, i-PrOH / hexane = 1 / 3, 1.0 mL/min, 210 nm; tr (minor) = 4.0 min, tr(major) = 6.4 min

^{1}H NMR (400 MHz, DMSO-d_6) *δ* 10.75 (s, 1H), 8.46 (d, *J* = 7.2 Hz, 2H), 8.34 (d, *J* = 8.7 Hz, 1H), 8.24 (d, *J* = 8.6 Hz, 1H), 8.03 (s, 1H), 8.00 (d, *J* = 8.4 Hz, 1H), 7.88 (s, 1H), 7.71 – 7.47 (m, 9H), 7.36 – 7.27 (m, 2H), 7.22 (d, *J* = 8.9 Hz, 1H), 7.16 (t, *J* = 7.7 Hz, 1H), 6.94 (d, *J* = 8.1 Hz, 1H), 6.77 (t, *J* = 7.5 Hz, 1H), 6.57 (s, 1H), 6.37 (t, *J* = 7.6 Hz, 1H), 5.65 (s, 1H).

^{13}C NMR (101 MHz, DMSO-d_6) *δ* 155.45, 148.96, 144.05, 140.34, 139.13, 139.01, 135.94, 131.36, 130.60, 130.42, 130.33, 130.14, 129.95, 129.34, 129.17, 128.94, 128.69, 127.95, 127.64, 127.41, 127.11, 126.24, 125.42, 124.87, 124.77, 124.36, 123.53, 122.12, 121.08, 119.14, 118.60, 118.41, 117.28, 110.98.

HRMS (ESI) m/z: $[M+H]^+$ Calcd for $C_{41}H_{27}N_2^+$ 547.2169; Found 547.2172.

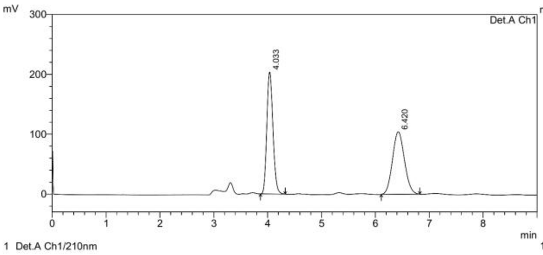

PeakTable

Detector A Ch1 210nm

Peak#	Ret. Time	Area	Height	Area %	Height %
1	4.033	1610285	203606	49.969	66.123
2	6.420	1612277	104316	50.031	33.877
Total		3222561	307922	100.000	100.000

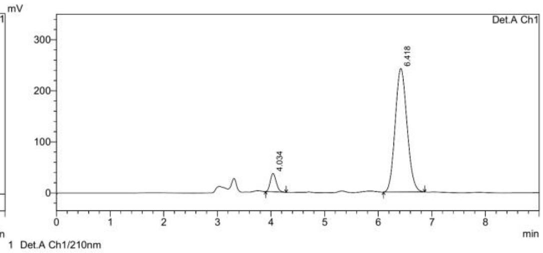

PeakTable

Detector A Ch1 210nm

Peak#	Ret. Time	Area	Height	Area %	Height %
1	4.034	278676	35807	6.972	12.931
2	6.418	3718285	241099	93.028	87.069
Total		3996961	276906	100.000	100.000

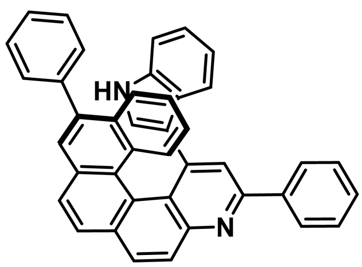

1-(1H-indol-3-yl)-3,10-diphenylphenanthro[4,3-f]quinoline (**5ag**)

Yellow solid, 28.9 mg, 53% yield with 99% ee. $[\alpha]_D^{24}$= - 1433.0 (c = 0.1, acetone).

HPLC separation Chiralpak AD-H, i-PrOH / hexane = 1 / 3, 1.0 mL/min, 210 nm; tr (minor) = 8.2 min, tr(major) = 6.2 min

^{1}H NMR (400 MHz, DMSO-d_6) *δ* 10.91 (s, 1H), 8.46 (d, *J* = 7.6 Hz, 2H), 8.33 (d, *J* = 8.7 Hz, 1H), 8.22 (d, *J* = 8.6 Hz, 1H), 8.12 (d, *J* = 8.1 Hz, 1H), 8.00 (d, *J* = 8.3 Hz, 1H), 7.93 – 7.87 (m, 2H), 7.62 (t, *J* = 7.5 Hz, 2H), 7.59 – 7.53 (m, 3H), 7.47 (t, *J* = 7.3 Hz, 1H), 7.43 – 7.31 (m, 4H), 7.22 – 7.10 (m, 2H), 7.01 (d, *J* = 8.1 Hz, 1H), 6.70 (t, *J* = 7.6 Hz, 1H), 6.45 (s, 1H), 6.31 (s, 1H), 5.77 (s, 1H).

^{13}C NMR (101 MHz, DMSO-d_6) *δ* 155.48, 149.13, 144.03, 140.13, 139.01, 138.08, 135.80, 132.11, 131.27, 130.51, 129.98, 129.84, 129.36, 129.07, 128.77, 127.79, 127.72, 127.63, 127.49, 127.07, 126.12, 125.32, 125.36, 125.10, 125.04, 124.83, 124.21, 123.39, 121.12, 119.08, 118.90, 118.47, 110.80.

HRMS (ESI) m/z: $[M+H]^+$ Calcd for $C_{41}H_{27}N_2^+$ 547.2169; Found 547.2174.

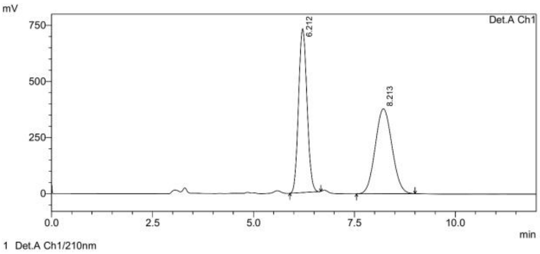

PeakTable

Detector A Ch1 210nm

Peak#	Ret. Time	Area	Height	Area %	Height %
1	6.212	10656121	729623	49.280	65.848
2	8.213	10967425	378418	50.720	34.152
Total		21623546	1108042	100.000	100.000

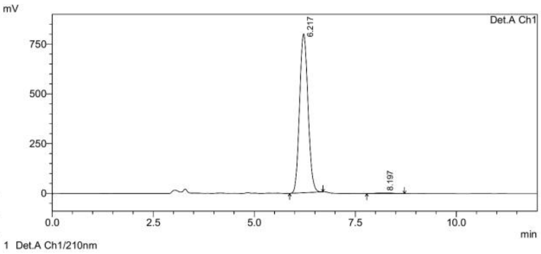

PeakTable

Detector A Ch1 210nm

Peak#	Ret. Time	Area	Height	Area %	Height %
1	6.217	11593047	795469	99.262	99.612
2	8.197	86177	3096	0.738	0.388
Total		11679224	798564	100.000	100.000

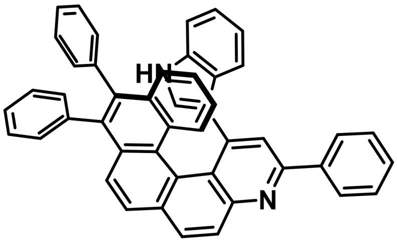

1-(1H-indol-3-yl)-3,9,10-triphenylphenanthro[4,3-f]quinoline (**5ah**)

Yellow solid, 32.3 mg, 52% yield with 99% ee. $[\alpha]_D^{24}$= - 1257.4 (c = 0.1, acetone).

HPLC separation Chiralpak AD-H, i-PrOH / hexane = 1 / 3, 1.0 mL/min, 210 nm; tr (minor) = 4.7 min, tr(major) = 6.4 min

^{1}H NMR (400 MHz, DMSO-d_6) δ 11.00 (s, 1H), 8.47 (d, J = 7.6 Hz, 2H), 8.33 – 8.19 (m, 2H), 7.97 (d, J = 8.4 Hz, 2H), 7.86 (s, 1H), 7.62 (t, J = 7.5 Hz, 2H), 7.57 – 7.50 (m, 1H), 7.34 (t, J = 7.4 Hz, 1H), 7.29 – 7.09 (m, 10H), 7.04 (d, J = 8.2 Hz, 1H), 6.99 (d, J = 7.0 Hz, 1H), 6.94 – 6.83 (m, 2H), 6.67 – 6.58 (m, 2H), 6.33 (s, 1H), 5.44 (s, 1H).

^{13}C NMR (101 MHz, DMSO-d_6) δ 155.53, 149.03, 144.09, 139.35, 139.28, 138.98, 136.28, 136.03, 134.88, 131.66, 131.58, 131.38, 131.16, 130.49, 130.36, 130.24, 130.06, 129.98, 129.33, 129.07, 128.00, 127.92, 127.65, 126.92, 126.82, 126.50, 126.17, 126.12, 125.92, 125.22, 125.09, 125.05, 124.81, 124.67, 123.42, 121.01, 119.50, 118.94, 118.42, 111.21.

HRMS (ESI) m/z: $[M+H]^+$ Calcd for $C_{47}H_{31}N_2^+$ 623.2482; Found 623.2487.

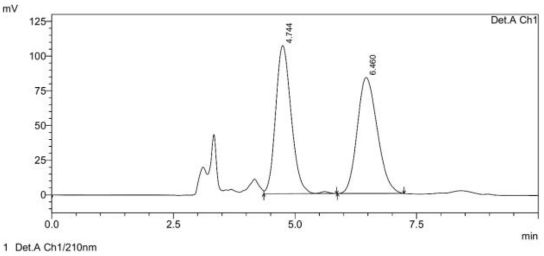

PeakTable

Detector A Ch1 210nm

Peak#	Ret. Time	Area	Height	Area %	Height %
1	4.744	2363928	106582	49.629	56.080
2	6.460	2399312	83470	50.371	43.920
Total		4763240	190052	100.000	100.000

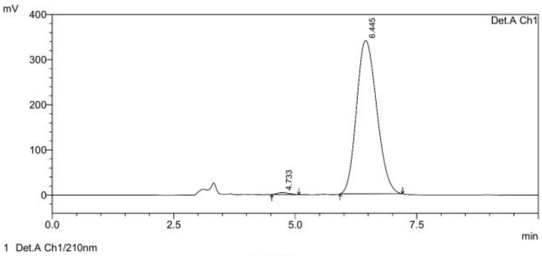

PeakTable

Detector A Ch1 210nm

Peak#	Ret. Time	Area	Height	Area %	Height %
1	4.733	72569	4402	0.732	1.283
2	6.445	9843884	338595	99.268	98.717
Total		9916452	342998	100.000	100.000

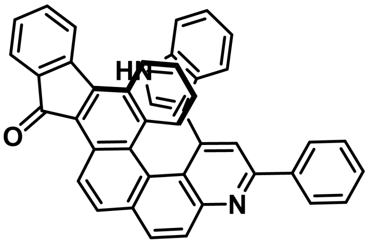

8-(1H-indol-3-yl)-6-phenyl-17H-indeno[1',2':9,10]phenanthro[4,3-f]quinolin-17-one (**5ai**)

Red solid, 25.7 mg, 45% yield with 95% ee. $[\alpha]_D^{24}$= 61.6 (c = 0.1, acetone).

HPLC separation Chiralpak AD-H, i-PrOH / hexane = 1 / 3, 1.0 mL/min, 210 nm; tr (minor) = 10.3 min, tr(major) = 7.1 min

^{1}H NMR (400 MHz, DMSO-*d*$_6$) *δ* 10.86 (s, 1H), 8.84 (d, *J* = 8.3 Hz, 1H), 8.44 (d, *J* = 7.6 Hz, 2H), 8.30 (d, *J* = 8.7 Hz, 1H), 8.26 (d, *J* = 8.4 Hz, 1H), 8.22 (d, *J* = 8.6 Hz, 1H), 8.17 (d, *J* = 8.4 Hz, 1H), 8.01 (d, *J* = 7.5 Hz, 1H), 7.92 (d, *J* = 8.5 Hz, 1H), 7.86 (s, 1H), 7.72 – 7.50 (m, 5H), 7.45 (s, 1H), 7.40 (t, *J* = 7.4 Hz, 1H), 7.33 – 7.25 (m, 1H), 6.66 (d, *J* = 8.1 Hz, 1H), 6.55 – 6.41(m, 2H), 6.24 (s, 1H), 5.67 (s, 1H).

^{13}C NMR (101 MHz, DMSO-*d*$_6$) *δ* 195.22, 155.80, 149.19, 144.39, 143.68, 143.11, 138.87, 135.78, 134.95, 134.64, 134.51, 132.06, 130.24, 130.07, 129.55, 129.48, 129.42, 129.35, 128.86, 128.40, 127.66, 127.48, 126.34, 125.78, 125.58, 125.27, 124.87, 124.36, 124.24, 123.81, 123.52, 123.40, 122.97, 122.36, 120.89, 119.36, 118.92, 118.52, 110.62.

HRMS (ESI) m/z: $[M+H]^+$ Calcd for $C_{42}H_{25}N_2O^+$ 573.1961; Found 573.1967.

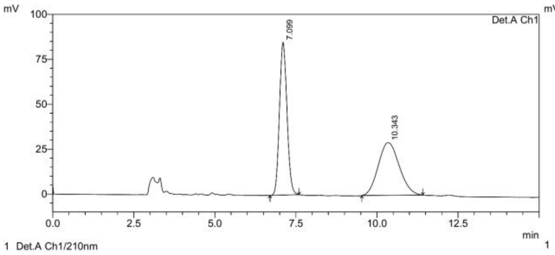

PeakTable

Detector A Ch1 210nm

Peak#	Ret. Time	Area	Height	Area %	Height %
1	7.099	1373917	84780	50.940	74.295
2	10.343	1323225	29333	49.060	25.705
Total		2697141	114112	100.000	100.000

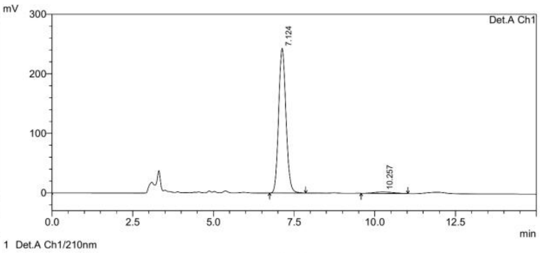

PeakTable

Detector A Ch1 210nm

Peak#	Ret. Time	Area	Height	Area %	Height %
1	7.124	3832507	242015	97.619	99.015
2	10.257	93477	2408	2.381	0.985
Total		3925984	244423	100.000	100.000

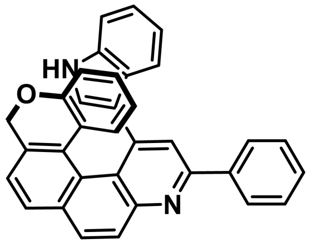

10-(1H-indol-3-yl)-8-phenyl-2H-benzo[3,4]isochromeno[5,6-f]quinoline (**5aj**)

Yellow solid, 21.8 mg, 46% yield with 86% ee. $[\alpha]_D^{24}$= - 575.7 (c = 0.1, acetone).

HPLC separation Chiralpak AD-H, i-PrOH / hexane = 1 / 3, 1.0 mL/min, 210 nm; tr (minor) = 7.4 min, tr(major) = 11.4 min

^{1}H NMR (400 MHz, DMSO-*d*$_6$) *δ* 11.06 (s, 1H), 8.43 (d, *J* = 8.8 Hz, 2H), 8.04 (d, *J* = 8.8 Hz, 1H), 7.92 (d, *J* = 8.9 Hz, 1H), 7.87 (d, *J* = 7.8 Hz, 1H), 7.83 (s, 1H), 7.62 – 7.48 (m, 3H), 7.36 (d, *J* = 7.8 Hz, 1H), 7.20 (d, *J* = 8.0 Hz, 1H), 7.08 (d, *J* = 7.5 Hz, 1H), 6.94 (t, *J* = 7.4 Hz, 1H), 6.80 – 6.57 (m, 5H), 6.43 (d, *J* = 7.9 Hz, 1H), 4.94 (d, *J* = 14.2 Hz, 1H), 4.48 (d, *J* = 14.1 Hz, 1H).

^{13}C NMR (101 MHz, DMSO-*d*$_6$) *δ* 154.90, 153.61, 149.99, 143.84, 138.39, 136.21, 134.97, 133.20, 130.60, 129.40, 129.20, 128.82, 128.00, 127.81, 126.98, 126.06, 125.32, 123.74, 123.72, 123.38, 122.78, 122.32, 121.44, 121.04, 118.78, 117.78, 116.75, 116.46, 111.10, 68.29.

HRMS (ESI) m/z: $[M+H]^+$ Calcd for $C_{34}H_{23}N_2O^+$ 475.1805; Found 475.1809.

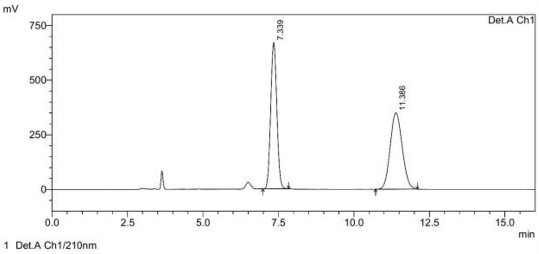

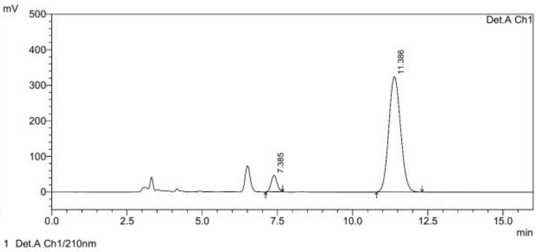

PeakTable

Detector A Ch1 210nm

Peak#	Ret. Time	Area	Height	Area %	Height %
1	7.339	9455506	668578	49.506	65.708
2	11.386	9644077	348915	50.494	34.292
Total		19099583	1017492	100.000	100.000

PeakTable

Detector A Ch1 210nm

Peak#	Ret. Time	Area	Height	Area %	Height %
1	7.385	626376	46441	6.610	12.542
2	11.386	8849284	323846	93.390	87.458
Total		9475661	370287	100.000	100.000

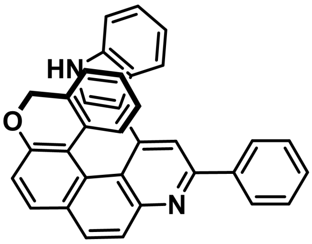

10-(1H-indol-3-yl)-8-phenyl-1H-benzo[3,4]chromeno[5,6-f]quinoline (**5ak**)

Yellow solid, 28.0 mg, 59% yield with 94% ee. $[\alpha]_D^{24}$= - 821.0 (c = 0.1, acetone).

HPLC separation Chiralpak AD-H, i-PrOH / hexane = 1 / 9, 1.0 mL/min, 210 nm; tr (minor) = 14.9 min, tr(major) = 22.9 min

^{1}H NMR (400 MHz, DMSO-d_6) δ 11.10 (s, 1H), 8.40 (d, J = 7.6 Hz, 2H), 8.08 (d, J = 8.8 Hz, 1H), 7.92 (d, J = 8.0 Hz, 2H), 7.85 (s, 1H), 7.59 (t, J = 7.5 Hz, 2H), 7.55 – 7.45 (m, 1H), 7.27 (d, J = 8.1 Hz, 1H), 7.20 – 6.82 (m, 8H), 6.55 (s, 1H), 6.07 (s, 1H), 4.58 (d, J = 11.7 Hz, 1H).

^{13}C NMR (101 MHz, DMSO-d_6) δ 155.36, 154.68, 150.62, 144.37, 138.86, 136.41, 132.21, 131.26, 130.35, 129.91, 129.27, 129.07, 127.76, 127.57, 127.25, 127.12, 126.83, 125.40, 124.80, 124.10, 122.80, 122.48, 122.12, 121.44, 119.65,119.32， 119.10, 117.24, 111.71, 67.41.

HRMS (ESI) m/z: $[M+H]^+$ Calcd for $C_{34}H_{23}N_2O^+$ 475.1805; Found 475.1807.

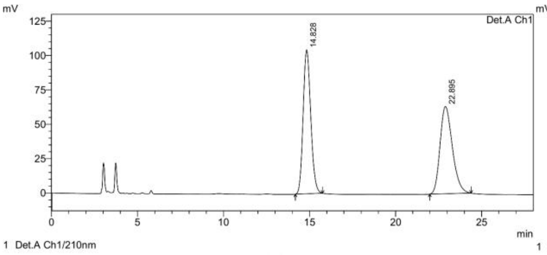

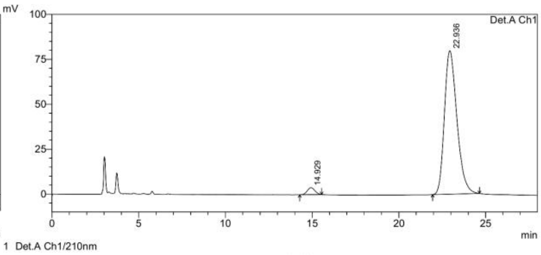

PeakTable

Detector A Ch1 210nm

Peak#	Ret. Time	Area	Height	Area %	Height %
1	14.828	3221518	104600	50.450	62.272
2	22.895	3164071	63374	49.550	37.728
Total		6385589	167974	100.000	100.000

PeakTable

Detector A Ch1 210nm

Peak#	Ret. Time	Area	Height	Area %	Height %
1	14.929	118850	3893	2.890	4.650
2	22.936	3993564	79817	97.110	95.350
Total		4112415	83710	100.000	100.000

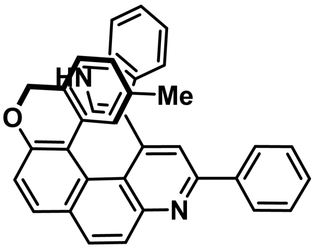

10-(1H-indol-3-yl)-12-methyl-8-phenyl-1H-benzo[3,4]chromeno[5,6-f]quinoline (**5al**)

Yellow solid, 39.2 mg, 81% yield with 97% ee. $[\alpha]_D^{24}$= - 846.0 (c = 0.1, acetone).

HPLC separation Chiralpak AD-H, i-PrOH / hexane = 1 / 3, 1.0 mL/min, 210 nm; tr (minor) = 5.5 min,

tr(major) = 6.1 min

^{1}H NMR (400 MHz, DMSO-d_6) δ 11.06 (s, 1H), 8.38 (d, J = 7.2 Hz, 2H), 8.06 (d, J = 8.9 Hz, 1H), 7.91 (d, J = 2.8 Hz, 1H), 7.89 (d, J = 3.1 Hz, 1H), 7.84 (s, 1H), 7.59 (t, J = 7.5 Hz, 2H), 7.55 – 7.48 (m, 1H), 7.25 (d, J = 8.1 Hz, 1H), 7.11 (d, J = 8.5 Hz, 1H), 7.02 – 6.92 (m, 2H), 6.86 – 6.79 (m, 2H), 6.73 (s, 1H), 6.57 (s, 1H),6.17 (s, 1H), 4.56 (d, J = 11.6 Hz, 1H), 2.06 (s, 3H).

^{13}C NMR (101 MHz, DMSO-d_6) δ 155.56, 154.76, 150.53, 144.46, 139.08, 136.66, 136.52, 131.90, 131.29, 130.19, 129.88, 129.31, 129.06, 127.75, 127.59, 127.19, 126.74, 125.02, 124.51, 124.19, 122.76, 122.63, 122.45, 121.40, 119.49, 119.08, 117.32, 111.67, 67.40, 21.48.

HRMS (ESI) m/z: $[M+H]^+$ Calcd for $C_{35}H_{25}N_2O^+$ 489.1961; Found 489.1967.

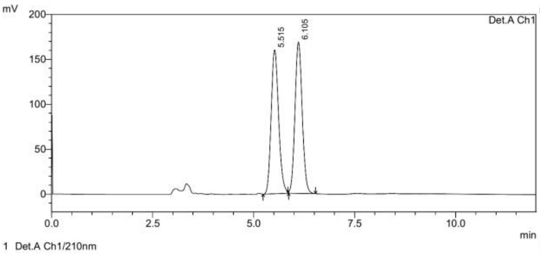

PeakTable

Detector A Ch1 210nm

Peak#	Ret. Time	Area	Height	Area %	Height %
1	5.515	2054522	160184	49.511	48.777
2	6.105	2095097	168217	50.489	51.223
Total		4149619	328401	100.000	100.000

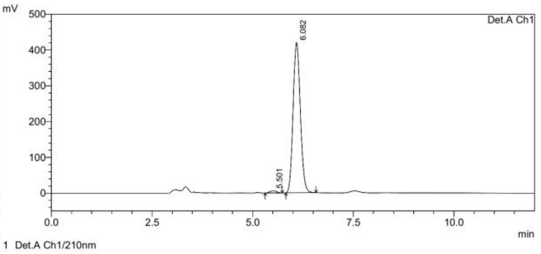

PeakTable

Detector A Ch1 210nm

Peak#	Ret. Time	Area	Height	Area %	Height %
1	5.501	75019	6349	1.403	1.490
2	6.082	5271921	419882	98.597	98.510
Total		5346940	426231	100.000	100.000

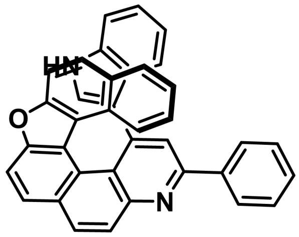

1-(1H-indol-3-yl)-3-phenylnaphtho[2',1':2,3]benzofuro[4,5-f]quinoline (**5am**)

Yellow solid, 23.5 mg, 46% yield with 93% ee. $[\alpha]_D^{24}$= - 635.0 (c = 0.1, acetone).

HPLC separation Chiralpak AD-H, i-PrOH / hexane = 1 / 3, 1.0 mL/min, 210 nm; tr (minor) = 5.2 min, tr(major) = 6.7 min

^{1}H NMR (400 MHz, DMSO-d_6) δ 10.60 (s, 1H), 8.52 – 8.43 (m, 2H), 8.37 (d, J = 8.9 Hz, 1H), 8.26 (d, J = 8.6 Hz, 1H), 8.13 (d, J = 8.8 Hz, 1H), 8.02 – 7.95 (m, 2H), 7.93 (d, J = 8.0 Hz, 1H), 7.77 (d, J = 8.9 Hz, 1H), 7.65 (t, J = 7.5 Hz, 2H), 7.61 – 7.50 (m, 3H), 7.48 (t, J = 7.4 Hz, 1H), 7.31 (t, J = 7.6 Hz, 1H), 6.83 (d, J = 8.1 Hz, 1H), 6.68 (t, J = 7.5 Hz, 1H), 6.30 – 6.19 (m, 2H), 5.59 (s, 1H).

^{13}C NMR (101 MHz, DMSO-d_6) δ 155.57, 154.77, 153.13, 150.09, 145.66, 139.23, 135.56, 131.66, 129.95, 129.51, 129.47, 129.40, 129.36, 129.07, 128.74, 127.73, 127.60, 127.40, 126.22, 125.57, 125,53, 125.25, 124.45, 124.43, 122.74, 121.25, 120.97, 119.67, 119.07, 117.81, 112.30, 111.55, 111.06.

HRMS (ESI) m/z: $[M+H]^+$ Calcd for $C_{37}H_{23}N_2O^+$ 511.1808; Found 511.1810.

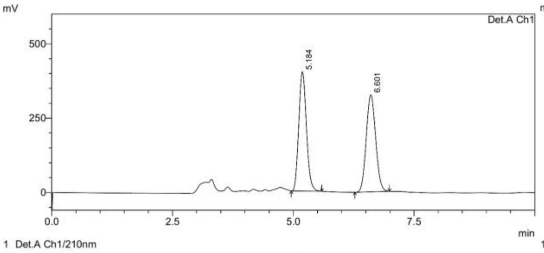

PeakTable

Detector A Ch1 210nm

Peak#	Ret. Time	Area	Height	Area %	Height %
1	5.184	4439655	401081	50.102	55.156
2	6.601	4421551	326096	49.898	44.844
Total		8861207	727177	100.000	100.000

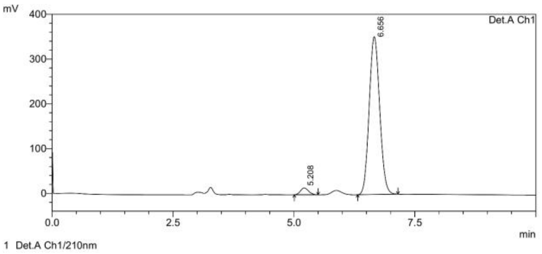

PeakTable

Detector A Ch1 210nm

Peak#	Ret. Time	Area	Height	Area %	Height %
1	5.208	177681	15131	3.232	4.119
2	6.656	5320055	352181	96.768	95.881
Total		5497736	367313	100.000	100.000

The synthesis of 6. To a solution of **5** (0.05 mmol, 23.5 mg) in THF (2 mL) added NaH (0.1 mmol, 4 mg, 60%, dispersion in paraffin liquid) at room temperature for 15 min. Then added Br-R for another 2 h. The reaction was quenched with water (10 mL) and the aqueous layer was extracted with ethyl acetate. The combined organic layers were washed with brine, dried over anhydrous Na_2SO_4, concentrated in vacuo. The crude product was purified by silica column chromatography (Petroleum ether: Ethyl acetate = 10:1) to afford **6**.

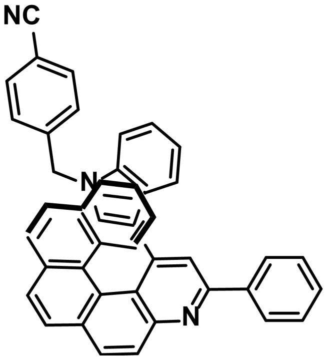

4-((3-(3-phenylphenanthro[4,3-f]quinolin-1-yl)-1H-indol-1-yl)methyl)benzonitrile (**6a**)

Yellow solid, 28.6 mg, 98% yield with 98% ee. $[\alpha]_D^{24}$= - 1612.7 (c = 0.1, acetone).

HPLC separation Chiralpak AD-H, i-PrOH / hexane = 1 / 3, 1.0 mL/min, 210 nm; tr (minor) = 8.5 min, tr(major) = 10.3 min

^{1}H NMR (400 MHz, DMSO-*d*$_6$) *δ* 8.43 (d, *J* = 7.2 Hz, 2H), 8.32 (d, *J* = 8.7 Hz, 1H), 8.21 (d, *J* = 8.6 Hz, 1H), 8.10 (d, *J* = 8.2 Hz, 1H), 8.07 (d, *J* = 8.5 Hz, 1H), 7.91 (d, *J* = 8.1 Hz, 1H), 7.90 – 7.82 (m, 3H), 7.63 (t, *J* = 7.5 Hz, 2H), 7.59 – 7.51 (m, 2H), 7.51 (s, 2H), 7.24 – 7.11 (m, 4 H), 6.84 (d, *J* = 8.2 Hz, 1H), 6.75 (t, *J* = 7.6 Hz, 1H), 6.60 (s, 1H), 6.45 (s, 1H), 6.00 (s, 1H), 5.04 (s, 2H).

^{13}C NMR (101 MHz, DMSO-*d*$_6$) *δ* 155.53, 149.14, 143.56, 143.41, 138.91, 135.76, 132.91, 132.26, 130.82, 130.68, 130.67, 130.59, 130.04, 129.38, 128.82, 128.66, 128.36, 128.17, 127.98, 127.62, 127.50, 127.33, 126.83, 126.07, 125.65, 125.33, 125.22, 124.48, 124.09, 121.67, 119.63, 119.25, 119.02, 118.82, 116.98, 110.64, 109.71, 49.08.

HRMS (ESI) m/z: $[M+H]^+$ Calcd for $C_{43}H_{28}N_3^+$ 586.2278; Found 586.2278.

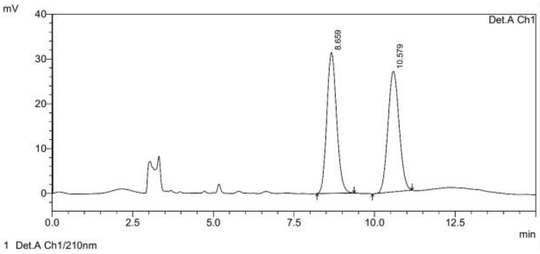

PeakTable

Detector A Ch1 210nm

Peak#	Ret. Time	Area	Height	Area %	Height %
1	8.659	664761	31457	50.633	53.896
2	10.579	648136	26910	49.367	46.104
Total		1312897	58367	100.000	100.000

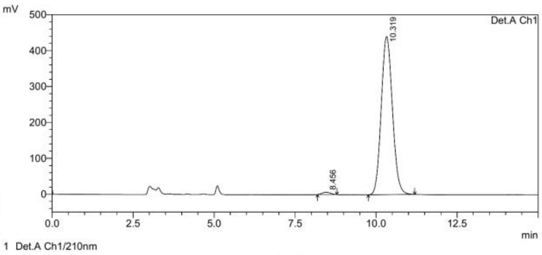

PeakTable

Detector A Ch1 210nm

Peak#	Ret. Time	Area	Height	Area %	Height %
1	8.456	115473	6391	1.080	1.436
2	10.319	10572665	438484	98.920	98.564
Total		10688139	444875	100.000	100.000

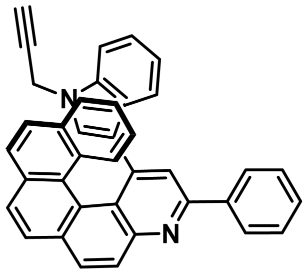

3-phenyl-1-(1-(prop-2-yn-1-yl)-1H-indol-3-yl)phenanthro[4,3-f]quinoline (**6b**)

Yellow solid, 25.1 mg, 99% yield with 98% ee. $[\alpha]_D^{24}$= - 373.8 (c = 0.1, acetone).

HPLC separation Chiralpak AD-H, i-PrOH / hexane = 1 / 3, 1.0 mL/min, 210 nm; tr (minor) = 5.1 min, tr(major) = 6.4 min

^{1}H NMR (400 MHz, DMSO-*d*$_6$) *δ* 8.44 (d, *J* = 7.6 Hz, 2H), 8.32 (d, *J* = 8.7 Hz, 1H), 8.22 (d, *J* = 8.7 Hz, 1H), 8.08 (d, *J* = 8.1 Hz, 1H), 7.96 (d, *J* = 8.4 Hz, 1H), 7.87 (d, *J* = 8.2 Hz, 1H), 7.85 (s, 1H), 7.62 (t, *J* = 7.5 Hz, 2H), 7.57 - 7.51 (m, 2H), 7.43 (d, *J* = 8.6 Hz, 1H), 7.36 (d, *J* = 8.5 Hz, 1H), 7.25 (t, *J* = 7.4 Hz, 1H), 7.14 (t, *J* = 7.6 Hz, 1H), 7.06 (d, *J* = 8.2 Hz, 1H), 6.83 (t, *J* = 7.6 Hz, 1H), 6.41 (s, 2H), 5.83 (s, 1H), 4.61 (d, *J* = 19.2 Hz, 1H), 4.56 (d, *J* = 19.2 Hz, 1H), 3.46 (s, 1H).

^{13}C NMR (101 MHz, DMSO-*d*$_6$) *δ* 155.53, 149.05, 143.19, 138.92, 135.29, 132.14, 130.68, 130.60, 130.45, 130.02, 129.38, 128.80, 127.93, 127.63, 127.50, 127.38, 127.21, 126.73, 125.97, 125.47, 125.19, 125.09, 124.96, 124.33, 124.22, 121.54, 119.63, 118.81, 118.67, 116.80, 109.57, 78.61, 76.61, 35.40.

HRMS (ESI) m/z: $[M+H]^+$ Calcd for $C_{38}H_{25}N_2^+$ 509.2012; Found 509.2010.

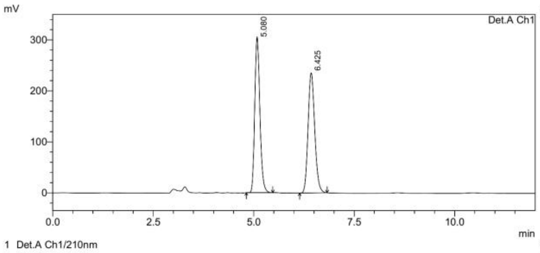

PeakTable

Detector A Ch1 210nm

Peak#	Ret. Time	Area	Height	Area %	Height %
1	5.080	2725288	304495	50.084	56.556
2	6.425	2716186	233898	49.916	43.444
Total		5441473	538393	100.000	100.000

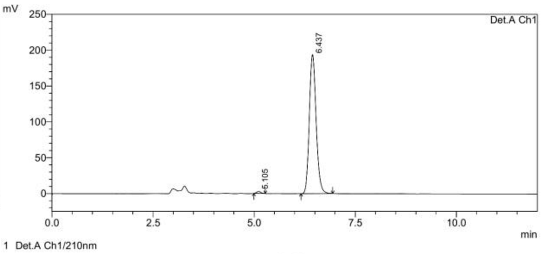

PeakTable

Detector A Ch1 210nm

Peak#	Ret. Time	Area	Height	Area %	Height %
1	5.105	20225	2790	0.883	1.418
2	6.437	2269355	194023	99.117	98.582
Total		2289580	196813	100.000	100.000

1.5. Crystal structure data of *M*-5a and *M*-5al

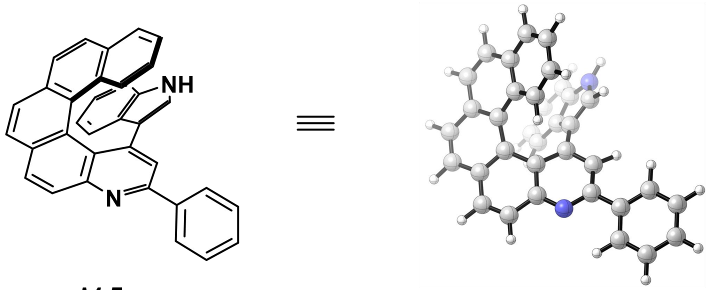

M-**5a**

Supplementary Fig. 1. The X-ray crystal structure of enantiopure *M*-**5a** (**CDCC 2174258**)

The crystal of enantiopure **5a** was obtained through slow vapor diffusion of n-hexane into a EA solution of **5a** at room temperature. The structure and absolute configuration (*M*) of **5a** were then determined by X-ray crystallography.

Supplementary Table 2. Crystal data and structure refinement for *M*-**5a**.

Identification code	p20220501a
Empirical formula	$C_{36}H_{24}N_2O_{0.5}$
Formula weight	492.57
Temperature/K	293.15
Crystal system	monoclinic
Space group	C2
a/Å	14.65711(5)
b/Å	9.40438(4)
c/Å	19.93949(7)
α/°	90
β/°	90.7845(3)
γ/°	90
Volume/Å^3	2748.223(17)
Z	4
ρ_{calc}g/cm^3	1.190
μ/mm^{-1}	0.546
F(000)	1032.0
Crystal size/mm^3	0.23 × 0.2 × 0.17
Radiation	Cu Kα (λ = 1.54184)
2Θ range for data collection/°	8.87 to 158.066
Index ranges	-18 ≤ h ≤ 18, -11 ≤ k ≤ 11, -25 ≤ l ≤ 25
Reflections collected	30726
Independent reflections	5708 [R_{int} = 0.0171, R_{sigma} = 0.0115]
Data/restraints/parameters	5708/75/391
Goodness-of-fit on F^2	1.087
Final R indexes [I>=2σ (I)]	R_1 = 0.0416, wR_2 = 0.1146
Final R indexes [all data]	R_1 = 0.0417, wR_2 = 0.1148
Largest diff. peak/hole / e Å^{-3}	0.37/-0.19
Flack parameter	0.10(8)

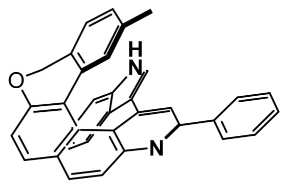

≡

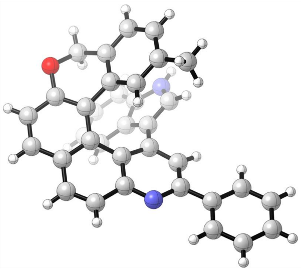

M-**5al**

Supplementary Fig. 2. The X-ray crystal structure of enantiopure *M*-**5al** (**CDCC 2212769**)

The crystal of enantiopure **5al** was obtained through slow vapor diffusion of n-hexane into a EA solution of **5al** at room temperature. The structure and absolute configuration (*M*) of **5al** were then determined by X-ray crystallography.

Supplementary Table 3. Crystal data and structure refinement for *M*-**5al**.

Identification code	p20220916c
Empirical formula	$C_{35}H_{24}N_2O$
Formula weight	488.56
Temperature/K	294.15
Crystal system	orthorhombic
Space group	$P2_12_12_1$
a/Å	9.17600(10)
b/Å	10.69990(10)
c/Å	26.6528(4)
α/°	90
β/°	90
γ/°	90
Volume/Å^3	2616.83(5)
Z	4
ρ_{calc}g/cm^3	1.240
μ/mm^{-1}	0.583
F(000)	1024.0
Crystal size/mm^3	0.24 × 0.22 × 0.15
Radiation	Cu Kα (λ = 1.54184)
2Θ range for data collection/°	8.906 to 158.088
Index ranges	-11 ≤ h ≤ 8, -13 ≤ k ≤ 13, -27 ≤ l ≤ 33
Reflections collected	26290
Independent reflections	5537 [R_{int} = 0.0307, R_{sigma} = 0.0216]
Data/restraints/parameters	5537/0/344
Goodness-of-fit on F^2	1.054
Final R indexes [I>=2σ (I)]	R_1 = 0.0443, wR_2 = 0.1225
Final R indexes [all data]	R_1 = 0.0474, wR_2 = 0.1249
Largest diff. peak/hole / e Å^{-3}	0.22/-0.15
Flack parameter	-0.12(10)

1.6. DFT calculations

Computational Methods.

Geometry optimizations and frequencies were performed with Gaussian 16 Rev. A.03[11] at the B3LYP[12-14]-D3(BJ)[15, 16] /def2-SVP[17] in gas phase at 298.15 K. Frequency calculations confirmed that optimized structures are minima (no imaginary frequency) or transition structures (one imaginary frequency). Single point energies were computed at the M06-2X[18, 19]-D3/def2-TZVPP level of theory in SMD[20](Toluene) solvent model.

1.6.1. DFT calculations on the mechanism and the enantioselectivity

We attempted to cultivate single crystals of **4a** to determine the relative and absolute stereochemistry. Unfortunately, we have tried many methods without success. At the same time, we also attempted to cultivate single crystals of other **4** (**4c**, **4d**, **4v**), and didn't get a satisfactory result either. But, according to reviews of enantioselective Povarov reactions reported by Benaglia et al.[21] and Menéndez et al.[22], and works reported by Ricci et al.[23], Shi et al.[24] and Corti et al.[25], and absolute configuration of *M*-**5a**, we can speculate that the absolute configuration of **4a** should be *M, S, R*. Additionally, the helical chiral racemization barriers for **4a** is 29.4 kcal/mol by DFT calculations.

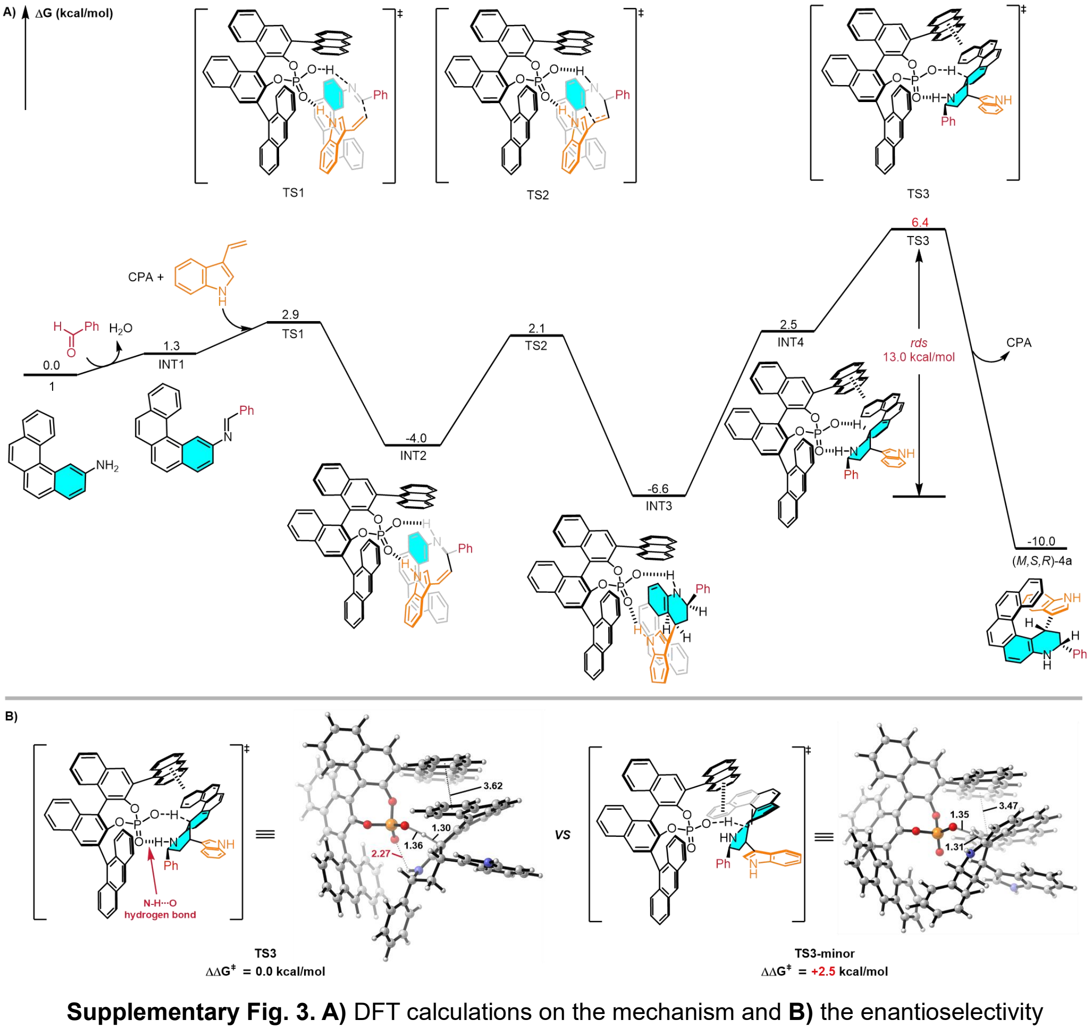

Supplementary Fig. 3. A) DFT calculations on the mechanism and **B)** the enantioselectivity

To gain insights into the mechanism and the enantioselectivity, we performed computational studies at the SMD(Toluene)/M06-2X-D3/def2-TZVPP//B3LYP D3(BJ)/def -2-SVP level of theory, employing the

CPA catalyst (*S*)-**A5**, as show in **Supplementary Fig. 3**. Firstly, **1a** and **2a** undergo dehydration condensation to obtain the **INT1** intermediate. Subsequently, with the assistance of CPA (*S*)-**A5**, **INT1** undergoes a stepwise [4+2] cyclization reaction with **3a** through the **TS1**, **INT2** and **TS3** to obtain **INT3** intermediate. The indole forms a strong N-H···O hydrogen bond with CPA (*S*)-**A5**, effectively promoting the occurrence of cyclization process. Then, **INT3** was isomerized into **INT4** to facilitate the proton migration process **TS3** which leads to the thermodynamically stable product (*M,S,R)*-**4a**. Among them, the proton migration process **TS3** is the rate-determining step of the reaction, so we further investigate **TS3** to understand the excellent enantioselective. The computed transition structure **TS3**, leading to the major enantiomer, is more stable than **TS3**-minor by 2.5 kcal/mol, agreeing well with the level and sense of the enantioselectivity experimentally observed (99% ee). Further analysis of the structure shows that there is a obvious N-H···O hydrogen bond in **TS3**, but this interaction cannot be observed in **TS3**-minor, which is the key factor in the origin of enantioselectivity.

1.6.2. DFT calculations on the enantiomerization process

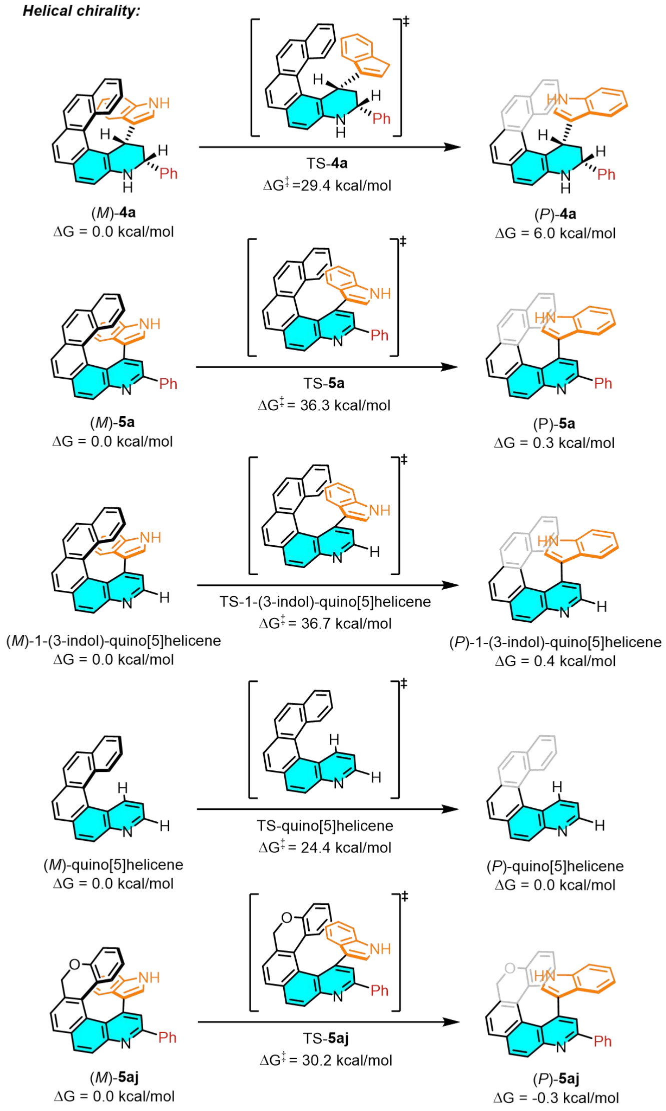

Supplementary Fig. 4. DFT calculations on the enantiomerization of helical chirality.

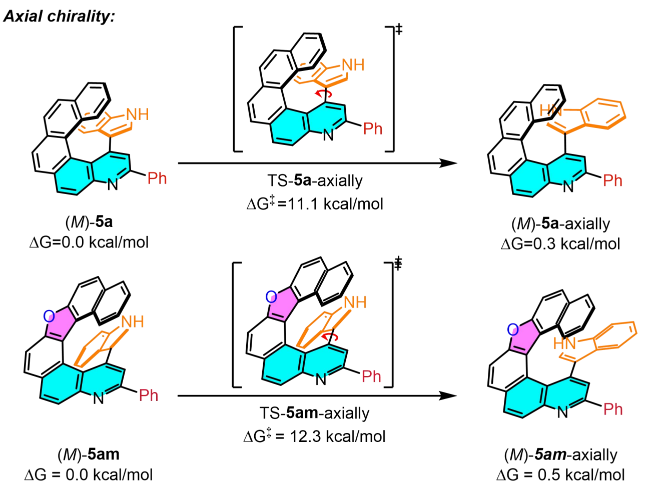

Supplementary Fig. 5. DFT calculations on the enantiomerization of axial chirality.

1.6.3. IRC Analysis of key transition states.

IRC-TS-1-(3-indol)-quino[5]helicene

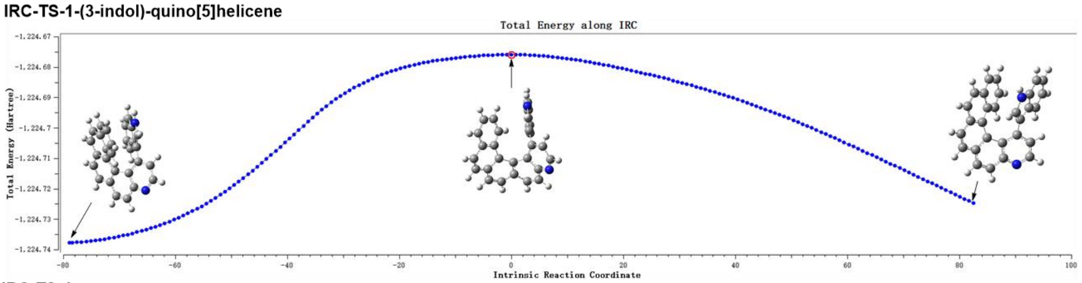

IRC-TS-4a

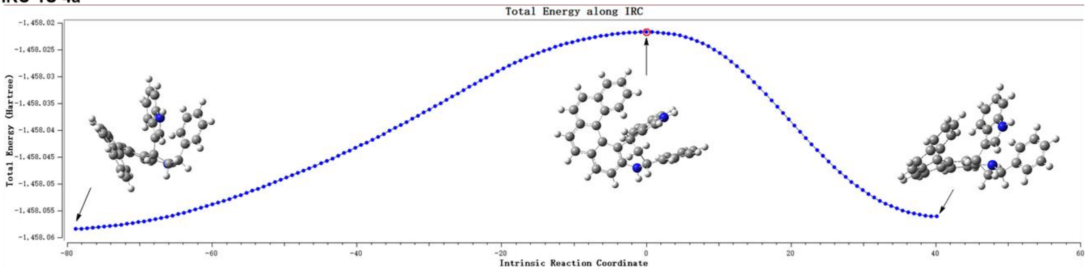

IRC-TS-5a

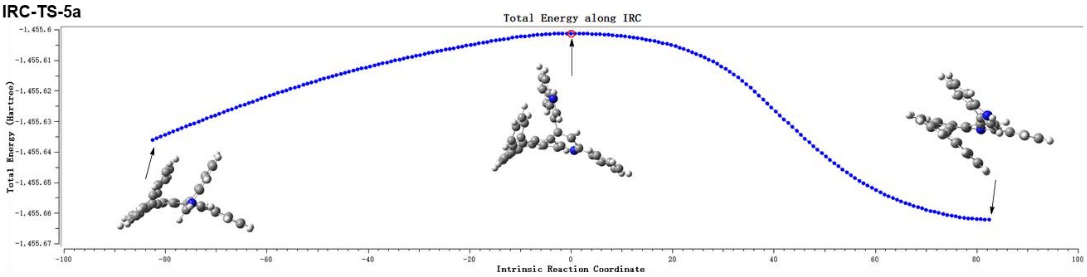

IRC-TS-5aj

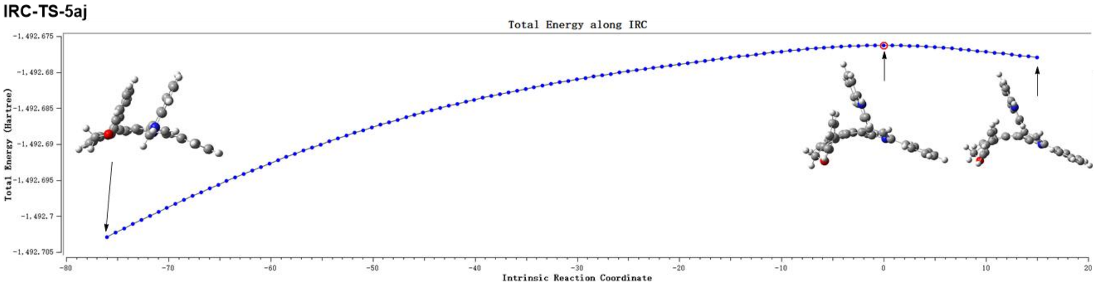

IRC-TS-Quino[5]helicene

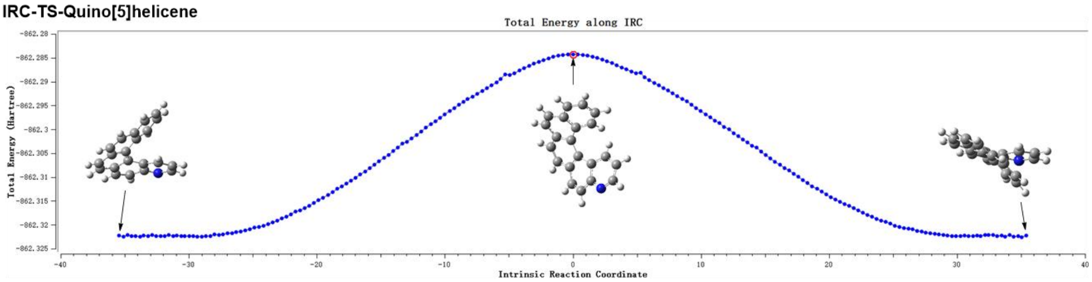

IRC-TS-5am-axially

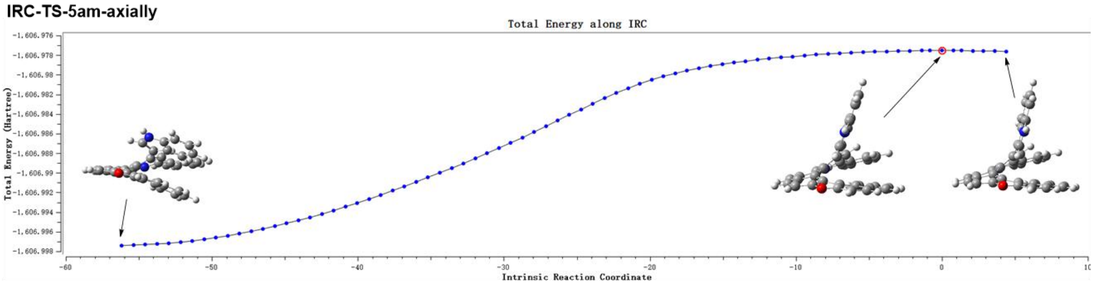

Supplementary Fig. 6. IRC Analysis of key transition states.

1.6.4. Cartesian coordinates and energies for all optimized compounds and transition states.

1a

C	1.53543	-1.13985	0.27272
C	2.8796	-1.50972	0.24128
C	3.85397	-0.53178	-0.0926
H	4.90835	-0.81575	-0.14428
C	3.46956	0.77274	-0.30195
H	4.22633	1.53639	-0.4998
C	2.11074	1.17149	-0.23875
C	1.10009	0.17689	-0.01772
C	-0.29748	0.58876	-0.01572
C	-1.42712	-0.32684	-0.08602
C	-1.33204	-1.67478	-0.53018
H	-0.38305	-2.04758	-0.90642
C	-2.43165	-2.51417	-0.56489
H	-2.31509	-3.53809	-0.92846
C	-3.70007	-2.05222	-0.15962
H	-4.56147	-2.72401	-0.17079
C	-3.84829	-0.72805	0.20185
H	-4.83387	-0.33218	0.4607
C	-2.74407	0.16078	0.21019
C	-2.94715	1.5566	0.44028
H	-3.9559	1.90967	0.66848
C	-1.91254	2.43447	0.30613
H	-2.07937	3.5098	0.4071
C	-0.57784	1.97956	0.05981
C	0.46467	2.94429	-0.10704
H	0.19886	4.00382	-0.08883
C	1.75553	2.55045	-0.31192
H	2.54547	3.2869	-0.48051
N	3.27202	-2.81499	0.49483
H	2.60219	-3.40091	0.97952
H	4.22325	-2.9461	0.81807
H	0.81211	-1.88883	0.5863

Zero-point correction= 0.258723 (Hartree/Particle)
Thermal correction to Energy= 0.272140
Thermal correction to Enthalpy= 0.273084
Thermal correction to Gibbs Free Energy= 0.219266
sp-E = -748.511482928 hartree

2

C	-0.36123	-1.29422	0.00044

C	0.53525	-0.21623	0.00054
C	0.04719	1.10116	0.0004
C	-1.32567	1.33287	-0.00012
C	-2.21803	0.2519	-0.00034
C	-1.73764	-1.06119	-0.00004
H	0.02704	-2.317	0.00057
H	0.76857	1.92097	0.00054
H	-1.70915	2.3559	-0.00031
H	-3.29517	0.43637	-0.00077
H	-2.43696	-1.90034	-0.00023
C	1.99654	-0.46683	-0.00048
H	2.27962	-1.55257	-0.00159
O	2.84346	0.39648	-0.00007

Zero-point correction= 0.110058 (Hartree/Particle)

Thermal correction to Energy= 0.116309

Thermal correction to Enthalpy= 0.117253

Thermal correction to Gibbs Free Energy= 0.079576

sp-E = -345.566323955 hartree

3

C	-0.46282	-1.51015	0.00055
C	0.02344	-0.18887	0.00015
C	-0.92483	0.87705	-0.00017
C	-2.30724	0.65878	-0.00039
C	-2.75202	-0.66004	-0.00021
C	-1.83697	-1.73265	0.00029
H	0.22128	-2.35817	0.00124
H	-3.01173	1.49401	-0.0007
H	-3.82483	-0.86641	-0.00041
H	-2.21531	-2.75747	0.0006
N	-0.21683	2.06213	-0.00007
H	-0.62663	2.98574	-0.00046
C	1.12955	1.78896	0.00022
C	1.34373	0.42017	0.00031
C	2.67037	-0.17713	0.00034
H	3.4877	0.55475	0.00129
C	3.0022	-1.47899	-0.00082
H	2.25962	-2.27783	-0.00217
H	4.05104	-1.78454	-0.00065
H	1.86426	2.59224	0.00021

Zero-point correction= 0.163084 (Hartree/Particle)

Thermal correction to Energy= 0.171735

Thermal correction to Enthalpy= 0.172679

Thermal correction to Gibbs Free Energy= 0.129105

sp-E = -441.207999092 hartree

4a

N	1.7712	1.68773	2.45299
H	2.37347	2.36754	2.89433
C	-0.43037	-0.51569	-1.07235
C	-1.55509	-1.23214	-1.53391
C	-1.39861	-2.49159	-2.17635
H	-2.28974	-3.01546	-2.53251
C	-0.16622	-3.09073	-2.23212
H	-0.06584	-4.11013	-2.61339
C	0.98771	-2.41713	-1.76534
C	0.87507	-1.05326	-1.33379
C	2.12564	-0.35251	-1.0866
C	2.2853	1.08083	-1.19827
C	1.38985	1.89423	-1.93857
H	0.57903	1.4134	-2.48517
C	1.54765	3.26702	-2.00484
H	0.84535	3.86651	-2.58939
C	2.62284	3.89444	-1.33902
H	2.73398	4.98067	-1.37961
C	3.56607	3.11893	-0.68982
H	4.44244	3.5844	-0.23022
C	3.44688	1.70851	-0.64216
C	4.48974	0.89325	-0.1039
H	5.3621	1.37848	0.34139
C	4.43404	-0.46541	-0.23081
H	5.27243	-1.08875	0.08964
C	3.27907	-1.11597	-0.77066
C	3.29905	-2.52035	-1.02814
H	4.20969	-3.08281	-0.81024
C	2.22055	-3.12027	-1.61487
H	2.25602	-4.17217	-1.91
C	0.30435	0.79143	0.989
C	0.88724	1.95763	1.42819
H	0.78261	2.96618	1.03398
C	1.78954	0.3301	2.69365
C	0.85358	-0.27139	1.79936
C	0.68978	-1.66942	1.83073
H	-0.01007	-2.15894	1.15265
C	1.44988	-2.41968	2.72165
H	1.33627	-3.506	2.74428
C	2.37408	-1.80425	3.59322
H	2.96018	-2.42149	4.2784
C	2.55269	-0.42348	3.59351

H	3.26994	0.05383	4.26567
C	-0.68253	0.68413	-0.14344
H	-0.60377	1.62185	-0.71592
C	-2.11249	0.65152	0.4292
H	-2.3037	1.58914	0.96921
H	-2.21269	-0.17033	1.15606
C	-3.1548	0.45978	-0.67423
N	-2.84407	-0.78666	-1.33429
H	-3.58175	-1.24376	-1.85328
C	-4.56655	0.44829	-0.12513
C	-5.36196	1.59925	-0.19401
C	-5.08123	-0.69223	0.50877
C	-6.64419	1.61533	0.36058
H	-4.97142	2.49165	-0.69116
C	-6.36384	-0.68026	1.06076
H	-4.46596	-1.59302	0.56526
C	-7.14936	0.47418	0.98931
H	-7.2531	2.52043	0.29617
H	-6.75188	-1.57632	1.55149
H	-8.15342	0.48327	1.42011
H	-3.07013	1.31589	-1.37631

Zero-point correction= 0.514658 (Hartree/Particle)
Thermal correction to Energy= 0.541628
Thermal correction to Enthalpy= 0.542572
Thermal correction to Gibbs Free Energy= 0.456951
sp-E = -1458.90126572 hartree

CPA

C	1.24951	2.82045	1.16873
H	4.45544	1.59074	1.53387
C	3.41529	1.63993	1.20439
C	0.72803	1.78624	0.31724
C	1.54627	0.70288	0.02748
C	2.89781	0.60528	0.45349
C	-2.86956	0.7212	-0.39507
C	-3.36151	1.81565	-1.07557
C	-1.50979	0.75029	0.01993
H	-4.40662	1.82244	-1.39306
O	1.04802	-0.34692	-0.72948
O	-1.03363	-0.36111	0.69354
P	-0.00971	-1.38766	-0.06281
O	0.46518	-2.44585	0.84068
C	0.45297	3.89813	1.64766
C	3.14476	3.77764	2.4223

C	2.35262	4.82212	2.84524
C	0.99007	4.87201	2.46276
H	-0.59928	3.94552	1.37017
H	4.1921	3.71474	2.7279
H	2.76725	5.60364	3.48597
H	0.35657	5.68667	2.82126
C	-0.8453	5.04071	-2.1627
C	-2.21196	5.05638	-2.53325
C	-3.03349	4.01349	-2.16583
H	-0.18962	5.85571	-2.47782
H	-2.6071	5.88802	-3.12103
H	-4.08506	4.00268	-2.46323
C	-0.66252	1.82432	-0.21096
C	-2.53246	2.9203	-1.4061
C	-1.1584	2.92366	-0.99292
C	-0.33229	4.00311	-1.41326
H	0.72299	4.00027	-1.14306
C	2.61823	2.74898	1.59287
C	3.70633	-0.59398	0.09209
C	3.84797	-1.64554	1.02332
C	4.30768	-0.6704	-1.18307
C	4.64099	-2.80175	0.66996
C	5.09283	-1.83285	-1.52934
C	5.24214	-2.86297	-0.59261
C	-3.70957	-0.47264	-0.09219
C	-4.15772	-0.69294	1.23032
C	-4.03216	-1.38635	-1.12216
C	-4.95678	-1.85933	1.52775
C	-4.8357	-2.54971	-0.81397
C	-5.27563	-2.75377	0.49895
H	5.83889	-3.74045	-0.85637
H	-5.87995	-3.63575	0.72745
C	-5.4016	-2.07219	2.87098
C	-5.08042	-1.18921	3.86935
C	-4.29576	-0.03536	3.578
C	-3.85181	0.20524	2.30239
H	-6.00399	-2.95902	3.0835
H	-5.42398	-1.36446	4.89146
H	-4.04687	0.66221	4.38108
H	-3.25471	1.09295	2.09222
C	-4.69929	-3.27448	-3.14045
C	-5.15293	-3.47225	-1.86189
C	-3.57312	-1.22478	-2.47099
C	-3.89431	-2.13707	-3.4445

H	-4.94703	-3.98779	-3.92994
H	-5.76518	-4.34373	-1.61639
H	-2.94253	-0.36992	-2.71218
H	-3.52639	-1.99509	-4.4633
C	5.69688	-1.89869	-2.82475
C	5.53671	-0.8836	-3.73295
C	4.7608	0.26336	-3.39399
C	4.16863	0.36679	-2.16037
H	6.2899	-2.78197	-3.07583
H	6.00187	-0.94849	-4.71943
H	4.63848	1.06534	-4.12591
H	3.57824	1.24925	-1.91029
C	4.78436	-3.85977	1.62252
C	4.1753	-3.79244	2.84879
C	3.37933	-2.66097	3.19228
C	3.22019	-1.62398	2.30998
H	5.38895	-4.72761	1.34668
H	4.28918	-4.60929	3.56541
H	2.88075	-2.62912	4.16362
H	2.59267	-0.77363	2.57624
O	-0.73329	-1.82826	-1.427
H	-1.26916	-2.62867	-1.31772

Zero-point correction= 0.634033 (Hartree/Particle)
Thermal correction to Energy= 0.672798
Thermal correction to Enthalpy= 0.673742
Thermal correction to Gibbs Free Energy= 0.561392
sp-E = -2489.01305740 hartree

H_2O

O	0.	0.	0.12028
H	0.	-0.75679	-0.4811
H	0.	0.75679	-0.4811

Zero-point correction= 0.021232 (Hartree/Particle)
Thermal correction to Energy= 0.024067
Thermal correction to Enthalpy= 0.025012
Thermal correction to Gibbs Free Energy= 0.003569
sp-E = -76.4331253958 hartree

INT1

N	-2.34589	-1.28896	-0.36139
C	-2.79189	-0.37323	0.41025
C	0.04418	-0.78977	-0.15775
C	-1.00824	-1.68957	-0.31543
C	-0.7265	-3.07127	-0.47109

H	-1.55878	-3.75754	-0.63575
C	0.56895	-3.51846	-0.34778
H	0.78674	-4.58859	-0.3929
C	1.64756	-2.61987	-0.13854
C	1.39896	-1.20677	-0.12769
C	2.52328	-0.29187	0.00836
C	2.45694	1.1406	-0.24036
C	1.42618	1.77107	-0.99084
H	0.66818	1.16322	-1.47792
C	1.3946	3.14098	-1.1828
H	0.59143	3.58176	-1.77813
C	2.40091	3.96458	-0.63904
H	2.36186	5.04736	-0.77833
C	3.46127	3.38162	0.02561
H	4.28379	3.99574	0.40139
C	3.53424	1.97739	0.20526
C	4.70929	1.38432	0.76253
H	5.51778	2.03801	1.09889
C	4.843	0.02802	0.80577
H	5.76993	-0.42878	1.16118
C	3.76949	-0.83556	0.41828
C	3.96787	-2.25161	0.45005
H	4.95604	-2.62904	0.72394
C	2.96101	-3.11255	0.12052
H	3.12965	-4.1921	0.10512
H	-0.19937	0.26109	-0.03498
H	-2.15505	0.08722	1.19149
C	-4.16958	0.1271	0.33662
C	-4.59343	1.12906	1.22535
C	-5.07749	-0.37888	-0.61252
C	-5.89925	1.61943	1.16942
H	-3.88998	1.52414	1.96362
C	-6.37943	0.11011	-0.6656
H	-4.73102	-1.15741	-1.29441
C	-6.794	1.11058	0.22429
H	-6.22008	2.39912	1.86413
H	-7.08064	-0.28668	-1.40375
H	-7.81675	1.49263	0.17889

Zero-point correction= 0.344875 (Hartree/Particle)

Thermal correction to Energy= 0.363534

Thermal correction to Enthalpy= 0.364478

Thermal correction to Gibbs Free Energy= 0.296629

sp-E = -1017.64399312 hartree

INT2

N	1.54183	1.18326	0.9388
C	1.69822	0.7135	2.2783
C	3.92347	1.29593	0.50421
C	2.5938	1.46788	0.10854
C	2.30858	1.85956	-1.23538
H	1.26955	2.02686	-1.51998
C	3.33347	1.92824	-2.14177
H	3.11557	2.16806	-3.18561
C	4.67987	1.63163	-1.78675
C	5.00041	1.36981	-0.41548
C	6.37593	1.05442	-0.06295
C	6.87627	0.96119	1.30094
C	6.23719	1.55482	2.42489
H	5.37169	2.19403	2.27066
C	6.72934	1.40778	3.71023
H	6.2149	1.89454	4.54248
C	7.90162	0.66218	3.94764
H	8.27715	0.53371	4.96537
C	8.59776	0.14469	2.87298
H	9.54393	-0.38032	3.02857
C	8.13202	0.31089	1.54455
C	8.94153	-0.10096	0.43982
H	9.89648	-0.59185	0.64301
C	8.55682	0.1801	-0.83783
H	9.20759	-0.06661	-1.6806
C	7.28113	0.76637	-1.12045
C	6.92024	1.02752	-2.47654
H	7.662	0.84228	-3.25698
C	5.67643	1.50056	-2.79117
H	5.40611	1.71426	-3.82806
H	4.12761	1.00286	1.52442
H	2.55989	1.22026	2.7365
C	0.47061	1.02603	3.11166
C	0.58499	1.80535	4.26922
C	-0.79619	0.56452	2.72558
C	-0.54742	2.13631	5.02047
H	1.56798	2.17163	4.57849
C	-1.92717	0.89585	3.47271
H	-0.92686	-0.02856	1.82163
C	-1.80786	1.68568	4.62026
H	-0.44275	2.75387	5.91582
H	-2.9077	0.5473	3.14683
H	-2.69882	1.9498	5.19457

C	4.81705	-1.90872	-1.33753
C	3.44824	-1.74329	-1.12476
C	2.58059	-1.5988	-2.23372
C	3.03449	-1.63402	-3.54793
C	4.40705	-1.81909	-3.74172
C	5.28562	-1.95202	-2.6541
H	5.51331	-1.98507	-0.50048
H	2.34518	-1.5199	-4.38562
H	4.80431	-1.84904	-4.75864
H	6.35413	-2.07491	-2.83768
N	1.28009	-1.40042	-1.74488
H	0.38245	-1.20717	-2.27115
C	1.27853	-1.39989	-0.42371
C	2.61569	-1.57754	0.07256
C	2.97771	-1.3511	1.37234
H	4.03097	-1.44973	1.64844
C	2.00862	-0.86494	2.37403
H	2.35115	-1.05622	3.39943
H	1.04148	-1.36806	2.24894
H	0.35257	-1.31662	0.13365
C	-5.41736	-2.1012	0.01908
H	-3.70074	-5.0785	-0.31046
C	-3.86379	-4.00364	-0.20272
C	-4.28941	-1.22655	0.16108
C	-3.01297	-1.76133	0.01747
C	-2.77732	-3.15665	-0.13392
C	-3.78418	2.54945	-0.09483
C	-4.46854	3.00144	1.01431
C	-3.7968	1.14936	-0.36242
H	-4.4729	4.07022	1.23921
O	-1.92135	-0.9363	0.06322
O	-3.11373	0.69066	-1.46693
P	-1.58815	0.11438	-1.22488
O	-1.18582	-0.69185	-2.42891
C	-6.75834	-1.62522	0.01273
C	-6.31198	-4.37773	-0.28548
C	-7.59892	-3.88589	-0.26781
C	-7.82001	-2.49386	-0.1269
H	-6.93881	-0.55485	0.11183
H	-6.12793	-5.44801	-0.40935
H	-8.44897	-4.56389	-0.37329
H	-8.84081	-2.10455	-0.13486
C	-6.18899	0.31247	3.79151
C	-6.25511	1.70731	4.02667

C	-5.72262	2.58205	3.10454
H	-6.59123	-0.3817	4.53314
H	-6.71683	2.08458	4.94206
H	-5.74709	3.66007	3.2828
C	-4.42587	0.22516	0.46143
C	-5.10835	2.10761	1.91281
C	-5.07341	0.69655	1.65418
C	-5.61593	-0.17908	2.63697
H	-5.56327	-1.25504	2.47696
C	-5.19427	-3.50798	-0.15527
C	-1.37529	-3.66608	-0.1753
C	-0.77137	-3.99032	-1.41005
C	-0.64909	-3.7947	1.03212
C	0.58469	-4.49463	-1.42997
C	0.69851	-4.31611	1.00355
C	1.2768	-4.65812	-0.22501
C	-2.96404	3.45216	-0.95052
C	-3.36186	3.72464	-2.27797
C	-1.74507	3.96119	-0.44395
C	-2.51168	4.5264	-3.12705
C	-0.89084	4.74216	-1.3097
C	-1.29802	5.00799	-2.6222
H	2.3028	-5.03389	-0.24649
H	-0.647	5.59857	-3.27281
C	-2.92788	4.79527	-4.46945
C	-4.11156	4.30229	-4.95541
C	-4.95211	3.51	-4.11981
C	-4.58909	3.23229	-2.82622
H	-2.27615	5.40377	-5.10196
H	-4.41616	4.51345	-5.98329
H	-5.89318	3.12174	-4.51663
H	-5.24125	2.62772	-2.19488
C	0.77436	4.91372	0.4652
C	0.37077	5.20139	-0.81261
C	-1.2815	3.68781	0.88326
C	-0.06824	4.14964	1.32371
H	1.74584	5.25905	0.8266
H	1.01201	5.77971	-1.48272
H	-1.88987	3.0716	1.54168
H	0.26196	3.90835	2.33534
C	1.42157	-4.43675	2.23171
C	0.86271	-4.04989	3.42345
C	-0.45779	-3.51449	3.45357
C	-1.19056	-3.39787	2.29809

H	2.43749	-4.83811	2.19675
H	1.42913	-4.14155	4.3532
H	-0.88798	-3.19186	4.40418
H	-2.20078	-2.98934	2.33263
C	1.20203	-4.77372	-2.68951
C	0.52816	-4.56171	-3.86477
C	-0.80526	-4.05723	-3.84803
C	-1.43507	-3.78195	-2.66126
H	2.23069	-5.14167	-2.6938
H	1.015	-4.76814	-4.82092
H	-1.32229	-3.87049	-4.79171
H	-2.43921	-3.36091	-2.65836
O	-0.68974	1.14758	-0.61454
H	0.59885	1.26552	0.52375

Zero-point correction= 1.151219 (Hartree/Particle)

Thermal correction to Energy= 1.217887

Thermal correction to Enthalpy= 1.218831

Thermal correction to Gibbs Free Energy= 1.047779

sp-E = -3947.93408609 hartree

INT3

N	1.31808	-1.8845	-0.68415
C	1.54924	-1.65056	-2.10958
C	3.65916	-1.52267	-0.41561
C	2.31561	-1.84294	0.16051
C	2.09603	-1.95114	1.56514
H	1.07883	-2.09752	1.92745
C	3.16679	-1.7809	2.37983
H	3.01955	-1.81463	3.46223
C	4.51849	-1.53785	1.91664
C	4.82581	-1.47186	0.54015
C	6.17722	-1.23437	0.11671
C	6.66678	-1.18724	-1.27465
C	5.9529	-1.63787	-2.41694
H	4.98765	-2.11301	-2.31589
C	6.44948	-1.53237	-3.7062
H	5.84849	-1.90309	-4.53974
C	7.71152	-0.96396	-3.94405
H	8.09579	-0.86623	-4.96152
C	8.4662	-0.55884	-2.86162
H	9.46786	-0.14787	-3.00921
C	7.98521	-0.68047	-1.53532
C	8.85807	-0.33309	-0.45577
H	9.85621	0.04305	-0.69113

C	8.46299	-0.50882	0.83077
H	9.13888	-0.28359	1.65849
C	7.13793	-0.94845	1.14829
C	6.79156	-1.0268	2.51657
H	7.55871	-0.81012	3.26252
C	5.51469	-1.3401	2.89822
H	5.24119	-1.39457	3.95366
H	3.83924	-2.28538	-1.18255
H	2.16534	-2.49365	-2.46687
C	0.23607	-1.68161	-2.85677
C	-0.07355	-2.7381	-3.72261
C	-0.7217	-0.68769	-2.62827
C	-1.3312	-2.81418	-4.32883
H	0.66939	-3.51827	-3.91193
C	-1.97575	-0.76093	-3.23196
H	-0.51748	0.14141	-1.95635
C	-2.29039	-1.8286	-4.07663
H	-1.56473	-3.65098	-4.99128
H	-2.71611	0.01112	-3.02622
H	-3.28386	-1.89197	-4.52548
C	5.08921	1.82843	1.0331
C	3.71581	1.56388	0.87474
C	2.79823	2.07128	1.85211
C	3.23245	2.78663	2.9756
C	4.59977	3.01176	3.11271
C	5.51801	2.54503	2.14561
H	5.81176	1.47773	0.29474
H	2.51486	3.16172	3.70657
H	4.96827	3.56739	3.97858
H	6.58346	2.75101	2.27462
N	1.53009	1.72556	1.45048
H	0.64416	1.79436	1.96616
C	1.60746	1.02381	0.28149
C	2.9232	0.86341	-0.11042
C	3.3969	-0.11669	-1.1512
H	4.35634	0.20816	-1.57104
C	2.3857	-0.35074	-2.29383
H	2.90797	-0.43494	-3.25816
H	1.71487	0.51221	-2.36998
H	0.70365	0.65875	-0.18456
C	-5.18309	2.32898	-0.49488
H	-3.27228	5.20092	-0.41364
C	-3.50612	4.13386	-0.4206
C	-4.11534	1.37083	-0.48012

C	-2.80658	1.8316	-0.35075
C	-2.47739	3.21759	-0.33013
C	-3.9085	-2.35001	0.34276
C	-4.6164	-2.91329	-0.69851
C	-3.80822	-0.92814	0.39707
H	-4.71134	-3.99999	-0.75491
O	-1.78087	0.93175	-0.26365
O	-3.11289	-0.3667	1.43843
P	-1.53333	0.05999	1.15959
O	-1.07371	0.97551	2.24505
C	-6.55543	1.95178	-0.48121
C	-5.91937	4.68184	-0.51726
C	-7.23771	4.28188	-0.52921
C	-7.55486	2.90168	-0.4988
H	-6.81231	0.89325	-0.44756
H	-5.66067	5.74384	-0.52004
H	-8.03878	5.02446	-0.54819
H	-8.60104	2.58687	-0.48269
C	-6.10318	-0.55628	-3.88089
C	-6.28846	-1.96038	-3.89794
C	-5.82926	-2.72187	-2.84531
H	-6.44536	0.04598	-4.72583
H	-6.78222	-2.43528	-4.74893
H	-5.94395	-3.80883	-2.85447
C	-4.35318	-0.09608	-0.57372
C	-5.17595	-2.12035	-1.73445
C	-5.02683	-0.6943	-1.69283
C	-5.49243	0.05882	-2.80765
H	-5.35086	1.13877	-2.81433
C	-4.86473	3.72787	-0.486
C	-1.05885	3.6598	-0.20001
C	-0.62222	4.26245	1.00333
C	-0.1605	3.49341	-1.27815
C	0.72784	4.77044	1.09762
C	1.20311	3.95609	-1.15217
C	1.60513	4.60237	0.02079
C	-3.19123	-3.17387	1.35493
C	-3.61614	-3.16581	2.70282
C	-2.04503	-3.90769	0.96778
C	-2.86687	-3.90208	3.694
C	-1.29734	-4.6335	1.97022
C	-1.72586	-4.61166	3.30231
H	2.636	4.9524	0.1127
H	-1.15411	-5.1604	4.05586

C	-3.30748	-3.88162	5.05526
C	-4.42371	-3.17712	5.42565
C	-5.16814	-2.45301	4.44909
C	-4.77771	-2.44899	3.13416
H	-2.73041	-4.44177	5.79564
H	-4.74836	-3.16731	6.46889
H	-6.05667	-1.89552	4.7545
H	-5.35596	-1.89248	2.39637
C	0.30496	-5.35692	0.27775
C	-0.11844	-5.34718	1.58134
C	-1.55407	-3.93136	-0.37867
C	-0.42724	-4.63907	-0.71203
H	1.20382	-5.90997	-0.00629
H	0.43669	-5.88805	2.35231
H	-2.07257	-3.35544	-1.14396
H	-0.08116	-4.6378	-1.74662
C	2.11424	3.73644	-2.23324
C	1.7096	3.11292	-3.38525
C	0.35834	2.67815	-3.52391
C	-0.54446	2.86809	-2.50789
H	3.14698	4.07213	-2.11235
H	2.41738	2.94655	-4.20088
H	0.04132	2.18273	-4.44446
H	-1.57475	2.53293	-2.62772
C	1.15293	5.39198	2.31322
C	0.31042	5.48117	3.39197
C	-1.00656	4.94097	3.31699
C	-1.45806	4.35271	2.16251
H	2.17484	5.77375	2.36802
H	0.64975	5.95097	4.31842
H	-1.65668	4.98377	4.19384
H	-2.4544	3.91456	2.12607
O	-0.76219	-1.15858	0.71156
H	0.3499	-1.78647	-0.26963

Zero-point correction= 1.153457 (Hartree/Particle)
Thermal correction to Energy= 1.219339
Thermal correction to Enthalpy= 1.220284
Thermal correction to Gibbs Free Energy= 1.051504
sp-E = -3947.94199730 hartree

INT4

N	1.75247	2.54124	0.03828
C	1.80414	2.36925	-1.41414
C	2.4814	0.27374	0.16651

C	2.05021	1.53452	0.83254
C	2.04858	1.67397	2.24902
H	1.67928	2.59639	2.68973
C	2.52882	0.65345	3.00115
H	2.57245	0.76839	4.08744
C	3.00334	-0.59606	2.45446
C	2.88504	-0.86253	1.07618
C	3.33358	-2.12199	0.5679
C	3.11715	-2.60192	-0.79724
C	2.06327	-2.15515	-1.63004
H	1.27035	-1.50833	-1.26238
C	1.95698	-2.57259	-2.94605
H	1.11465	-2.21553	-3.54102
C	2.8895	-3.47259	-3.49162
H	2.8034	-3.79022	-4.53236
C	3.86503	-4.01137	-2.67415
H	4.54843	-4.77527	-3.05434
C	3.97117	-3.62584	-1.31631
C	4.86914	-4.31412	-0.43672
H	5.49362	-5.11182	-0.8473
C	4.88554	-4.03283	0.89601
H	5.51064	-4.61072	1.58115
C	4.13262	-2.9375	1.43206
C	4.23317	-2.6398	2.81178
H	4.80226	-3.3146	3.45525
C	3.62741	-1.52259	3.3262
H	3.6769	-1.29765	4.3939
H	1.59663	-0.00125	-0.42985
H	1.0428	1.61451	-1.67585
C	1.51181	3.64829	-2.15489
C	0.58463	3.63507	-3.2032
C	2.21527	4.82999	-1.8785
C	0.38164	4.77587	-3.98264
H	0.0157	2.72339	-3.40069
C	1.99691	5.97755	-2.64252
H	2.9478	4.85304	-1.06739
C	1.08594	5.94925	-3.70404
H	-0.34361	4.7495	-4.79784
H	2.54834	6.89367	-2.41756
H	0.92393	6.84407	-4.30993
C	5.03714	3.04545	0.99672
C	5.49739	1.78272	0.57
C	6.78921	1.35343	1.00621
C	7.60038	2.13584	1.83726

C	7.1112	3.37461	2.2414
C	5.84156	3.82435	1.82228
H	4.0595	3.41361	0.68973
H	8.58414	1.78498	2.1583
H	7.72005	4.00778	2.89088
H	5.4848	4.80253	2.15331
N	7.01298	0.10673	0.46425
H	7.84552	-0.44913	0.60045
C	5.92103	-0.26772	-0.28878
C	4.96314	0.72299	-0.26055
C	3.61171	0.60267	-0.9005
H	3.64009	-0.27641	-1.54988
C	3.20097	1.81584	-1.76674
H	3.18876	1.53627	-2.82914
H	3.93471	2.6261	-1.65772
H	5.8868	-1.23491	-0.78609
C	-4.99719	0.19643	2.05783
H	-4.59767	3.61918	2.27066
C	-4.45841	2.5988	1.90572
C	-4.13677	-0.04244	0.93153
C	-3.42132	1.02484	0.40714
C	-3.5713	2.3608	0.87731
C	-2.48065	-3.29408	-0.21436
C	-3.58928	-4.04043	-0.57101
C	-2.68584	-1.95048	0.22537
H	-3.45138	-5.07634	-0.88782
O	-2.51302	0.79363	-0.59139
O	-1.59098	-1.24341	0.61511
P	-1.04501	0.16929	-0.09414
O	-0.47305	0.98521	1.02486
C	-5.65858	-0.85035	2.75965
C	-6.03361	1.78562	3.63383
C	-6.67986	0.75047	4.27354
C	-6.47653	-0.58112	3.83693
H	-5.50523	-1.88043	2.44035
H	-6.16463	2.8166	3.97291
H	-7.33646	0.9515	5.12332
H	-6.96859	-1.40406	4.36091
C	-7.46405	-2.35554	-0.66663
C	-7.27496	-3.70315	-1.06073
C	-6.01425	-4.25633	-1.01727
H	-8.45966	-1.90911	-0.72557
H	-8.12533	-4.29366	-1.40972
H	-5.84752	-5.28835	-1.33684

C	-3.96202	-1.39719	0.34099
C	-4.89697	-3.49642	-0.57183
C	-5.09115	-2.14623	-0.13324
C	-6.40318	-1.59901	-0.21527
H	-6.5641	-0.56173	0.07633
C	-5.17096	1.54041	2.53
C	-2.77317	3.45261	0.2523
C	-1.68529	4.02462	0.9499
C	-3.1089	3.90975	-1.04272
C	-0.91218	5.07492	0.3255
C	-2.34906	4.98075	-1.6431
C	-1.26559	5.52939	-0.94889
C	-1.13145	-3.92596	-0.28486
C	-0.68565	-4.4487	-1.52565
C	-0.35289	-4.11953	0.88387
C	0.50215	-5.27082	-1.57365
C	0.87341	-4.88207	0.80597
C	1.25486	-5.45739	-0.4113
H	-0.67621	6.32024	-1.41629
H	2.16375	-6.06187	-0.45332
C	0.88278	-5.87426	-2.8136
C	0.17278	-5.64359	-3.96241
C	-0.94948	-4.76549	-3.93801
C	-1.36463	-4.19159	-2.76211
H	1.76926	-6.51262	-2.82388
H	0.47704	-6.1087	-4.90327
H	-1.48271	-4.54587	-4.86617
H	-2.21738	-3.51367	-2.76046
C	1.26573	-4.53449	3.1874
C	1.659	-5.06464	1.98687
C	-0.74165	-3.61726	2.16767
C	0.04437	-3.80725	3.27618
H	1.8815	-4.67088	4.0793
H	2.58716	-5.63385	1.90333
H	-1.67295	-3.06296	2.26054
H	-0.27124	-3.39853	4.23899
C	-2.7211	5.45272	-2.94152
C	-3.76922	4.89102	-3.62379
C	-4.51261	3.82354	-3.03983
C	-4.19646	3.35373	-1.79039
H	-2.1456	6.27203	-3.37692
H	-4.04216	5.2595	-4.6157
H	-5.34361	3.38038	-3.59351
H	-4.7758	2.54227	-1.35008

C	0.20535	5.62924	1.03092
C	0.51974	5.20662	2.29907
C	-0.25448	4.18285	2.92339
C	-1.30236	3.59461	2.26146
H	0.79003	6.41046	0.53973
H	1.36273	5.65126	2.83427
H	0.00595	3.84882	3.9305
H	-1.84543	2.77008	2.71875
O	-0.26092	-0.13026	-1.34514
H	1.33007	3.3822	0.43243

Zero-point correction= 1.153656 (Hartree/Particle)

Thermal correction to Energy= 1.219977

Thermal correction to Enthalpy= 1.220921

Thermal correction to Gibbs Free Energy= 1.051013

sp-E = -3947.92698482 hartree

TS1-b

N	-2.14253	0.59346	0.67158
C	-2.51367	1.21678	1.76231
C	-4.35893	0.49892	-0.33077
C	-2.96983	0.20497	-0.37035
C	-2.4049	-0.4405	-1.49545
H	-1.32847	-0.59331	-1.54428
C	-3.22293	-0.85011	-2.51808
H	-2.79036	-1.32236	-3.40175
C	-4.64455	-0.74136	-2.42445
C	-5.24059	-0.19304	-1.25138
C	-6.67649	-0.17697	-1.11873
C	-7.39158	0.13136	0.11516
C	-6.81541	0.03864	1.41192
H	-5.81674	-0.373	1.53051
C	-7.52275	0.374	2.55375
H	-7.04564	0.27312	3.53167
C	-8.85925	0.81087	2.46321
H	-9.40973	1.09207	3.36378
C	-9.48179	0.82067	1.23015
H	-10.53852	1.0874	1.14741
C	-8.7885	0.44652	0.05172
C	-9.49462	0.29686	-1.18418
H	-10.56023	0.53641	-1.21141
C	-8.8632	-0.20999	-2.28142
H	-9.41796	-0.40096	-3.20334
C	-7.45476	-0.46574	-2.27645
C	-6.82811	-0.98248	-3.44806

H	-7.45775	-1.23634	-4.30433
C	-5.47326	-1.16981	-3.5007
H	-5.00143	-1.59922	-4.38765
H	-4.7685	0.76378	0.63115
H	-3.57455	1.43568	1.87815
C	-1.64945	1.62557	2.84288
C	-2.25391	2.30327	3.92565
C	-0.25683	1.39086	2.8724
C	-1.49134	2.75071	4.99863
H	-3.33181	2.48576	3.90802
C	0.4961	1.84108	3.95182
H	0.23377	0.87566	2.04637
C	-0.10955	2.52324	5.01275
H	-1.96981	3.28002	5.82575
H	1.57458	1.66951	3.95416
H	0.49349	2.87819	5.85192
C	-0.98146	4.48386	1.30786
C	-1.08062	3.76176	0.10849
C	0.10113	3.23603	-0.47444
C	1.35183	3.32642	0.13636
C	1.42083	4.03939	1.33281
C	0.27252	4.62944	1.89926
H	-1.86937	4.90463	1.78539
H	2.22173	2.84274	-0.2982
H	2.38308	4.13505	1.83858
H	0.36337	5.17742	2.83948
N	-0.25301	2.55337	-1.63124
H	0.38035	1.8655	-2.08436
C	-1.59774	2.55123	-1.75418
C	-2.19035	3.27955	-0.70306
C	-3.56383	3.25352	-0.34537
H	-3.84868	3.79375	0.56326
C	-4.51472	2.45111	-0.97006
H	-5.56639	2.57597	-0.70252
H	-4.34255	2.15377	-2.00668
H	-2.07671	2.01931	-2.57054
C	5.62864	-2.08693	-0.53834
H	6.94232	0.87906	-1.7106
C	6.22034	0.18938	-1.26735
C	4.36781	-1.58361	-0.07088
C	4.05207	-0.24823	-0.31131
C	4.97719	0.66115	-0.89936
C	1.08226	-3.32727	0.80041
C	1.44635	-4.06384	1.91161

C	2.08947	-2.54192	0.16802
H	0.69809	-4.68853	2.40441
O	2.82549	0.24305	0.06135
O	1.74899	-1.81873	-0.95019
P	1.43761	-0.20817	-0.74089
O	1.33184	0.41144	-2.10051
C	5.97566	-3.46635	-0.4779
C	7.82958	-1.66887	-1.56927
C	8.14293	-3.00681	-1.47282
C	7.1982	-3.91336	-0.93229
H	5.25534	-4.17638	-0.07278
H	8.54048	-0.9609	-2.00297
H	9.11048	-3.3731	-1.82371
H	7.43816	-4.978	-0.87997
C	5.28602	-3.70955	3.64913
C	4.3275	-4.5757	4.22981
C	3.0846	-4.70658	3.65068
H	6.26215	-3.58608	4.12422
H	4.57144	-5.1252	5.14196
H	2.32665	-5.35325	4.10021
C	3.39196	-2.45235	0.64333
C	2.75007	-3.9927	2.46629
C	3.73481	-3.15089	1.85047
C	4.99775	-3.01591	2.49265
H	5.74264	-2.34749	2.06293
C	6.57146	-1.17799	-1.12273
C	4.61014	2.09145	-1.12281
C	4.06631	2.48551	-2.36603
C	4.82934	3.04367	-0.10095
C	3.74135	3.87645	-2.59227
C	4.52238	4.43493	-0.34565
C	3.98764	4.81317	-1.5822
C	-0.3312	-3.32838	0.32413
C	-0.66915	-3.87564	-0.93728
C	-1.34586	-2.81345	1.1692
C	-2.0545	-3.93188	-1.34487
C	-2.73027	-2.90632	0.76188
C	-3.04875	-3.46623	-0.47819
H	3.73978	5.86338	-1.75658
H	-4.09485	-3.51112	-0.7905
C	-2.38195	-4.45598	-2.63486
C	-1.40418	-4.91684	-3.47848
C	-0.03939	-4.89266	-3.06791
C	0.31405	-4.39552	-1.83895

H	-3.43359	-4.47631	-2.93219
H	-1.66534	-5.31106	-4.46351
H	0.73198	-5.2727	-3.74169
H	1.36179	-4.38378	-1.5411
C	-3.43284	-1.80685	2.82508
C	-3.74998	-2.3943	1.62695
C	-1.06257	-2.16481	2.41696
C	-2.06721	-1.68585	3.21835
H	-4.22122	-1.4254	3.47916
H	-4.79006	-2.48546	1.30486
H	-0.02392	-2.03123	2.71453
H	-1.81866	-1.18254	4.15462
C	4.743	5.38811	0.69762
C	5.22576	5.00021	1.92123
C	5.51795	3.62754	2.17074
C	5.32737	2.68243	1.19217
H	4.50394	6.43593	0.49969
H	5.38267	5.73849	2.71119
H	5.89792	3.32668	3.15
H	5.55297	1.63385	1.39078
C	3.16758	4.25869	-3.84596
C	2.93163	3.32825	-4.82502
C	3.25387	1.95735	-4.60472
C	3.80011	1.54925	-3.41559
H	2.92029	5.31151	-4.00369
H	2.4908	3.6326	-5.77747
H	3.04438	1.22256	-5.38512
H	4.01254	0.49482	-3.24612
O	0.33989	-0.05435	0.28644
H	-1.11616	0.32932	0.52704

Zero-point correction= 1.148522 (Hartree/Particle)

Thermal correction to Energy= 1.214278

Thermal correction to Enthalpy= 1.215223

Thermal correction to Gibbs Free Energy= 1.048029

sp-E = -3947.89007327 hartree

TS1

N	1.55257	0.93881	1.04988
C	1.6576	0.38563	2.28513
C	3.94998	1.07116	0.64645
C	2.63517	1.30431	0.25558
C	2.36001	1.86643	-1.02155
H	1.32402	2.06082	-1.29761
C	3.39902	2.07147	-1.89356

H	3.19388	2.44495	-2.89955
C	4.74102	1.75278	-1.55324
C	5.04202	1.29947	-0.2284
C	6.41535	0.96229	0.10901
C	6.89019	0.69787	1.45861
C	6.21367	1.12035	2.63674
H	5.33386	1.75306	2.5496
C	6.68586	0.81527	3.90133
H	6.14397	1.17426	4.77978
C	7.87369	0.0736	4.06265
H	8.23372	-0.18014	5.06226
C	8.60372	-0.27869	2.94469
H	9.56048	-0.79676	3.05016
C	8.15855	0.05221	1.64011
C	8.99858	-0.18819	0.5082
H	9.96185	-0.67983	0.66461
C	8.6309	0.2526	-0.72896
H	9.30346	0.13674	-1.58253
C	7.34643	0.84242	-0.95698
C	7.00523	1.28881	-2.27046
H	7.76589	1.22826	-3.0524
C	5.76188	1.78421	-2.54393
H	5.5089	2.13883	-3.54562
H	4.13905	0.61046	1.60662
H	2.5771	0.64278	2.81623
C	0.46034	0.31731	3.15668
C	0.64019	0.25819	4.5494
C	-0.84304	0.26304	2.63685
C	-0.45847	0.1609	5.40419
H	1.6514	0.29256	4.96477
C	-1.94002	0.16639	3.49253
H	-1.0152	0.30019	1.56501
C	-1.75351	0.11558	4.87713
H	-0.30331	0.12461	6.48506
H	-2.94564	0.13533	3.07049
H	-2.61713	0.04354	5.54225
C	4.75326	-1.74317	-1.74576
C	3.37943	-1.63804	-1.50164
C	2.50015	-1.34134	-2.57525
C	2.95354	-1.15806	-3.88108
C	4.32685	-1.27757	-4.10314
C	5.21511	-1.56622	-3.05005
H	5.45555	-1.94432	-0.93438
H	2.25893	-0.93017	-4.69129

H	4.71986	-1.13939	-5.11299
H	6.28467	-1.63881	-3.25466
N	1.2079	-1.26392	-2.0593
H	0.32286	-0.98051	-2.54049
C	1.22376	-1.48717	-0.74217
C	2.55266	-1.71476	-0.30219
C	2.95922	-1.77512	1.03311
H	4.03349	-1.85631	1.2251
C	2.08509	-1.60563	2.12189
H	2.46766	-1.8158	3.12349
H	1.04298	-1.89432	1.98092
H	0.30204	-1.49282	-0.16883
C	-5.57681	-1.73771	-0.35344
H	-4.05213	-4.78071	-0.9364
C	-4.14616	-3.71183	-0.7312
C	-4.39859	-0.96426	-0.08664
C	-3.15784	-1.56791	-0.26466
C	-3.00884	-2.955	-0.54238
C	-3.61078	2.76368	0.19006
C	-4.30647	3.11465	1.32839
C	-3.72322	1.42025	-0.27534
H	-4.23917	4.13939	1.70042
O	-2.01437	-0.83155	-0.11842
O	-3.05157	1.07221	-1.42397
P	-1.58943	0.32312	-1.27391
O	-1.26313	-0.33722	-2.57972
C	-6.88191	-1.17134	-0.34171
C	-6.60939	-3.899	-0.9326
C	-7.86068	-3.32297	-0.8961
C	-7.99369	-1.94288	-0.60653
H	-6.99357	-0.10812	-0.12845
H	-6.493	-4.95981	-1.16893
H	-8.74965	-3.92426	-1.10004
H	-8.98547	-1.4846	-0.59876
C	-6.31941	0.21606	3.66152
C	-6.30664	1.5698	4.07651
C	-5.67918	2.51731	3.29751
H	-6.79636	-0.53924	4.29068
H	-6.78142	1.85625	5.01775
H	-5.64014	3.56178	3.61703
C	-4.44437	0.44271	0.39833
C	-5.04695	2.16009	2.07461
C	-5.09652	0.79657	1.62948
C	-5.73241	-0.15993	2.471

H	-5.74424	-1.20672	2.17128
C	-5.44134	-3.12955	-0.67581
C	-1.64373	-3.55862	-0.55268
C	-0.97774	-3.80858	-1.77196
C	-1.01592	-3.85596	0.68064
C	0.33189	-4.42352	-1.75617
C	0.27872	-4.49894	0.68537
C	0.91328	-4.77282	-0.53246
C	-2.66568	3.69323	-0.49039
C	-2.95066	4.17958	-1.78487
C	-1.43514	3.9981	0.13846
C	-1.97249	4.98768	-2.47533
C	-0.45329	4.78622	-0.571
C	-0.74877	5.26282	-1.85342
H	1.90169	-5.23911	-0.52622
H	-0.00141	5.85699	-2.38652
C	-2.27573	5.47303	-3.78683
C	-3.47123	5.17908	-4.39056
C	-4.43761	4.38027	-3.7118
C	-4.18499	3.89816	-2.45257
H	-1.52808	6.0842	-4.29916
H	-3.68908	5.55417	-5.39341
H	-5.3865	4.15116	-4.20253
H	-4.93153	3.28962	-1.94091
C	1.11008	4.53986	1.28688
C	0.81602	5.03434	0.04286
C	-1.08354	3.50558	1.43639
C	0.14204	3.76763	1.992
H	2.08855	4.72549	1.73594
H	1.55392	5.61926	-0.5119
H	-1.79617	2.8878	1.97863
H	0.38396	3.36181	2.97683
C	0.89311	-4.81117	1.93929
C	0.29129	-4.47709	3.12586
C	-0.96459	-3.80422	3.12478
C	-1.60158	-3.51519	1.94298
H	1.86623	-5.30837	1.92843
H	0.77857	-4.70972	4.07562
H	-1.42188	-3.51155	4.07247
H	-2.56449	-3.00457	1.95622
C	1.01997	-4.6209	-2.99447
C	0.45895	-4.22619	-4.18153
C	-0.82793	-3.61249	-4.19839
C	-1.5256	-3.41389	-3.03418

H	2.01348	-5.07484	-2.97014
H	1.00207	-4.36782	-5.11884
H	-1.25395	-3.28046	-5.14773
H	-2.48962	-2.90777	-3.05236
O	-0.60476	1.18796	-0.53431
H	0.62567	1.04178	0.58224

Zero-point correction= 1.149109 (Hartree/Particle)

Thermal correction to Energy= 1.215781

Thermal correction to Enthalpy= 1.216725

Thermal correction to Gibbs Free Energy= 1.045115

sp-E = -3947.92044871 hartree

TS2

N	1.44195	-1.83564	-0.51606
C	1.67958	-1.67841	-1.93334
C	3.78046	-1.50135	-0.19804
C	2.46021	-1.73331	0.33252
C	2.21441	-1.62016	1.73489
H	1.19312	-1.74178	2.09571
C	3.24317	-1.23827	2.54135
H	3.05366	-1.06869	3.60433
C	4.5824	-1.0095	2.0644
C	4.90051	-1.25571	0.70384
C	6.25175	-1.06408	0.24915
C	6.74936	-1.43341	-1.07513
C	6.15589	-2.42249	-1.90327
H	5.34982	-3.03947	-1.51397
C	6.63419	-2.70161	-3.173
H	6.15357	-3.47988	-3.77047
C	7.75125	-2.01226	-3.68204
H	8.11511	-2.21963	-4.69073
C	8.4162	-1.1164	-2.86673
H	9.32858	-0.62525	-3.21438
C	7.96388	-0.84226	-1.5527
C	8.76204	-0.04597	-0.66862
H	9.68953	0.38816	-1.04946
C	8.40744	0.10092	0.63799
H	9.0527	0.64442	1.33219
C	7.1569	-0.39753	1.13057
C	6.80066	-0.15579	2.48292
H	7.5286	0.32842	3.13716
C	5.56044	-0.50236	2.95315
H	5.28685	-0.3219	3.99463
H	3.99451	-1.98577	-1.14045

H	2.40283	-2.45152	-2.23942
C	0.41102	-1.89375	-2.72688
C	0.32839	-2.92973	-3.66559
C	-0.7134	-1.08948	-2.50235
C	-0.86414	-3.17365	-4.35411
H	1.20031	-3.56465	-3.84769
C	-1.90427	-1.33246	-3.1862
H	-0.69512	-0.28527	-1.77073
C	-1.98723	-2.37848	-4.10964
H	-0.9166	-3.99288	-5.07516
H	-2.77644	-0.71198	-2.9826
H	-2.92919	-2.57262	-4.62751
C	4.90092	2.23515	1.00802
C	3.55143	1.89978	0.83074
C	2.58783	2.37079	1.77339
C	2.94939	3.13265	2.88747
C	4.30101	3.4396	3.04682
C	5.26331	3.00272	2.11358
H	5.65897	1.89263	0.30155
H	2.19469	3.48488	3.59165
H	4.61775	4.03608	3.90567
H	6.31301	3.26602	2.2633
N	1.34301	1.94175	1.35112
H	0.43437	1.97961	1.84652
C	1.47063	1.23715	0.2059
C	2.81464	1.13792	-0.16372
C	3.3156	0.21914	-1.14857
H	4.34176	0.39227	-1.48548
C	2.35795	-0.28161	-2.21313
H	2.88031	-0.37022	-3.17604
H	1.55668	0.45338	-2.34925
H	0.59749	0.82799	-0.28282
C	-5.29964	2.12035	-0.63715
H	-3.49596	5.05474	-0.84427
C	-3.69028	3.98397	-0.74924
C	-4.19697	1.20724	-0.55296
C	-2.9058	1.72407	-0.48432
C	-2.62749	3.1159	-0.59664
C	-3.81898	-2.42437	0.55643
C	-4.5009	-3.09666	-0.437
C	-3.78938	-0.99953	0.50448
H	-4.53892	-4.18799	-0.41402
O	-1.84767	0.87131	-0.32391
O	-3.11937	-0.32668	1.49663

P	-1.56679	0.16214	1.18546
O	-1.18858	1.22364	2.16998
C	-6.65612	1.69447	-0.57452
C	-6.12409	4.43341	-0.8531
C	-7.42623	3.98511	-0.81259
C	-7.69087	2.60179	-0.66047
H	-6.87131	0.6335	-0.44828
H	-5.90632	5.50002	-0.9515
H	-8.25507	4.69316	-0.88449
H	-8.72427	2.25137	-0.60475
C	-6.08926	-1.05914	-3.78654
C	-6.20056	-2.46848	-3.70123
C	-5.71108	-3.12626	-2.59376
H	-6.45716	-0.53914	-4.67423
H	-6.6628	-3.0295	-4.51678
H	-5.76997	-4.21541	-2.52343
C	-4.37496	-0.27027	-0.52379
C	-5.09713	-2.41212	-1.52798
C	-5.01907	-0.98111	-1.59294
C	-5.51723	-0.33597	-2.76045
H	-5.4305	0.74623	-2.8469
C	-5.03368	3.52529	-0.7545
C	-1.22318	3.61454	-0.54693
C	-0.77876	4.34643	0.57904
C	-0.34519	3.36774	-1.62716
C	0.55613	4.90177	0.58774
C	0.99886	3.89845	-1.59195
C	1.40778	4.66692	-0.49704
C	-3.04493	-3.1375	1.60982
C	-3.44278	-3.05536	2.9631
C	-1.87071	-3.83929	1.24695
C	-2.63767	-3.68377	3.98441
C	-1.06847	-4.45769	2.27847
C	-1.47122	-4.36372	3.6155
H	2.425	5.06528	-0.47299
H	-0.85806	-4.83059	4.39134
C	-3.05145	-3.58962	5.35102
C	-4.1938	-2.91592	5.69853
C	-4.99314	-2.29864	4.69235
C	-4.6299	-2.36752	3.37141
H	-2.43238	-4.06834	6.11429
H	-4.49749	-2.84932	6.746
H	-5.90227	-1.76517	4.9796
H	-5.25018	-1.89286	2.61078

C	0.53302	-5.21796	0.60147
C	0.13566	-5.1397	1.91094
C	-1.40516	-3.93091	-0.1053
C	-0.25168	-4.60416	-0.41737
H	1.45387	-5.7428	0.3352
H	0.73284	-5.59818	2.70351
H	-1.96493	-3.43305	-0.89513
H	0.07572	-4.65069	-1.45724
C	1.88619	3.61489	-2.67832
C	1.48051	2.85469	-3.74455
C	0.15279	2.33488	-3.78671
C	-0.72964	2.59002	-2.76732
H	2.90233	4.01409	-2.63144
H	2.17063	2.64063	-4.56419
H	-0.16207	1.72341	-4.63526
H	-1.74173	2.18853	-2.81413
C	0.99226	5.64368	1.72986
C	0.17421	5.80764	2.81827
C	-1.12718	5.22571	2.82958
C	-1.58877	4.52017	1.74714
H	2.00331	6.05704	1.7228
H	0.5223	6.36934	3.68861
H	-1.75622	5.3308	3.71644
H	-2.57029	4.04963	1.77918
O	-0.69826	-1.02679	0.86894
H	0.48597	-1.69914	-0.11554

Zero-point correction= 1.151440 (Hartree/Particle)

Thermal correction to Energy= 1.217282

Thermal correction to Enthalpy= 1.218226

Thermal correction to Gibbs Free Energy= 1.049311

sp-E = -3947.92596481 hartree

TS3-major

N	0.66391	2.76365	0.91863
C	0.75975	3.35036	-0.40437
C	2.22084	0.96679	0.3545
C	1.40122	1.7088	1.30157
C	1.22929	1.23176	2.63445
H	0.46287	1.68678	3.26053
C	2.07055	0.27446	3.11162
H	1.99962	-0.03735	4.15648
C	3.07259	-0.3446	2.29932
C	3.07828	-0.12447	0.89769
C	3.98999	-0.91207	0.10784

C	3.87825	-1.13915	-1.32588
C	2.66092	-1.01007	-2.03326
H	1.73196	-0.82467	-1.50159
C	2.60707	-1.16871	-3.40867
H	1.6466	-1.06525	-3.91853
C	3.76407	-1.49829	-4.13866
H	3.71667	-1.61435	-5.22414
C	4.94674	-1.73763	-3.45999
H	5.83836	-2.06799	-3.99996
C	5.02079	-1.59941	-2.05513
C	6.20484	-1.97697	-1.34799
H	7.07173	-2.31896	-1.91917
C	6.21725	-1.99236	0.01421
H	7.0884	-2.36113	0.56078
C	5.10965	-1.4938	0.77413
C	5.14364	-1.57697	2.19043
H	5.99833	-2.05932	2.66978
C	4.09287	-1.10427	2.92813
H	4.05691	-1.23366	4.01145
H	1.12941	0.39477	-0.0714
H	0.1259	2.77734	-1.10757
C	0.33655	4.80427	-0.42437
C	-0.01705	5.38696	-1.64938
C	0.40063	5.61208	0.71625
C	-0.28586	6.75239	-1.73666
H	-0.09205	4.75998	-2.54143
C	0.11409	6.97802	0.63436
H	0.67407	5.16941	1.67535
C	-0.22431	7.55429	-0.59201
H	-0.55617	7.19186	-2.69979
H	0.16163	7.59517	1.5349
H	-0.44094	8.62315	-0.65681
C	5.18178	2.24175	1.3597
C	5.2221	2.01928	-0.02935
C	6.4921	1.90834	-0.6712
C	7.69569	2.00319	0.03658
C	7.62194	2.21162	1.41176
C	6.37727	2.33194	2.0656
H	4.22823	2.33265	1.8817
H	8.65942	1.90917	-0.46964
H	8.54383	2.28427	1.99363
H	6.35434	2.49578	3.14539
N	6.25399	1.68101	-2.01093
H	6.96257	1.51131	-2.7102

C	4.89339	1.61593	-2.22865
C	4.218	1.80902	-1.04593
C	2.72513	1.78815	-0.88201
H	2.30209	1.336	-1.78831
C	2.22172	3.23682	-0.82903
H	2.36043	3.69788	-1.81574
H	2.825	3.82078	-0.11566
H	4.49556	1.37099	-3.2111
C	-5.52104	-1.63156	0.48528
H	-6.48861	1.66932	0.754
C	-5.86029	0.80947	0.51089
C	-4.22927	-1.41876	-0.11095
C	-3.79571	-0.11392	-0.30454
C	-4.58856	1.02201	0.0201
C	-1.18566	-3.7257	-0.03691
C	-1.65741	-4.80747	-0.75893
C	-2.04277	-2.59287	0.09946
H	-1.0322	-5.69788	-0.84993
O	-2.52852	0.10299	-0.80381
O	-1.59514	-1.54844	0.864
P	-1.287	-0.03587	0.27779
O	-1.39496	0.91802	1.41635
C	-6.00013	-2.9187	0.86032
C	-7.6425	-0.69735	1.33067
C	-8.08801	-1.96106	1.64959
C	-7.24808	-3.07867	1.42494
H	-5.36426	-3.78878	0.7052
H	-8.26805	0.17814	1.52269
H	-9.07772	-2.10236	2.09005
H	-7.589	-4.07777	1.70658
C	-5.34665	-4.65989	-2.8203
C	-4.53317	-5.81596	-2.91646
C	-3.33828	-5.87092	-2.23344
H	-6.28189	-4.60796	-3.38279
H	-4.84975	-6.65393	-3.54184
H	-2.69073	-6.74784	-2.31389
C	-3.32634	-2.55416	-0.44055
C	-2.90956	-4.78355	-1.42179
C	-3.75039	-3.63077	-1.28964
C	-4.96674	-3.59745	-2.02775
H	-5.59896	-2.71198	-1.96672
C	-6.35387	-0.49689	0.76246
C	-4.03753	2.39934	-0.11668
C	-3.66063	3.11121	1.04547

C	-3.90207	2.98713	-1.39367
C	-3.16827	4.46377	0.92018
C	-3.41482	4.34231	-1.50589
C	-3.07152	5.0481	-0.34707
C	0.17593	-3.76259	0.56622
C	1.29554	-3.99708	-0.27513
C	0.35433	-3.63579	1.967
C	2.60671	-4.1628	0.31151
C	1.68168	-3.73686	2.52915
C	2.76565	-4.01801	1.69237
H	-2.69687	6.06919	-0.43404
H	3.7611	-4.12432	2.12684
C	3.71914	-4.46999	-0.53225
C	3.56797	-4.58518	-1.8887
C	2.2914	-4.3711	-2.47907
C	1.19812	-4.0797	-1.70299
H	4.69987	-4.5937	-0.06892
H	4.42783	-4.80747	-2.52336
H	2.18919	-4.41073	-3.56542
H	0.23771	-3.8869	-2.17838
C	0.79087	-3.34494	4.76631
C	1.85992	-3.57431	3.93989
C	-0.73594	-3.44196	2.87644
C	-0.52633	-3.29256	4.22368
H	0.93909	-3.22221	5.84211
H	2.87324	-3.64556	4.3421
H	-1.75182	-3.40903	2.48779
H	-1.37792	-3.13561	4.88972
C	-3.29052	4.92802	-2.80576
C	-3.61483	4.22014	-3.93476
C	-4.09427	2.88149	-3.82652
C	-4.23845	2.28864	-2.59757
H	-2.92681	5.95522	-2.87567
H	-3.51226	4.67871	-4.92136
H	-4.3505	2.32663	-4.7322
H	-4.61023	1.26617	-2.52489
C	-2.77149	5.17264	2.09931
C	-2.84412	4.5821	3.3346
C	-3.31713	3.24223	3.46005
C	-3.71004	2.53211	2.35499
H	-2.40167	6.19347	1.9862
H	-2.53683	5.13286	4.22725
H	-3.35098	2.7732	4.4462
H	-4.03275	1.498	2.45924

O	-0.02884	-0.07134	-0.60309
H	-0.2233	2.8658	1.41122

Zero-point correction= 1.147831 (Hartree/Particle)

Thermal correction to Energy= 1.213637

Thermal correction to Enthalpy= 1.214581

Thermal correction to Gibbs Free Energy= 1.046138

sp-E = -3947.91582799 hartree

TS3-minor

N	1.02859	2.40578	-2.23825
C	0.44765	3.07761	-1.08369
C	2.40782	0.90622	-0.91059
C	2.00457	1.49774	-2.17925
C	2.55369	1.05346	-3.41783
H	2.1157	1.40783	-4.35316
C	3.67752	0.29087	-3.40335
H	4.16493	0.02463	-4.34331
C	4.28022	-0.16079	-2.18944
C	3.61377	0.01473	-0.94722
C	4.22797	-0.59436	0.20794
C	3.56007	-0.85697	1.4727
C	2.16033	-0.97445	1.5761
H	1.56899	-0.94654	0.66947
C	1.53203	-1.18272	2.79121
H	0.44574	-1.25167	2.81733
C	2.29565	-1.30173	3.96677
H	1.79933	-1.45321	4.92824
C	3.67791	-1.28996	3.88837
H	4.28555	-1.4576	4.78196
C	4.33364	-1.11017	2.64942
C	5.75118	-1.25718	2.54132
H	6.33298	-1.44117	3.44823
C	6.3487	-1.25688	1.31643
H	7.41815	-1.45485	1.21489
C	5.60696	-0.96063	0.12688
C	6.26555	-1.02999	-1.12714
H	7.30779	-1.35427	-1.16068
C	5.581	-0.7228	-2.26958
H	6.04531	-0.82935	-3.25244
H	1.28283	0.23702	-0.91695
H	-0.37377	2.46829	-0.67565
C	-0.10239	4.41657	-1.52126
C	-1.44593	4.73366	-1.30101
C	0.72414	5.35318	-2.16238

C	-1.9554	5.9702	-1.70609
H	-2.09738	4.00823	-0.8119
C	0.216	6.58828	-2.56709
H	1.77439	5.11267	-2.34699
C	-1.1282	6.90059	-2.33767
H	-3.00743	6.18998	-1.52479
H	0.87053	7.31005	-3.06187
H	-1.52872	7.86672	-2.65416
C	5.2887	2.70613	-0.53092
C	4.70863	2.47723	0.73047
C	5.53424	2.56246	1.89239
C	6.90074	2.85352	1.81506
C	7.44721	3.06247	0.55073
C	6.64813	2.99229	-0.61012
H	4.68548	2.64859	-1.43814
H	7.52054	2.90681	2.71336
H	8.51279	3.28513	0.45708
H	7.1081	3.16201	-1.58643
N	4.73408	2.28562	2.98134
H	5.05052	2.21976	3.93807
C	3.4557	1.99949	2.54913
C	3.39064	2.09281	1.17826
C	2.16679	1.83136	0.3528
H	1.42866	1.34006	0.9978
C	1.55133	3.18952	-0.03655
H	1.14333	3.65555	0.87001
H	2.33521	3.85852	-0.42605
H	2.68353	1.68417	3.24735
H	0.65714	2.64559	-3.15024
C	-4.76382	-2.16755	1.53925
H	-5.96117	0.9509	2.36947
C	-5.32664	0.19484	1.90145
C	-3.76933	-1.75246	0.5932
C	-3.51596	-0.39272	0.42592
C	-4.32582	0.60375	1.04053
C	-0.80298	-3.63316	-0.88729
C	-1.44018	-4.57405	-1.67499
C	-1.61357	-2.72906	-0.14584
H	-0.83635	-5.28626	-2.24154
O	-2.46491	0.00318	-0.3754
O	-1.0024	-1.80632	0.67035
P	-0.92529	-0.21517	0.21511
O	-0.65513	0.61248	1.42254
C	-4.97769	-3.53292	1.88116

C	-6.54251	-1.57445	3.13983
C	-6.73694	-2.90589	3.43434
C	-5.93711	-3.89123	2.80402
H	-4.36364	-4.30003	1.41005
H	-7.14167	-0.80231	3.62911
H	-7.49756	-3.20472	4.15937
H	-6.07849	-4.94497	3.05585
C	-5.64207	-4.59043	-2.15296
C	-4.85295	-5.551	-2.83245
C	-3.48599	-5.5567	-2.66371
H	-6.72357	-4.57165	-2.30721
H	-5.32943	-6.27502	-3.49749
H	-2.86214	-6.27839	-3.19723
C	-3.0003	-2.74171	-0.2053
C	-2.85121	-4.61678	-1.80452
C	-3.6517	-3.67029	-1.08412
C	-5.05824	-3.67496	-1.30305
H	-5.67763	-2.93835	-0.79264
C	-5.55192	-1.17176	2.20165
C	-4.22651	2.0624	0.72897
C	-3.51533	2.93832	1.57894
C	-4.99587	2.5717	-0.34278
C	-3.63605	4.3679	1.39025
C	-5.11709	4.00164	-0.5184
C	-4.45807	4.85947	0.36921
C	0.68471	-3.57259	-0.86395
C	1.37469	-3.2227	-2.04945
C	1.40169	-3.93528	0.29843
C	2.81442	-3.33365	-2.0936
C	2.84605	-3.94669	0.26903
C	3.51334	-3.6711	-0.92963
H	-4.56965	5.9404	0.25135
H	4.60452	-3.71132	-0.95551
C	3.49048	-3.09389	-3.33134
C	2.80154	-2.70361	-4.45009
C	1.3918	-2.50135	-4.3845
C	0.70253	-2.75334	-3.22536
H	4.57448	-3.22178	-3.35752
H	3.33001	-2.52849	-5.39066
H	0.8569	-2.14255	-5.26715
H	-0.37264	-2.5845	-3.18176
C	2.89901	-4.55367	2.62846
C	3.56233	-4.24854	1.46889
C	0.75141	-4.29656	1.52082

C	1.47498	-4.59016	2.64809
H	3.45775	-4.76611	3.54242
H	4.65388	-4.21456	1.44179
H	-0.33752	-4.31694	1.55322
H	0.95651	-4.84248	3.57602
C	-5.91348	4.50229	-1.59717
C	-6.55203	3.64974	-2.46047
C	-6.43574	2.23897	-2.2889
C	-5.68919	1.71874	-1.26191
H	-5.99882	5.58536	-1.71922
H	-7.1538	4.04622	-3.28173
H	-6.94635	1.56869	-2.98437
H	-5.60591	0.63839	-1.1403
C	-2.90052	5.24362	2.24924
C	-2.09087	4.74348	3.23688
C	-1.97575	3.33533	3.42718
C	-2.67052	2.46096	2.63072
H	-2.98992	6.32143	2.09211
H	-1.5263	5.42329	3.87986
H	-1.31352	2.94773	4.20446
H	-2.54018	1.38831	2.75504
O	-0.01093	-0.13728	-1.014

Zero-point correction= 1.147692 (Hartree/Particle)

Thermal correction to Energy= 1.212902

Thermal correction to Enthalpy= 1.213846

Thermal correction to Gibbs Free Energy= 1.046864

sp-E = -3947.91259843 hartree

(*M*)-1-3-indol-quino[5]helicene

N	4.10408	-1.43136	0.70584
N	-2.31644	-1.60831	1.60011
H	-3.18636	-2.12107	1.5711
C	3.40827	-2.39682	1.27455
C	2.01624	-2.32867	1.47057
H	1.5173	-3.06378	2.10517
C	1.28455	-1.2733	0.93179
C	1.99539	-0.3298	0.11589
C	3.42471	-0.38385	0.17893
C	4.19	0.74949	-0.2541
H	5.27501	0.67755	-0.1653
C	3.5618	1.891	-0.65921
H	4.13595	2.79361	-0.88354
C	2.14052	1.92866	-0.84323

C	1.37637	0.74598	-0.643
C	0.02736	0.71951	-1.14545
C	-0.67984	-0.49627	-1.50499
C	-0.01581	-1.71439	-1.79758
H	1.07212	-1.74618	-1.7695
C	-0.72051	-2.85591	-2.12964
H	-0.18124	-3.77788	-2.35947
C	-2.13145	-2.83327	-2.17934
H	-2.6855	-3.74252	-2.4252
C	-2.80262	-1.64379	-1.96781
H	-3.89074	-1.59715	-2.06487
C	-2.09765	-0.44931	-1.67413
C	-2.76958	0.81407	-1.62175
H	-3.85707	0.83512	-1.72574
C	-2.05542	1.97436	-1.54004
H	-2.55793	2.94268	-1.59963
C	-0.6369	1.95839	-1.34721
C	0.1168	3.16725	-1.35571
H	-0.40537	4.10959	-1.53779
C	1.47963	3.13727	-1.20958
H	2.07464	4.04722	-1.31898
C	-0.13514	-1.12327	1.28319
C	-1.08992	-2.12034	1.24741
H	-0.98749	-3.15209	0.92154
C	-2.1919	-0.26241	1.88192
C	-0.82018	0.08074	1.70981
C	-0.41457	1.4026	1.96633
H	0.63018	1.69235	1.84758
C	-1.36743	2.33797	2.35248
H	-1.06237	3.3696	2.5418
C	-2.72553	1.98132	2.49834
H	-3.45152	2.74053	2.79859
C	-3.15438	0.67695	2.27209
H	-4.20334	0.39635	2.39262
H	3.97352	-3.25133	1.66671

Zero-point correction= 0.387406 (Hartree/Particle)
Thermal correction to Energy= 0.408401
Thermal correction to Enthalpy= 0.409345
Thermal correction to Gibbs Free Energy= 0.338915
sp-E = -1225.45161821 hartree

(*M*)-**4a**

N	1.7712	1.68773	2.45299
H	2.37347	2.36754	2.89433

C	-0.43037	-0.51569	-1.07235
C	-1.55509	-1.23214	-1.53391
C	-1.39861	-2.49159	-2.17635
H	-2.28974	-3.01546	-2.53251
C	-0.16622	-3.09073	-2.23212
H	-0.06584	-4.11013	-2.61339
C	0.98771	-2.41713	-1.76534
C	0.87507	-1.05326	-1.33379
C	2.12564	-0.35251	-1.0866
C	2.2853	1.08083	-1.19827
C	1.38985	1.89423	-1.93857
H	0.57903	1.4134	-2.48517
C	1.54765	3.26702	-2.00484
H	0.84535	3.86651	-2.58939
C	2.62284	3.89444	-1.33902
H	2.73398	4.98067	-1.37961
C	3.56607	3.11893	-0.68982
H	4.44244	3.5844	-0.23022
C	3.44688	1.70851	-0.64216
C	4.48974	0.89325	-0.1039
H	5.3621	1.37848	0.34139
C	4.43404	-0.46541	-0.23081
H	5.27243	-1.08875	0.08964
C	3.27907	-1.11597	-0.77066
C	3.29905	-2.52035	-1.02814
H	4.20969	-3.08281	-0.81024
C	2.22055	-3.12027	-1.61487
H	2.25602	-4.17217	-1.91
C	0.30435	0.79143	0.989
C	0.88724	1.95763	1.42819
H	0.78261	2.96618	1.03398
C	1.78954	0.3301	2.69365
C	0.85358	-0.27139	1.79936
C	0.68978	-1.66942	1.83073
H	-0.01007	-2.15894	1.15265
C	1.44988	-2.41968	2.72165
H	1.33627	-3.506	2.74428
C	2.37408	-1.80425	3.59322
H	2.96018	-2.42149	4.2784
C	2.55269	-0.42348	3.59351
H	3.26994	0.05383	4.26567
C	-0.68253	0.68413	-0.14344
H	-0.60377	1.62185	-0.71592
C	-2.11249	0.65152	0.4292

H	-2.3037	1.58914	0.96921
H	-2.21269	-0.17033	1.15606
C	-3.1548	0.45978	-0.67423
N	-2.84407	-0.78666	-1.33429
H	-3.58175	-1.24376	-1.85328
C	-4.56655	0.44829	-0.12513
C	-5.36196	1.59925	-0.19401
C	-5.08123	-0.69223	0.50877
C	-6.64419	1.61533	0.36058
H	-4.97142	2.49165	-0.69116
C	-6.36384	-0.68026	1.06076
H	-4.46596	-1.59302	0.56526
C	-7.14936	0.47418	0.98931
H	-7.2531	2.52043	0.29617
H	-6.75188	-1.57632	1.55149
H	-8.15342	0.48327	1.42011
H	-3.07013	1.31589	-1.37631

Zero-point correction= 0.514658 (Hartree/Particle)

Thermal correction to Energy= 0.541628

Thermal correction to Enthalpy= 0.542572

Thermal correction to Gibbs Free Energy= 0.456951

sp-E = -1458.90126573 hartree

(*M*)-**5a**-axially

N	-2.86176	1.8007	0.05386
N	2.29502	-1.28882	-2.21118
H	3.123	-1.23404	-2.78778
C	-3.02106	0.49886	-0.13929
C	-1.92663	-0.31261	-0.52671
H	-2.09907	-1.32307	-0.89283
C	-0.63263	0.19194	-0.54936
C	-0.43303	1.54805	-0.11487
C	-1.62228	2.33438	0.01454
C	-1.52339	3.76527	0.03723
H	-2.45602	4.32626	0.11291
C	-0.31413	4.37428	-0.12463
H	-0.24485	5.4614	-0.21382
C	0.90105	3.61498	-0.13837
C	0.85546	2.20531	0.05788
C	2.09368	1.53144	0.36769
C	2.17399	0.26841	1.07943
C	1.12319	-0.22446	1.89271
H	0.22056	0.37109	2.01586
C	1.22959	-1.43772	2.54526

H	0.40508	-1.78986	3.16908
C	2.38825	-2.22798	2.4009
H	2.44895	-3.20496	2.88453
C	3.45349	-1.74638	1.6667
H	4.37671	-2.3256	1.58602
C	3.39087	-0.4783	1.03689
C	4.54908	0.10087	0.42453
H	5.47126	-0.48489	0.39007
C	4.52738	1.39441	-0.01207
H	5.43773	1.87405	-0.38087
C	3.31156	2.15411	-0.01621
C	3.31351	3.52896	-0.3936
H	4.26421	4.00127	-0.65302
C	2.15429	4.25851	-0.35686
H	2.16381	5.33507	-0.54361
C	0.44068	-0.64895	-1.0972
C	1.45063	-0.23808	-1.94553
H	1.63715	0.7472	-2.36339
C	1.85768	-2.41607	-1.54568
C	0.6711	-2.05669	-0.84315
C	0.03279	-3.03148	-0.05569
H	-0.85992	-2.77896	0.51849
C	0.56882	-4.31316	0.00355
H	0.07884	-5.07486	0.61448
C	1.74355	-4.64751	-0.7038
H	2.14257	-5.66216	-0.63507
C	2.40427	-3.70359	-1.48438
H	3.3188	-3.95664	-2.02587
C	-4.39445	-0.05405	-0.00851
C	-4.63632	-1.43794	0.04402
C	-5.49037	0.82269	0.08203
C	-5.93581	-1.93078	0.17368
H	-3.80453	-2.14292	0.00078
C	-6.7876	0.33	0.20973
H	-5.29171	1.8942	0.04914
C	-7.01716	-1.04944	0.25419
H	-6.10345	-3.00967	0.2168
H	-7.62785	1.02585	0.27448
H	-8.03442	-1.43563	0.35461

Zero-point correction= 0.468559 (Hartree/Particle)

Thermal correction to Energy= 0.494334

Thermal correction to Enthalpy= 0.495279

Thermal correction to Gibbs Free Energy= 0.413199

sp-E = -1456.50574945 hartree

(*M*)-**5aj**

N	3.01324	1.55136	-0.08776
N	-1.49576	-2.88375	1.20498
H	-1.84546	-3.82524	1.09598
C	3.22762	0.2629	0.15802
C	2.15166	-0.59892	0.47291
H	2.34392	-1.58337	0.89872
C	0.83199	-0.17045	0.35626
C	0.60665	1.14837	-0.15703
C	1.74993	2.00585	-0.19272
C	1.55911	3.43077	-0.26549
H	2.45657	4.0511	-0.25248
C	0.30574	3.96153	-0.25631
H	0.15884	5.04365	-0.21056
C	-0.85815	3.12318	-0.36149
C	-0.6926	1.70975	-0.49627
C	-1.82197	0.95003	-0.92902
C	-1.74923	-0.38421	-1.55124
C	-0.65521	-0.8505	-2.29834
H	0.2042	-0.196	-2.4456
C	-0.64691	-2.1389	-2.82807
H	0.21633	-2.48576	-3.40015
C	-1.74425	-2.98607	-2.62419
H	-1.73739	-4.00066	-3.03023
C	-2.87039	-2.52129	-1.9436
H	-3.76062	-3.13892	-1.81088
C	-2.88238	-1.21737	-1.44183
C	-3.10042	1.50679	-0.81697
C	-3.26931	2.86328	-0.49289
H	-4.27647	3.2807	-0.41265
C	-2.15899	3.67684	-0.35264
H	-2.27294	4.75421	-0.21043
C	-0.24595	-1.03904	0.84789
C	-0.35373	-2.39734	0.61634
H	0.27671	-3.03736	0.00469
C	-2.16343	-1.85516	1.84027
C	-1.39336	-0.67203	1.65564
C	-1.84524	0.52613	2.23766
H	-1.27156	1.44651	2.12595
C	-3.04343	0.52469	2.94253
H	-3.4057	1.45403	3.38799
C	-3.80525	-0.65473	3.08827
H	-4.74746	-0.62198	3.64035

C	-3.3727	-1.86007	2.54477
H	-3.95666	-2.77596	2.66046
C	4.63789	-0.20547	0.1759
C	4.96661	-1.57207	0.15847
C	5.68266	0.73574	0.19452
C	6.29955	-1.98601	0.17078
H	4.17706	-2.3242	0.11494
C	7.01336	0.32196	0.20854
H	5.41797	1.79337	0.19802
C	7.32864	-1.04107	0.19907
H	6.53549	-3.05282	0.15227
H	7.81252	1.0672	0.22795
H	8.37213	-1.36522	0.20959
O	-4.00561	-0.75388	-0.82813
C	-4.27998	0.61525	-1.10851
H	-5.13529	0.89167	-0.47683
H	-4.59079	0.70867	-2.16999

Zero-point correction= 0.467224 (Hartree/Particle)

Thermal correction to Energy= 0.493318

Thermal correction to Enthalpy= 0.494262

Thermal correction to Gibbs Free Energy= 0.411426

sp-E = -1493.61555490 hartree

(*M*)-**5a**

N	2.97671	1.53868	-0.16612
N	-1.59289	-2.80591	1.26693
H	-1.9615	-3.74282	1.1842
C	3.19118	0.26993	0.15736
C	2.11238	-0.57756	0.51337
H	2.30279	-1.53605	0.99558
C	0.79595	-0.16086	0.36047
C	0.56913	1.12856	-0.2281
C	1.7116	1.98624	-0.30559
C	1.51694	3.39878	-0.46751
H	2.41018	4.02457	-0.49598
C	0.25673	3.92204	-0.48683
H	0.1039	5.00424	-0.50158
C	-0.89861	3.0743	-0.53012
C	-0.7296	1.66321	-0.59688
C	-1.86949	0.86053	-0.95713
C	-1.77432	-0.45409	-1.5652
C	-0.61285	-0.90773	-2.24045
H	0.2447	-0.24269	-2.32777
C	-0.55312	-2.16988	-2.80014

H	0.35209	-2.48841	-3.32244
C	-1.65756	-3.04455	-2.70537
H	-1.5998	-4.04807	-3.13405
C	-2.8275	-2.60485	-2.11548
H	-3.71109	-3.24847	-2.09086
C	-2.92795	-1.29685	-1.57787
C	-4.18135	-0.77819	-1.11929
H	-5.05112	-1.4395	-1.10953
C	-4.30621	0.54099	-0.79176
H	-5.28186	0.96103	-0.53604
C	-3.1645	1.40361	-0.74637
C	-3.30985	2.79805	-0.49304
H	-4.31514	3.20472	-0.35961
C	-2.21359	3.62167	-0.48698
H	-2.32768	4.70452	-0.39474
C	-0.29024	-1.00346	0.88249
C	-0.43798	-2.3597	0.66859
H	0.17136	-3.02517	0.06278
C	-2.22673	-1.75076	1.89272
C	-1.42326	-0.5926	1.68747
C	-1.83375	0.62686	2.25501
H	-1.23522	1.52834	2.11859
C	-3.02278	0.6703	2.97356
H	-3.35478	1.6163	3.40713
C	-3.81446	-0.48538	3.14839
H	-4.74652	-0.41798	3.71438
C	-3.42438	-1.71103	2.61727
H	-4.03081	-2.60871	2.75872
C	4.60174	-0.19461	0.21483
C	4.93194	-1.5592	0.28416
C	5.64551	0.74747	0.18324
C	6.26524	-1.9697	0.33136
H	4.14369	-2.31391	0.28186
C	6.97639	0.33726	0.23238
H	5.37972	1.80293	0.11971
C	7.29311	-1.0234	0.30891
H	6.50232	-3.03529	0.38041
H	7.77465	1.08343	0.21184
H	8.33681	-1.34475	0.347

Zero-point correction= 0.468384 (Hartree/Particle)

Thermal correction to Energy= 0.494196

Thermal correction to Enthalpy= 0.495140

Thermal correction to Gibbs Free Energy= 0.412959

sp-E = -1456.50595010 hartree

(*M*)-**5a**-axially

N	-3.08589	1.80282	0.09441
N	2.15642	-0.63413	-2.65104
H	2.93792	-0.44395	-3.26269
C	-3.16677	0.50927	-0.19882
C	-2.04136	-0.1954	-0.68626
H	-2.16498	-1.17889	-1.1358
C	-0.78091	0.39368	-0.69798
C	-0.65867	1.71202	-0.1444
C	-1.88538	2.41495	0.07147
C	-1.86106	3.84632	0.20054
H	-2.82098	4.34874	0.32878
C	-0.69041	4.53284	0.07428
H	-0.68604	5.62587	0.07168
C	0.57152	3.85015	-0.0033
C	0.58968	2.42049	0.07396
C	1.86019	1.8165	0.31751
C	1.79218	-0.62342	1.41136
C	0.47508	-0.67048	1.93195
H	-0.16643	0.20207	1.83535
C	0.01093	-1.79384	2.58855
H	-1.00543	-1.80205	2.98957
C	0.83819	-2.92832	2.7443
H	0.45482	-3.81821	3.24866
C	2.13531	-2.90086	2.27506
H	2.7912	-3.76395	2.41353
C	2.6589	-1.74917	1.63121
C	4.03902	-1.6806	1.26572
H	4.66364	-2.56045	1.43588
C	4.60123	-0.5229	0.77229
H	5.66689	-0.43537	0.55649
C	3.01856	2.5752	0.06444
C	3.01522	3.95263	-0.18056
H	3.9476	4.49589	-0.33882
C	1.78317	4.58304	-0.13988
H	1.72344	5.66952	-0.23666
C	0.32539	-0.2932	-1.37633
C	1.26287	0.30419	-2.19817
H	1.35739	1.34883	-2.48174
C	1.82533	-1.87464	-2.14018
C	0.65482	-1.70497	-1.34513
C	0.11845	-2.82704	-0.68936
H	-0.75786	-2.72647	-0.04944

C	0.74392	-4.06026	-0.83594
H	0.33873	-4.93247	-0.31831
C	1.903	-4.20413	-1.62669
H	2.37376	-5.18565	-1.7187
C	2.45939	-3.11299	-2.28861
H	3.36147	-3.21809	-2.89577
C	-4.49591	-0.14225	-0.06854
C	-4.64073	-1.54047	-0.09175
C	-5.64556	0.64942	0.10204
C	-5.89915	-2.12954	0.04086
H	-3.76315	-2.18076	-0.19567
C	-6.90194	0.06062	0.23245
H	-5.52146	1.73228	0.12887
C	-7.03532	-1.33173	0.20034
H	-5.99154	-3.2182	0.02477
H	-7.78556	0.69107	0.35959
H	-8.02052	-1.7931	0.30316
C	3.73659	0.56517	0.56092
C	2.35185	0.51919	0.75479
O	4.14596	1.80573	0.15261

Zero-point correction= 0.485778 (Hartree/Particle)
Thermal correction to Energy= 0.513297
Thermal correction to Enthalpy= 0.514241
Thermal correction to Gibbs Free Energy= 0.428361
sp-E = -1607.95375489 hartree

(*M*)-**5am**

N	-3.11637	-1.65554	-0.38356
N	1.44546	2.18109	2.07074
H	1.78616	3.12034	2.22122
C	-3.29314	-0.47358	0.19908
C	-2.19884	0.22722	0.75111
H	-2.36951	1.06855	1.42183
C	-0.88887	-0.19896	0.53792
C	-0.69416	-1.34018	-0.309
C	-1.86767	-2.11431	-0.58855
C	-1.72577	-3.46996	-1.04381
H	-2.64382	-4.03572	-1.20864
C	-0.49062	-4.02944	-1.16976
H	-0.38329	-5.0885	-1.4178
C	0.70334	-3.24208	-1.03495
C	0.58559	-1.83527	-0.78419
C	1.77055	-1.06098	-0.97023
C	1.40744	1.55292	-1.41367

C	0.04182	1.59805	-1.79265
H	-0.50786	0.66616	-1.90409
C	-0.59156	2.8022	-2.02686
H	-1.64543	2.81174	-2.31475
C	0.11536	4.02092	-1.89774
H	-0.39734	4.96982	-2.0719
C	1.46207	4.00482	-1.59339
H	2.02623	4.93999	-1.5402
C	2.15228	2.78227	-1.37443
C	3.56544	2.76431	-1.15735
H	4.0997	3.71734	-1.13784
C	4.26252	1.5819	-1.03207
H	5.34724	1.54927	-0.92351
C	3.00923	-1.7344	-0.99043
C	3.15039	-3.12126	-1.06824
H	4.13795	-3.58226	-1.10296
C	1.97949	-3.85409	-1.15875
H	2.02267	-4.93567	-1.3059
C	0.19523	0.49059	1.2485
C	0.29704	1.86314	1.39107
H	-0.3388	2.64114	0.97609
C	2.12322	1.02332	2.40182
C	1.35263	-0.07242	1.91661
C	1.81327	-1.37944	2.15777
H	1.23971	-2.24063	1.81371
C	3.01982	-1.55845	2.82566
H	3.38975	-2.57058	3.00396
C	3.77952	-0.45682	3.2742
H	4.72646	-0.63076	3.79038
C	3.33697	0.84789	3.07619
H	3.91612	1.70341	3.43119
C	-4.68579	0.03104	0.31669
C	-4.9638	1.36911	0.64584
C	-5.76385	-0.83907	0.07565
C	-6.2807	1.82141	0.74308
H	-4.14598	2.07325	0.80873
C	-7.07859	-0.38743	0.17484
H	-5.53781	-1.87213	-0.18968
C	-7.34375	0.94442	0.51133
H	-6.47735	2.86628	0.99546
H	-7.90461	-1.07857	-0.01074
H	-8.37472	1.29827	0.58837
C	3.51152	0.39438	-1.04094
C	2.11466	0.34478	-1.11857

O	4.05463	-0.85613	-0.978

Zero-point correction= 0.485751 (Hartree/Particle)

Thermal correction to Energy= 0.513279

Thermal correction to Enthalpy= 0.514223

Thermal correction to Gibbs Free Energy= 0.428041

sp-E = -1607.95426247 hartree

(*M*)-quino[5]helicene

N	3.77691	-1.42403	0.0058
C	3.30677	-2.50301	0.59632
C	1.99431	-2.59021	1.11142
H	1.67734	-3.47472	1.66769
C	1.13042	-1.53371	0.90655
C	1.56378	-0.38817	0.19176
C	2.9508	-0.35967	-0.15973
C	3.5342	0.85764	-0.63548
H	4.59191	0.83554	-0.90169
C	2.7975	2.00489	-0.65106
H	3.26044	2.95836	-0.91777
C	1.40233	2.00449	-0.32212
C	0.72921	0.77834	-0.05853
C	-0.71488	0.79767	0.03971
C	-1.58054	-0.35167	-0.19361
C	-1.15956	-1.51035	-0.89853
H	-0.15066	-1.54995	-1.30399
C	-2.00869	-2.58043	-1.10895
H	-1.65135	-3.45122	-1.66345
C	-3.33436	-2.54723	-0.62644
H	-3.99579	-3.40326	-0.77837
C	-3.80031	-1.40602	-0.00486
H	-4.84134	-1.33865	0.3214
C	-2.95923	-0.27966	0.18477
C	-3.49722	0.95653	0.66434
H	-4.54761	0.99167	0.96338
C	-2.73548	2.08776	0.65288
H	-3.16982	3.05436	0.9198
C	-1.34801	2.04576	0.29977
C	-0.61093	3.25936	0.17619
H	-1.12913	4.20567	0.34872
C	0.69929	3.23964	-0.21693
H	1.24389	4.16931	-0.39799
H	3.99687	-3.34879	0.70627
H	0.12047	-1.57187	1.31054

Zero-point correction= 0.277218 (Hartree/Particle)

Thermal correction to Energy= 0.291581
Thermal correction to Enthalpy= 0.292525
Thermal correction to Gibbs Free Energy= 0.236296
sp-E = -862.824800601 hartree

(*P*)-1-3-indol-quino[5]helicene

N	2.82929	-3.28627	0.35043
N	-1.66926	0.51967	-2.24653
H	-1.85926	1.27781	-2.88694
C	1.63817	-3.81934	0.16685
C	0.52275	-3.0785	-0.26499
H	-0.38865	-3.59639	-0.56659
C	0.60722	-1.69415	-0.39327
C	1.83897	-1.06628	0.00372
C	2.95169	-1.94205	0.22029
C	4.2827	-1.40888	0.22355
H	5.09839	-2.11862	0.369
C	4.49699	-0.08946	-0.04467
H	5.5121	0.30118	-0.15186
C	3.4027	0.82977	-0.14879
C	2.07223	0.37315	0.0692
C	1.05223	1.36891	0.29493
C	-0.17978	1.1191	1.02173
C	-0.33495	0.03607	1.92244
H	0.50554	-0.63152	2.10269
C	-1.52648	-0.17821	2.58842
H	-1.61531	-1.01853	3.28051
C	-2.63006	0.67181	2.37081
H	-3.58185	0.47204	2.86703
C	-2.49045	1.77122	1.5478
H	-3.32195	2.46632	1.40732
C	-1.2599	2.04573	0.90052
C	-1.05612	3.27309	0.19065
H	-1.89376	3.96896	0.09672
C	0.1856	3.60974	-0.2665
H	0.369	4.59081	-0.71224
C	1.27826	2.68467	-0.1912
C	2.59025	3.07422	-0.59047
H	2.75334	4.09991	-0.9305
C	3.63705	2.19828	-0.47439
H	4.66167	2.51936	-0.67672
C	-0.51445	-0.96674	-1.0026
C	-0.41701	0.04941	-1.93371
H	0.4711	0.48622	-2.38168

C	-2.62003	-0.17889	-1.53043
C	-1.92905	-1.14594	-0.74416
C	-2.67599	-1.9809	0.10582
H	-2.17413	-2.71153	0.74176
C	-4.0595	-1.84664	0.14423
H	-4.64558	-2.49151	0.80312
C	-4.7228	-0.88348	-0.64587
H	-5.81068	-0.79956	-0.59152
C	-4.01219	-0.03588	-1.4905
H	-4.52145	0.7168	-2.09692
H	1.55293	-4.90454	0.30323

Zero-point correction= 0.387574 (Hartree/Particle)

Thermal correction to Energy= 0.408534

Thermal correction to Enthalpy= 0.409479

Thermal correction to Gibbs Free Energy= 0.339125

sp-E = -1225.45112930 hartree

(*P*)-**4a**

N	-2.50794	2.29632	-0.17772
H	-3.18683	2.98283	-0.47416
C	0.75924	-1.26133	-0.03798
C	-0.0309	-2.36638	0.33116
C	0.51282	-3.41432	1.11391
H	-0.13736	-4.22635	1.45083
C	1.8677	-3.46144	1.33748
H	2.30974	-4.32232	1.84535
C	2.7253	-2.45382	0.83561
C	2.16201	-1.26532	0.25171
C	3.10789	-0.21207	-0.1137
C	2.78746	1.20006	-0.19402
C	1.69227	1.76549	0.5005
H	1.09173	1.1275	1.13992
C	1.39937	3.11403	0.42209
H	0.54116	3.50675	0.97229
C	2.20436	3.97122	-0.35583
H	1.96091	5.03369	-0.43307
C	3.33594	3.46927	-0.97326
H	4.01007	4.13461	-1.51938
C	3.67872	2.09847	-0.86727
C	4.9441	1.61223	-1.32108
H	5.61469	2.29475	-1.84936
C	5.34457	0.34751	-0.99718
H	6.35496	0.00624	-1.23692
C	4.46169	-0.57325	-0.34414

C	4.95918	-1.83019	0.11959
H	6.0184	-2.05936	-0.01892
C	4.13341	-2.6876	0.78806
H	4.51983	-3.61353	1.22168
C	-0.93009	0.67956	-0.30542
C	-1.64135	1.62405	-1.01203
H	-1.59945	1.86524	-2.07187
C	-2.38975	1.79411	1.09773
C	-1.39172	0.77188	1.06258
C	-1.08586	0.1032	2.2665
H	-0.32345	-0.67369	2.29004
C	-1.77127	0.4415	3.42866
H	-1.53571	-0.07861	4.36015
C	-2.76497	1.44425	3.43109
H	-3.28717	1.68465	4.36015
C	-3.08252	2.13477	2.26601
H	-3.84836	2.91418	2.25963
C	0.0532	-0.27715	-0.95914
H	0.81277	0.31296	-1.48814
C	-0.68988	-1.12726	-2.01707
H	0.03961	-1.79119	-2.50647
H	-1.12363	-0.48663	-2.79686
C	-1.80669	-1.97415	-1.37647
N	-1.35617	-2.44909	-0.07108
H	-1.83644	-3.27964	0.25331
C	-3.15806	-1.26708	-1.30162
C	-3.81004	-0.91415	-2.49319
C	-3.77781	-0.96694	-0.08467
C	-5.03231	-0.24181	-2.46942
H	-3.35118	-1.16272	-3.45489
C	-5.00124	-0.29167	-0.05656
H	-3.27805	-1.23539	0.84496
C	-5.6311	0.0797	-1.24608
H	-5.52233	0.02917	-3.40819
H	-5.45436	-0.04595	0.90686
H	-6.58755	0.60806	-1.22355
H	-1.95525	-2.84793	-2.04098

Zero-point correction= 0.515653 (Hartree/Particle)

Thermal correction to Energy= 0.542138

Thermal correction to Enthalpy= 0.543083

Thermal correction to Gibbs Free Energy= 0.459904

sp-E = -1458.89461270 hartree

(*P*)-**5aj**

N	-2.88127	-1.82311	0.01567
N	2.23919	1.34593	-2.19673
H	3.09614	1.29407	-2.72977
C	-3.04701	-0.51415	-0.13307
C	-1.9588	0.31324	-0.49433
H	-2.1369	1.33326	-0.82912
C	-0.66041	-0.18584	-0.53491
C	-0.45696	-1.5511	-0.14322
C	-1.64047	-2.34675	-0.04032
C	-1.53483	-3.78121	-0.07276
H	-2.46747	-4.3456	-0.02735
C	-0.32635	-4.38193	-0.24477
H	-0.2539	-5.46558	-0.36719
C	0.89246	-3.6205	-0.21241
C	0.83596	-2.20923	0.0151
C	2.0553	-1.55549	0.38153
C	2.14409	-0.25403	1.06976
C	1.16967	0.24825	1.94775
H	0.28235	-0.34987	2.15635
C	1.31831	1.50159	2.53642
H	0.54523	1.88058	3.20779
C	2.44356	2.28298	2.24977
H	2.54415	3.28049	2.68189
C	3.44951	1.78345	1.42389
H	4.35309	2.35546	1.20785
C	3.3127	0.50918	0.87145
C	3.27534	-2.19142	0.1227
C	3.31889	-3.53471	-0.29052
H	4.28299	-4.01412	-0.48033
C	2.1446	-4.25931	-0.37397
H	2.16524	-5.33128	-0.58438
C	0.40599	0.66983	-1.06982
C	1.42266	0.27737	-1.91992
H	1.62916	-0.70515	-2.33451
C	1.78264	2.46488	-1.53045
C	0.60649	2.08359	-0.82203
C	-0.04605	3.04651	-0.03181
H	-0.92944	2.77791	0.54936
C	0.46615	4.33829	0.02401
H	-0.03448	5.09075	0.63772
C	1.63139	4.69344	-0.68825
H	2.01222	5.71522	-0.62144
C	2.30589	3.76163	-1.47196
H	3.2144	4.03015	-2.01588

C	-4.42331	0.02616	0.01757
C	-4.67263	1.40621	0.11658
C	-5.51425	-0.85912	0.0796
C	-5.97462	1.8873	0.26329
H	-3.84457	2.1166	0.09653
C	-6.81411	-0.37805	0.22463
H	-5.30986	-1.92782	0.01073
C	-7.05112	0.99779	0.31514
H	-6.14811	2.96321	0.34254
H	-7.65048	-1.08028	0.26681
H	-8.07036	1.37487	0.42917
O	4.32881	-0.0065	0.1131
C	4.53251	-1.39488	0.36166
H	4.89075	-1.52495	1.40381
H	5.33475	-1.7197	-0.31594

Zero-point correction= 0.467430 (Hartree/Particle)
Thermal correction to Energy= 0.493457
Thermal correction to Enthalpy= 0.494402
Thermal correction to Gibbs Free Energy= 0.411879
sp-E = -1493.61650290 hartree

(*P*)-**5a**

N	-2.86149	-1.80099	0.05377
N	2.29451	1.28939	-2.21176
H	3.12216	1.2349	-2.78884
C	-3.02091	-0.49914	-0.13922
C	-1.92658	0.31248	-0.52659
H	-2.09916	1.32295	-0.8926
C	-0.63253	-0.19195	-0.54936
C	-0.43279	-1.54808	-0.11502
C	-1.62196	-2.33455	0.01433
C	-1.52292	-3.76543	0.03681
H	-2.4555	-4.32652	0.11244
C	-0.31361	-4.37428	-0.12519
H	-0.24421	-5.46138	-0.21455
C	0.90149	-3.61486	-0.13883
C	0.85577	-2.20522	0.05764
C	2.0939	-1.53129	0.36764
C	2.17408	-0.26839	1.07966
C	1.12318	0.22427	1.89294
H	0.22053	-0.37131	2.01583
C	1.2295	1.43733	2.54587
H	0.40491	1.78926	3.16971
C	2.3882	2.22761	2.40194

H	2.44886	3.20442	2.8859
C	3.45354	1.7462	1.66775
H	4.37679	2.32541	1.58741
C	3.39098	0.47832	1.03753
C	4.54929	-0.10067	0.42521
H	5.47147	0.4851	0.39107
C	4.52767	-1.39409	-0.01175
H	5.43809	-1.87362	-0.38053
C	3.31187	-2.15381	-0.01625
C	3.31395	-3.52859	-0.39391
H	4.26472	-4.00077	-0.65335
C	2.1548	-4.25824	-0.35738
H	2.16441	-5.33476	-0.54433
C	0.44064	0.64909	-1.09725
C	1.45046	0.23845	-1.94582
H	1.63711	-0.7468	-2.36372
C	1.857	2.41658	-1.54627
C	0.67075	2.0569	-0.84334
C	0.03238	3.03157	-0.05576
H	-0.86006	2.77884	0.51871
C	0.56803	4.31344	0.00317
H	0.07801	5.07503	0.61419
C	1.74242	4.64808	-0.70459
H	2.14114	5.66287	-0.6361
C	2.40321	3.70428	-1.48527
H	3.31748	3.95757	-2.02708
C	-4.39434	0.05364	-0.0083
C	-4.63626	1.4375	0.04472
C	-5.49022	-0.82319	0.08185
C	-5.93579	1.93023	0.17448
H	-3.80449	2.14252	0.0018
C	-6.78748	-0.3306	0.20965
H	-5.29152	-1.89468	0.0486
C	-7.0171	1.04882	0.2546
H	-6.10348	3.0091	0.21799
H	-7.6277	-1.02651	0.27409
H	-8.03439	1.43493	0.3551

Zero-point correction= 0.468557 (Hartree/Particle)

Thermal correction to Energy= 0.494334

Thermal correction to Enthalpy= 0.495278

Thermal correction to Gibbs Free Energy= 0.413193

sp-E = -1456.50575450 hartree

(*P*)-quino[5]helicene

N	-3.77691	-1.42403	0.00581
C	-3.30678	-2.503	0.59634
C	-1.9943	-2.59021	1.11143
H	-1.67735	-3.47471	1.6677
C	-1.13042	-1.53371	0.90655
C	-1.56378	-0.38817	0.19176
C	-2.9508	-0.35966	-0.15973
C	-3.5342	0.85763	-0.63549
H	-4.59191	0.83554	-0.9017
C	-2.79749	2.00489	-0.65108
H	-3.26044	2.95835	-0.9178
C	-1.40233	2.00448	-0.32214
C	-0.72922	0.77834	-0.05853
C	0.71488	0.79767	0.03971
C	1.58054	-0.35166	-0.19361
C	1.15957	-1.51035	-0.89854
H	0.15066	-1.54995	-1.304
C	2.0087	-2.58042	-1.10896
H	1.65136	-3.45121	-1.66347
C	3.33437	-2.54722	-0.62645
H	3.9958	-3.40325	-0.77839
C	3.80031	-1.40602	-0.00486
H	4.84134	-1.33864	0.32139
C	2.95923	-0.27966	0.18477
C	3.49722	0.95653	0.66435
H	4.5476	0.99167	0.96339
C	2.73547	2.08776	0.6529
H	3.16981	3.05436	0.91984
C	1.34801	2.04575	0.29978
C	0.61092	3.25936	0.1762
H	1.12912	4.20567	0.34875
C	-0.69929	3.23964	-0.21694
H	-1.24389	4.16931	-0.39801
H	-3.99687	-3.34879	0.70629
H	-0.12046	-1.57187	1.31053

Zero-point correction= 0.277218 (Hartree/Particle)

Thermal correction to Energy= 0.291581

Thermal correction to Enthalpy= 0.292525

Thermal correction to Gibbs Free Energy= 0.236296

sp-E = -862.824800626 hartree

TS-1-3-indol-quino[5]helicene

N	-0.47542	-3.39464	2.26209
N	3.57687	1.1707	0.77135

H	4.27435	1.85584	1.02668
C	0.22593	-2.67841	3.11894
C	0.88915	-1.51476	2.71523
H	1.70455	-1.12089	3.32507
C	0.66237	-0.97085	1.44573
C	-0.43797	-1.49729	0.67612
C	-0.76658	-2.85056	1.06001
C	-1.37433	-3.73315	0.10875
H	-1.42527	-4.78943	0.37755
C	-1.87735	-3.23477	-1.047
H	-2.35432	-3.88584	-1.78318
C	-2.01648	-1.81341	-1.19701
C	-1.29355	-0.88698	-0.37699
C	-1.77767	0.49734	-0.40536
C	-1.10579	1.71355	0.04943
C	0.25702	1.96022	-0.20682
H	0.80955	1.23361	-0.78908
C	0.8712	3.14629	0.16763
H	1.9219	3.30505	-0.08123
C	0.14341	4.13774	0.84765
H	0.63413	5.05722	1.1764
C	-1.22245	3.9784	1.01226
H	-1.82772	4.78645	1.43134
C	-1.87891	2.81798	0.5397
C	-3.29823	2.81959	0.34705
H	-3.88968	3.65307	0.73326
C	-3.85034	1.8722	-0.45945
H	-4.89811	1.93549	-0.76362
C	-3.1015	0.72372	-0.88961
C	-3.71176	-0.20355	-1.77298
H	-4.6685	0.05396	-2.23366
C	-3.11262	-1.40708	-2.00932
H	-3.56873	-2.13965	-2.67882
C	1.76003	-0.1601	0.90585
C	2.53728	0.76058	1.57688
H	2.38557	1.20007	2.55981
C	3.5194	0.50164	-0.43597
C	2.38632	-0.36101	-0.38541
C	2.09496	-1.17319	-1.49611
H	1.23247	-1.84099	-1.48091
C	2.92151	-1.10304	-2.61336
H	2.70528	-1.72725	-3.48322
C	4.03676	-0.23843	-2.64563
H	4.66447	-0.20527	-3.53907

C	4.35269	0.57255	-1.55879
H	5.21838	1.23855	-1.58226
H	0.37671	-3.0937	4.12244

Zero-point correction= 0.386353 (Hartree/Particle)
Thermal correction to Energy= 0.406661
Thermal correction to Enthalpy= 0.407605
Thermal correction to Gibbs Free Energy= 0.338798
sp-E = -1225.39301975 hartree

TS-**4a**

N	-2.34048	2.28884	-0.49947
H	-3.07838	2.94487	-0.71281
C	0.4739	-1.5997	-0.30839
C	-0.28075	-2.55874	0.40207
C	0.33463	-3.58544	1.15233
H	-0.28132	-4.31124	1.68932
C	1.69134	-3.70682	1.09445
H	2.19308	-4.5493	1.57538
C	2.49159	-2.74414	0.43395
C	1.92455	-1.55688	-0.18107
C	2.9072	-0.45286	-0.45877
C	2.656	0.97784	-0.76949
C	1.52987	1.51385	-1.43459
H	0.8399	0.85328	-1.91587
C	1.2792	2.86898	-1.57019
H	0.36522	3.18416	-2.07838
C	2.18027	3.81775	-1.0659
H	1.97396	4.88779	-1.14014
C	3.36743	3.35693	-0.53573
H	4.14334	4.05792	-0.2175
C	3.64779	1.9718	-0.4318
C	4.97712	1.58331	-0.10282
H	5.7254	2.3543	0.09505
C	5.29897	0.26715	-0.11588
H	6.32608	-0.05849	0.06215
C	4.29124	-0.74666	-0.22222
C	4.76925	-2.05473	0.07871
H	5.84686	-2.2227	0.12695
C	3.88735	-3.01376	0.44422
H	4.22448	-3.98907	0.80285
C	-1.03364	0.45212	-0.66367
C	-1.94436	1.25356	-1.31749
H	-2.36884	1.15078	-2.31316
C	-1.70921	2.17304	0.71913

C	-0.85787	1.02871	0.65119
C	-0.08243	0.7087	1.78435
H	0.59332	-0.14393	1.76953
C	-0.18524	1.49905	2.92376
H	0.41658	1.25299	3.80172
C	-1.04687	2.61624	2.9697
H	-1.10527	3.21543	3.88151
C	-1.81705	2.96862	1.86647
H	-2.48167	3.83565	1.89132
C	-0.40772	-0.79311	-1.25736
H	0.21199	-0.54822	-2.12867
C	-1.50136	-1.71092	-1.84783
H	-1.00958	-2.57144	-2.32834
H	-2.06099	-1.17941	-2.62971
C	-2.4674	-2.22169	-0.77354
N	-1.67332	-2.55408	0.39619
H	-2.07982	-3.27307	0.98149
C	-3.62041	-1.27405	-0.45422
C	-4.6143	-1.05594	-1.41892
C	-3.70967	-0.60282	0.76925
C	-5.65313	-0.15352	-1.18322
H	-4.56914	-1.59108	-2.37258
C	-4.74647	0.302	1.00851
H	-2.93521	-0.76777	1.51783
C	-5.71873	0.53603	0.03242
H	-6.41726	0.00891	-1.94784
H	-4.78364	0.83546	1.96139
H	-6.53041	1.24353	0.22001
H	-2.92863	-3.14616	-1.17321

Zero-point correction= 0.515445 (Hartree/Particle)
Thermal correction to Energy= 0.541263
Thermal correction to Enthalpy= 0.542207
Thermal correction to Gibbs Free Energy= 0.460926
sp-E = -1458.85840406 hartree

TS-**5a**-axially

N	2.16266	-2.11963	-0.7001
N	-1.86366	2.95052	-0.97586
H	-2.76792	3.39749	-1.03915
C	2.57026	-1.01036	-0.09563
C	1.71433	0.1084	0.00312
H	2.11779	1.05582	0.34203
C	0.37378	0.05382	-0.38443
C	-0.13626	-1.26775	-0.66722

C	0.85592	-2.24524	-1.00757
C	0.46872	-3.39893	-1.77029
H	1.2664	-4.0809	-2.06868
C	-0.82789	-3.57584	-2.15108
H	-1.11211	-4.40053	-2.80925
C	-1.86276	-2.73757	-1.62294
C	-1.52549	-1.68187	-0.72527
C	-2.57823	-1.15164	0.10649
C	-2.3542	-0.50986	1.39614
C	-1.14277	-0.62142	2.12364
H	-0.34377	-1.24989	1.74062
C	-0.96152	0.02714	3.33119
H	-0.01606	-0.08758	3.86608
C	-1.98981	0.8213	3.87903
H	-1.8343	1.34538	4.82483
C	-3.20764	0.89473	3.232
H	-4.03446	1.46057	3.66911
C	-3.42683	0.21348	2.00874
C	-4.72753	0.19154	1.40972
H	-5.53142	0.76151	1.88222
C	-4.97721	-0.59671	0.32374
H	-5.98994	-0.68813	-0.07688
C	-3.92515	-1.32064	-0.32574
C	-4.22175	-2.21837	-1.38986
H	-5.26282	-2.34424	-1.69658
C	-3.2261	-2.97211	-1.95519
H	-3.45944	-3.74701	-2.68941
C	-0.31622	1.33481	-0.61436
C	-1.67253	1.63112	-0.67303
H	-2.53413	0.99726	-0.52709
C	-0.64863	3.57576	-1.1512
C	0.3644	2.59245	-0.95199
C	1.6984	2.98801	-1.18931
H	2.51913	2.27572	-1.12701
C	1.97761	4.30595	-1.54158
H	3.01411	4.59986	-1.72225
C	0.95433	5.2637	-1.68007
H	1.20559	6.29156	-1.95128
C	-0.3765	4.90381	-1.49652
H	-1.18477	5.6267	-1.63044
C	3.97885	-0.96017	0.37675
C	4.45416	0.07708	1.19844
C	4.86998	-1.98306	0.00553
C	5.78183	0.09527	1.6289

H	3.78268	0.87368	1.52287
C	6.19594	-1.96296	0.43381
H	4.49022	-2.78703	-0.62535
C	6.65939	-0.92268	1.24641
H	6.1311	0.908	2.27039
H	6.87503	-2.76434	0.13205
H	7.69899	-0.90724	1.58259

Zero-point correction= 0.468743 (Hartree/Particle)

Thermal correction to Energy= 0.493686

Thermal correction to Enthalpy= 0.494630

Thermal correction to Gibbs Free Energy= 0.414533

sp-E = -1456.48988195 hartree

TS-**5aj**

N	-2.83048	-1.07099	0.98706
N	1.0162	3.63008	-0.65573
H	1.27722	4.40892	-1.2441
C	-3.0613	-0.18401	0.02808
C	-2.01666	0.66604	-0.38341
H	-2.2493	1.59551	-0.9027
C	-0.70167	0.45518	0.03392
C	-0.39801	-0.75873	0.74502
C	-1.56631	-1.33461	1.36398
C	-1.42319	-2.21495	2.48907
H	-2.33259	-2.47268	3.03396
C	-0.20595	-2.71629	2.80384
H	-0.07902	-3.40493	3.64244
C	0.9008	-2.52215	1.90673
C	0.87027	-1.51388	0.87777
C	1.94264	-1.58584	-0.09397
C	2.37996	-0.59689	-1.11672
C	2.76228	0.72049	-0.8343
H	2.70244	1.07796	0.18882
C	3.25825	1.56025	-1.83341
H	3.54744	2.58127	-1.57877
C	3.38365	1.09305	-3.14448
H	3.74572	1.75437	-3.93576
C	3.13863	-0.25376	-3.42544
H	3.34542	-0.68652	-4.40588
C	2.71397	-1.09526	-2.39739
C	2.69799	-2.77064	-0.17781
C	2.7326	-3.70343	0.86596
H	3.38951	-4.57488	0.80255
C	1.90416	-3.5162	1.94957

H	1.90366	-4.22534	2.78031
C	0.16941	1.6346	-0.03486
C	0.26564	2.54969	-1.06203
H	-0.10992	2.47997	-2.08009
C	1.4092	3.45464	0.65644
C	0.87862	2.20539	1.09099
C	1.12435	1.77721	2.40743
H	0.72692	0.82395	2.76005
C	1.89067	2.58429	3.24286
H	2.0922	2.26248	4.26704
C	2.41551	3.81466	2.7913
H	3.01577	4.42371	3.47108
C	2.18045	4.2678	1.4957
H	2.58317	5.2221	1.14831
C	-4.45013	-0.0406	-0.4722
C	-4.73932	0.67442	-1.64715
C	-5.50999	-0.64408	0.22755
C	-6.05286	0.79375	-2.10354
H	-3.9296	1.12503	-2.22414
C	-6.82148	-0.52329	-0.22808
H	-5.2731	-1.205	1.1321
C	-7.09952	0.19829	-1.39416
H	-6.2594	1.34888	-3.0218
H	-7.63486	-0.99445	0.32941
H	-8.12797	0.29187	-1.75134
O	2.74628	-2.45022	-2.57266
C	3.43549	-3.03729	-1.46814
H	4.47533	-2.65253	-1.4446
H	3.47176	-4.11865	-1.65959

Zero-point correction= 0.466537 (Hartree/Particle)

Thermal correction to Energy= 0.491825

Thermal correction to Enthalpy= 0.492769

Thermal correction to Gibbs Free Energy= 0.411947

sp-E = -1493.56795830 hartree

TS-**5a**

N	-2.73891	-0.91715	1.12855
N	0.97845	3.7055	-0.88328
H	1.17405	4.48296	-1.49837
C	-2.99524	-0.16425	0.06755
C	-1.97191	0.66082	-0.44102
H	-2.2348	1.53685	-1.03417
C	-0.65048	0.52252	-0.01434
C	-0.31368	-0.62474	0.79437

C	-1.46505	-1.11407	1.51542
C	-1.28419	-1.80683	2.75706
H	-2.17455	-1.98304	3.36272
C	-0.04978	-2.24349	3.10849
H	0.1113	-2.7899	4.04064
C	1.01065	-2.2126	2.14098
C	0.94862	-1.38495	0.97206
C	1.93449	-1.68564	-0.07166
C	2.34384	-0.8619	-1.20695
C	2.56129	0.52475	-1.08902
H	2.4506	0.98061	-0.11316
C	3.01513	1.28923	-2.15366
H	3.19954	2.35464	-2.00537
C	3.24797	0.69413	-3.40546
H	3.57053	1.30083	-4.25528
C	3.15532	-0.68244	-3.52611
H	3.44456	-1.17748	-4.45681
C	2.79387	-1.48444	-2.41843
C	3.07167	-2.88947	-2.42221
H	3.39973	-3.36736	-3.34831
C	3.08409	-3.55835	-1.23667
H	3.44853	-4.5869	-1.17668
C	2.56484	-2.96582	-0.03503
C	2.65569	-3.68778	1.18302
H	3.25371	-4.60169	1.21411
C	1.9784	-3.24632	2.28322
H	2.03035	-3.78896	3.22968
C	0.17769	1.72727	-0.15153
C	0.21413	2.60746	-1.21247
H	-0.20662	2.49812	-2.2091
C	1.43436	3.58005	0.4148
C	0.93808	2.34241	0.91745
C	1.25132	1.96445	2.2355
H	0.88346	1.02111	2.64168
C	2.0426	2.81117	3.00575
H	2.29457	2.5287	4.03033
C	2.5281	4.03127	2.48802
H	3.14882	4.67248	3.11804
C	2.22944	4.43368	1.18896
H	2.60183	5.38003	0.78984
C	-4.38717	-0.11443	-0.43946
C	-4.69239	0.4438	-1.69269
C	-5.43294	-0.65007	0.33299
C	-6.0085	0.47765	-2.15601

H	-3.89206	0.83668	-2.32277
C	-6.7469	-0.61501	-0.13002
H	-5.18331	-1.08951	1.29925
C	-7.04134	-0.04864	-1.37524
H	-6.22783	0.91077	-3.13497
H	-7.54954	-1.03154	0.48371
H	-8.07181	-0.02226	-1.73784

Zero-point correction= 0.467343 (Hartree/Particle)
Thermal correction to Energy= 0.492453
Thermal correction to Enthalpy= 0.493397
Thermal correction to Gibbs Free Energy= 0.413020
sp-E = -1456.44819747 hartree

TS-(M)-**5a**-axially

N	2.32134	-2.2149	-0.59039
N	-1.66845	2.78358	-1.64785
H	-2.5574	3.25049	-1.76277
C	2.68197	-1.03744	-0.08659
C	1.82395	0.07439	-0.18714
H	2.19879	1.06372	0.05501
C	0.50408	-0.04819	-0.63546
C	0.02317	-1.3962	-0.80163
C	1.04465	-2.38906	-0.97671
C	0.72213	-3.61076	-1.66497
H	1.54585	-4.30226	-1.8484
C	-0.53968	-3.83862	-2.1207
H	-0.76557	-4.72234	-2.72252
C	-1.62703	-2.98235	-1.7341
C	-1.35359	-1.83782	-0.91269
C	-2.47451	-1.28611	-0.21704
C	-1.8992	0.14969	1.98517
C	-0.54556	-0.18923	2.22864
H	-0.07655	-0.96029	1.62665
C	0.17148	0.41409	3.24388
H	1.2147	0.13211	3.40516
C	-0.43628	1.38083	4.07498
H	0.14192	1.86142	4.86745
C	-1.77108	1.68801	3.90468
H	-2.26283	2.40173	4.57077
C	-2.54296	1.07138	2.88455
C	-3.94726	1.31979	2.78587
H	-4.40042	2.02118	3.49038
C	-4.73423	0.66322	1.86609
H	-5.81479	0.80181	1.81198

C	-3.77036	-1.62722	-0.6621
C	-4.04535	-2.60721	-1.61717
H	-5.07183	-2.83494	-1.9063
C	-2.95761	-3.31609	-2.09785
H	-3.11325	-4.16285	-2.77024
C	-0.16918	1.19067	-1.0614
C	-1.51377	1.52442	-1.13403
H	-2.38557	0.96116	-0.83984
C	-0.43794	3.32145	-1.95632
C	0.54424	2.34688	-1.61618
C	1.88667	2.63841	-1.93616
H	2.67743	1.91096	-1.75776
C	2.20673	3.86468	-2.51227
H	3.24878	4.08325	-2.75653
C	1.21536	4.82538	-2.79467
H	1.49857	5.78117	-3.24135
C	-0.12367	4.55914	-2.52856
H	-0.90572	5.28345	-2.76839
C	4.04266	-0.9237	0.49812
C	4.42121	0.17893	1.28436
C	4.98003	-1.94962	0.28339
C	5.70314	0.2582	1.83093
H	3.70584	0.9773	1.48964
C	6.2607	-1.86799	0.82712
H	4.67298	-2.80546	-0.31832
C	6.62938	-0.76288	1.6019
H	5.97798	1.12058	2.44313
H	6.97859	-2.67194	0.64625
H	7.63327	-0.69988	2.02881
C	-4.08305	-0.20801	0.97543
C	-2.69909	-0.42302	0.94093
O	-4.73149	-0.93491	0.01889

Zero-point correction= 0.485730 (Hartree/Particle)

Thermal correction to Energy= 0.512475

Thermal correction to Enthalpy= 0.513420

Thermal correction to Gibbs Free Energy= 0.429277

sp-E = -1607.93587935 hartree

TS-Quino[5]helicene

N	3.71413	-1.29291	-0.83419
C	3.41556	-2.51557	-0.44268
C	2.36228	-2.76563	0.45099
H	2.23543	-3.74925	0.90779
C	1.50808	-1.72379	0.77237

C	1.6506	-0.4353	0.20572
C	2.91523	-0.26439	-0.46636
C	3.45909	1.04522	-0.64727
H	4.44851	1.12111	-1.0998
C	2.7736	2.11318	-0.16828
H	3.20364	3.11667	-0.20582
C	1.41187	1.98955	0.26921
C	0.74071	0.72275	0.34024
C	-0.73179	0.74488	0.33129
C	-1.66943	-0.39064	0.2099
C	-1.5038	-1.67695	0.7752
H	-0.74652	-1.82729	1.52478
C	-2.3575	-2.73913	0.51831
H	-2.16953	-3.70295	0.99722
C	-3.4721	-2.56898	-0.31763
H	-4.13073	-3.40853	-0.55055
C	-3.76651	-1.29399	-0.76105
H	-4.68773	-1.1	-1.31629
C	-2.92984	-0.19306	-0.45714
C	-3.42056	1.12889	-0.68756
H	-4.39973	1.25754	-1.15437
C	-2.70843	2.18945	-0.22957
H	-3.1053	3.20456	-0.30115
C	-1.36122	2.03149	0.2366
C	-0.63954	3.23226	0.45957
H	-1.18926	4.17511	0.50404
C	0.72211	3.21128	0.47956
H	1.29911	4.13664	0.54108
H	4.06797	-3.32639	-0.7887
H	0.78477	-1.89592	1.55249

Zero-point correction= 0.276494 (Hartree/Particle)

Thermal correction to Energy= 0.290319

Thermal correction to Enthalpy= 0.291263

Thermal correction to Gibbs Free Energy= 0.236219

sp-E = -862.785785701 hartree

1.7. Barriers to enantiomerization of experiments

Supplementary Table 4. Measured data of rotation barrier and half-life of **5a**

P/M	（1+(P/M)）/(1-(P/M))	t	ln
0.020408163	1.041666667	0	0.040821995
0.117318436	1.265822785	3600	0.235722334
0.239157373	1.628664495	7200	0.487760351
0.377410468	2.212389381	10800	0.794073099
0.470588235	2.777777778	14400	1.021651248
0.538461538	3.333333333	18000	1.203972804

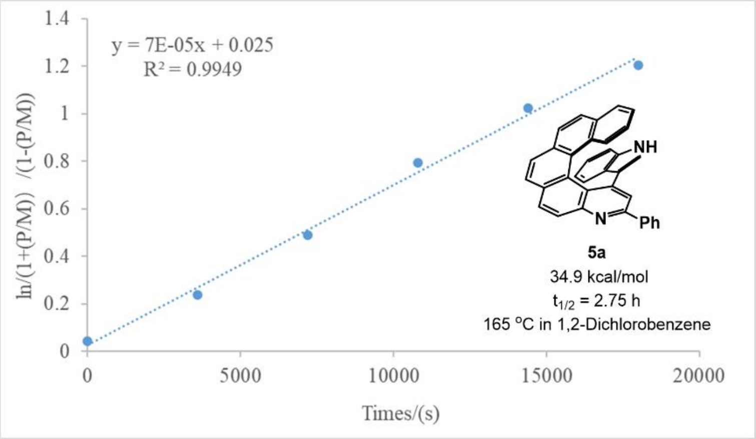

Supplementary Fig. 7. rotating barrier and half-life of **5a**

Supplementary Table 5. Measured data of rotation barrier and half-life of **5aj**

P/M	（1+(P/M)）/(1-(P/M))	t	ln
0.071811361	1.154734411	0	0.14387037
0.150747986	1.35501355	3600	0.303811454
0.239157373	1.628664495	7200	0.487760351
0.333333333	2.000000000	10800	0.693147181
0.400560224	2.336448598	14400	0.848632083
0.477810651	2.830028329	18000	1.040286722

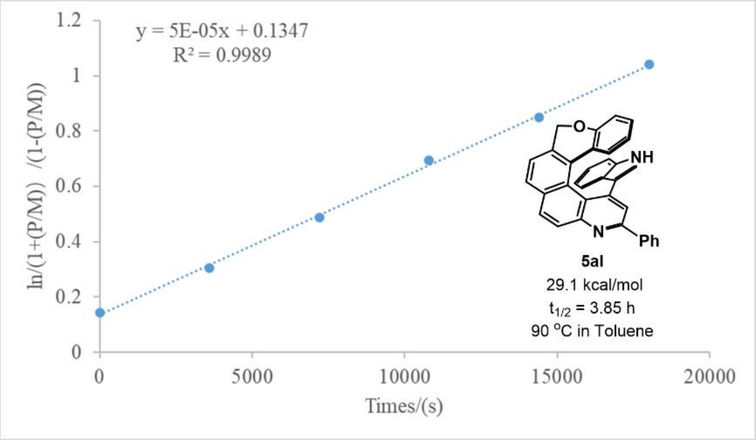

Supplementary Fig. 8. rotating barrier and half-life of **5aj**

1.8. Electrochemical and Photophysical properties

1.8.1. Electrochemical properties of 5a, 5aj and 5am

Normalized Cyclic voltammograms patterns obtained on glassy carbon electrode at a 0.2 V/s scan rate for **5a**, **5aj** and **5am** with 5 × 10^{-4} M in CH_3CN + 0.1 M tetrabutylammonium hexafluorophosphate. Both compounds gave rise to two reduction waves and three (for **5a** and **5aj**) or two (for **5am**) oxidation waves.

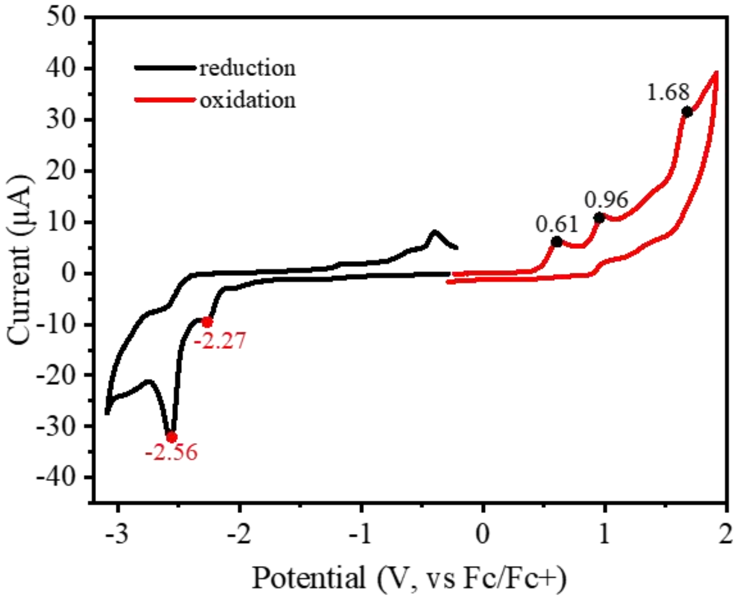

Supplementary Fig. 9. Cyclic voltammogram of **5a**

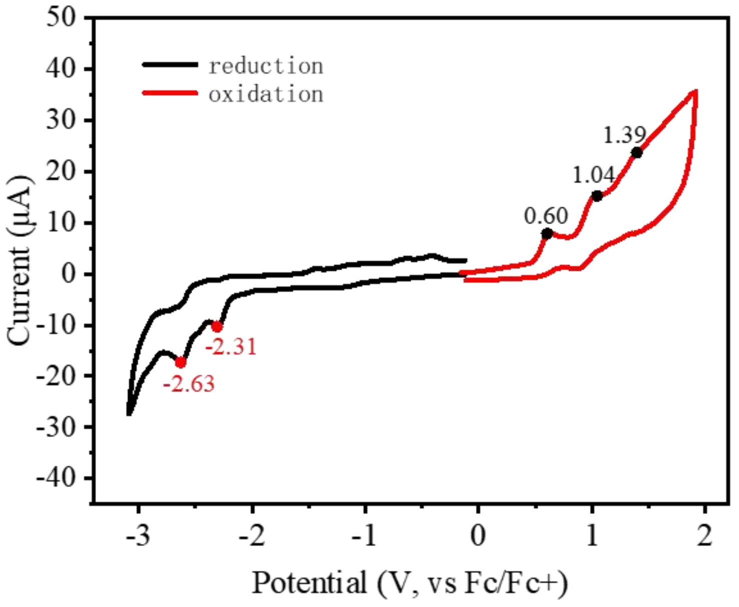

Supplementary Fig. 10. Cyclic voltammogram of **5aj**

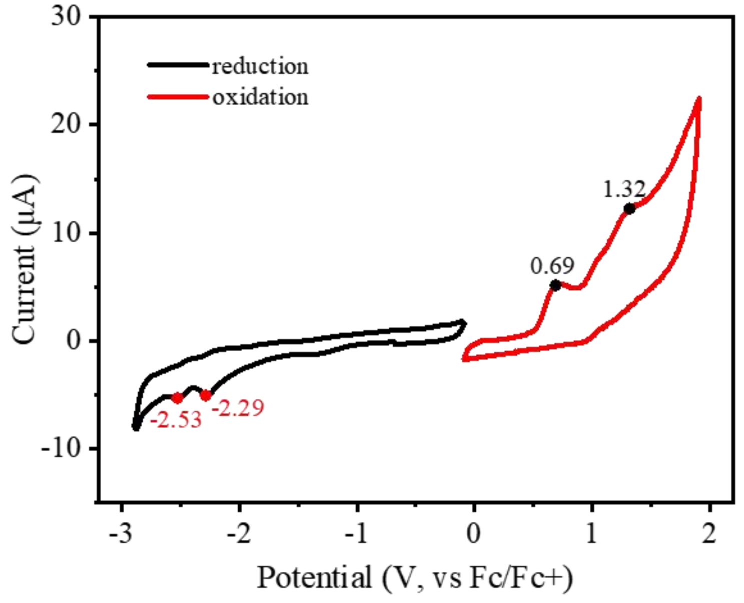

Supplementary Fig. 11. Cyclic voltammogram of **5am**

1.8.2. Optical study absorption and emission neutral form of selected products

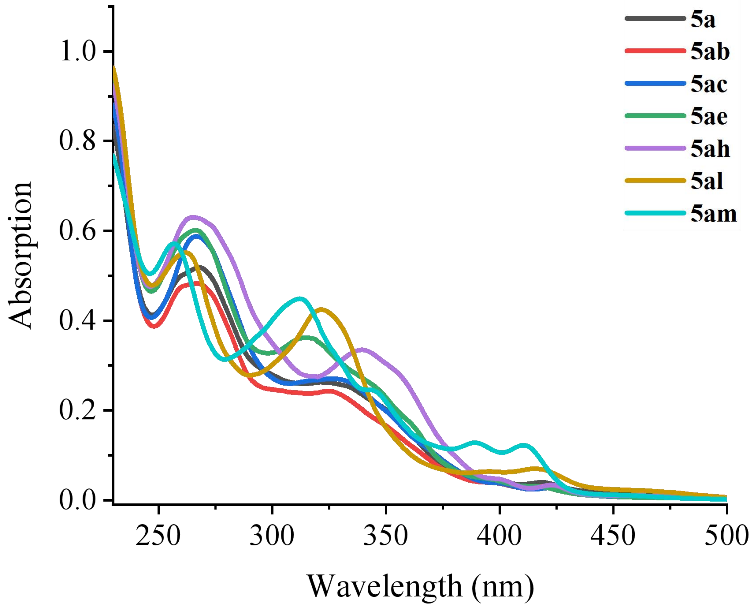

Supplementary Fig. 12. Absorption spectra for selected products measured at 1×10^{-5} M in DCM

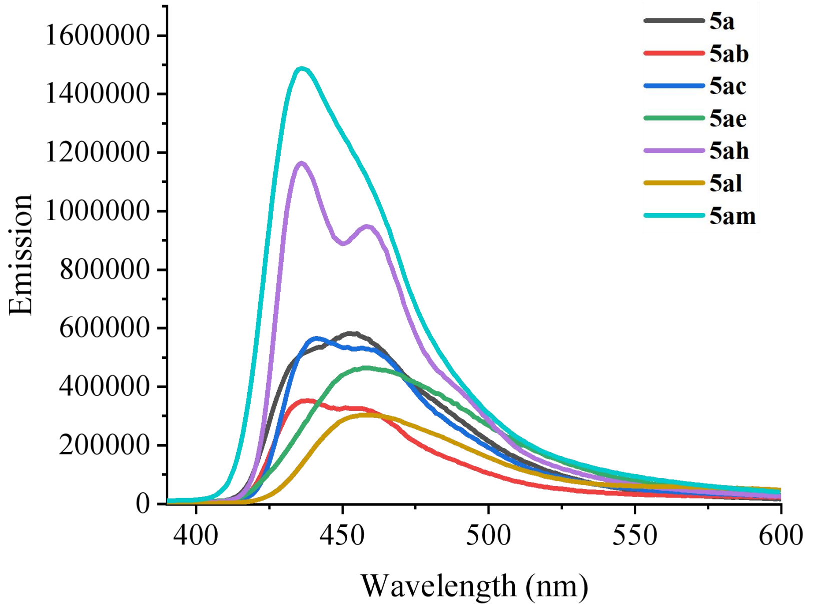

Supplementary Fig. 13. Emission spectra for selected products measured at 1×10^{-5} M in DCM

1.8.3. Optical study absorption and emission about solvent effect and acid effect of 5a

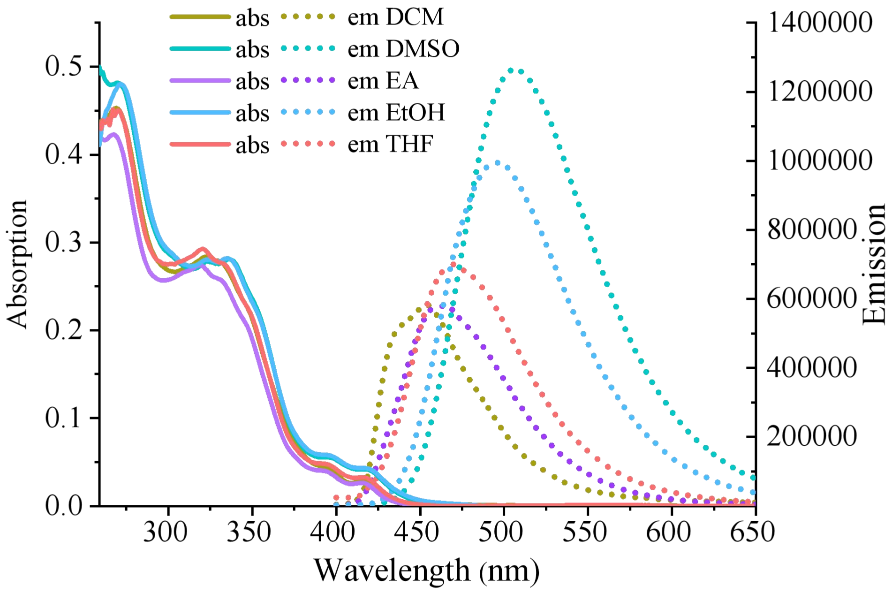

Supplementary Fig. 14. Absorbance and Emission spectra for solvent effect of **5a** measured at 1×10^{-5} M

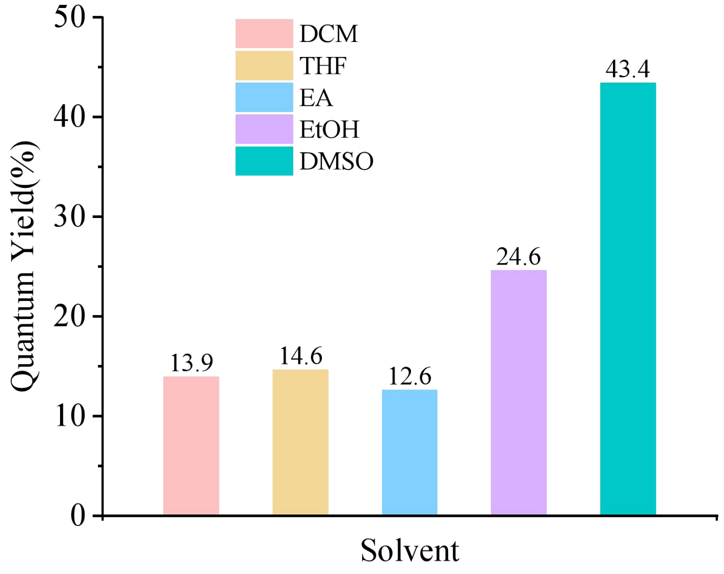

Supplementary Fig. 15. Quantum yield for solvent effect of **5a** measured under the absorbance lower than 0.1.

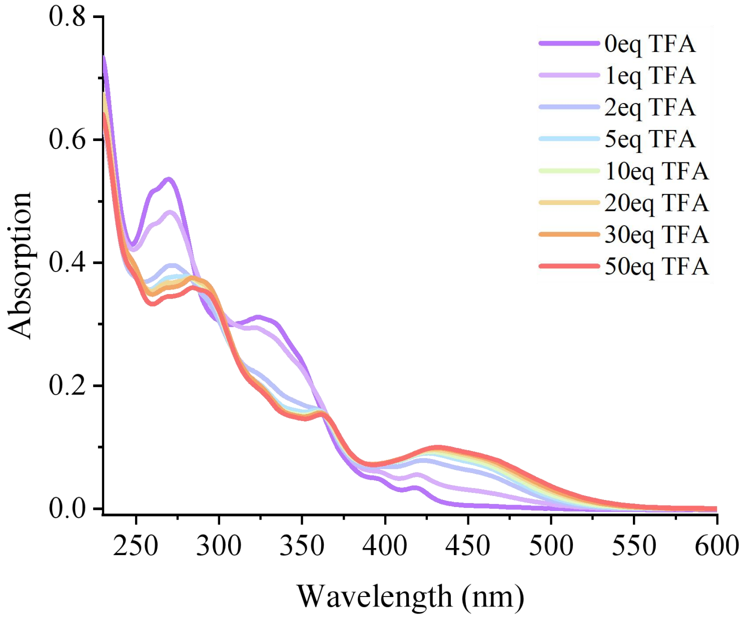

Supplementary Fig. 16. Absorbance for acid effect of **5a** measured at 1×10^{-5} M

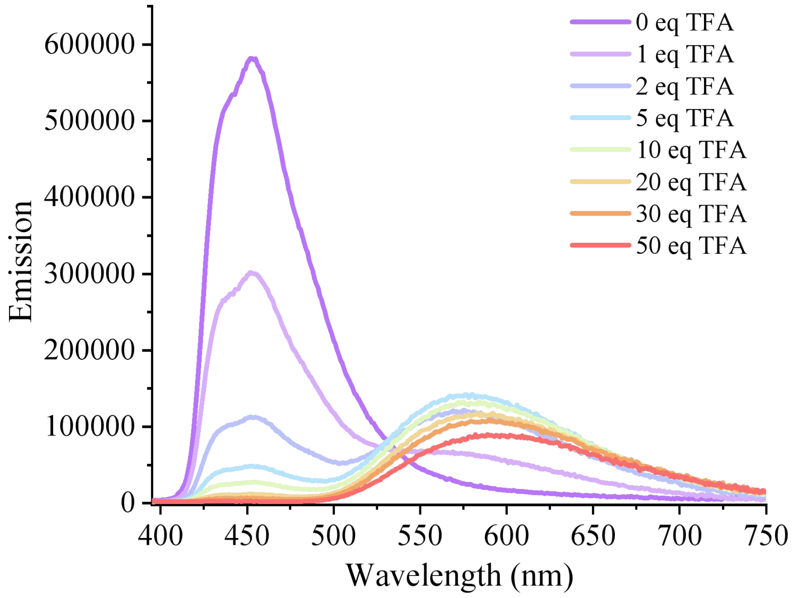

Supplementary Fig. 17. Emission spectra for acid effect of **5a** measured at 1×10^{-5} M

1.8.4. Circular dichroism (CD) and circularly polarized uminescence (CPL) study

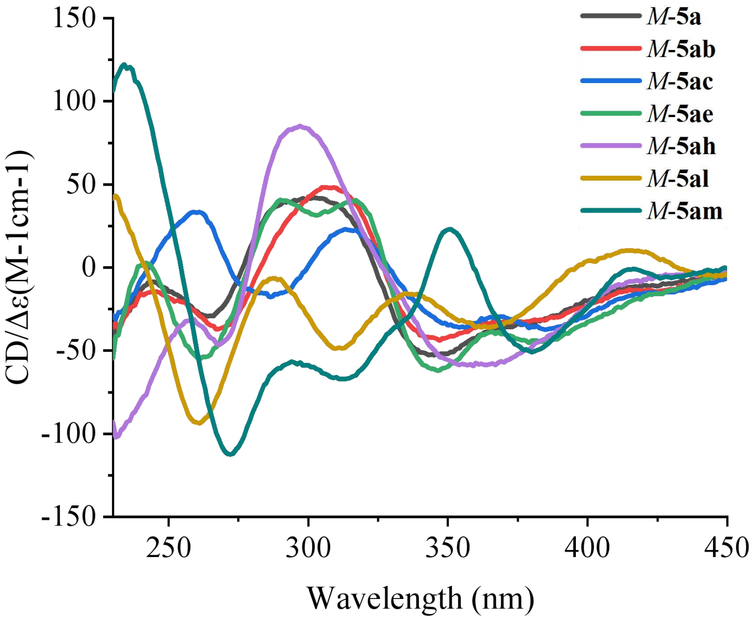

Supplementary Fig. 18. Circular dichroism (CD) spectra of selected products measured at 1×10^{-5} M in DCM

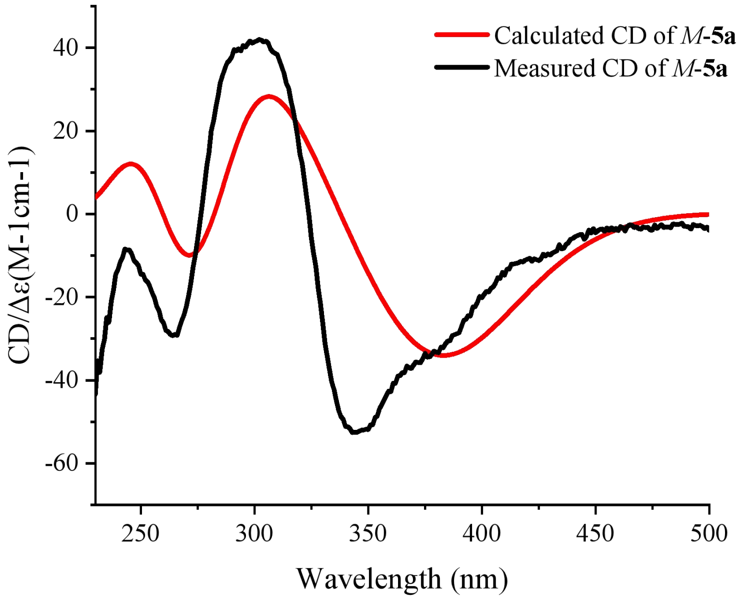

Supplementary Fig. 19. Circular dichroism (CD) spectra of *M*-**5a** measured at 1×10^{-5} M in DCM and calculated at the at the SMD(DCM)/M06-2X-D3/def2-TZVPP//B3LYP D3(BJ)/def -2-SVP level of theory.

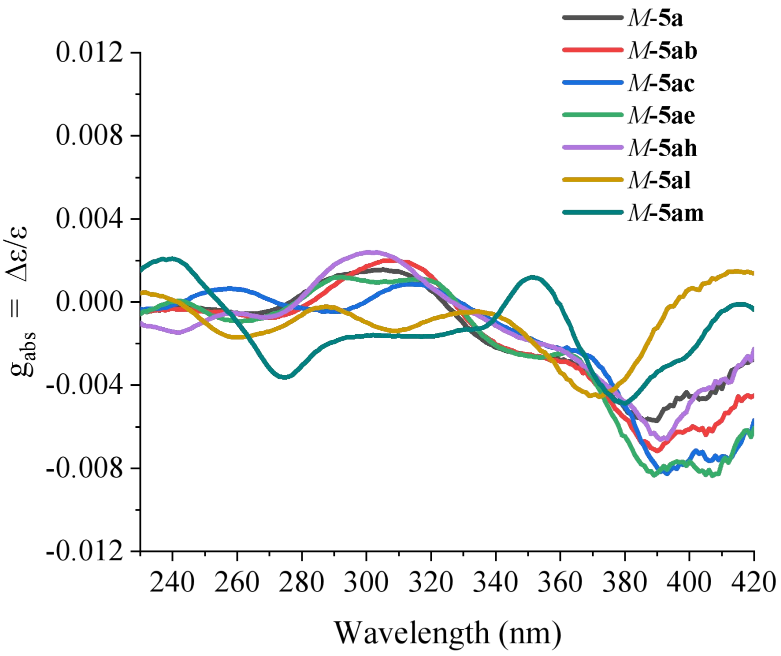

Supplementary Fig. 20. g_{abs} spectra of selected products

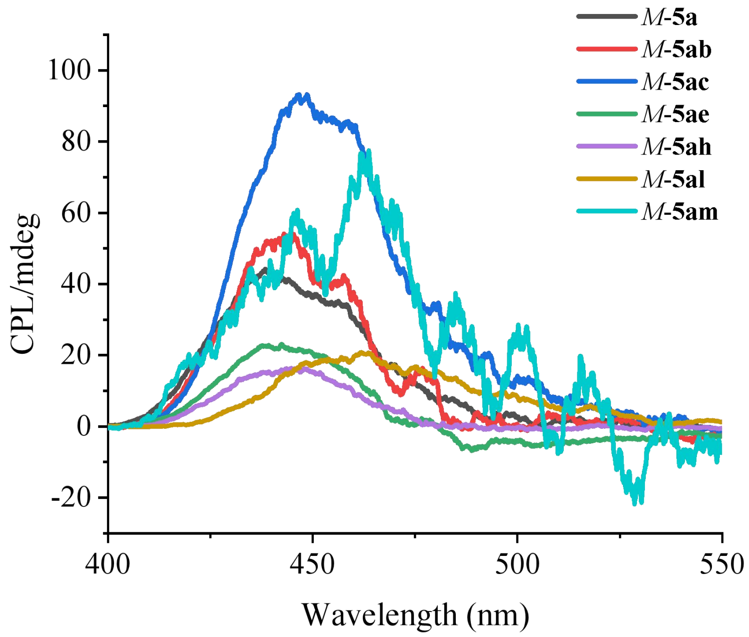

Supplementary Fig. 21. Circularly Polarized Luminescence (CPL) of selected products measured at 1×10^{-5} M in DCM

1.9. NMR spectra

^1H NMR (400 MHz, $CDCl_3$) of **1a**

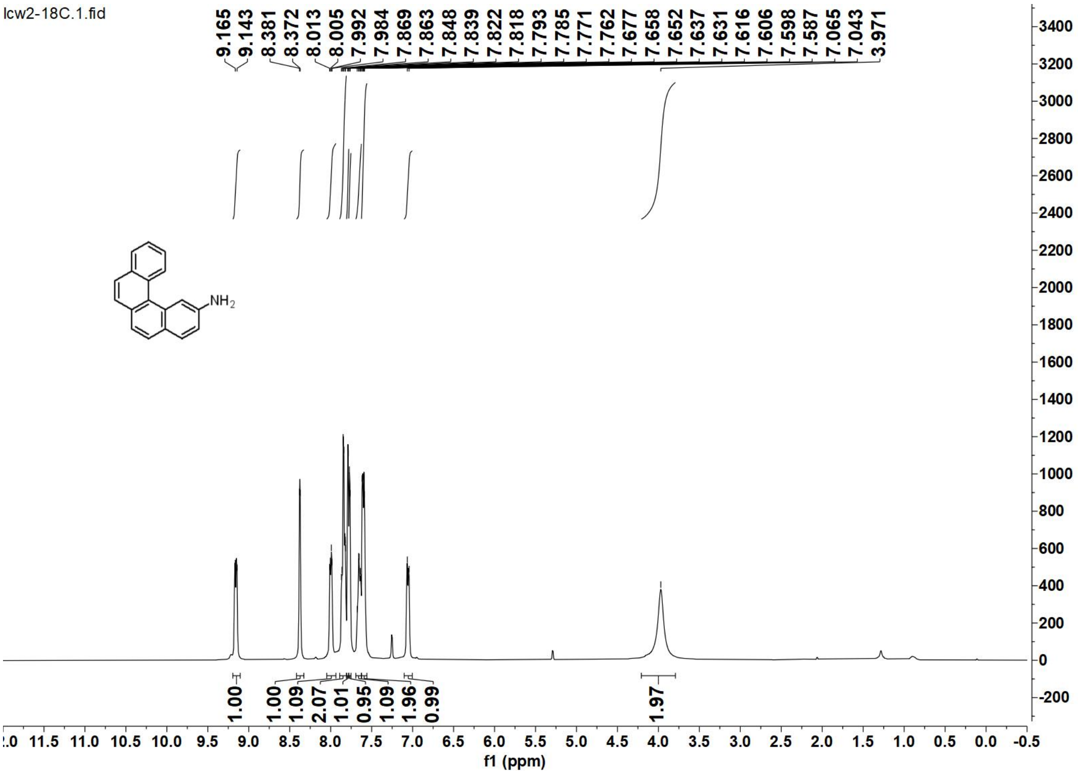

^{13}C NMR (101 MHz, $CDCl_3$) of **1a**

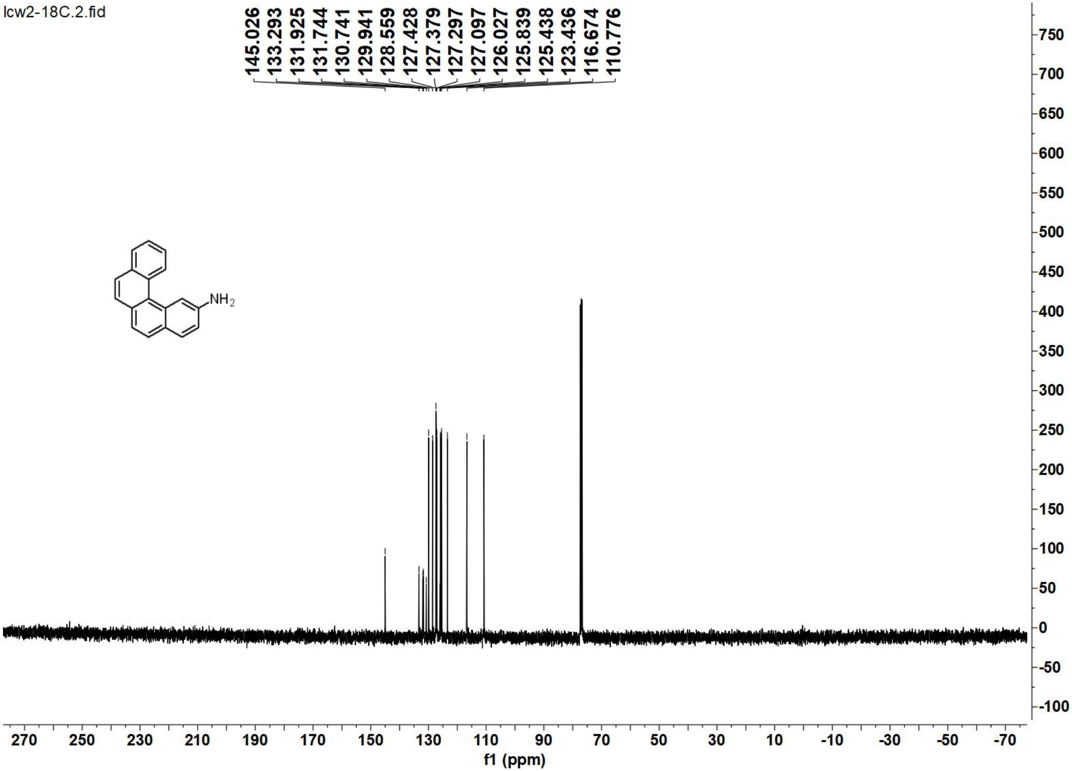

^{1}H NMR (400 MHz, $CDCl_3$) of **1b**

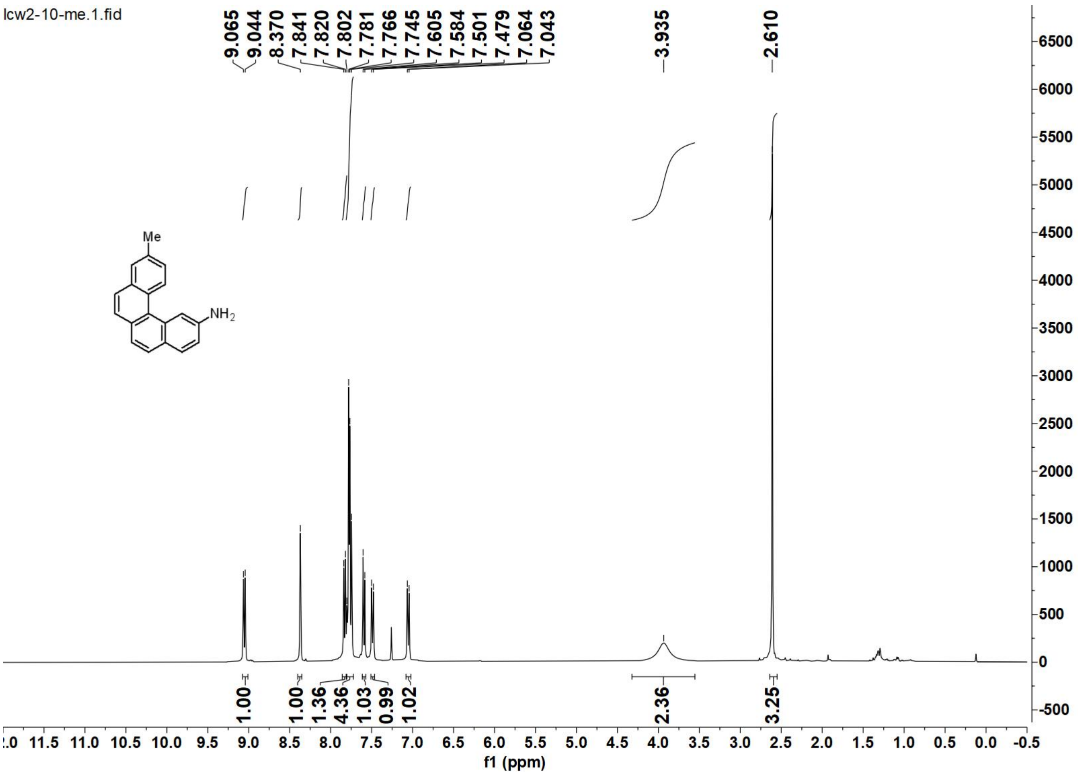

^{13}C NMR (101 MHz, $CDCl_3$) of **1b**

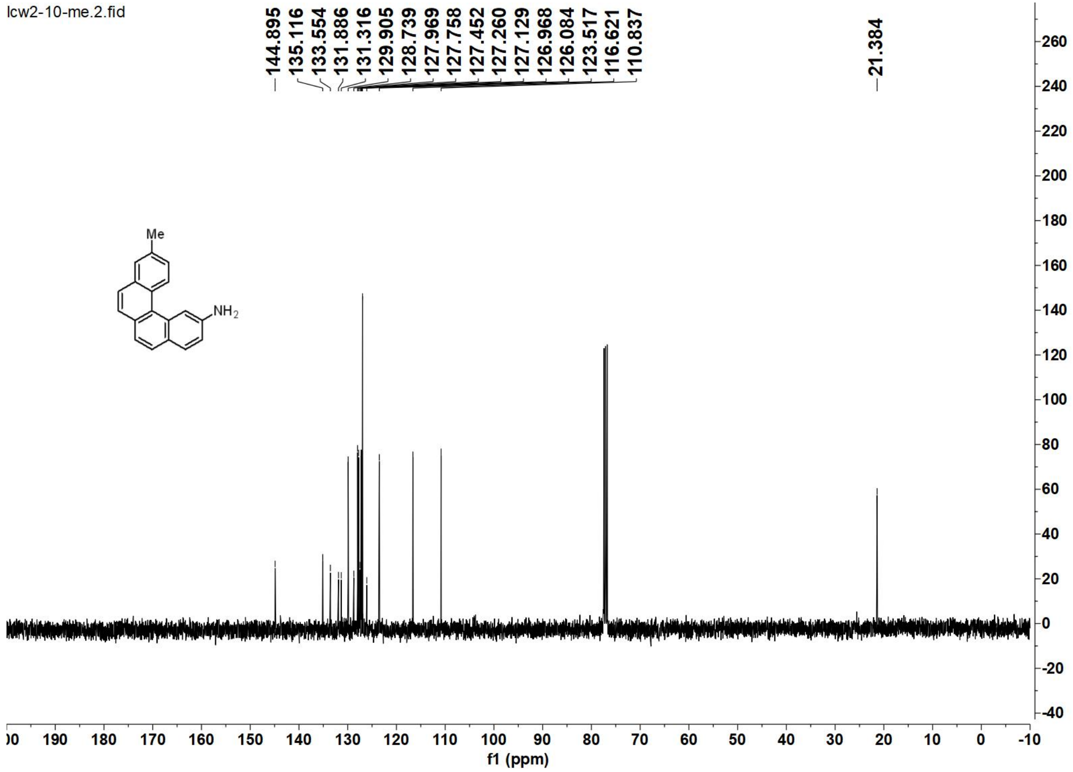

^{1}H NMR (400 MHz, $CDCl_3$) of **1c**

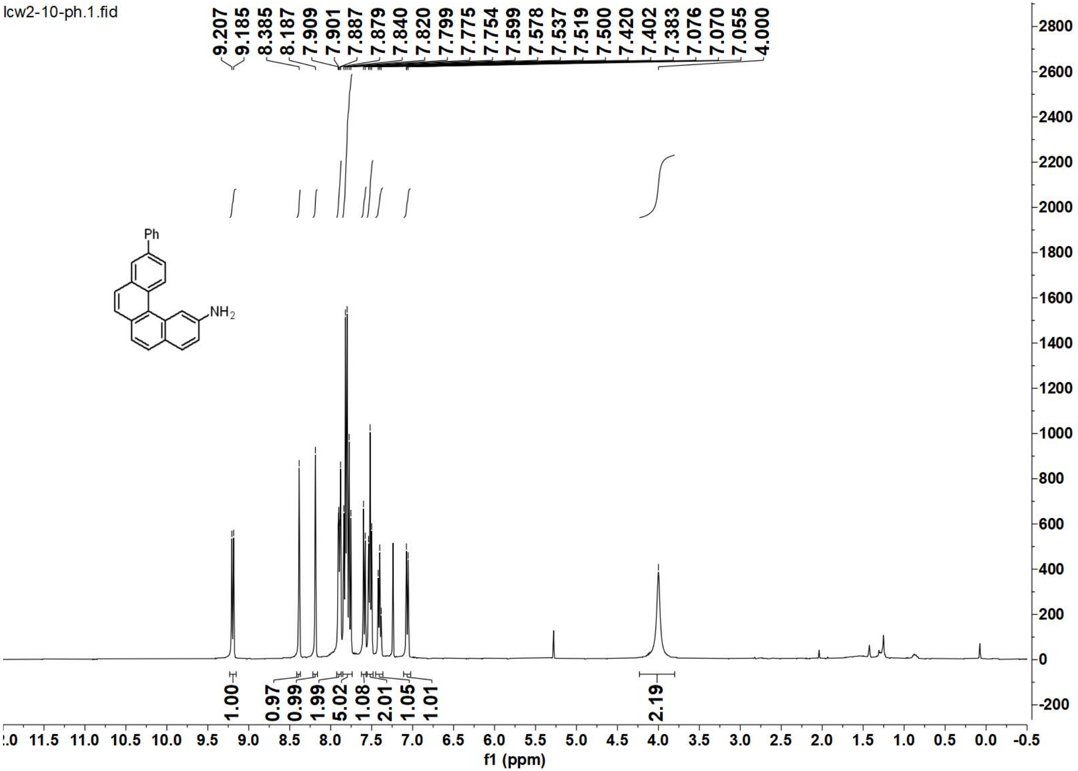

^{13}C NMR (101 MHz, $CDCl_3$) of **1c**

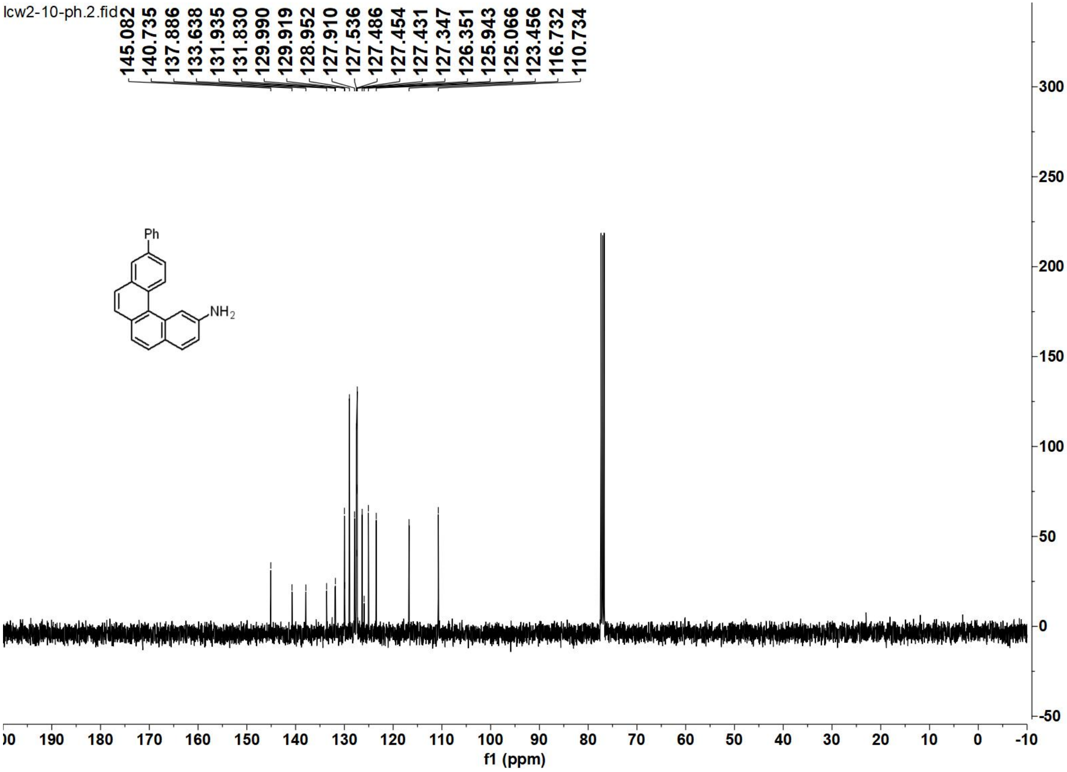

^{1}H NMR (400 MHz, $CDCl_3$) of **1d**

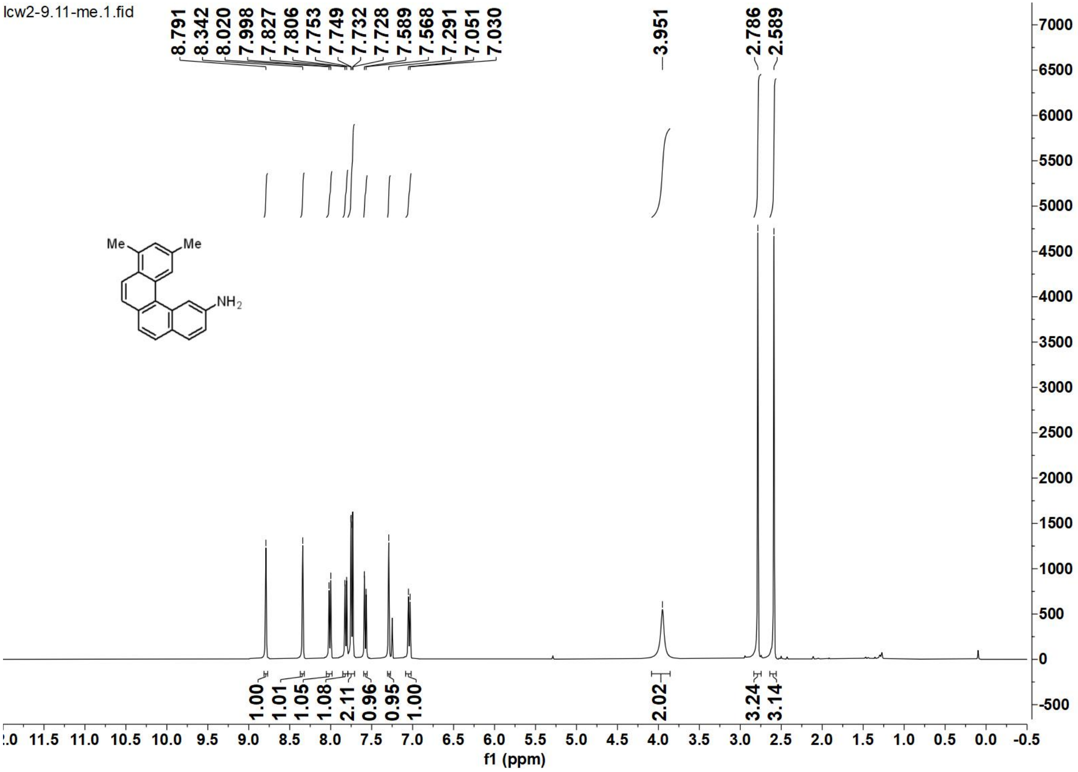

^{13}C NMR (101 MHz, $CDCl_3$) of **1d**

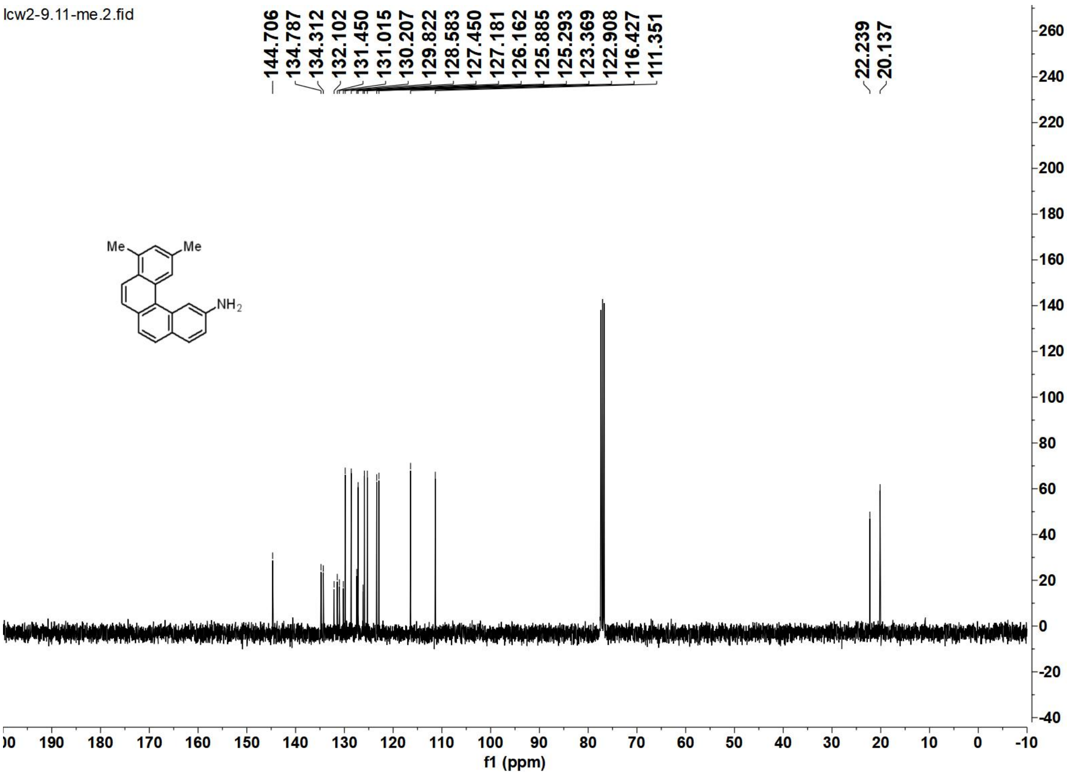

^1H NMR (400 MHz, $CDCl_3$) of **1e**

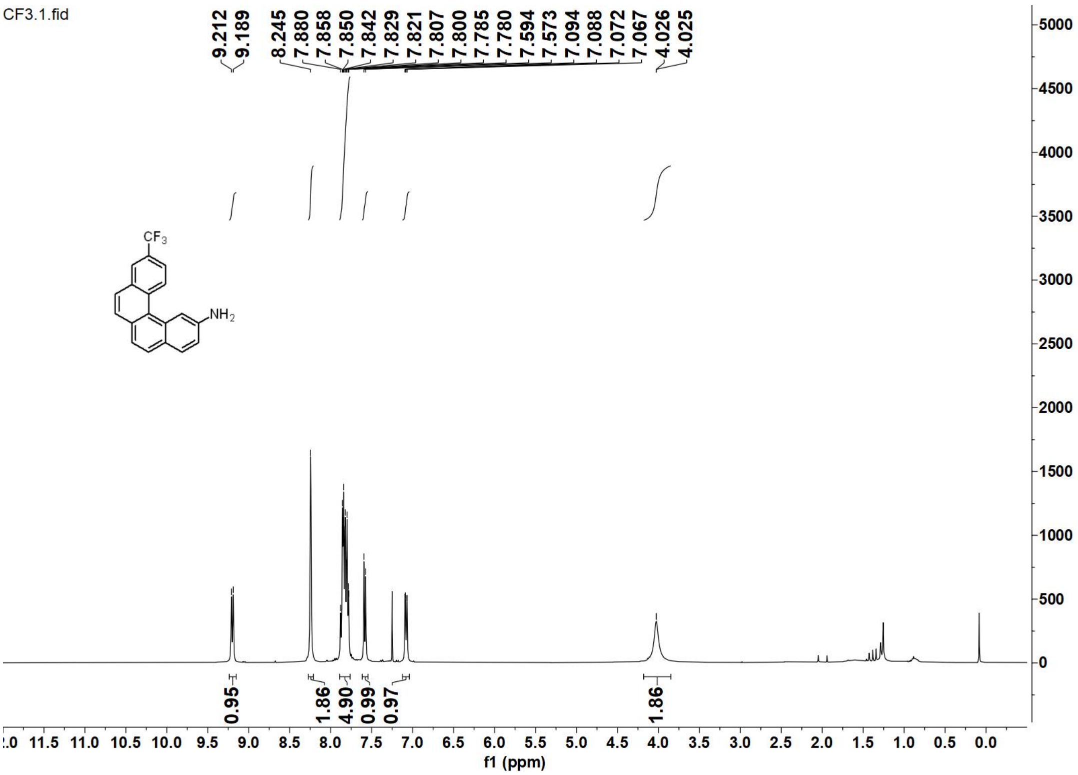

^{13}C NMR (101 MHz, $CDCl_3$) of **1e**

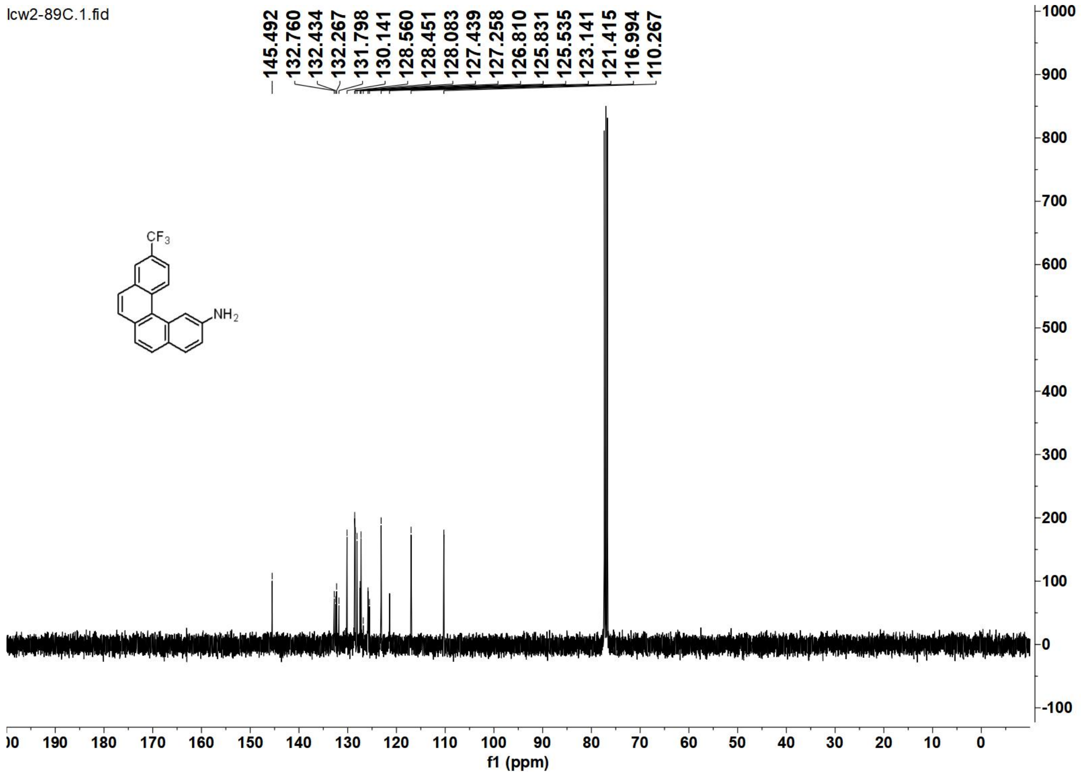

^{19}F NMR (376 MHz, $CDCl_3$) of **1e**

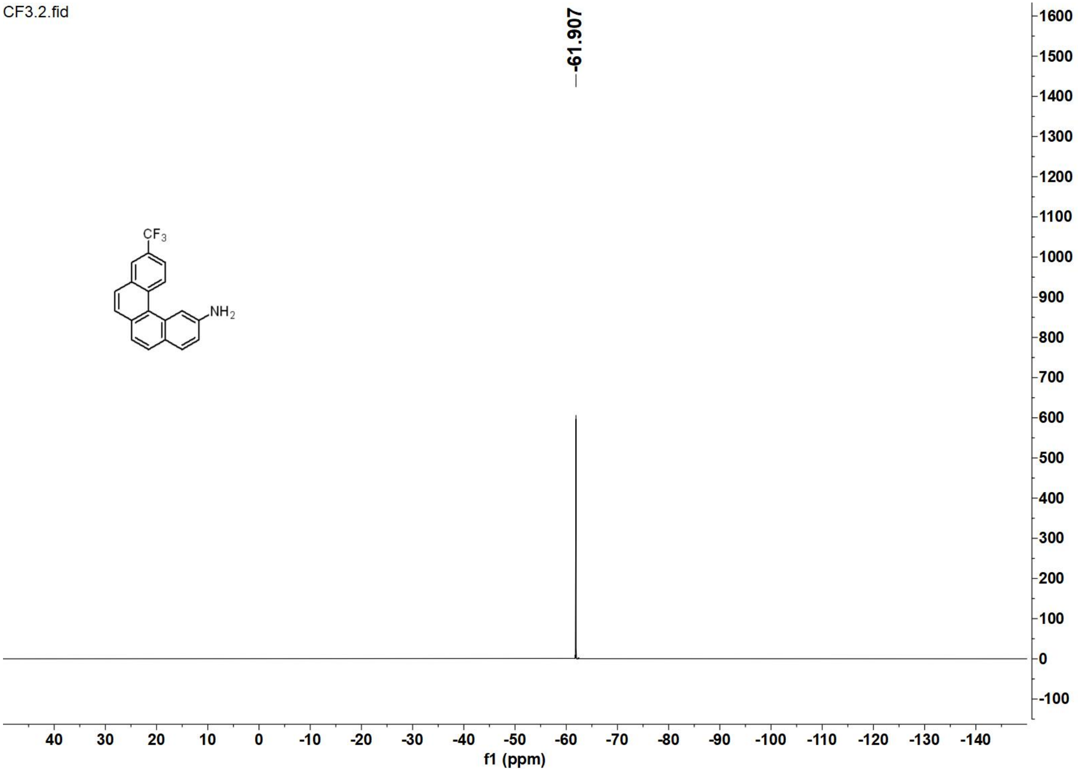

^1H NMR (400 MHz, $CDCl_3$) of **1f**

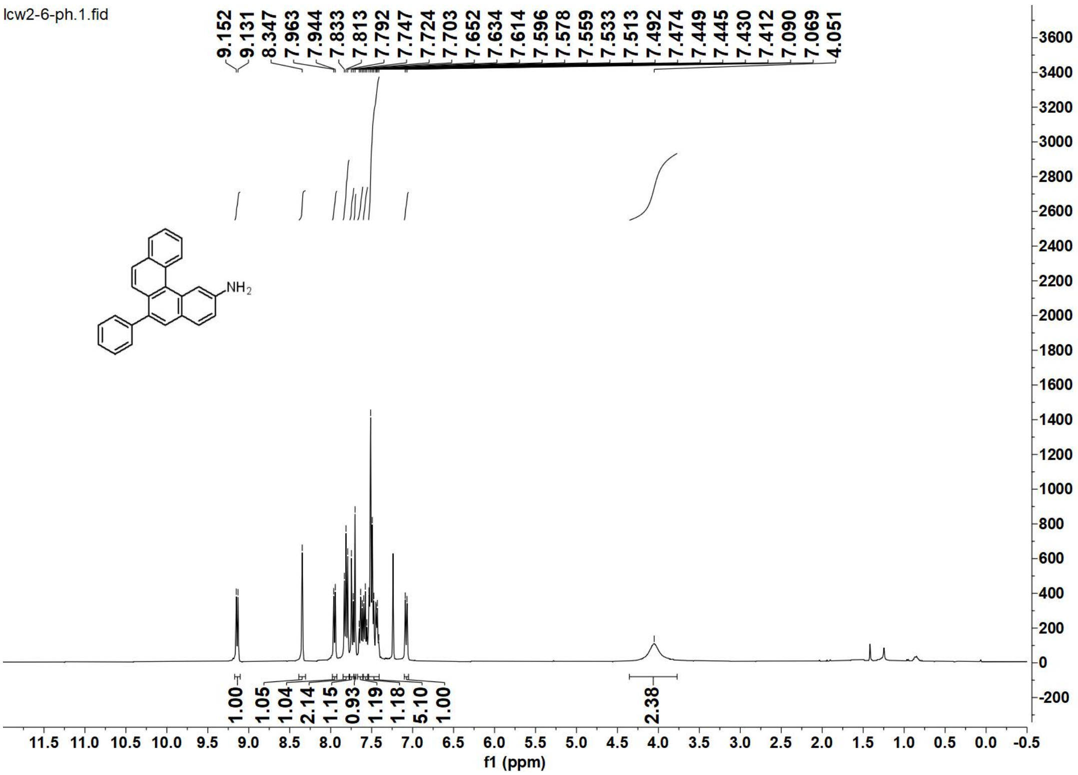

^{13}C NMR (101 MHz, $CDCl_3$) of **1f**

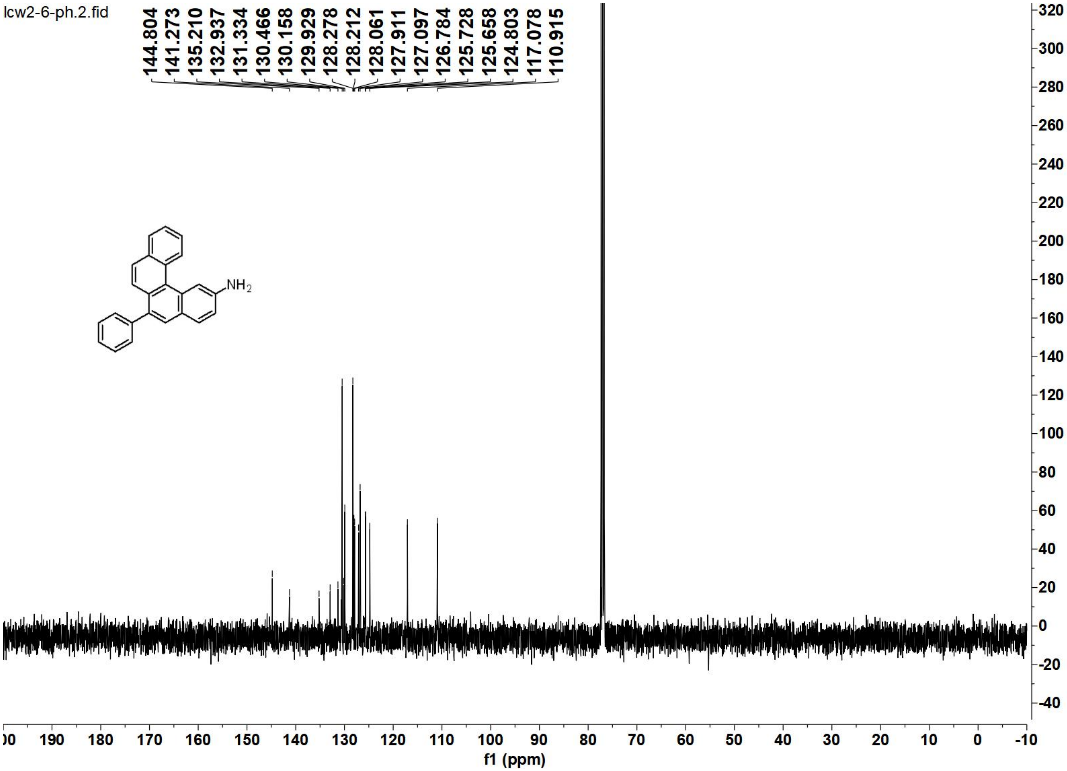

^1H NMR (400 MHz, $CDCl_3$) of **1g**

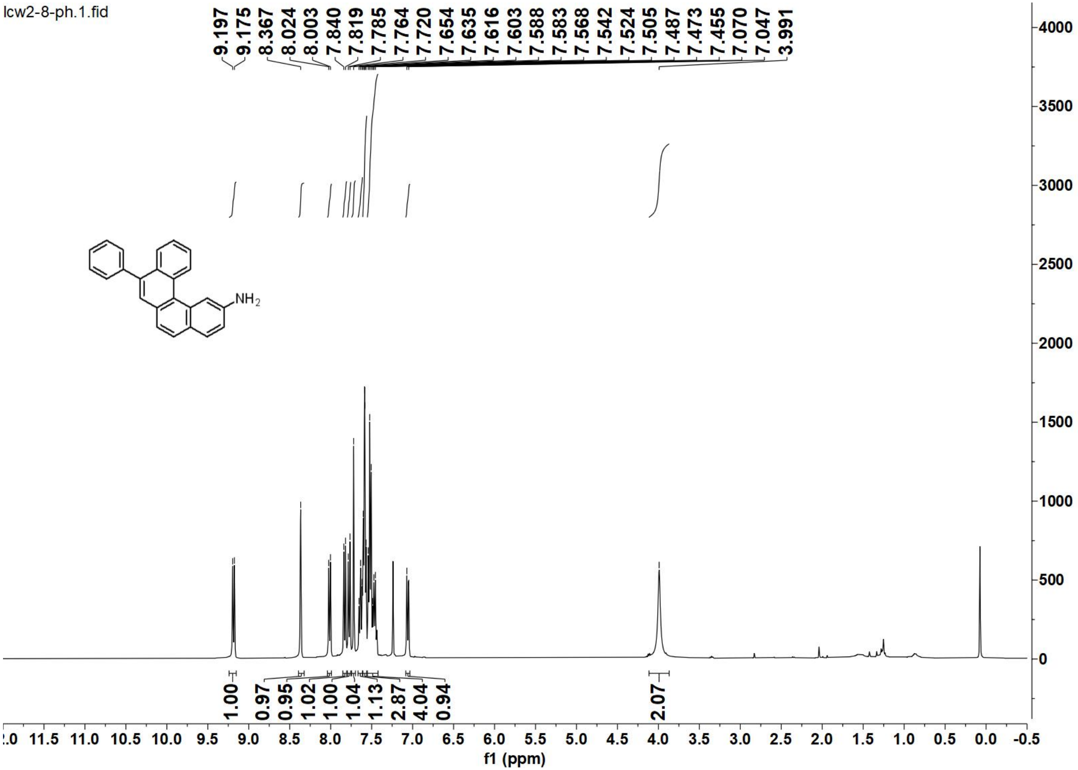

^{13}C NMR (101 MHz, $CDCl_3$) of **1g**

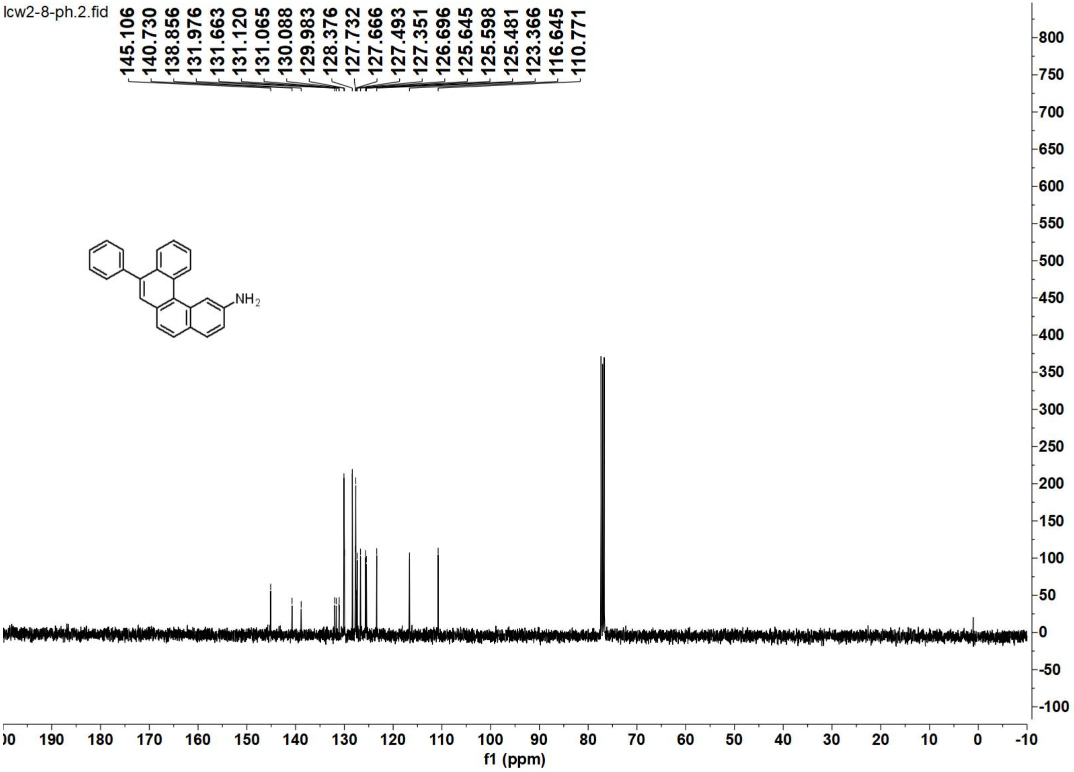

^{1}H NMR (400 MHz, $CDCl_3$) of **1h**

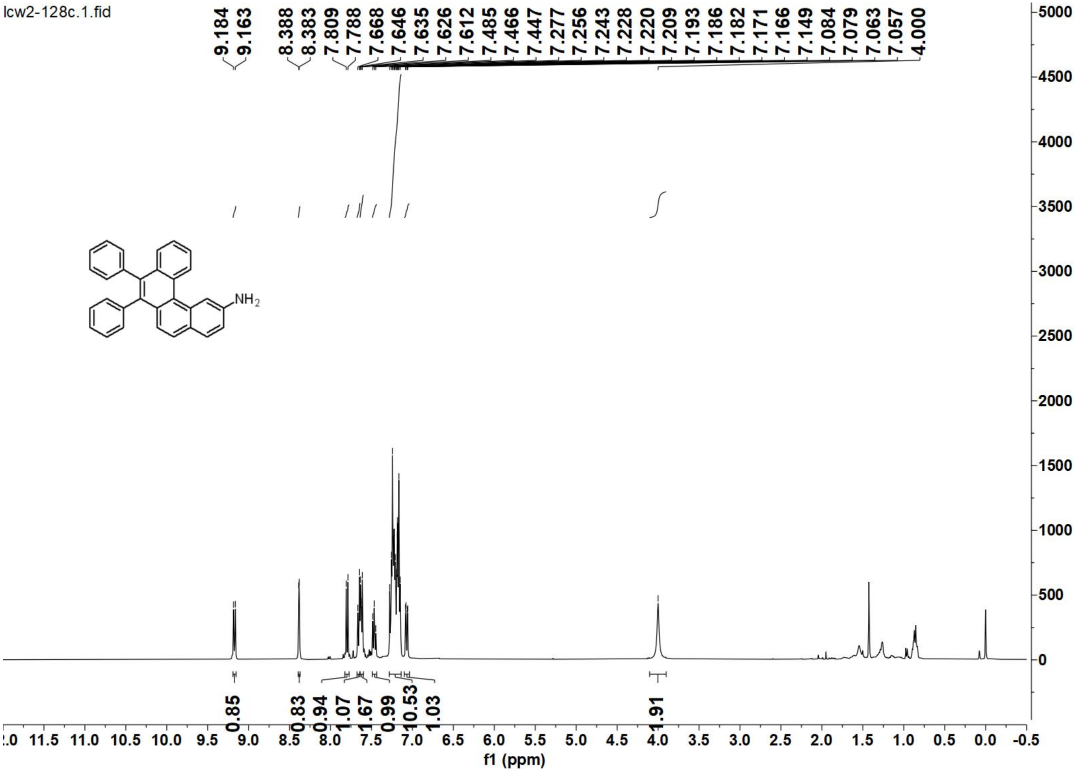

^{13}C NMR (101 MHz, $CDCl_3$) of **1h**

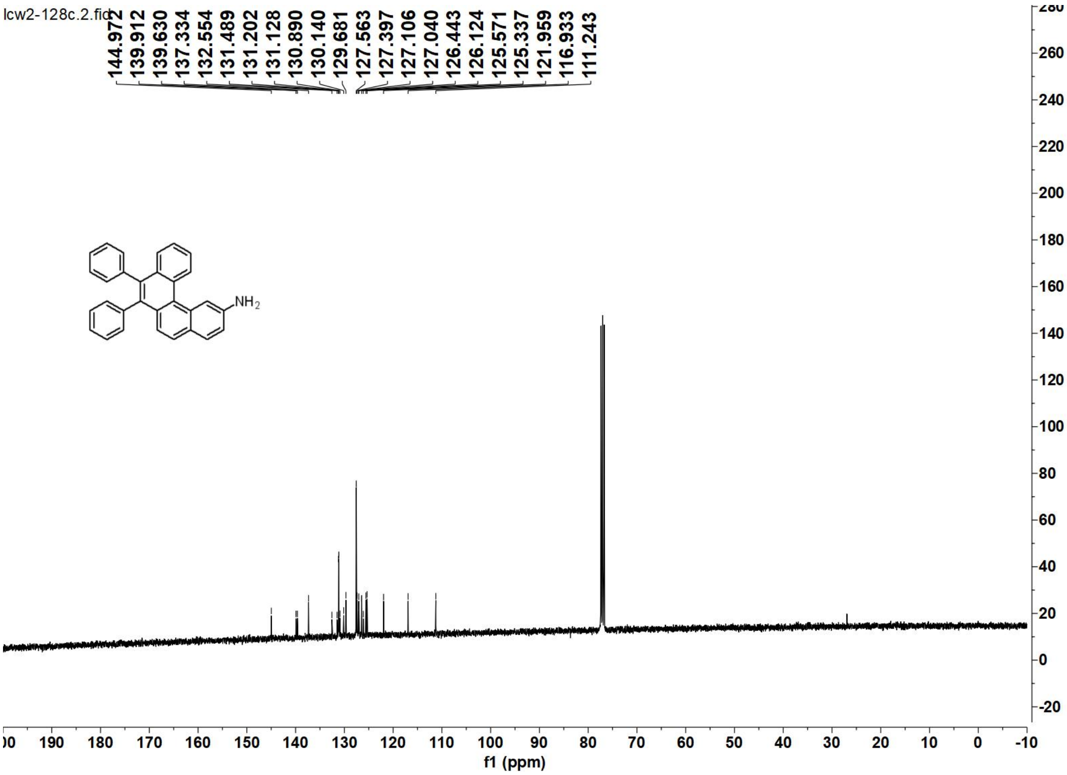

^{1}H NMR (400 MHz, DMSO-d_6) of **1i**

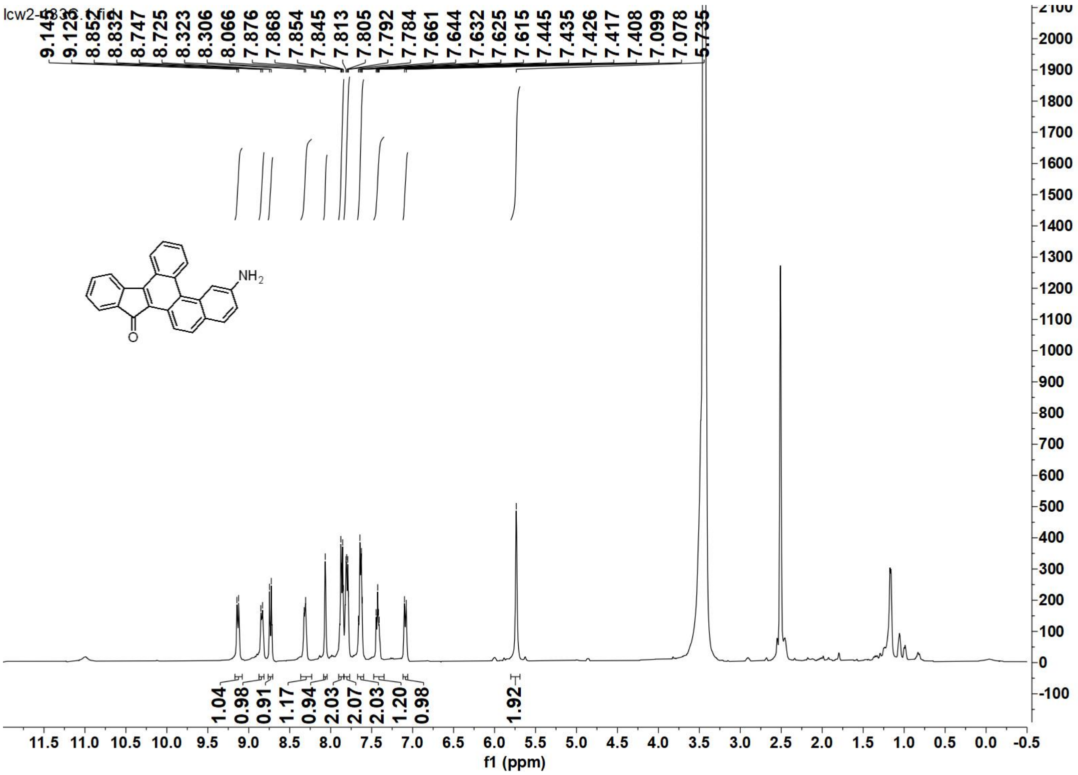

^{13}C NMR (101 MHz, DMSO-d_6) of **1i**

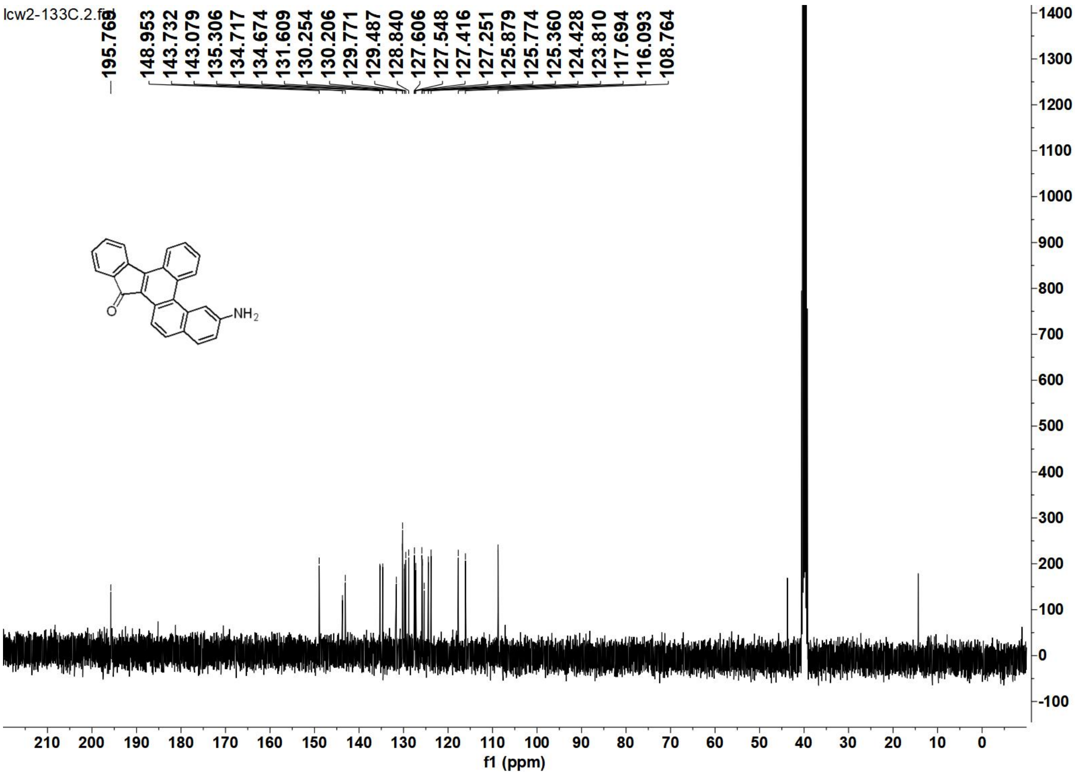

^{1}H NMR (400 MHz, $CDCl_3$) of **1j**

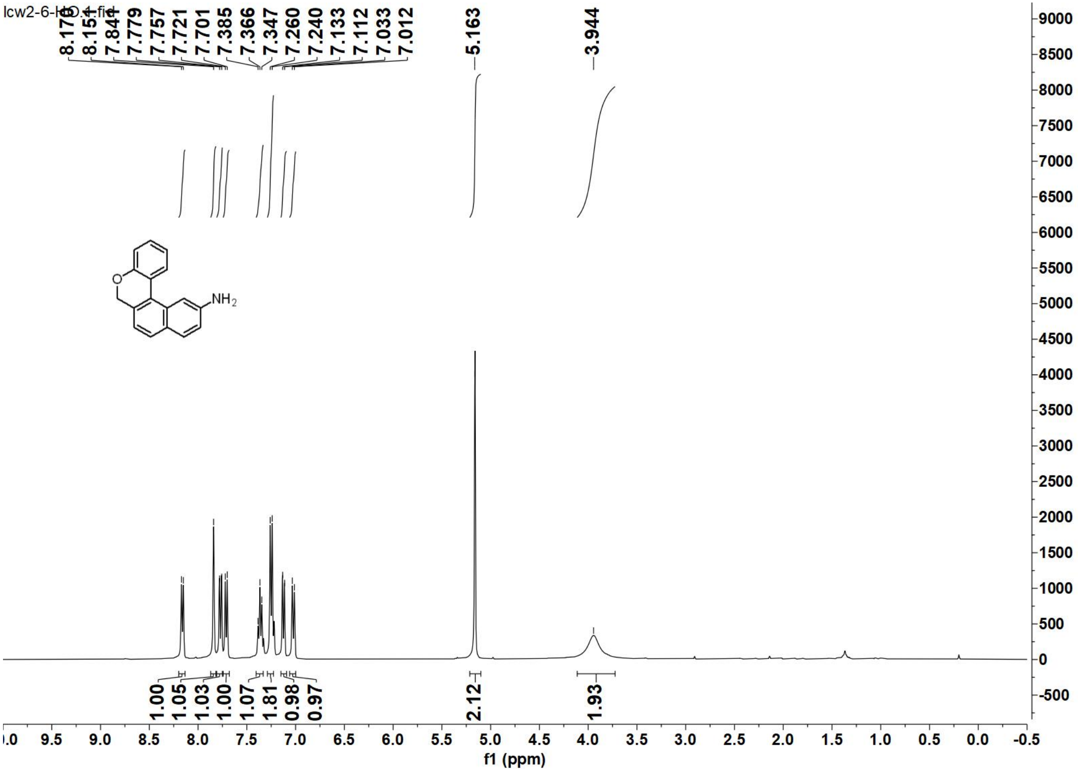

^{13}C NMR (101 MHz, $CDCl_3$) of **1j**

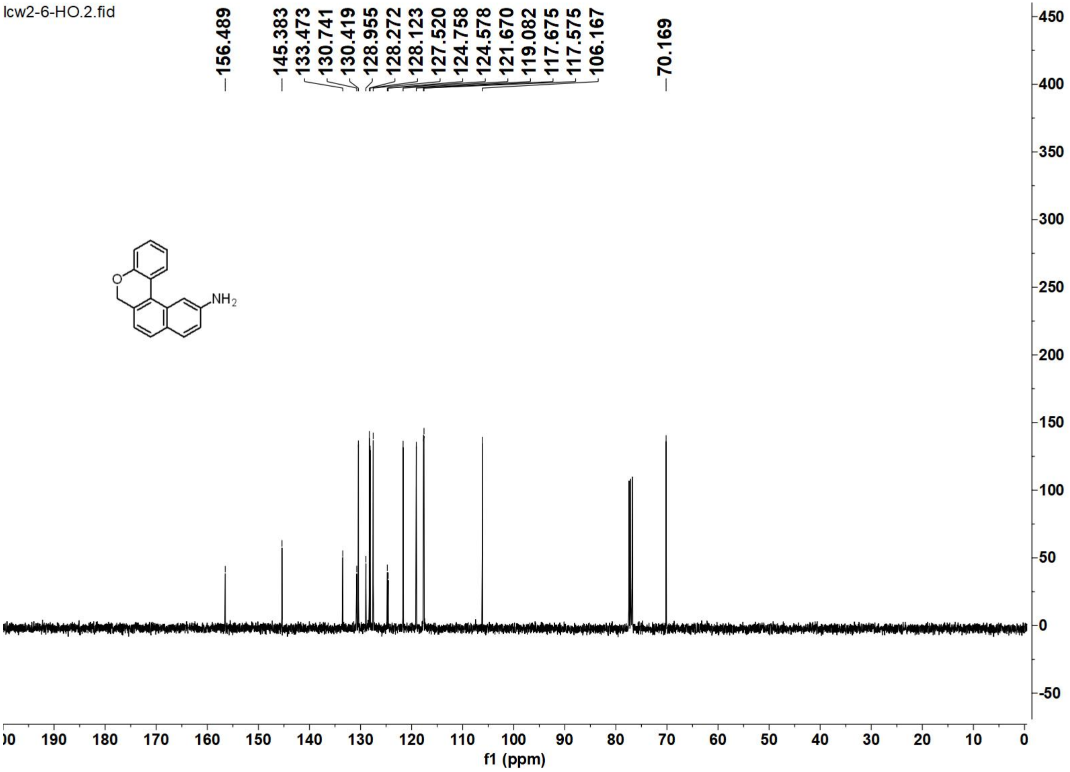

^{1}H NMR (400 MHz, $CDCl_3$) of **1k**

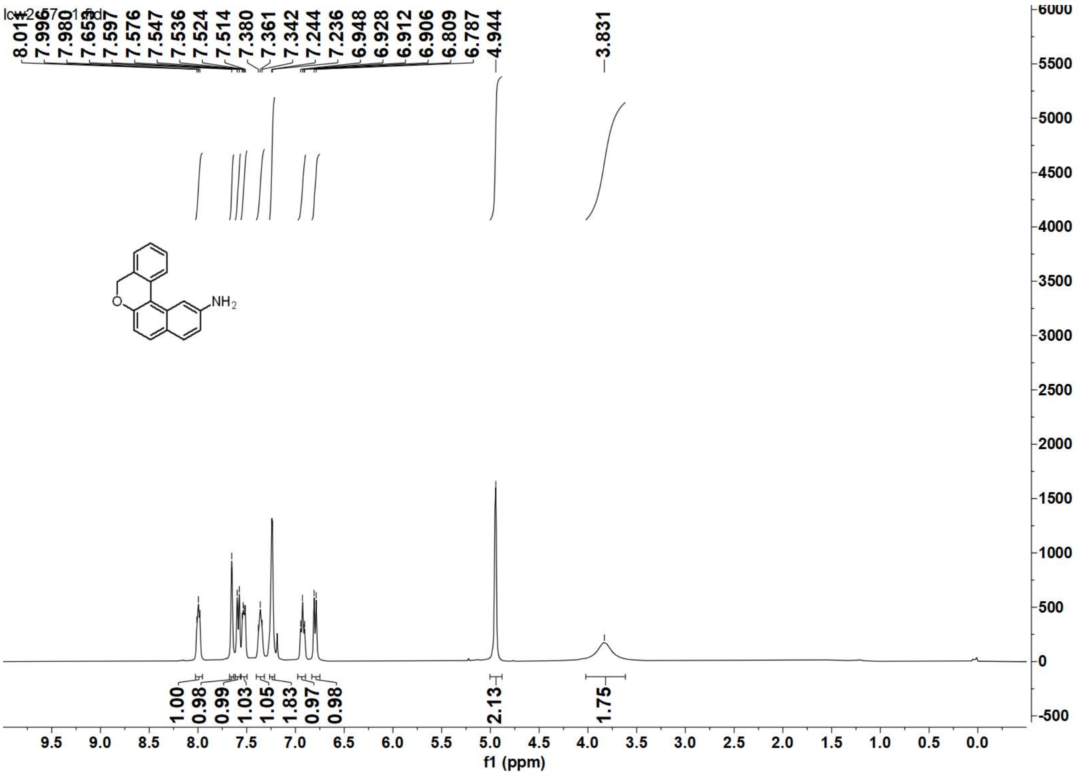

^{13}C NMR (101 MHz, $CDCl_3$) of **1k**

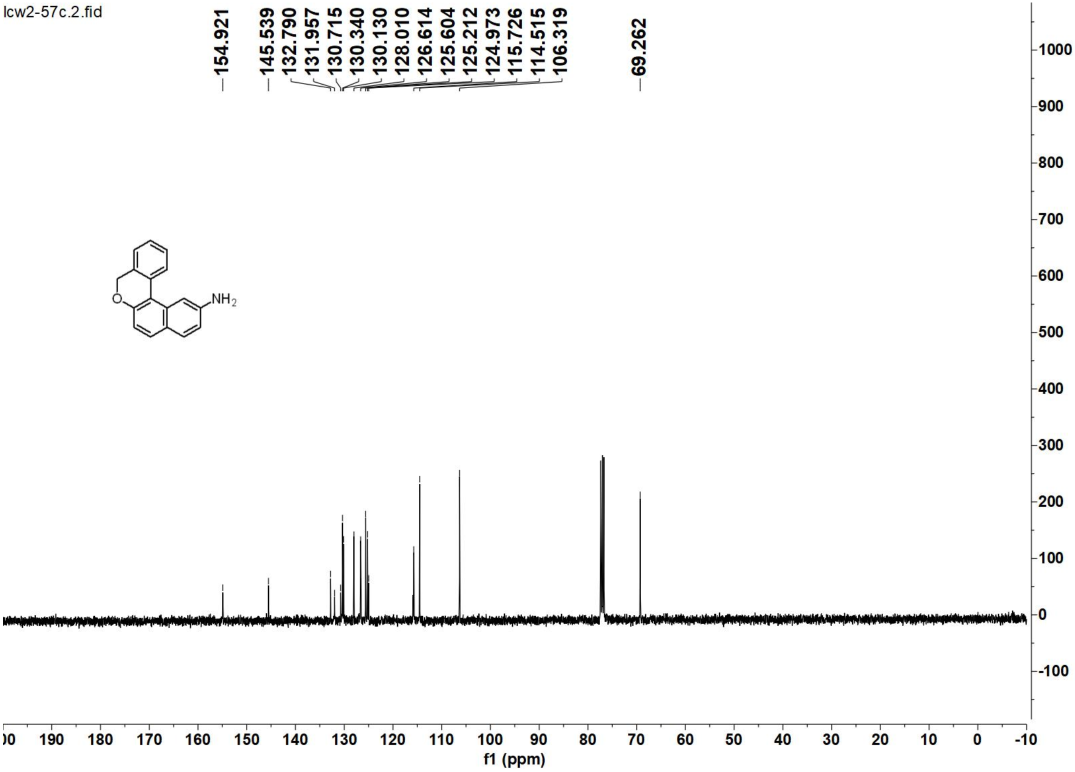

^1H NMR (400 MHz, $CDCl_3$) of **1l**

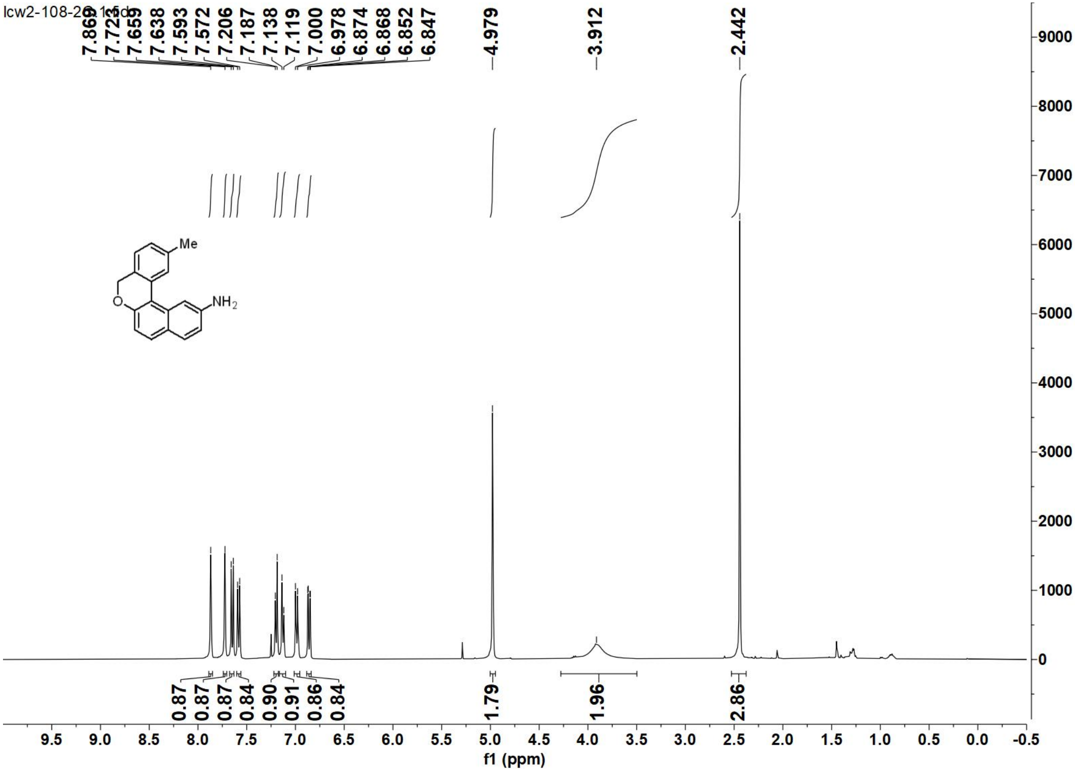

^{13}C NMR (101 MHz, $CDCl_3$) of **1l**

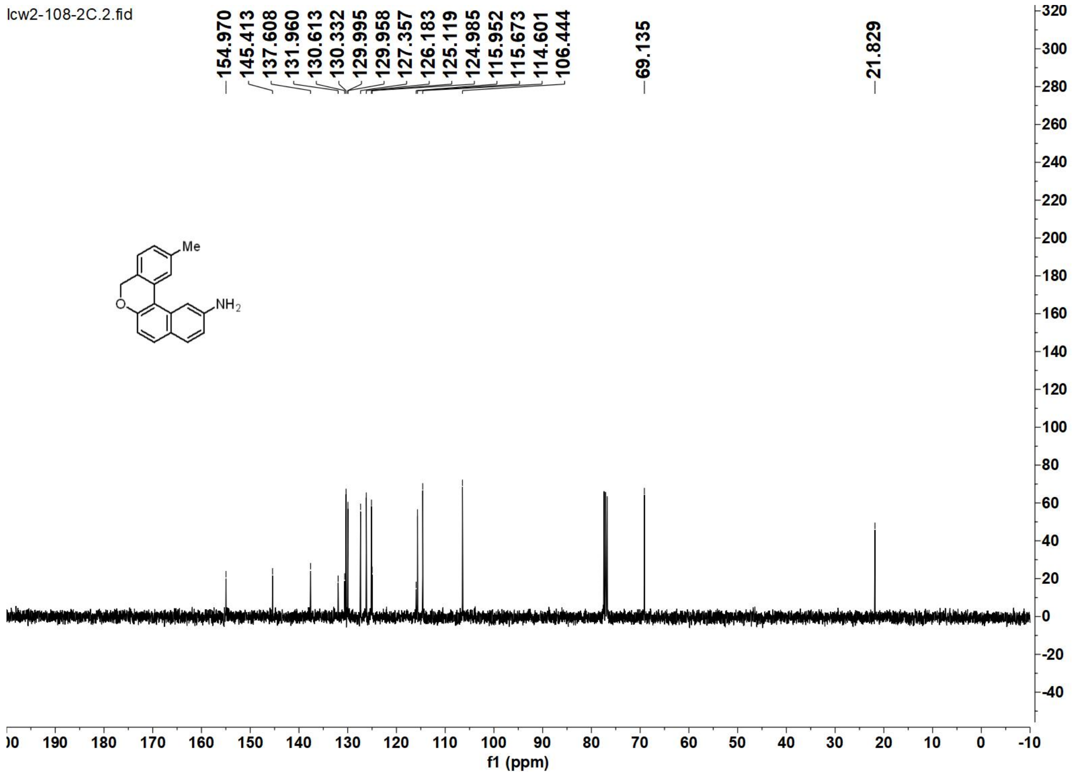

^{1}H NMR (400 MHz, $CDCl_3$) of **1m**

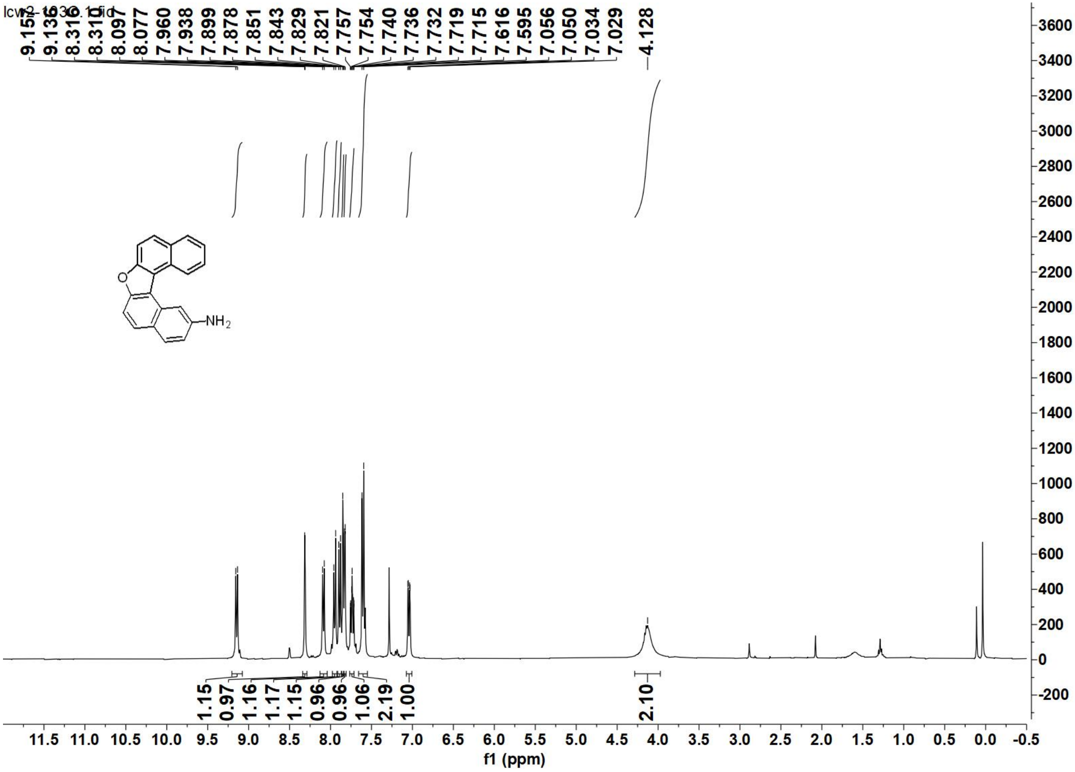

^{13}C NMR (101 MHz, $CDCl_3$) of **1m**

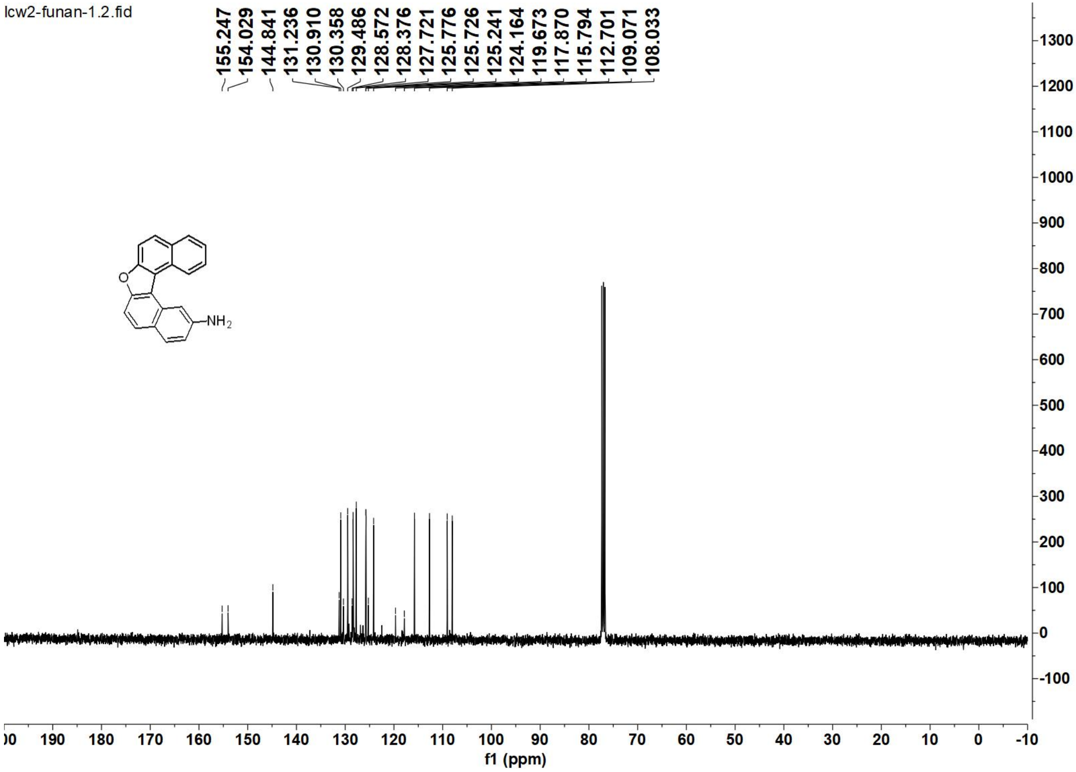

^{1}H NMR (400 MHz, DMSO-d$_6$) of **1n**

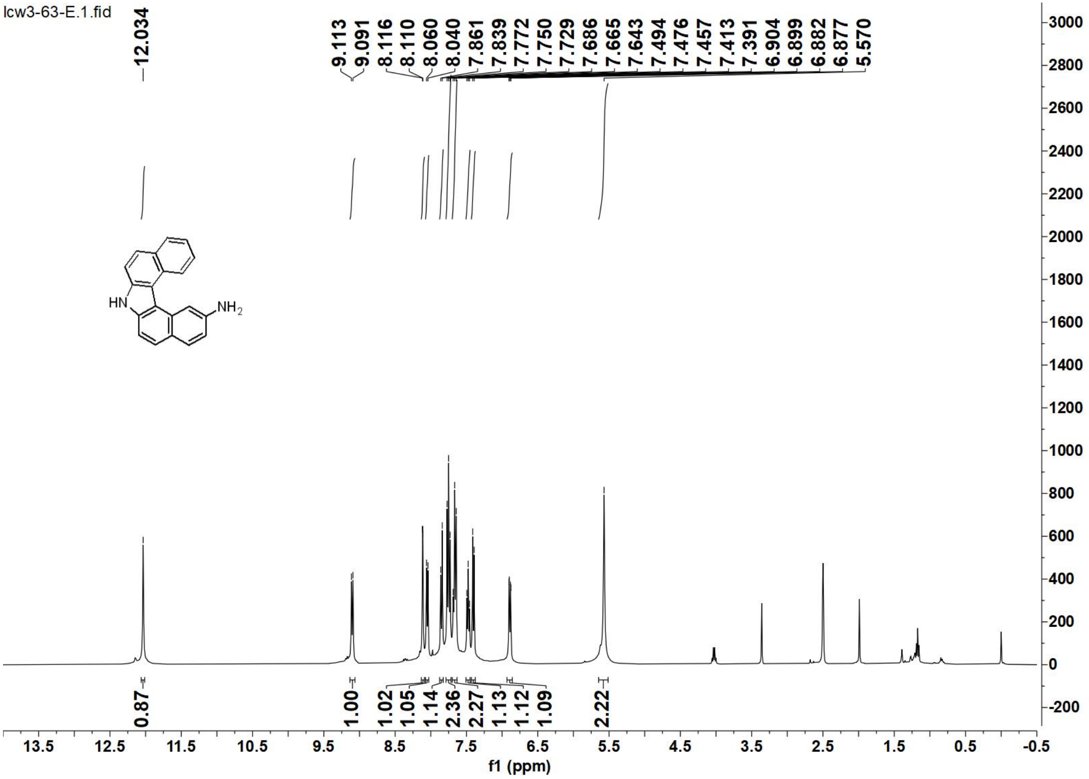

^{13}C NMR (101 MHz, DMSO-d_6) of **1n**

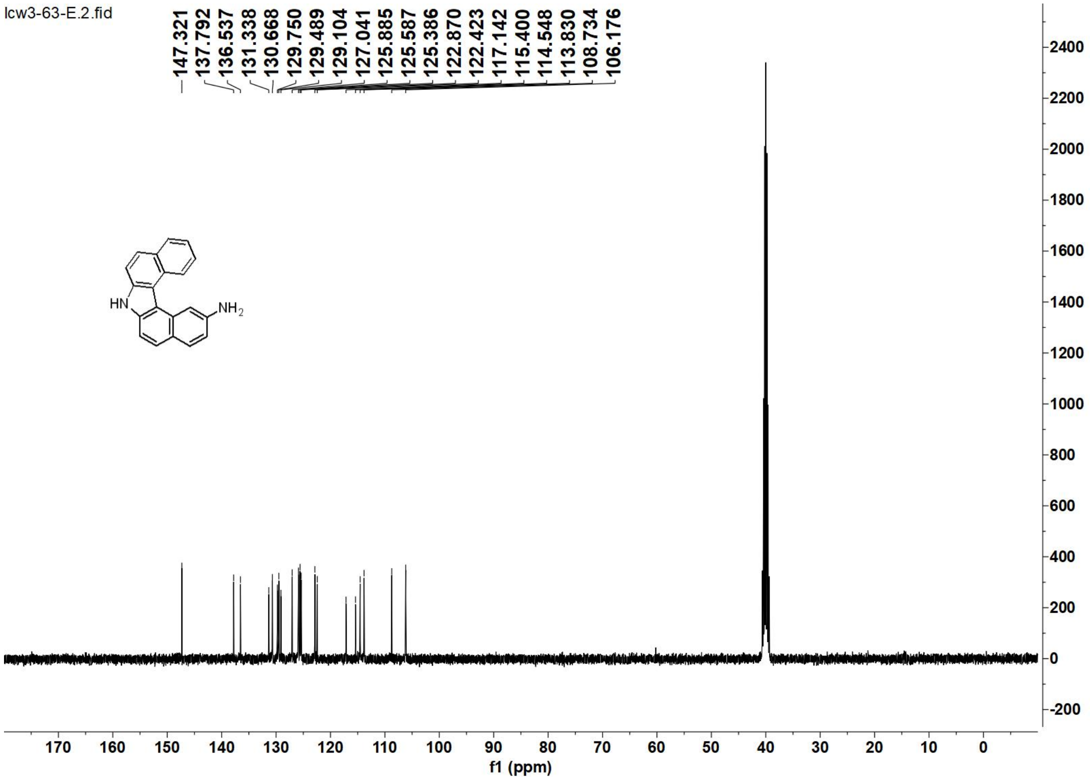

^1H NMR (400 MHz, DMSO-d_6) of **4a** (dr > 20:1)

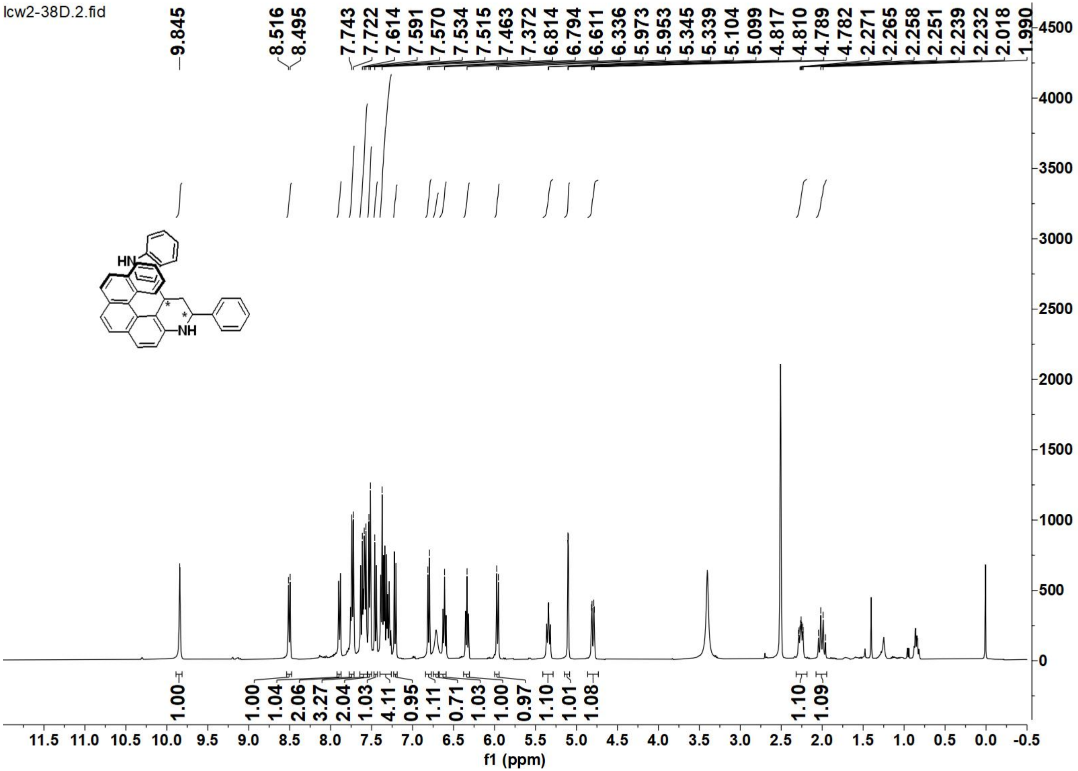

^{13}C NMR (101 MHz, DMSO-d_6) of **4a**(dr > 20:1)

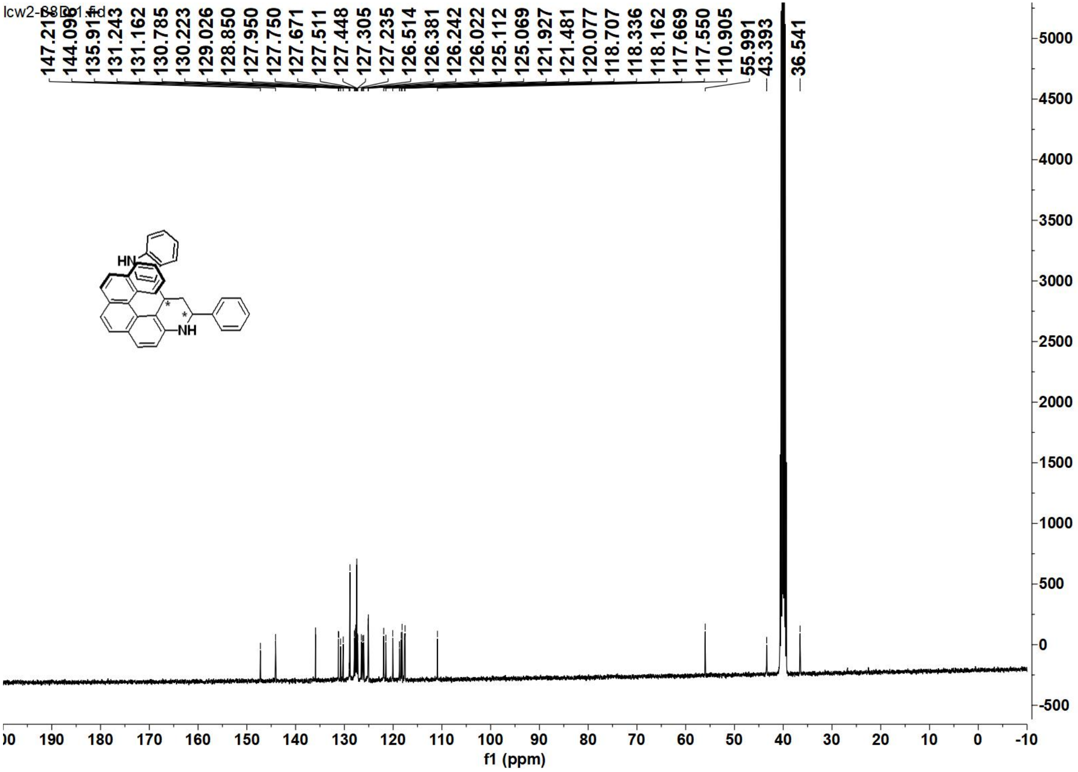

^{1}H NMR (400 MHz, DMSO-d_6) of **5a**

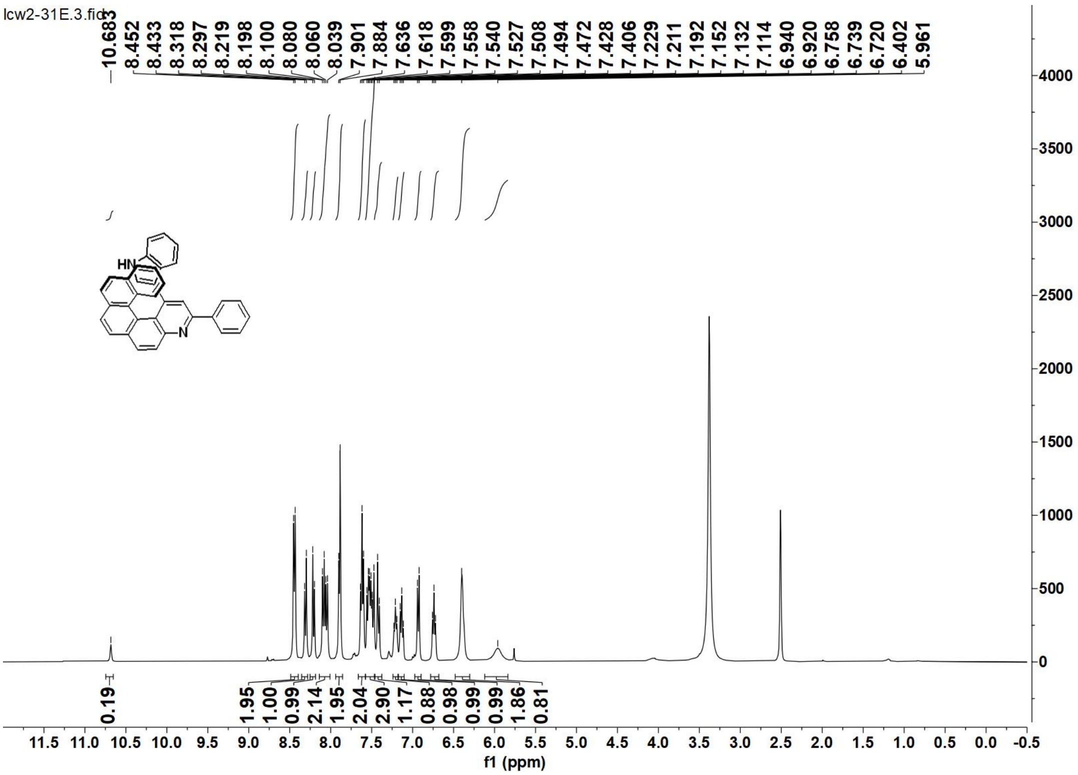

^{13}C NMR (101 MHz, DMSO-d_6) of **5a**

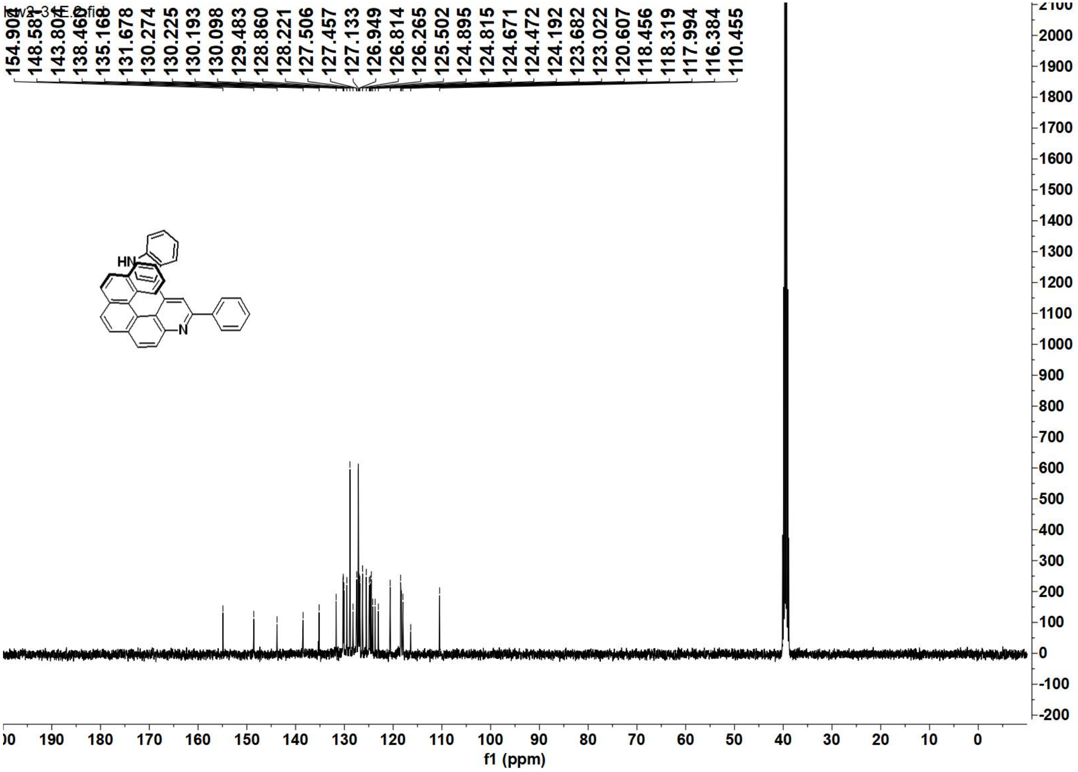

^{1}H NMR (400 MHz, DMSO-d_6) of **5b**

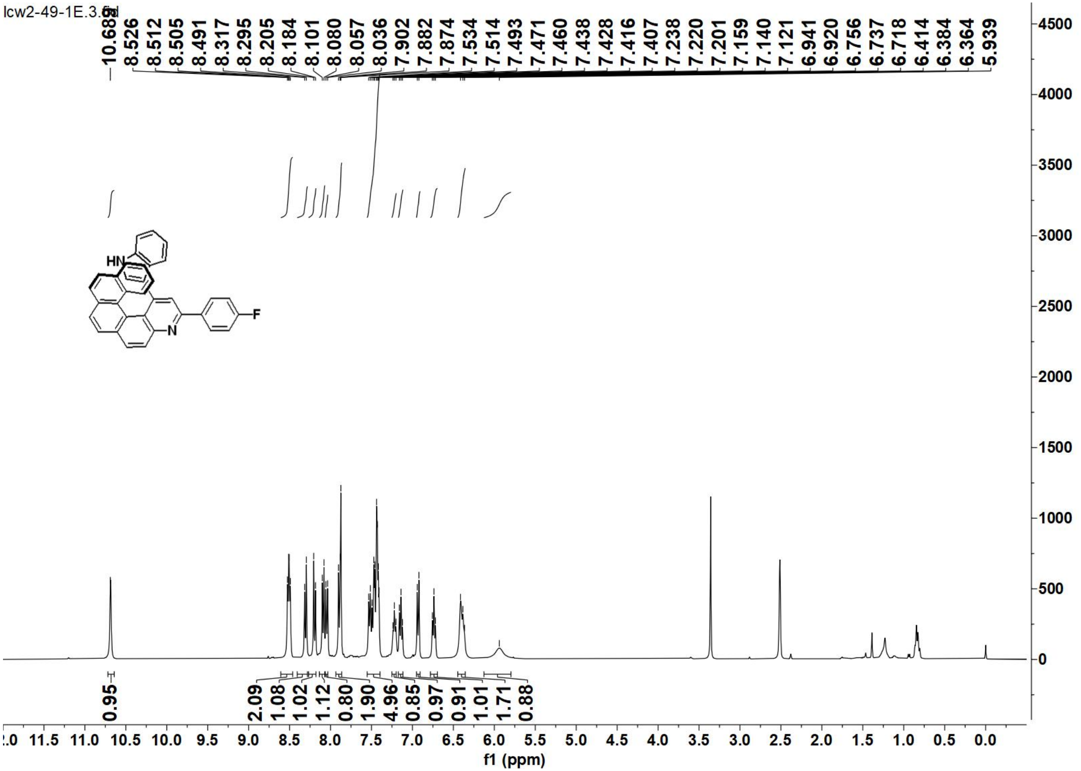

^{13}C NMR (101 MHz, DMSO-d_6) of **5b**

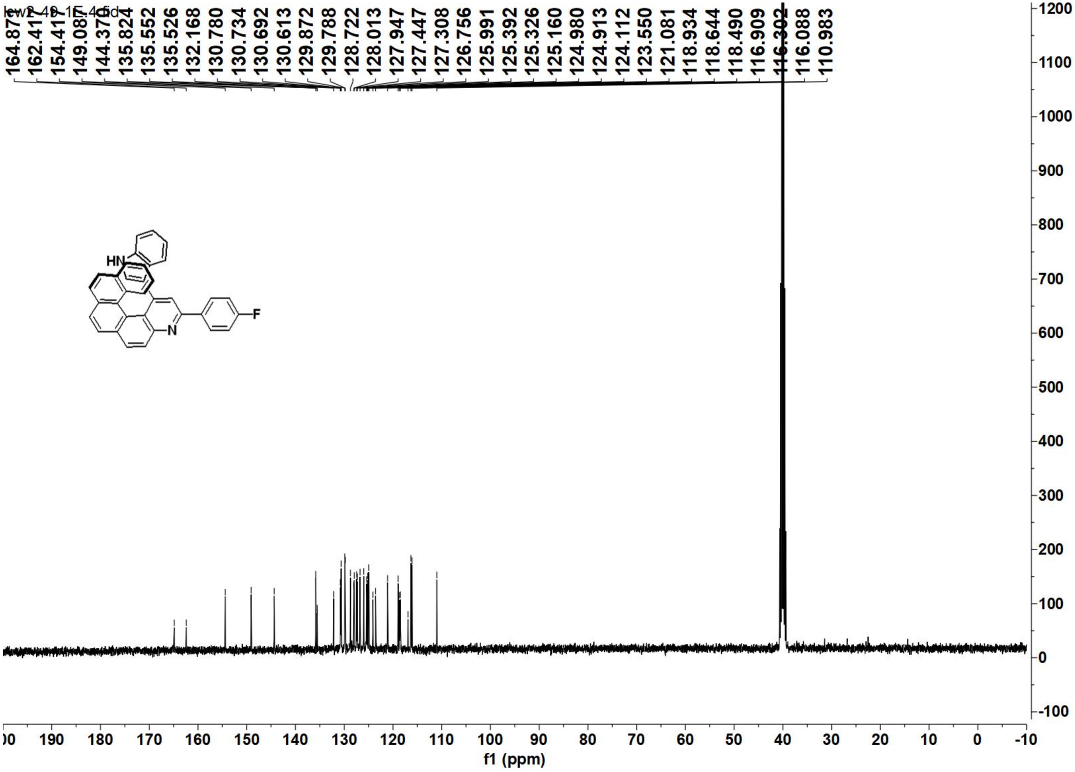

^{19}F NMR (376 MHz, DMSO-d_6) of **5b**

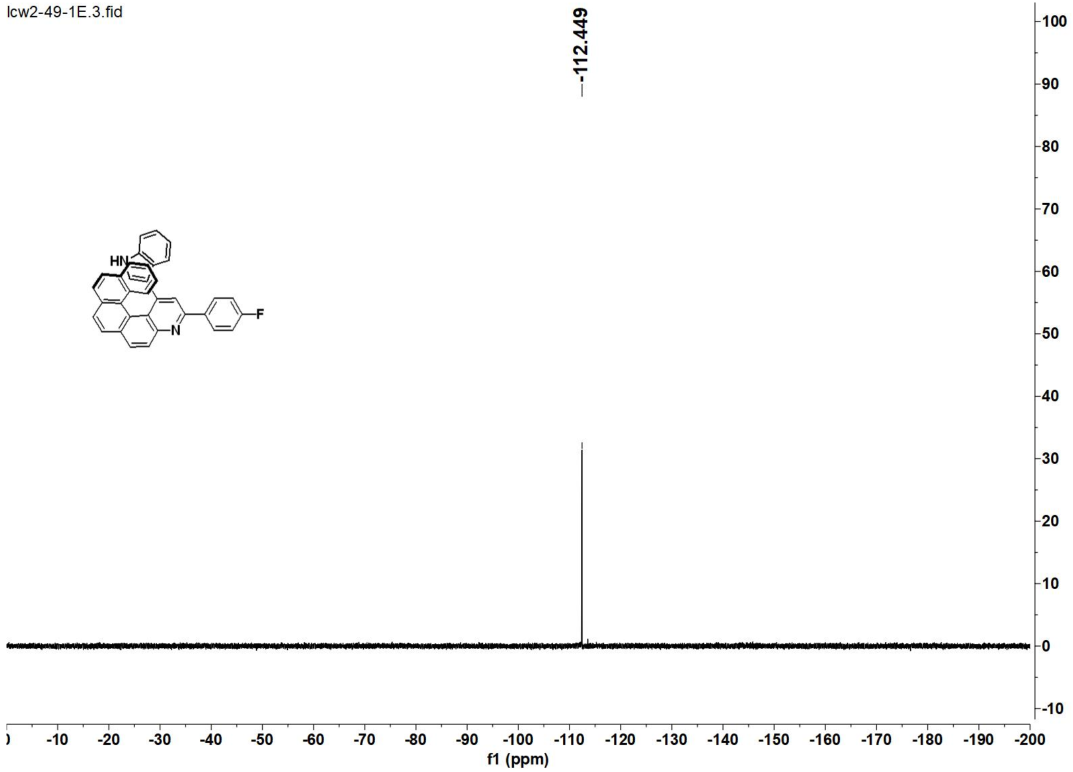

^{1}H NMR (400 MHz, DMSO-d_6) of **5c**

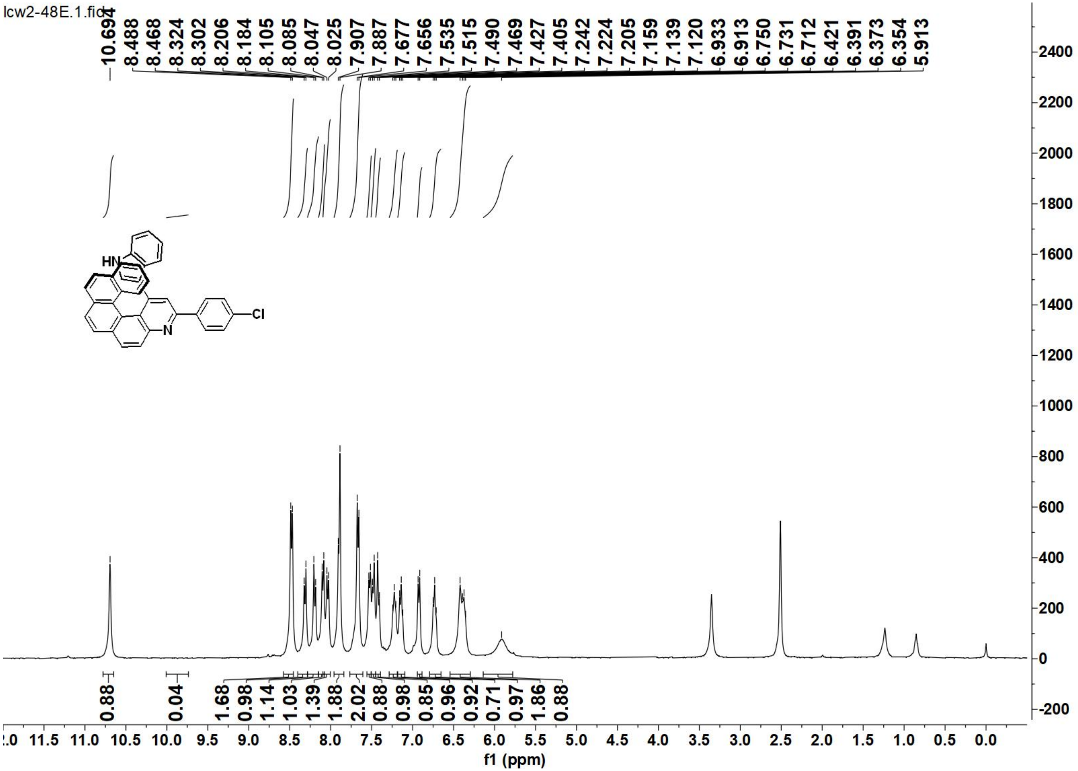

^{13}C NMR (101 MHz, DMSO-d_6) of **5c**

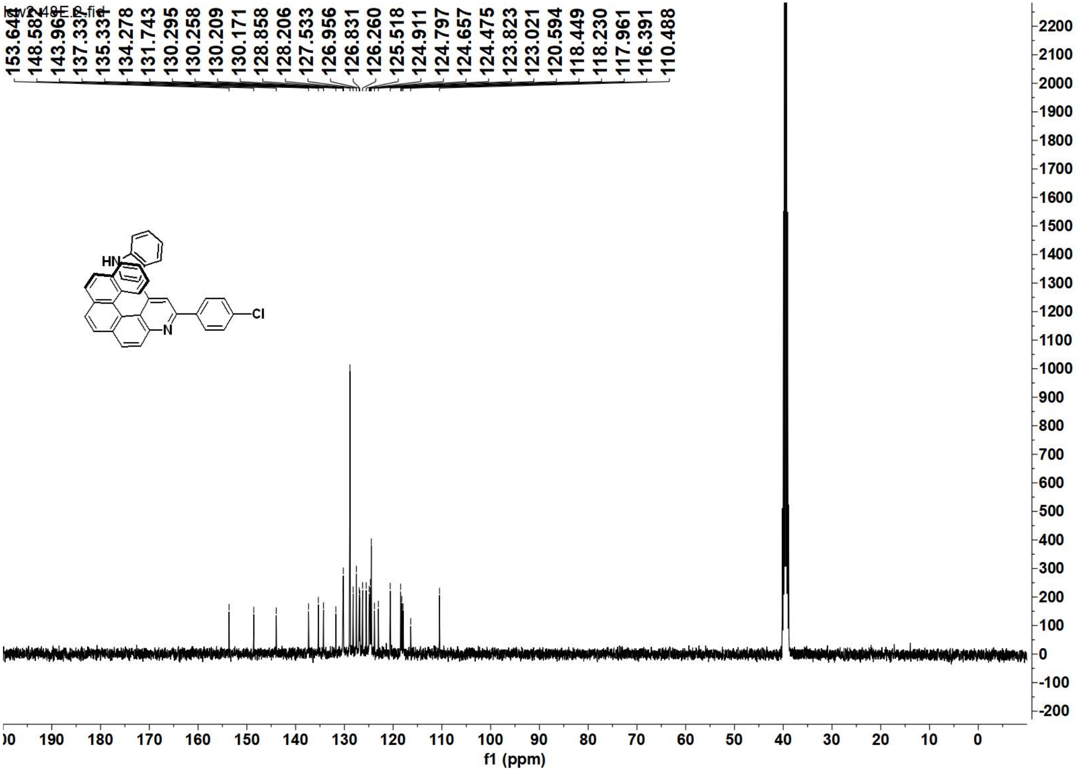

^{1}H NMR (400 MHz, DMSO-d_6) of **5d**

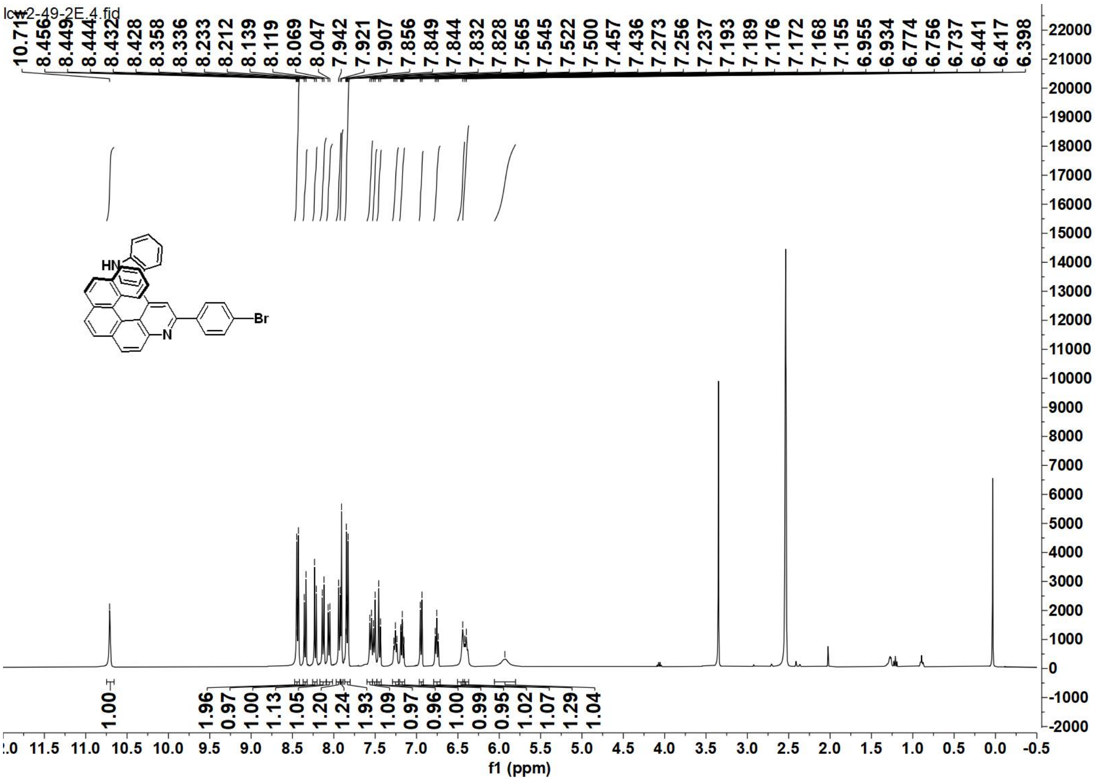

^{13}C NMR (101 MHz, DMSO-d_6) of **5d**

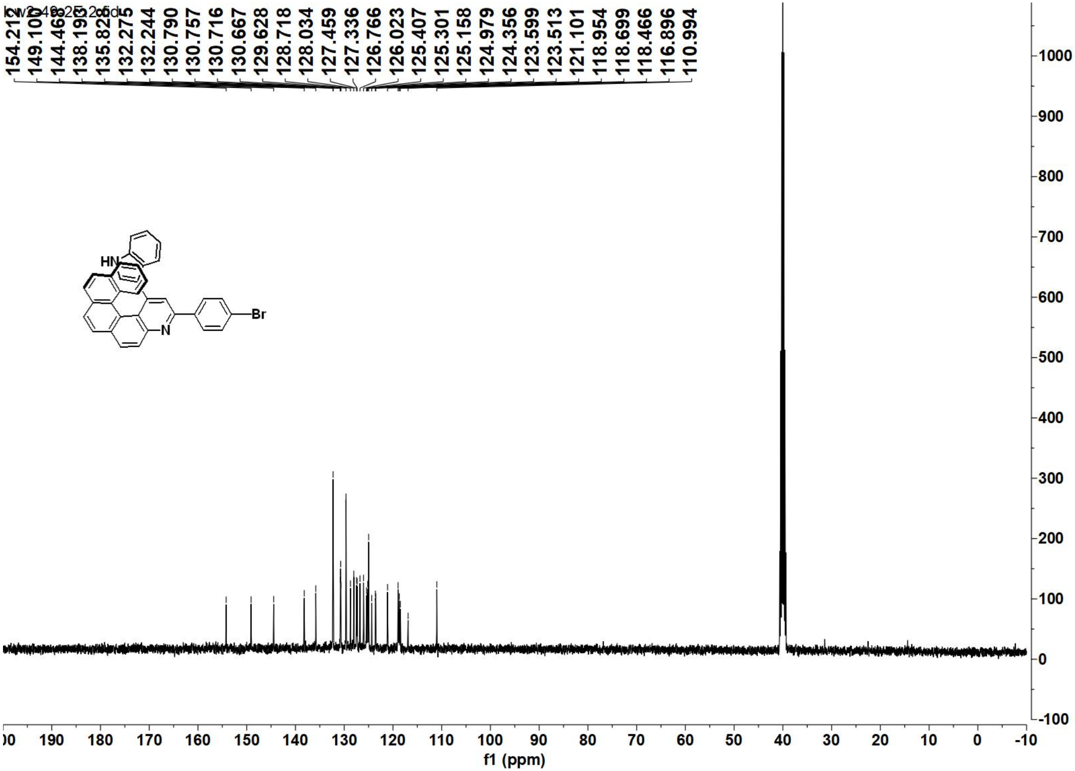

^{1}H NMR (400 MHz, DMSO-d_6) of **5e**

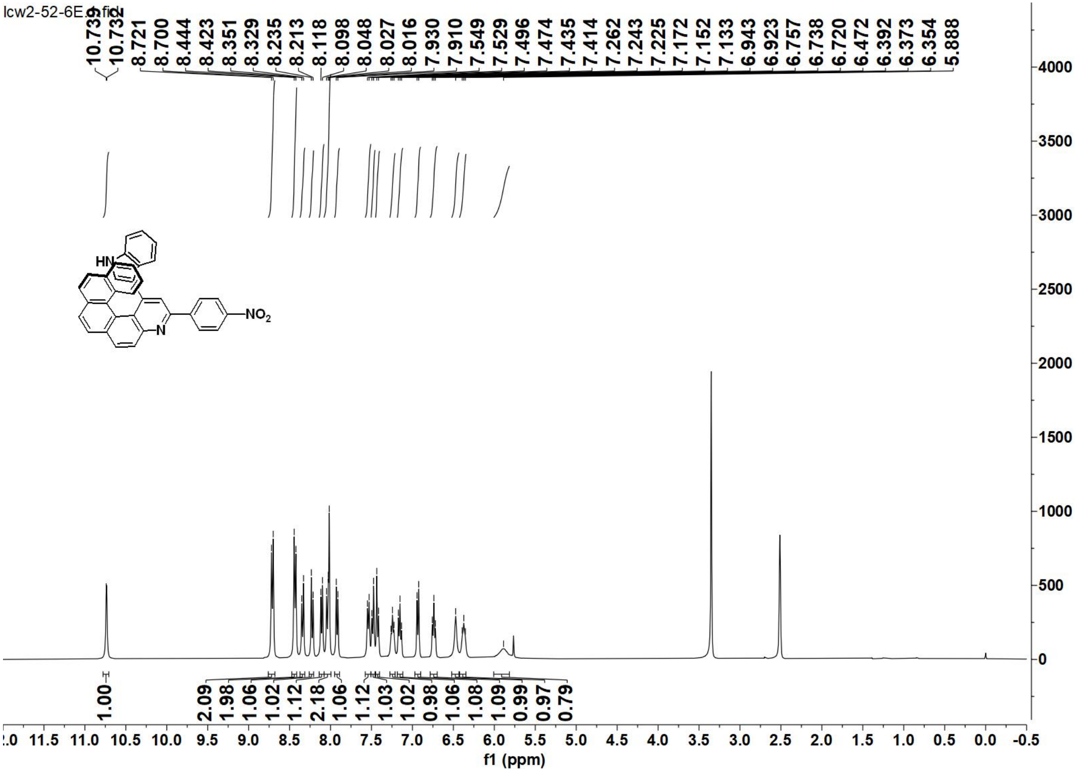

^{13}C NMR (101 MHz, DMSO-d_6) of **5e**

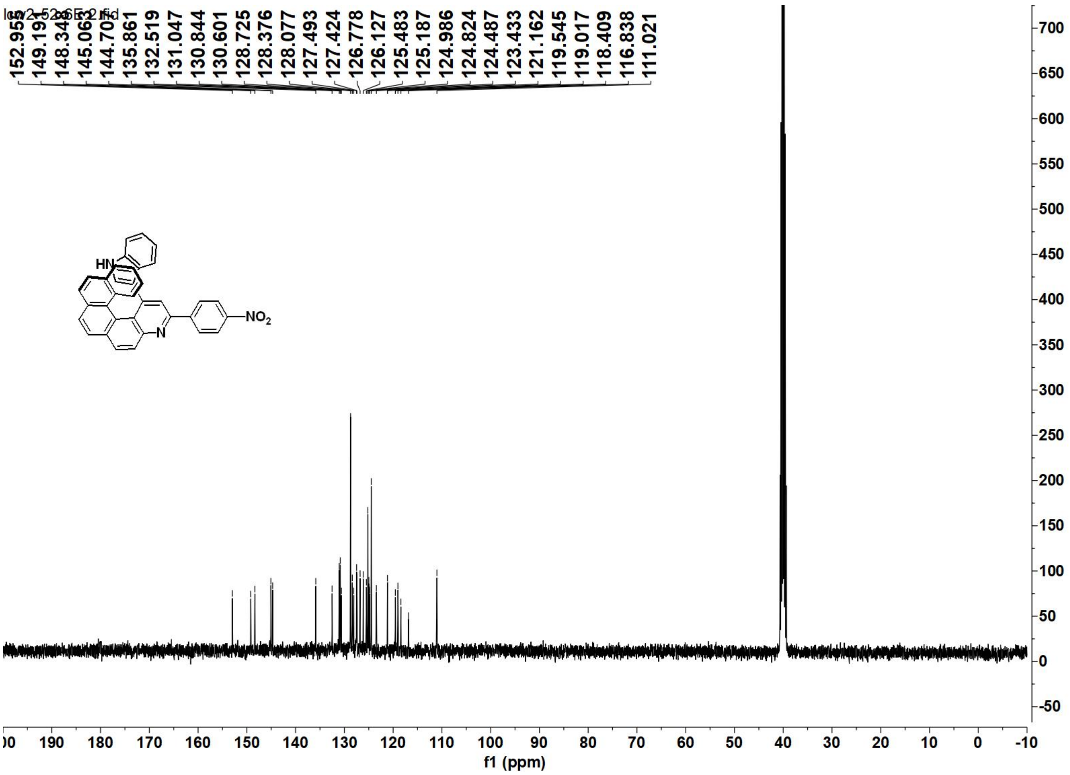

^{1}H NMR (400 MHz, DMSO-d_6) of **5f**

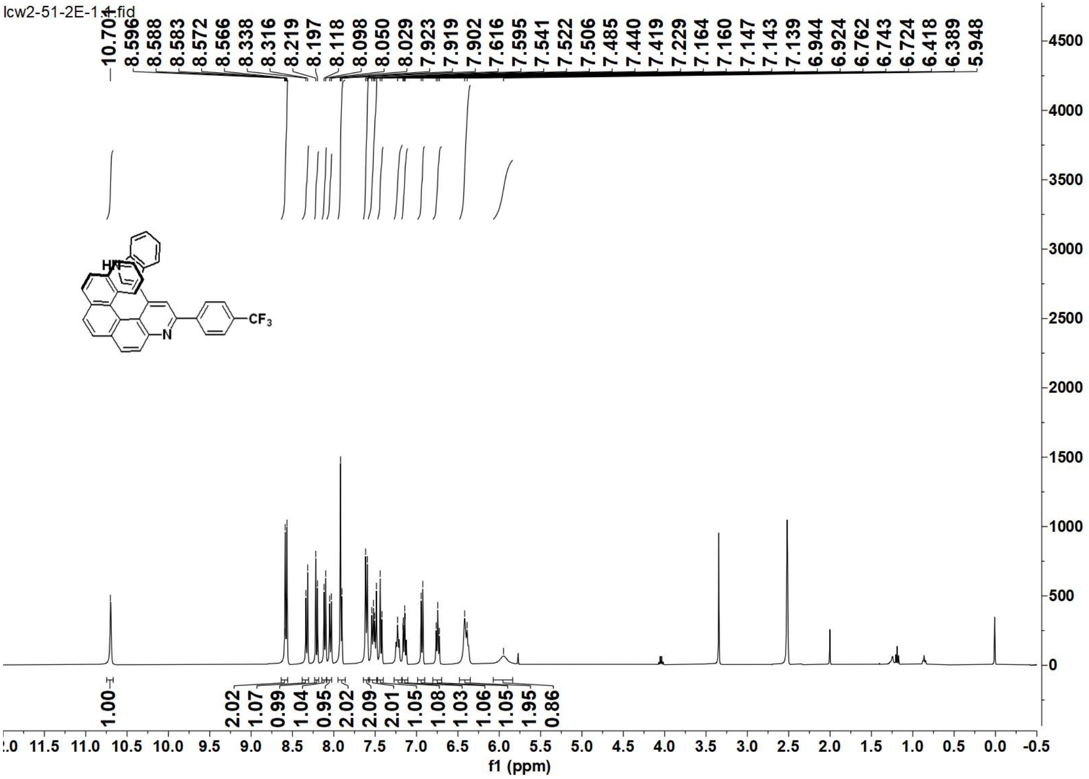

^{13}C NMR (101 MHz, DMSO-d_6) of **5f**

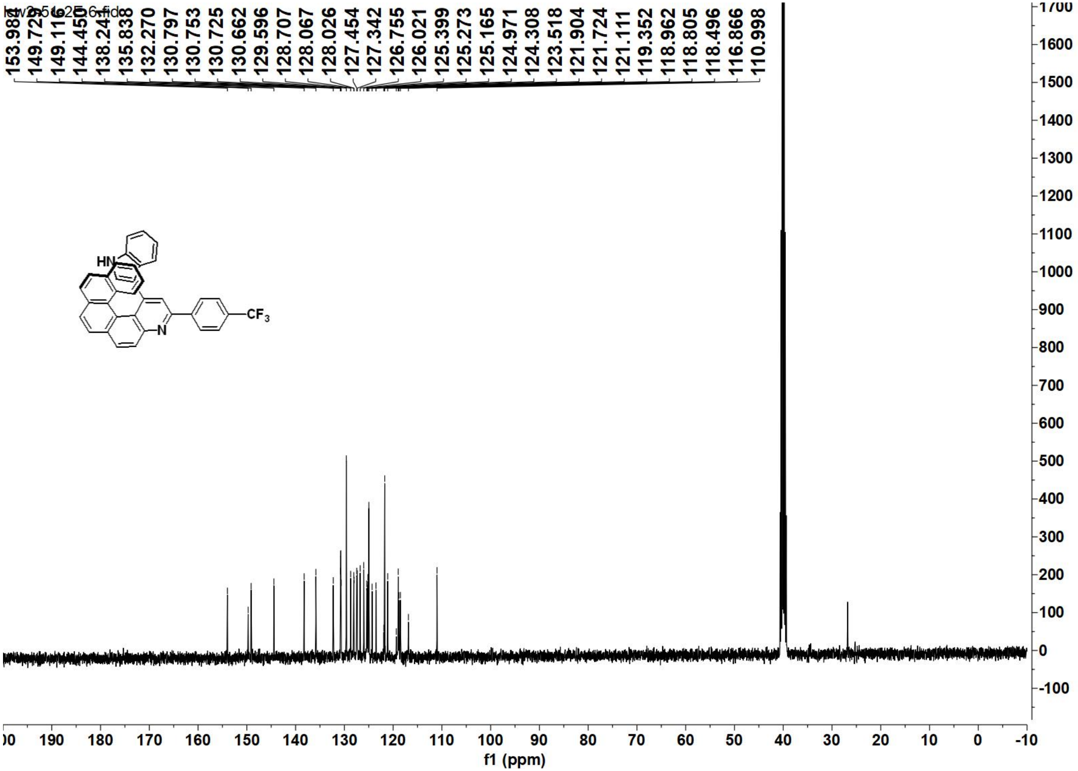

^{19}F NMR (376 MHz, DMSO-d_6) of **5f**

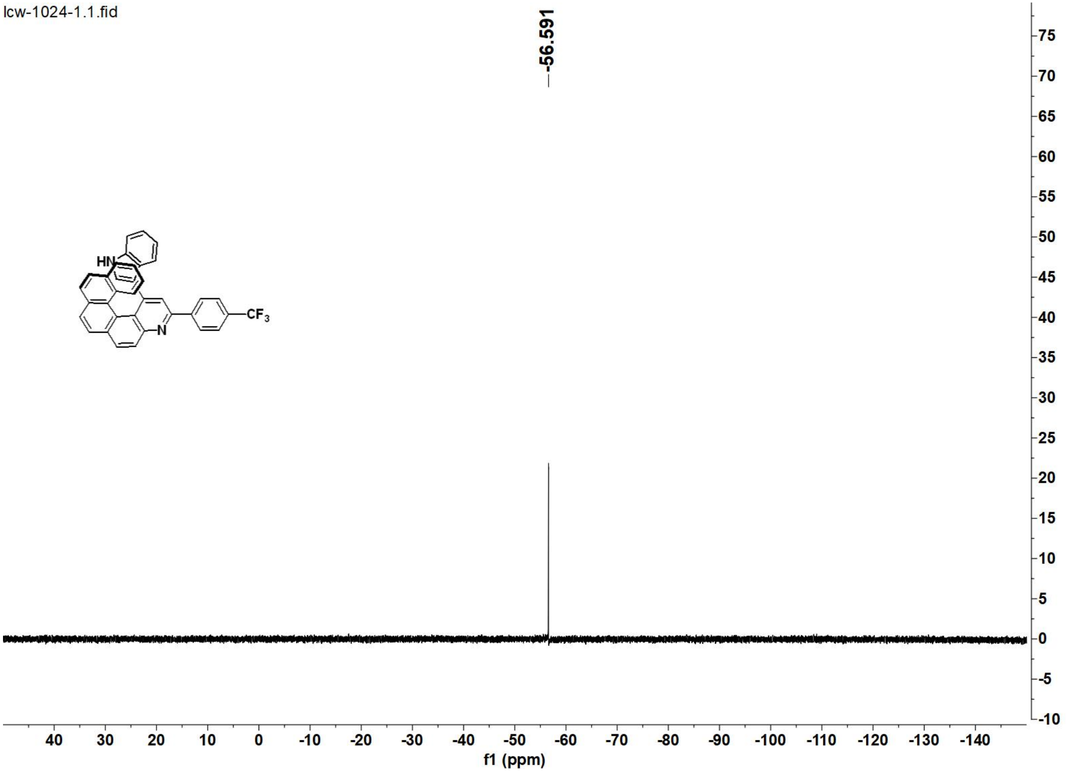

^{1}H NMR (400 MHz, DMSO-d_6) of **5g**

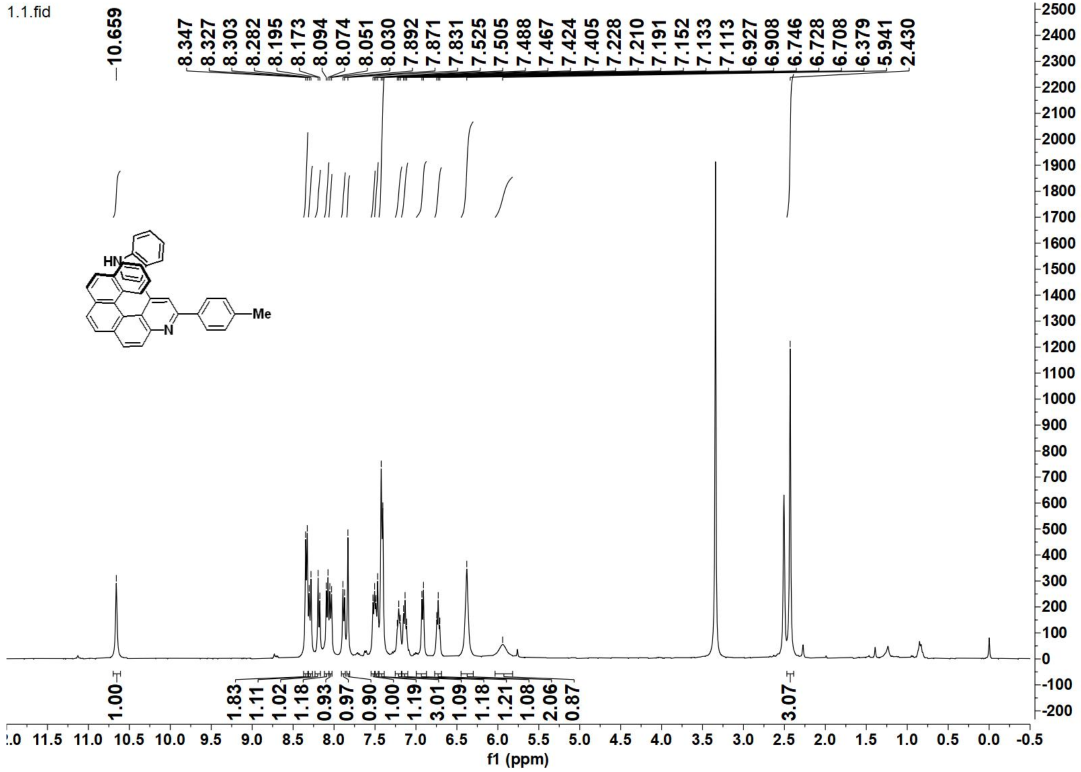

^{13}C NMR (101 MHz, DMSO-d_6) of **5g**

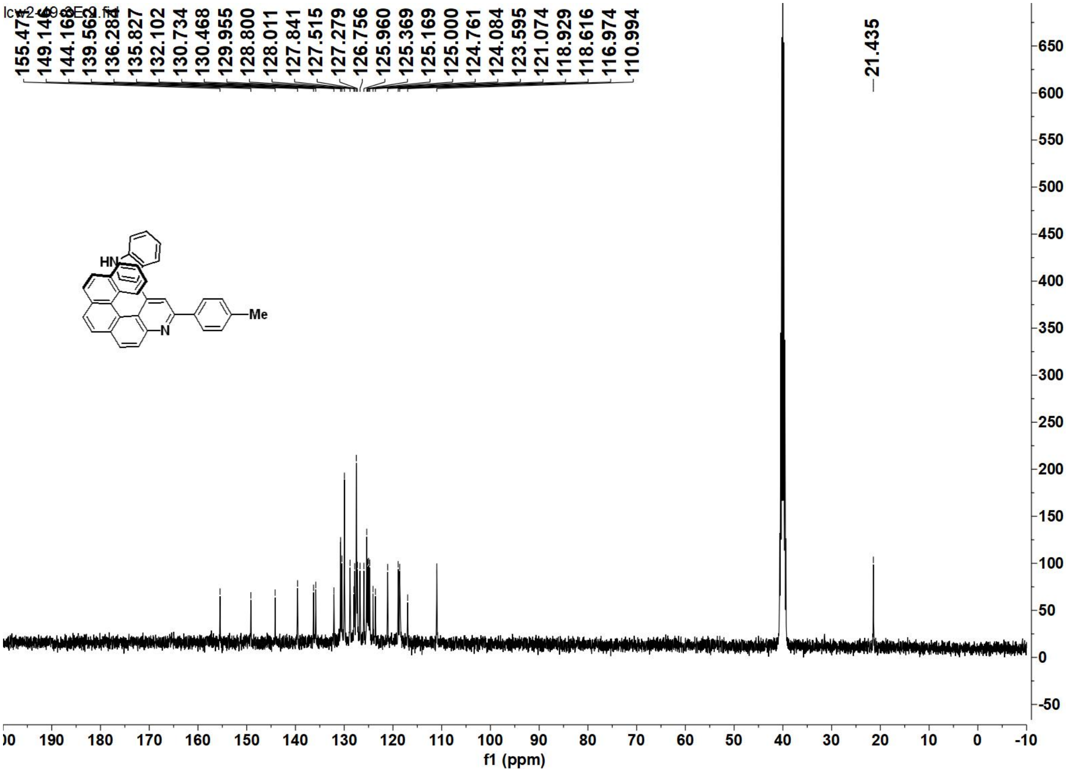

^1H NMR (400 MHz, DMSO-d_6) of **5h**

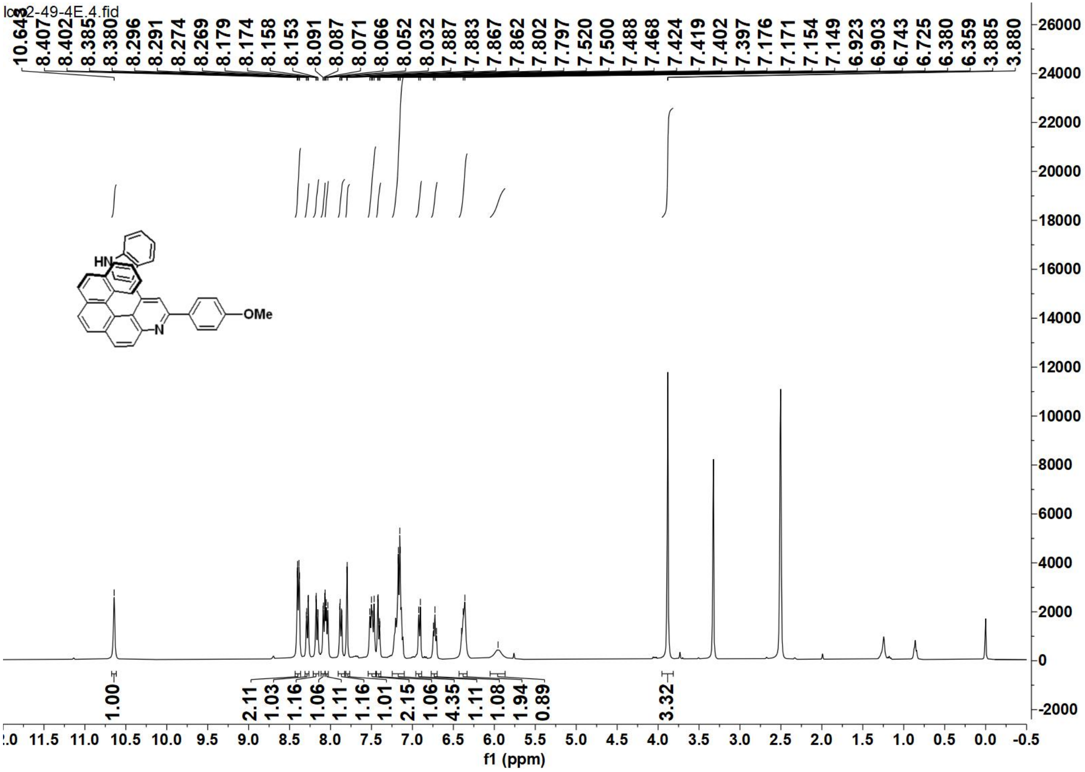

^{13}C NMR (101 MHz, DMSO-d_6) of **5h**

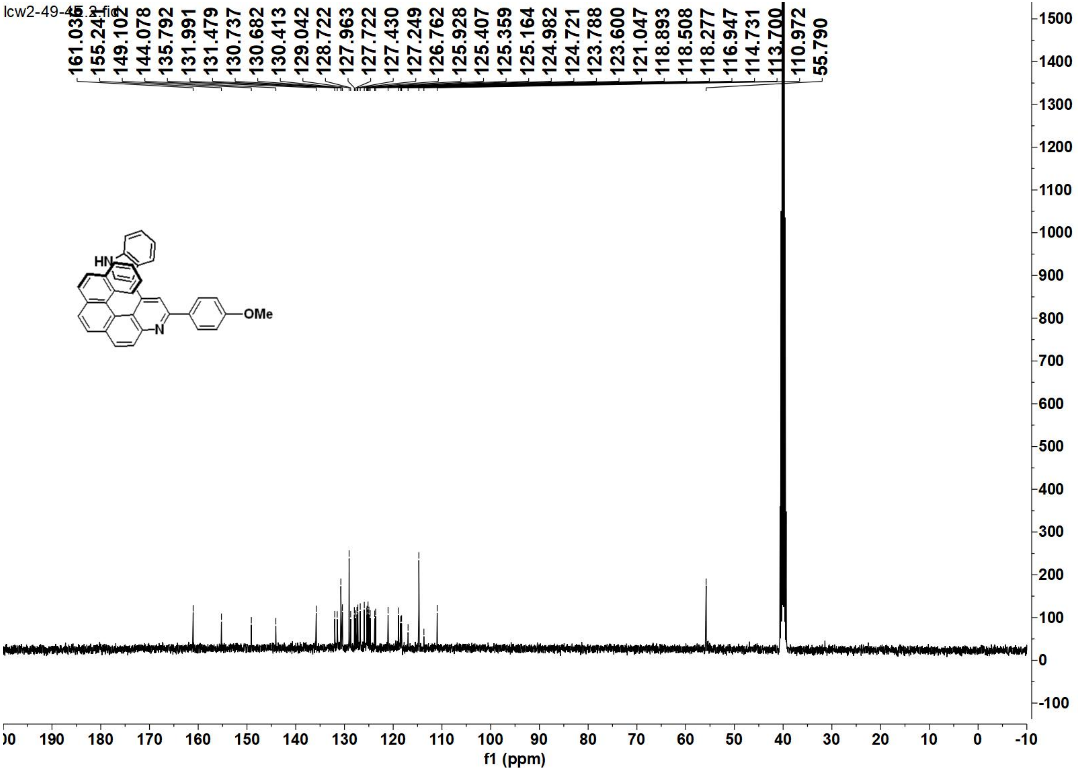

^{1}H NMR (400 MHz, DMSO-d_6) of **5i**

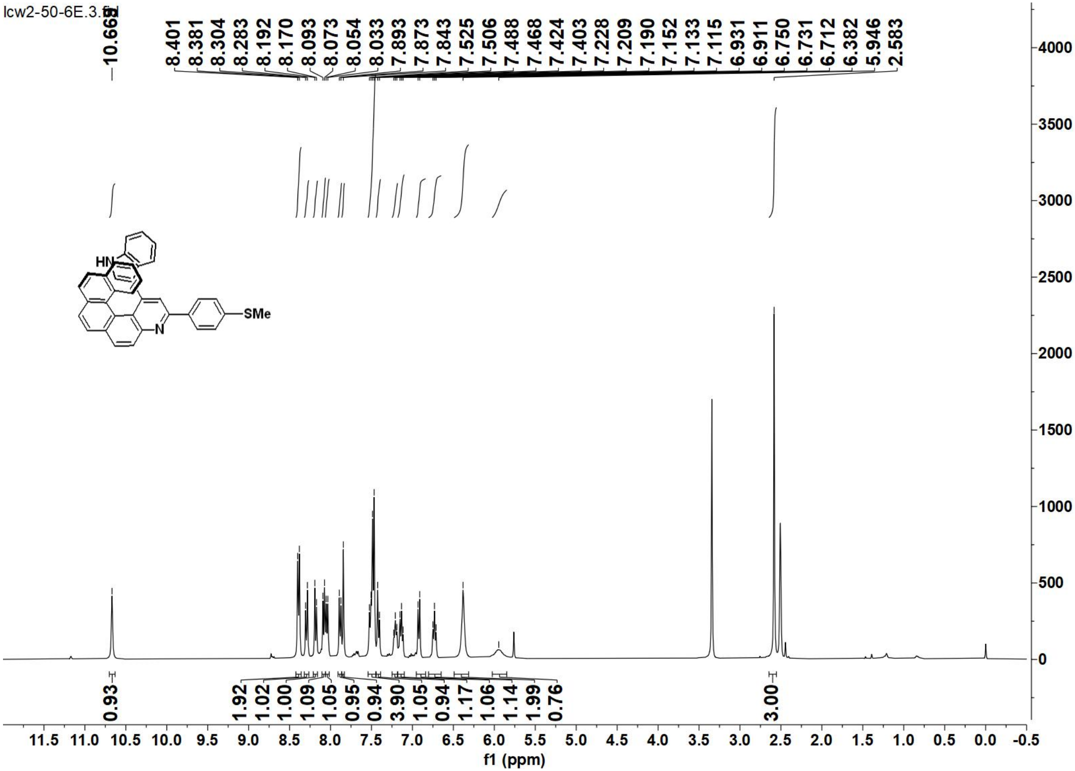

^{13}C NMR (101 MHz, DMSO-d_6) of **5i**

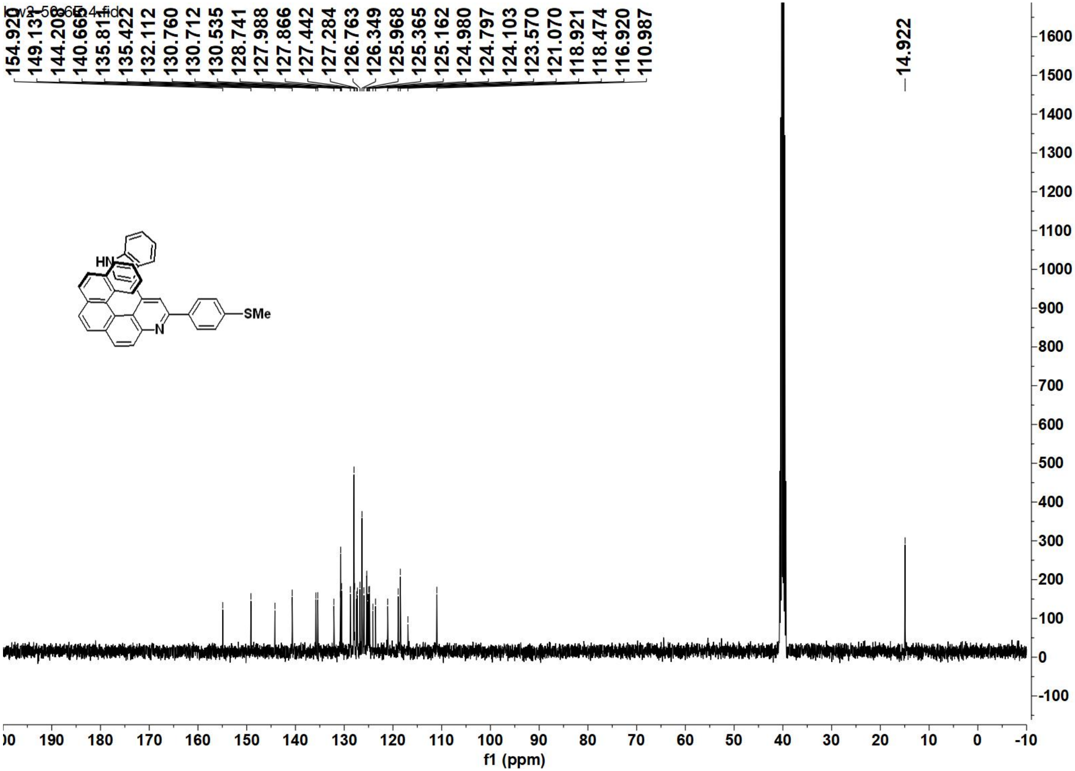

^{1}H NMR (400 MHz, DMSO-d_6) of **5j**

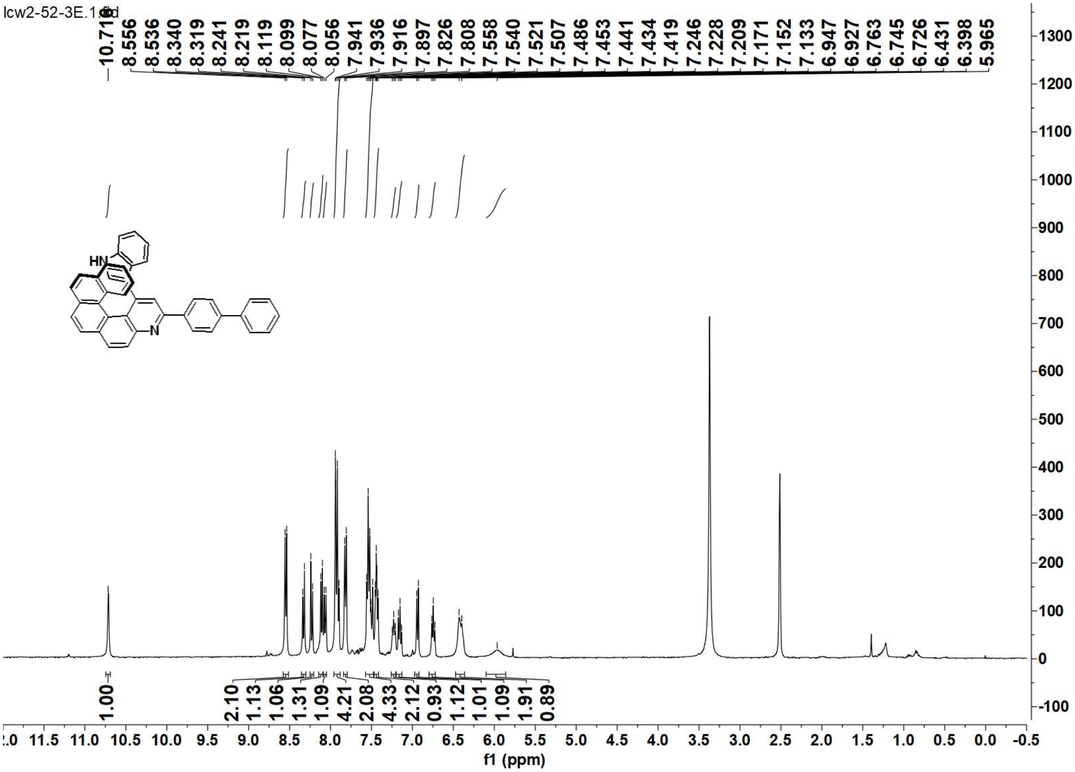

^{13}C NMR (101 MHz, DMSO-d_6) of **5j**

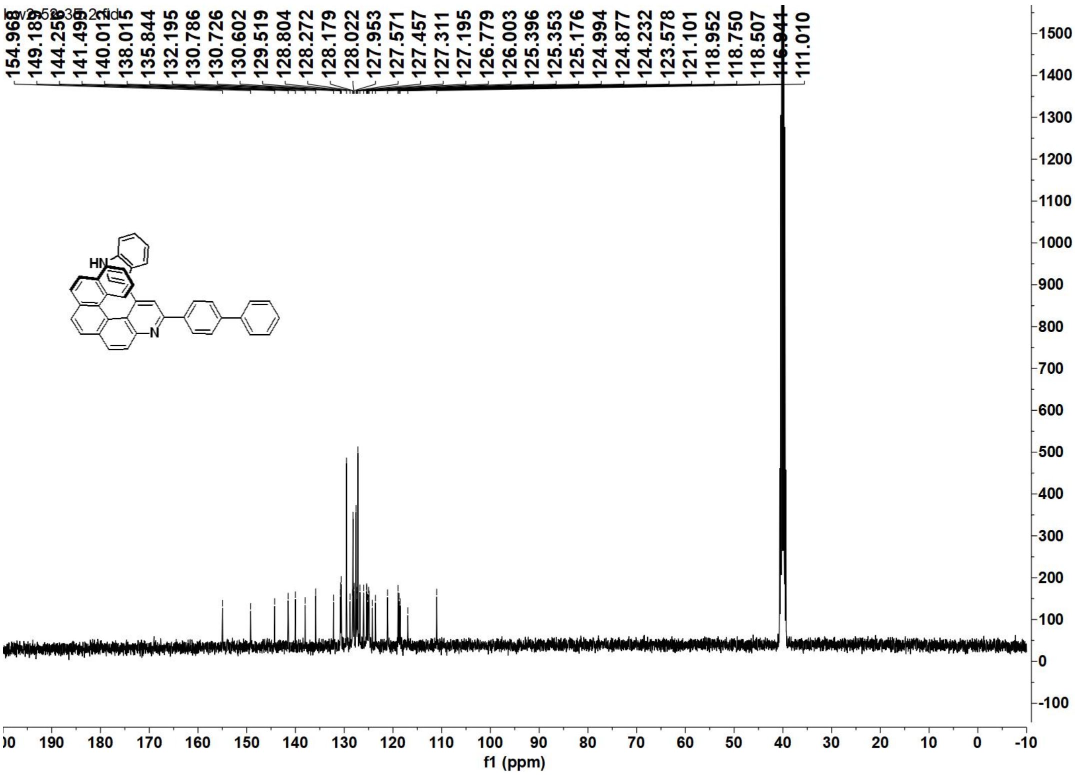

^{1}H NMR (400 MHz, DMSO-d_6) of **5k**

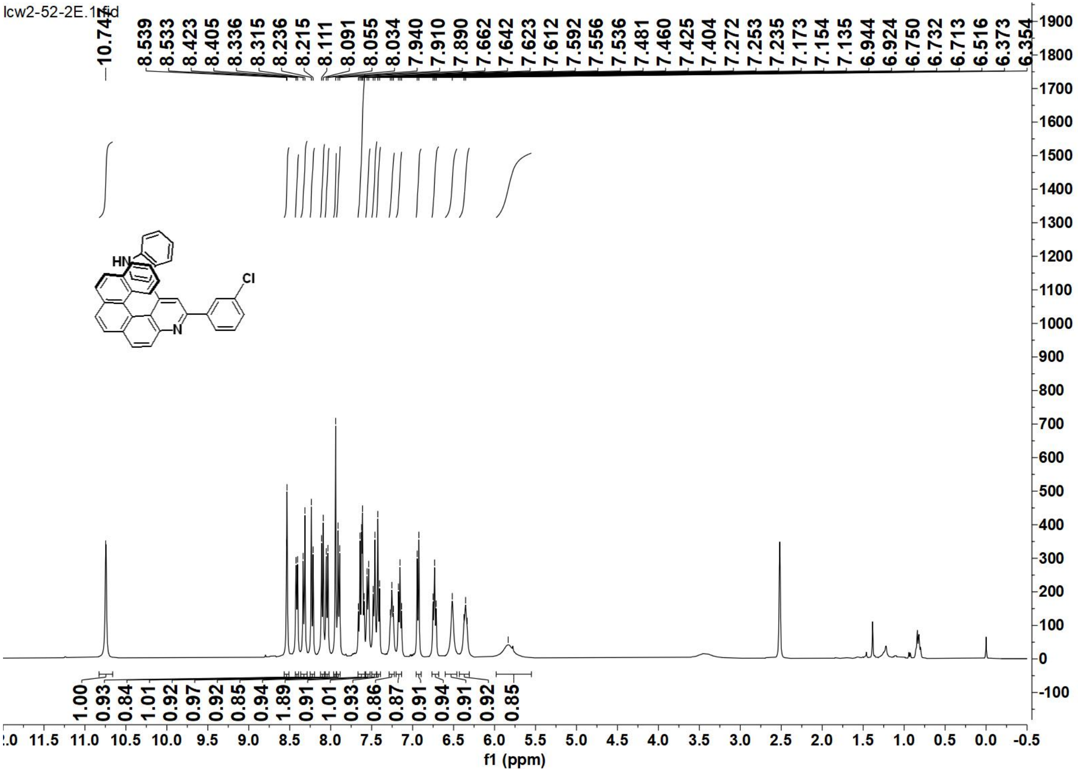

^{13}C NMR (101 MHz, DMSO-d_6) of **5k**

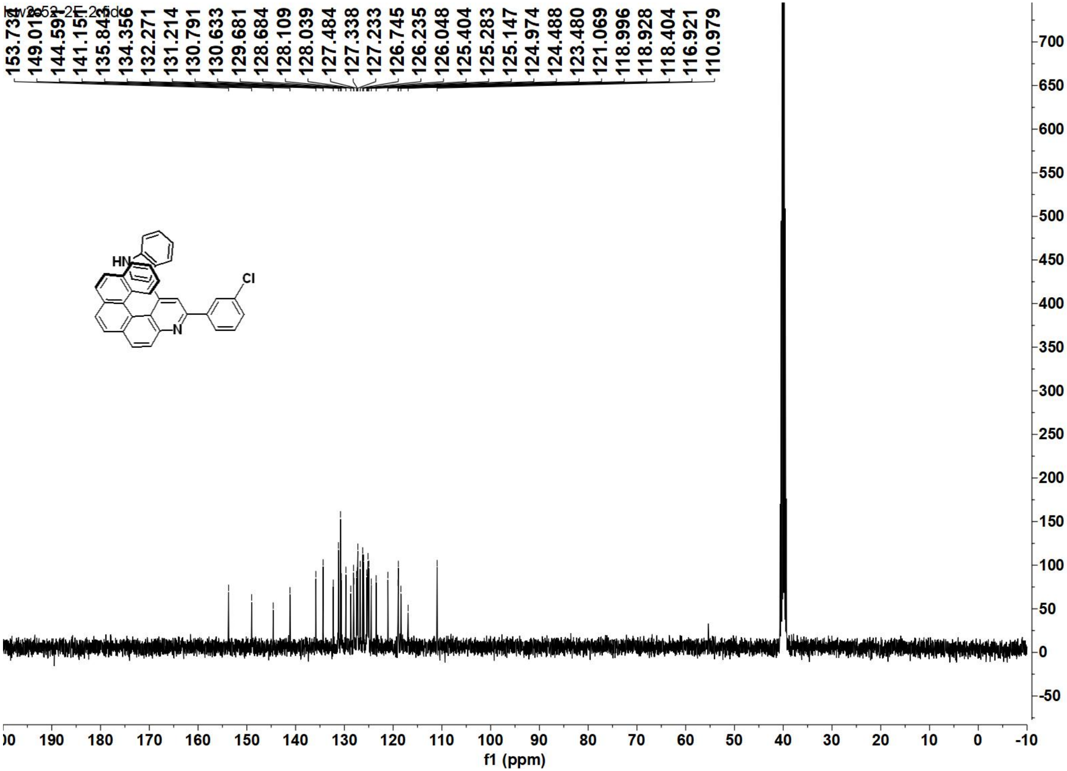

^{1}H NMR (400 MHz, DMSO-d_6) of **5l**

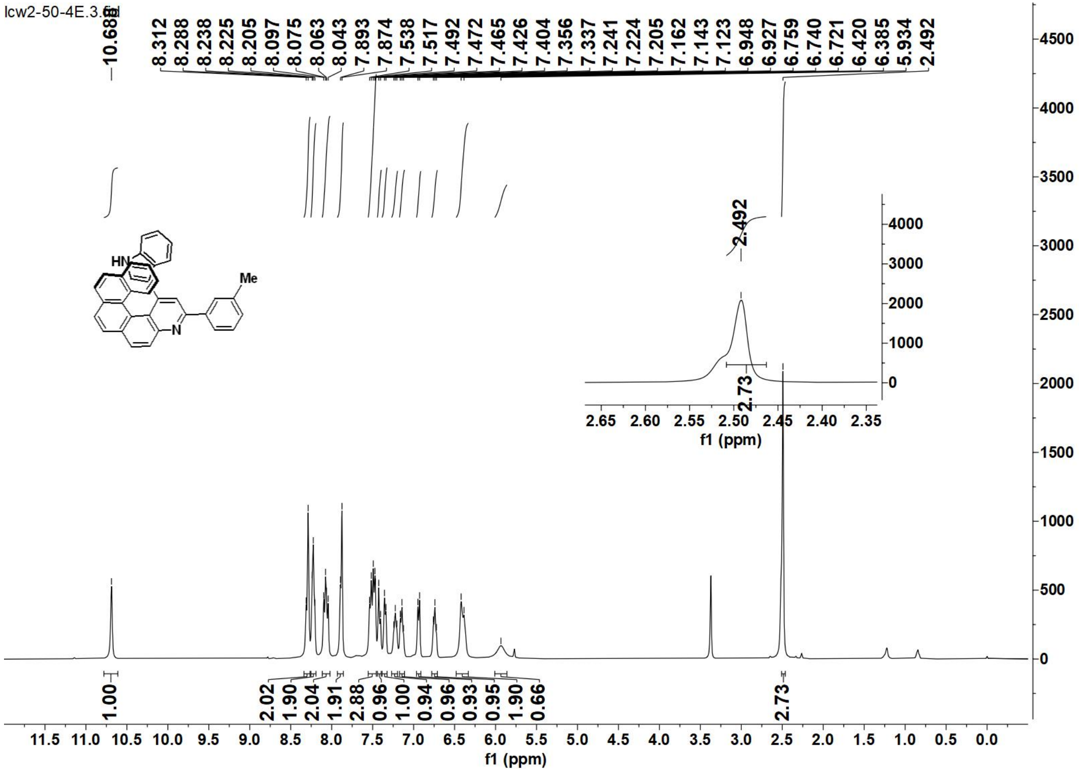

^{13}C NMR (101 MHz, DMSO-d_6) of **5l**

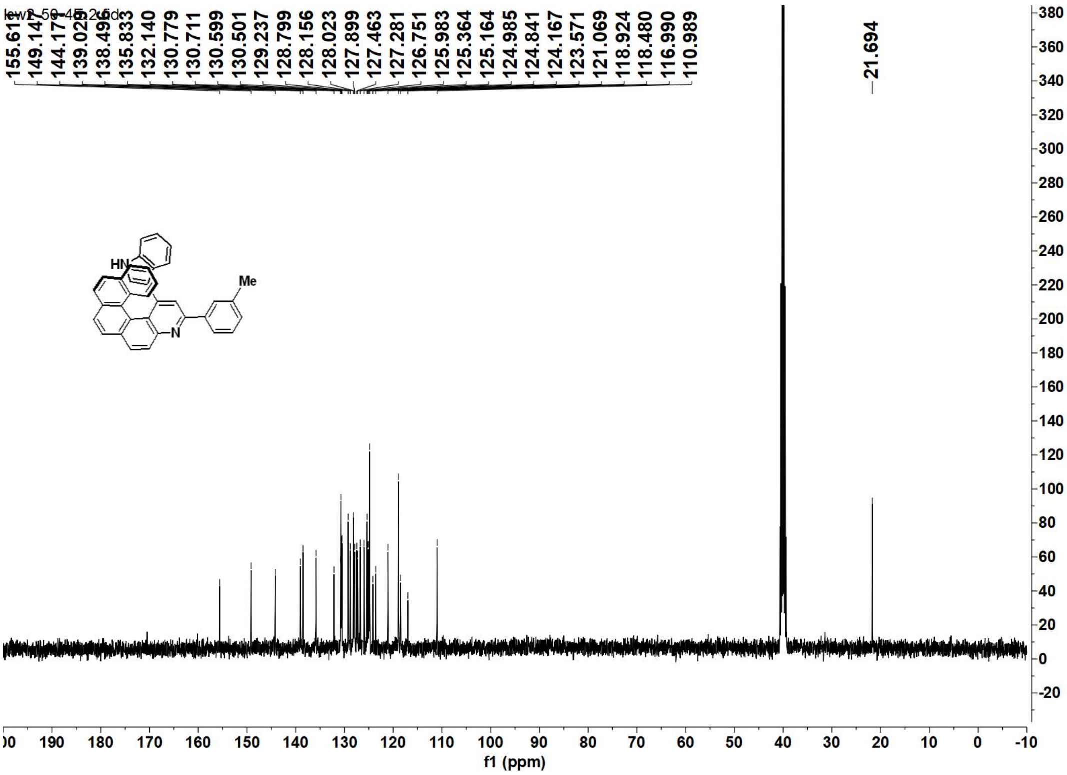

^{1}H NMR (400 MHz, DMSO-d_6) of **5m**

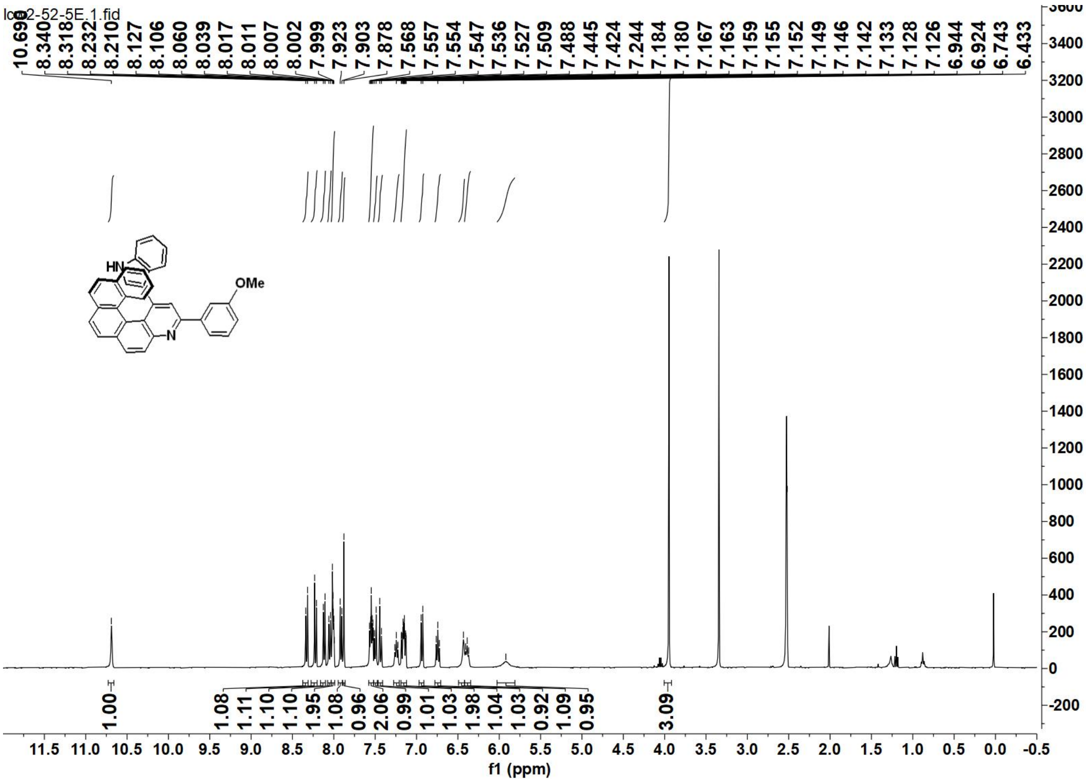

^{13}C NMR (400 MHz, DMSO-d_6) of **5m**

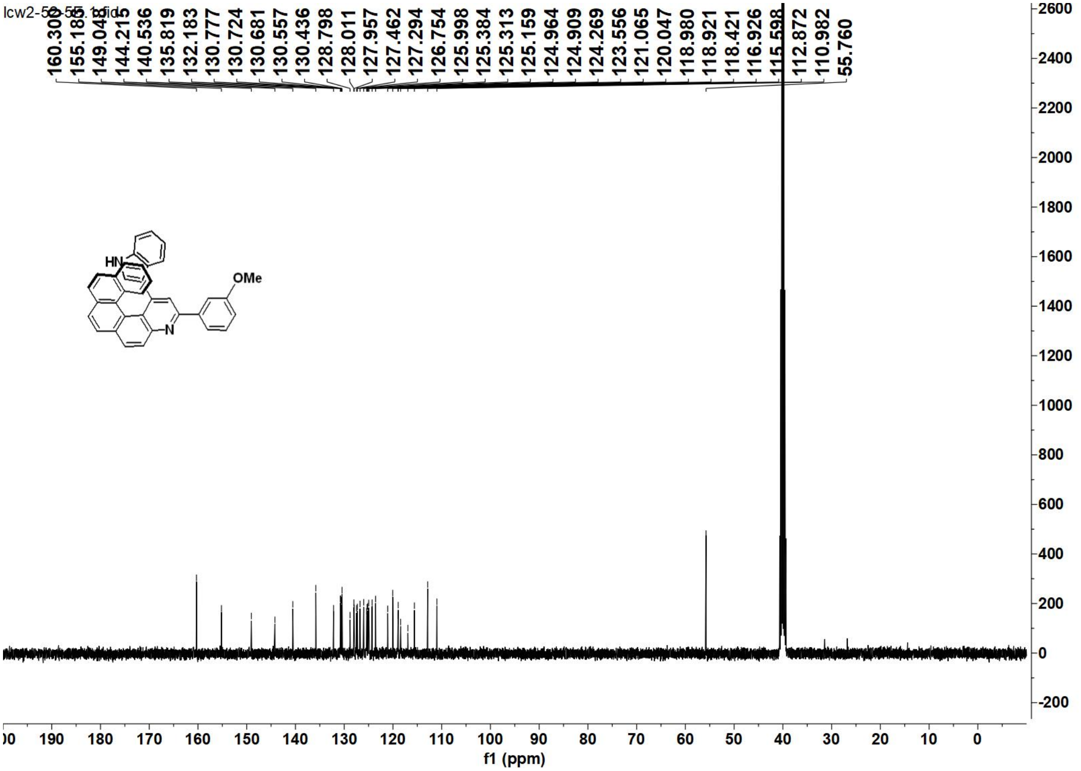

^{1}H NMR (400 MHz, DMSO-d_6) of **5n**

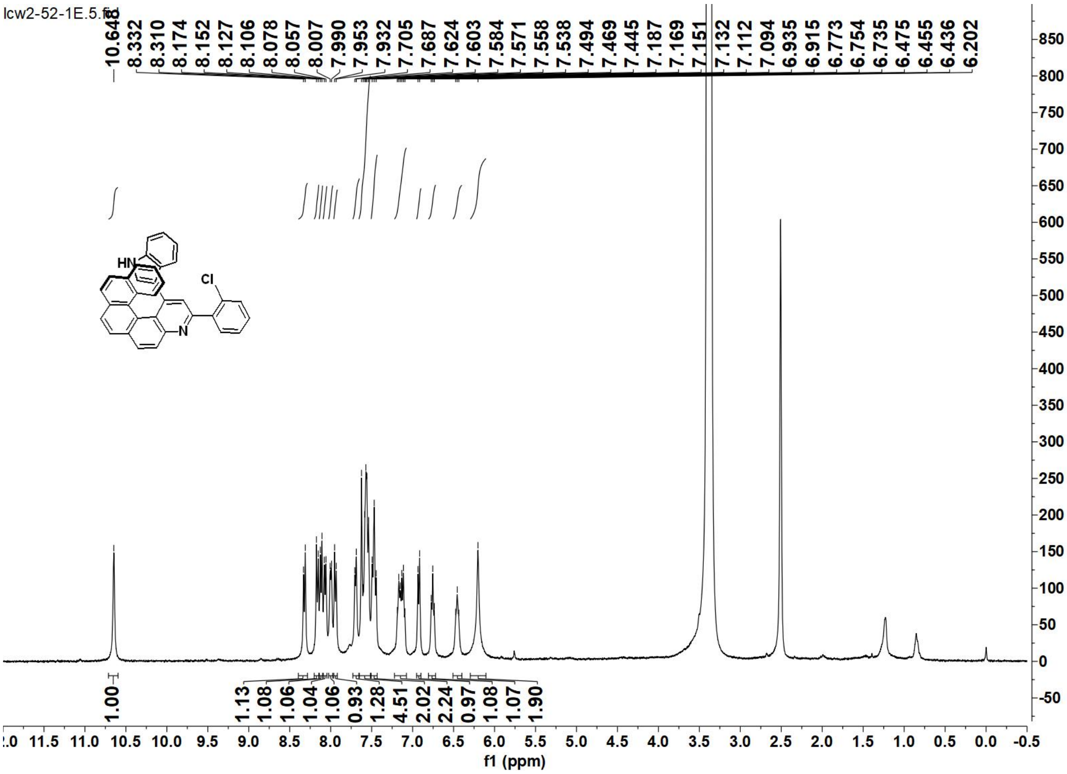

^{13}C NMR (101 MHz, DMSO-d_6) of **5n**

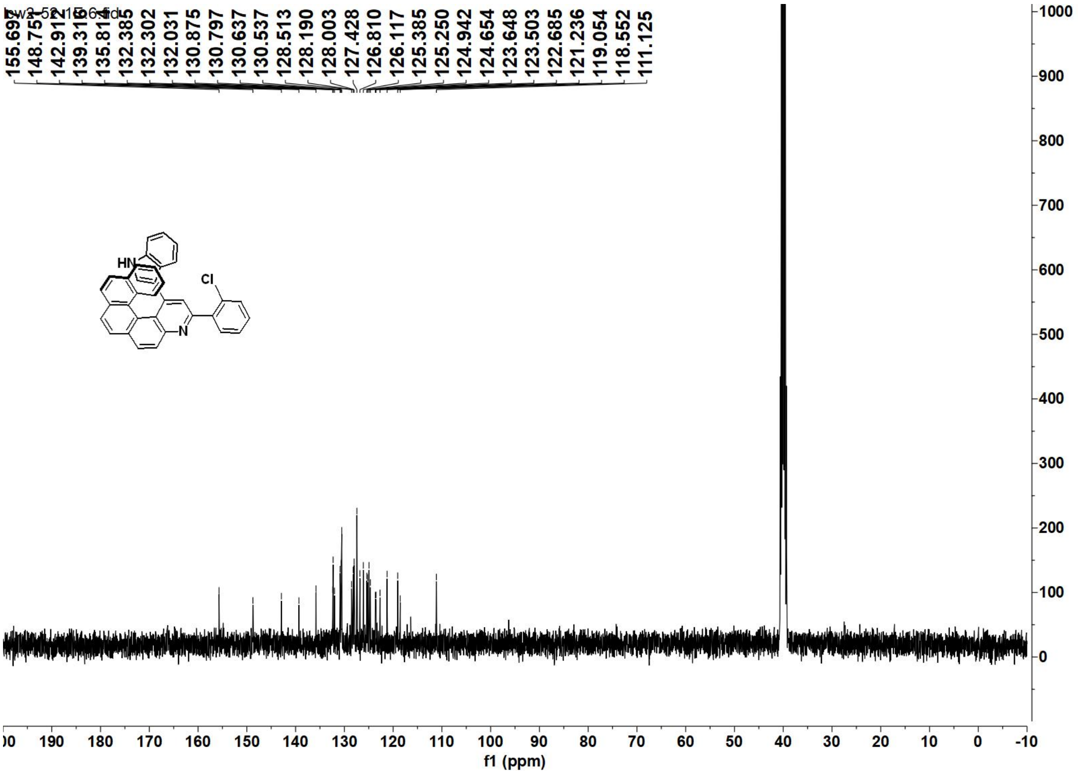

^{1}H NMR (400 MHz, DMSO-d_6) of **5o**

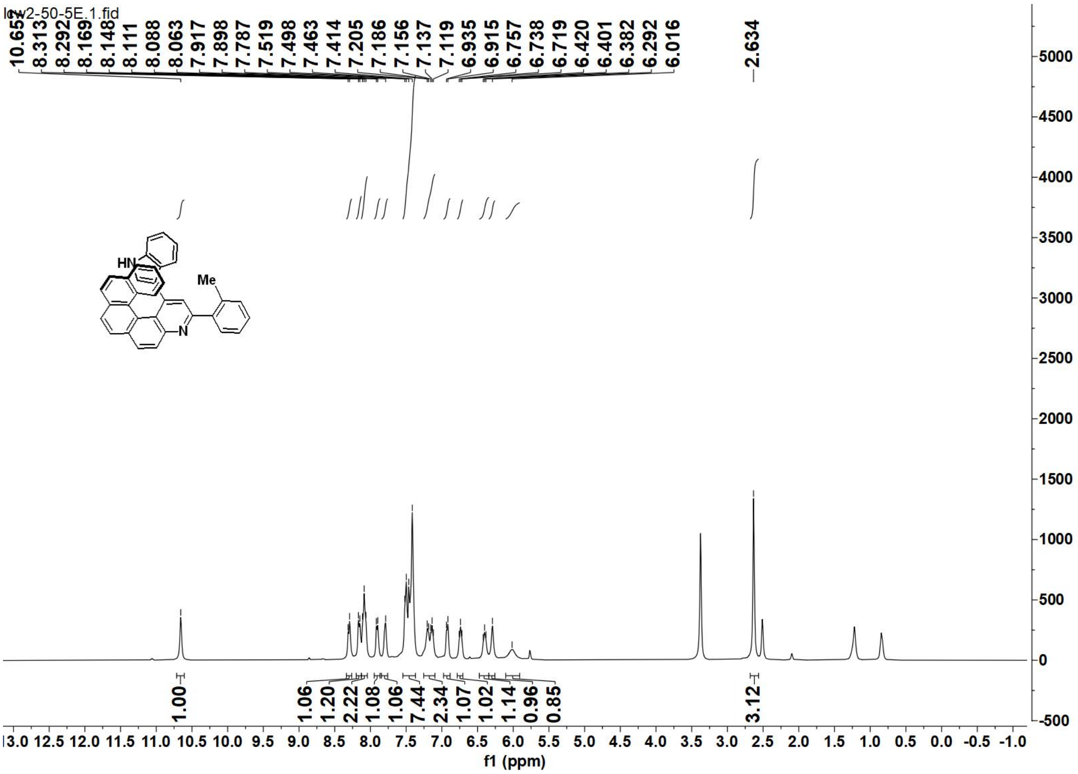

^{13}C NMR (101 MHz, DMSO-d_6) of **5o**

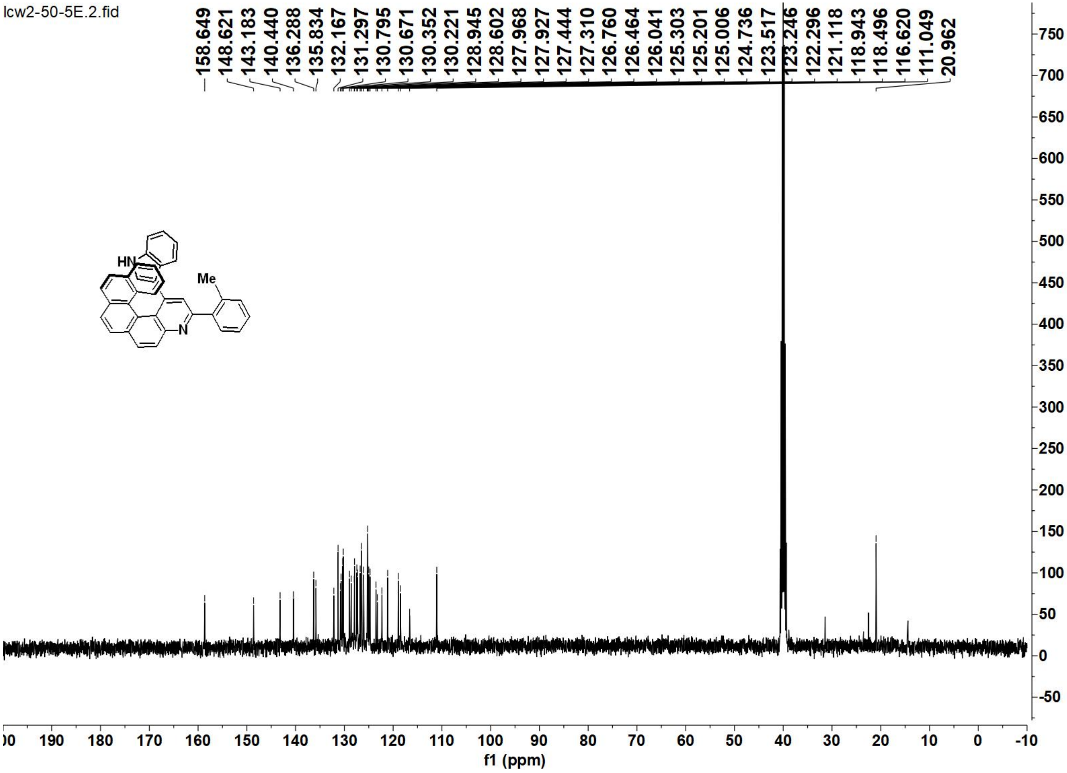

^{1}H NMR (400 MHz, DMSO-d_6) of **5p**

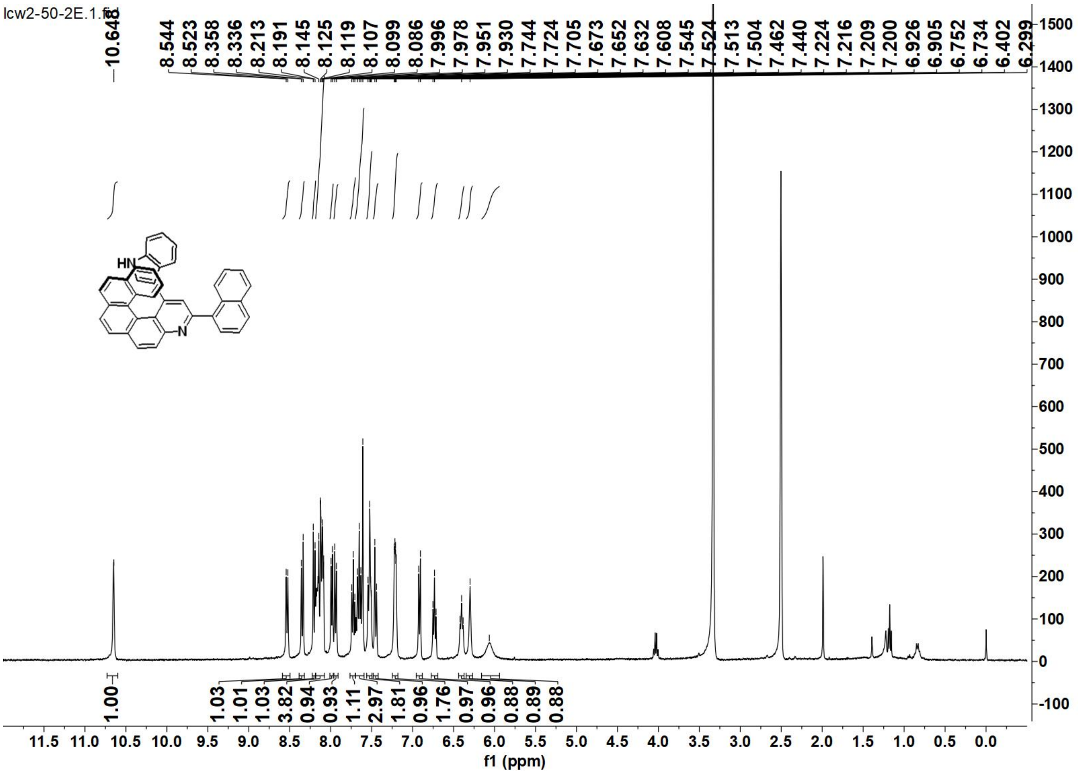

^{13}C NMR (101 MHz, DMSO-d_6) of **5p**

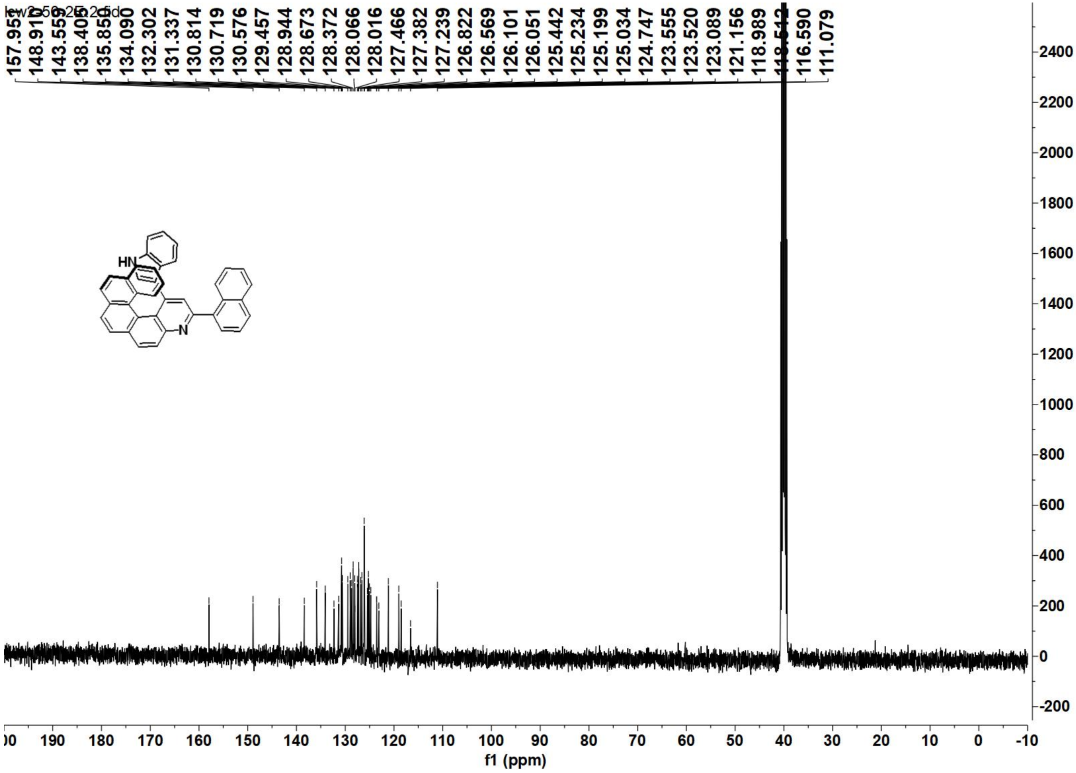

^{1}H NMR (400 MHz, DMSO-d_6) of **5q**

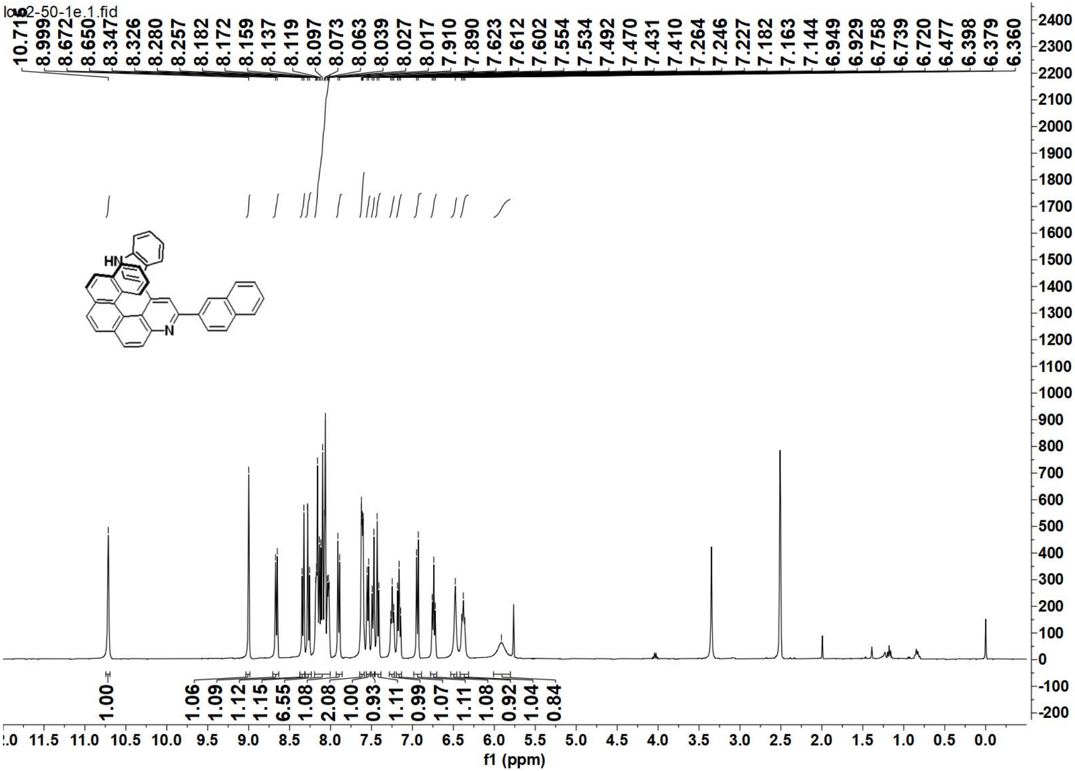

^{13}C NMR (101 MHz, DMSO-d_6) of **5q**

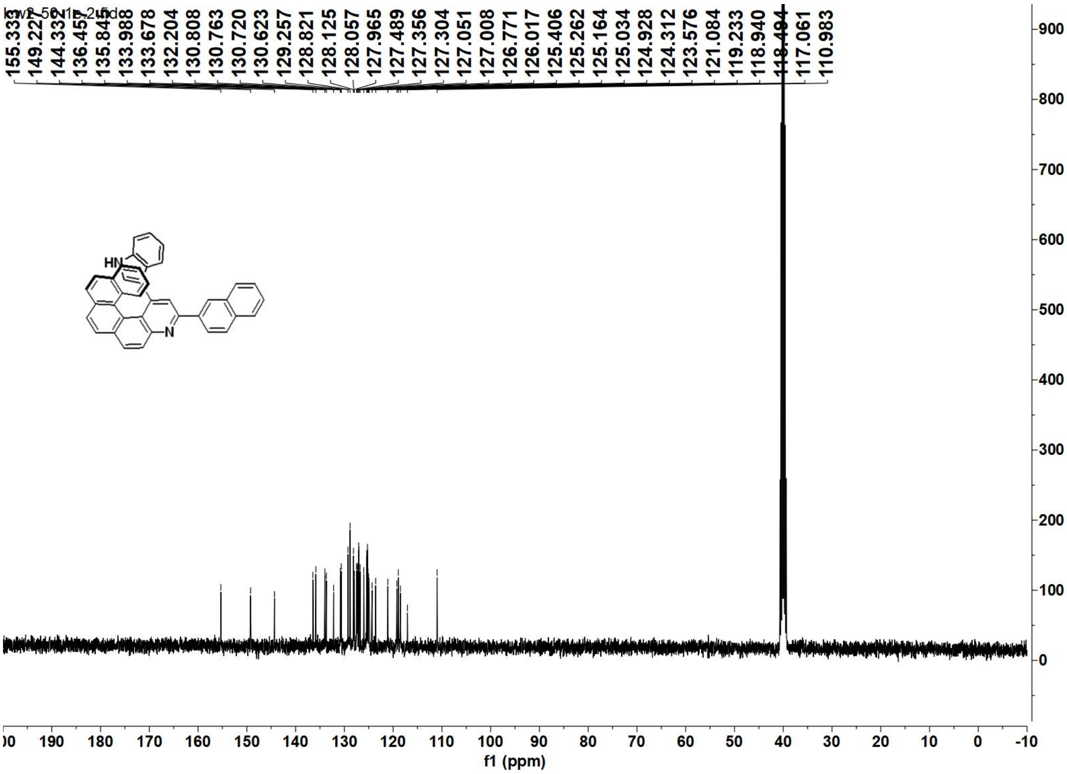

^{1}H NMR (400 MHz, DMSO-d_6) of **5r**

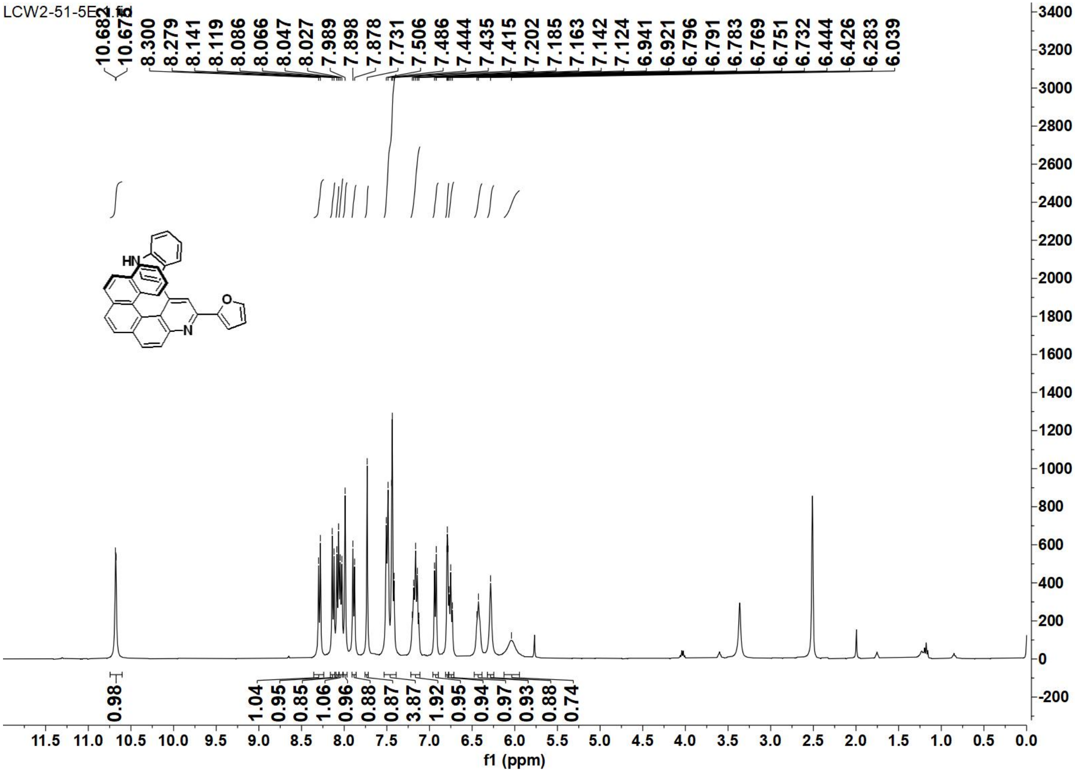

^{13}C NMR (101 MHz, DMSO-d_6) of **5r**

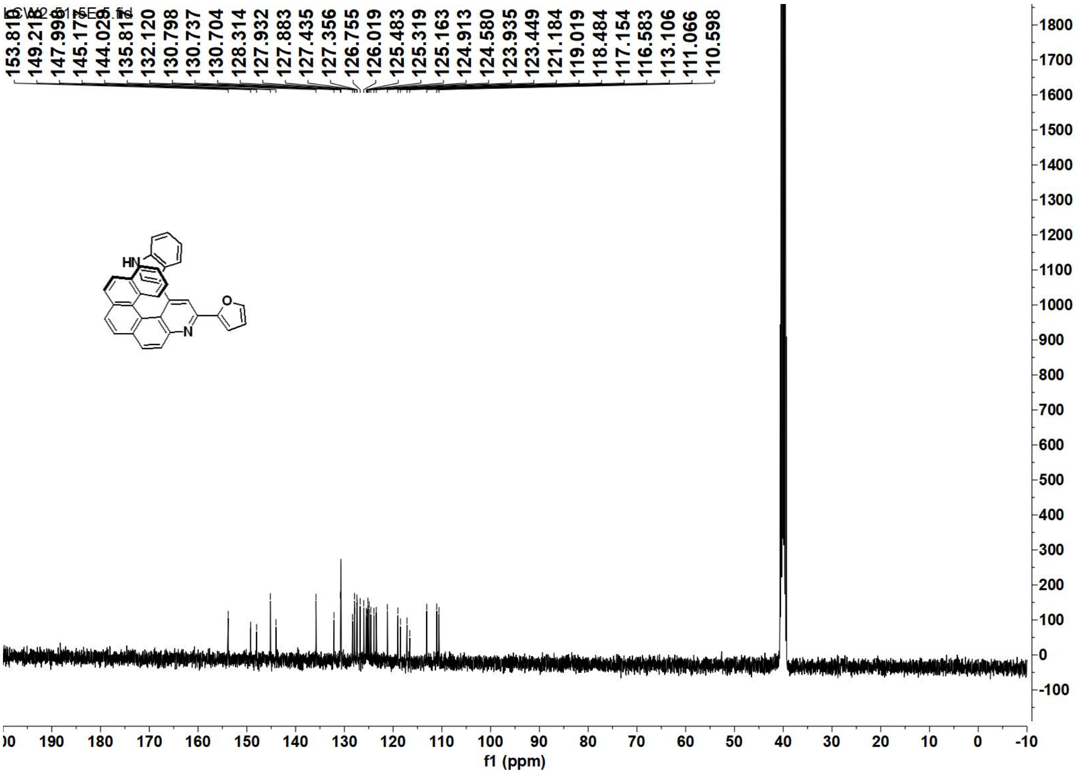

^1H NMR (400 MHz, DMSO-d_6) of **5s**

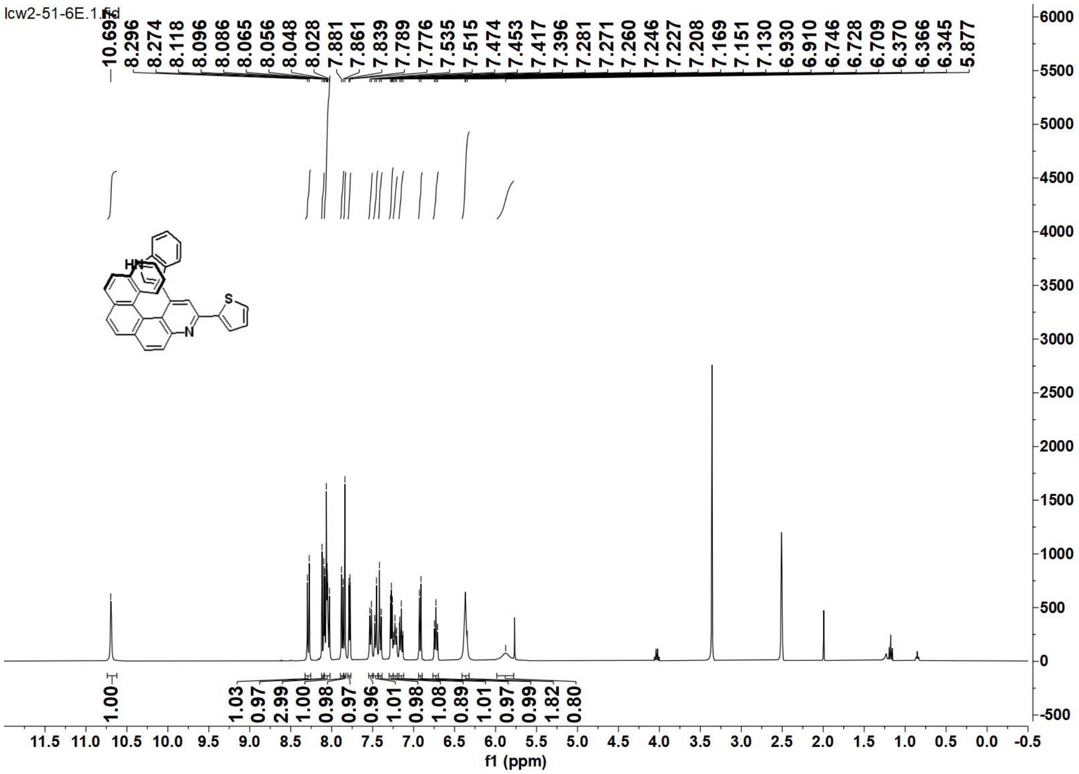

^{13}C NMR (101 MHz, DMSO-d_6) of **5s**

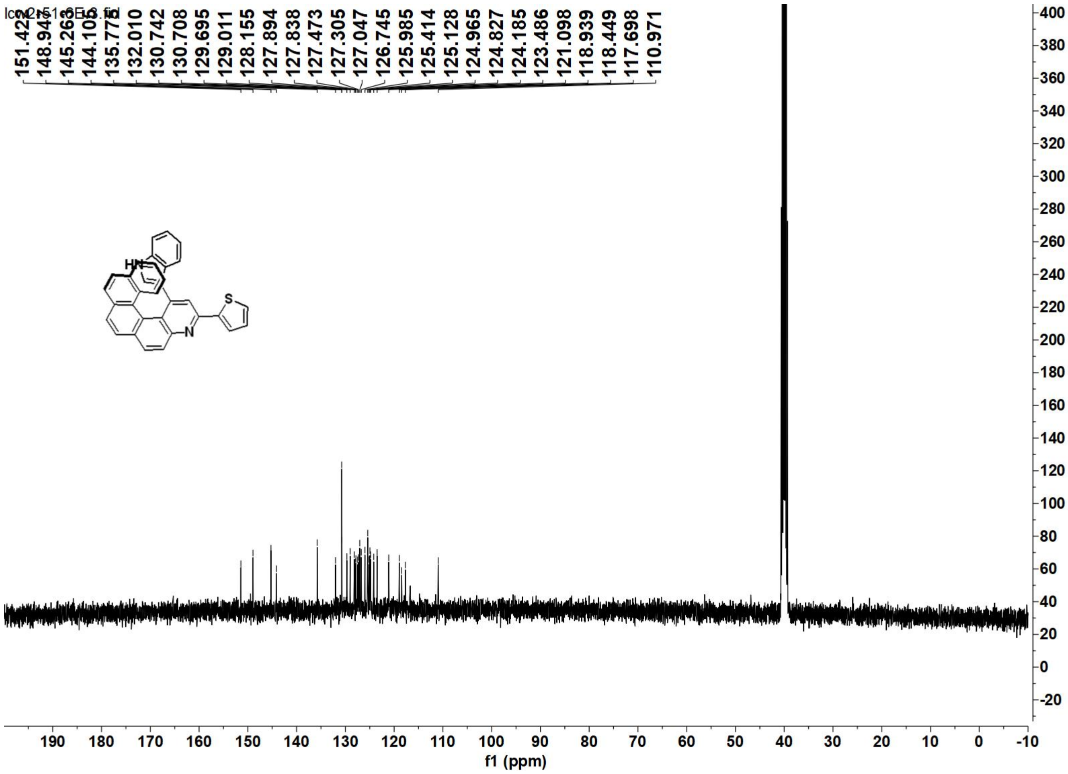

^{1}H NMR (400 MHz, DMSO-d_6) of **5t**

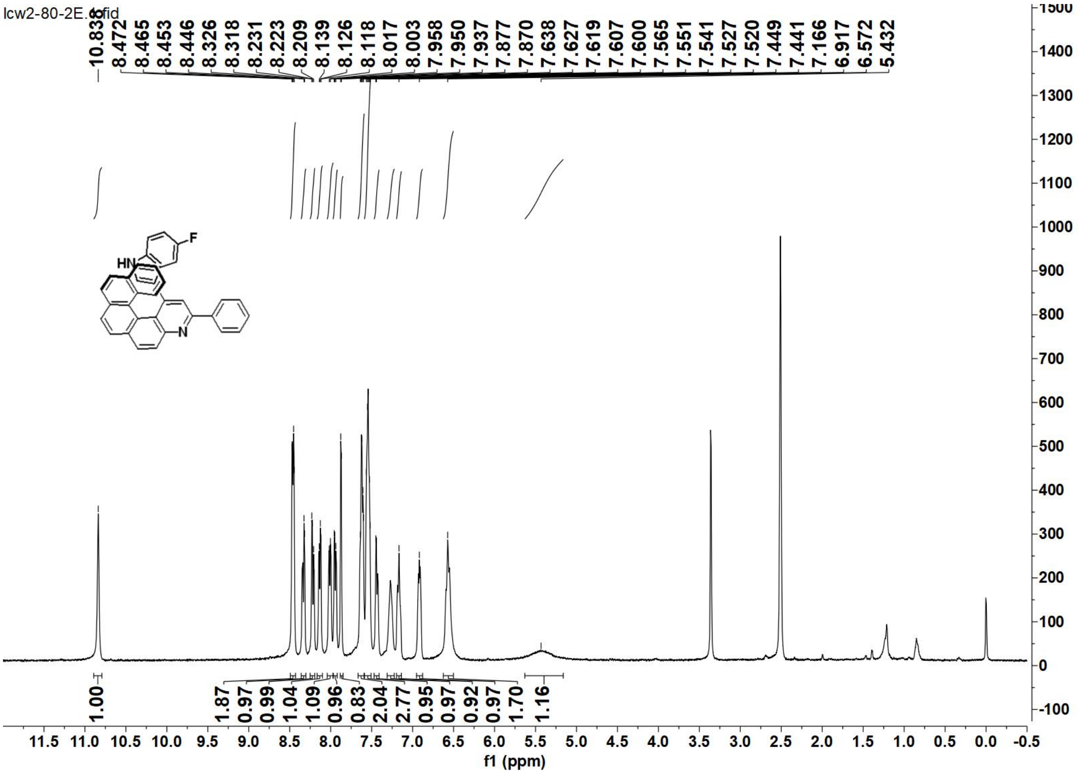

^{13}C NMR (101 MHz, DMSO-d_6) of **5t**

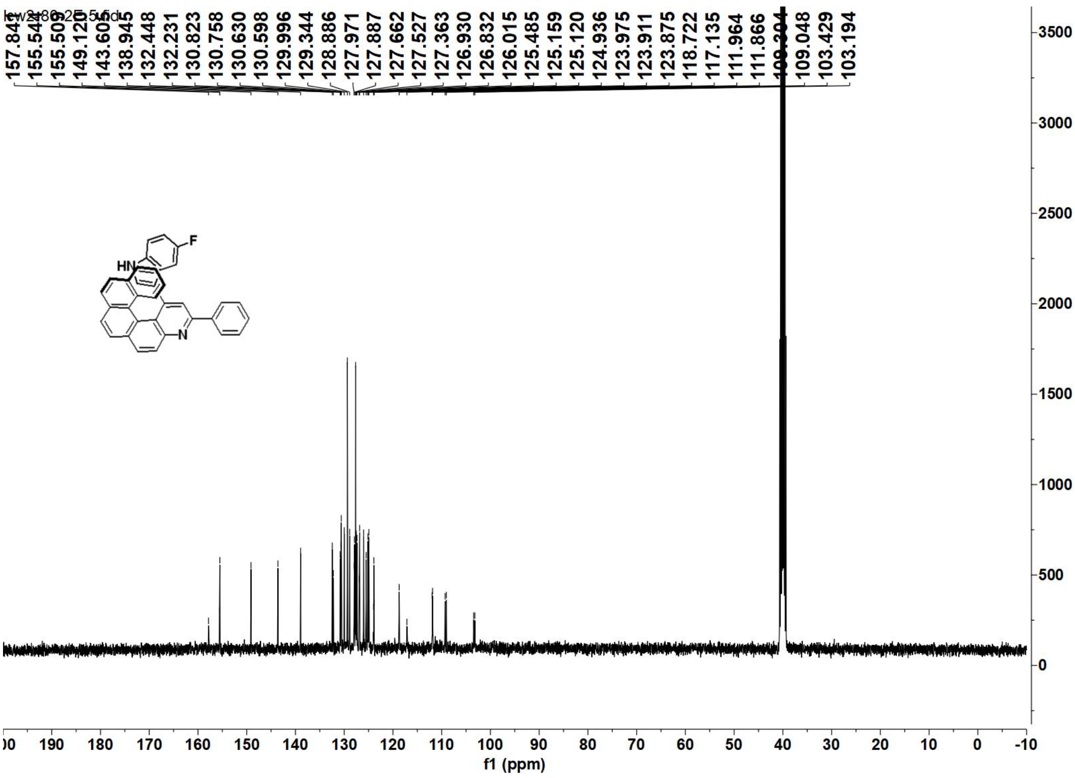

^{19}F NMR (376 MHz, DMSO-d_6) of **5t**

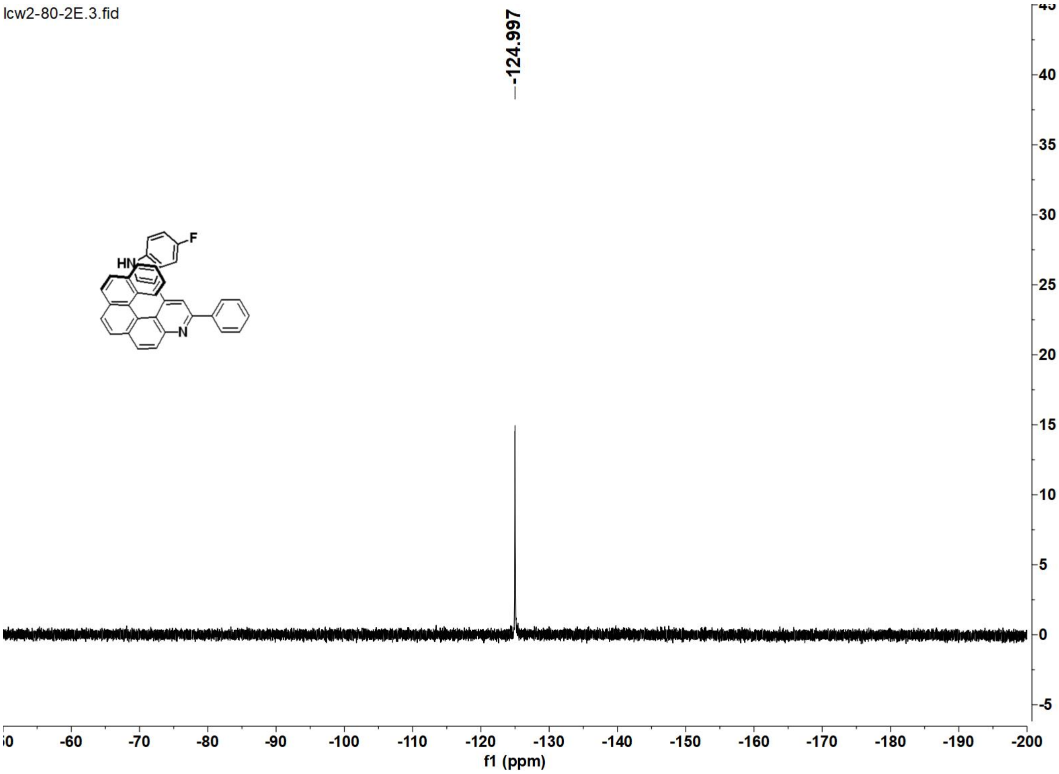

^1H NMR (400 MHz, DMSO-d_6) of **5u**

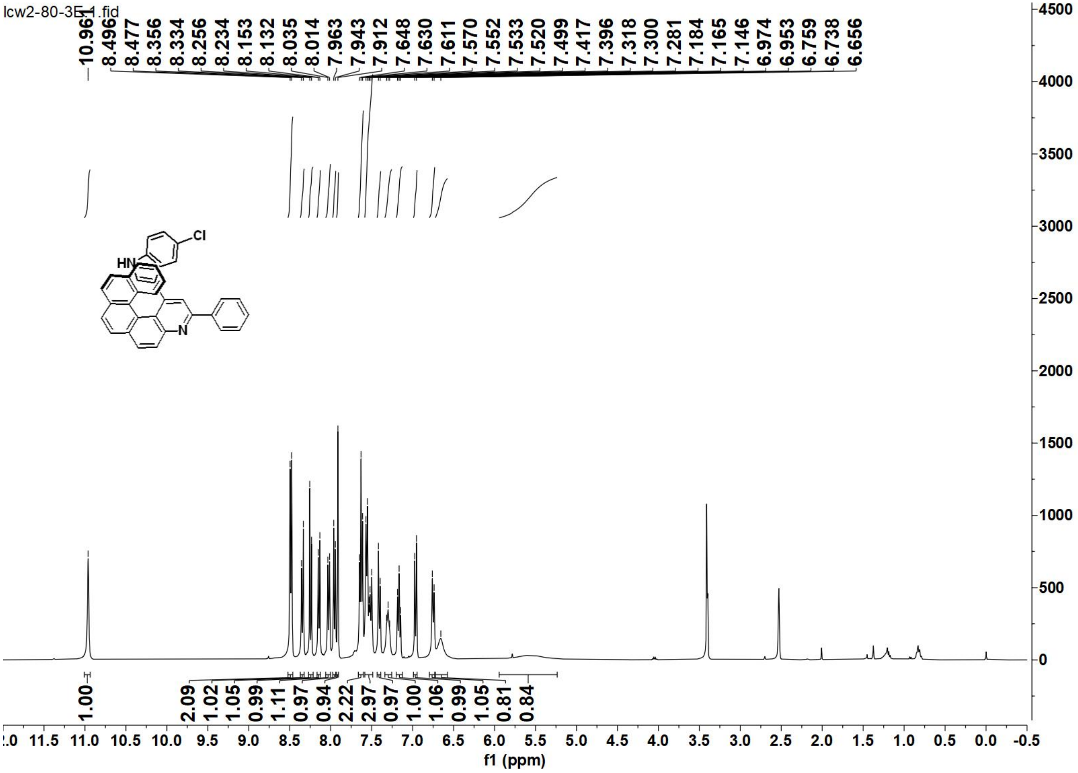

^{13}C NMR (101 MHz, DMSO-d_6) of **5u**

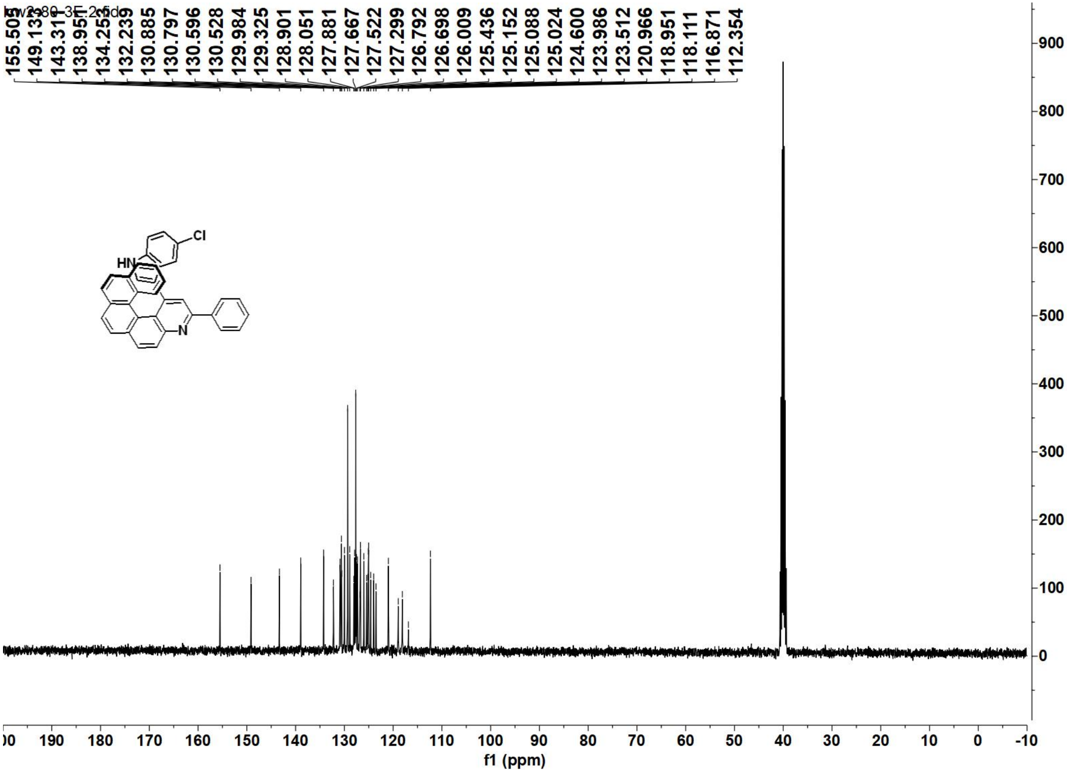

^{1}H NMR (400 MHz, DMSO-d_6) of **5v**

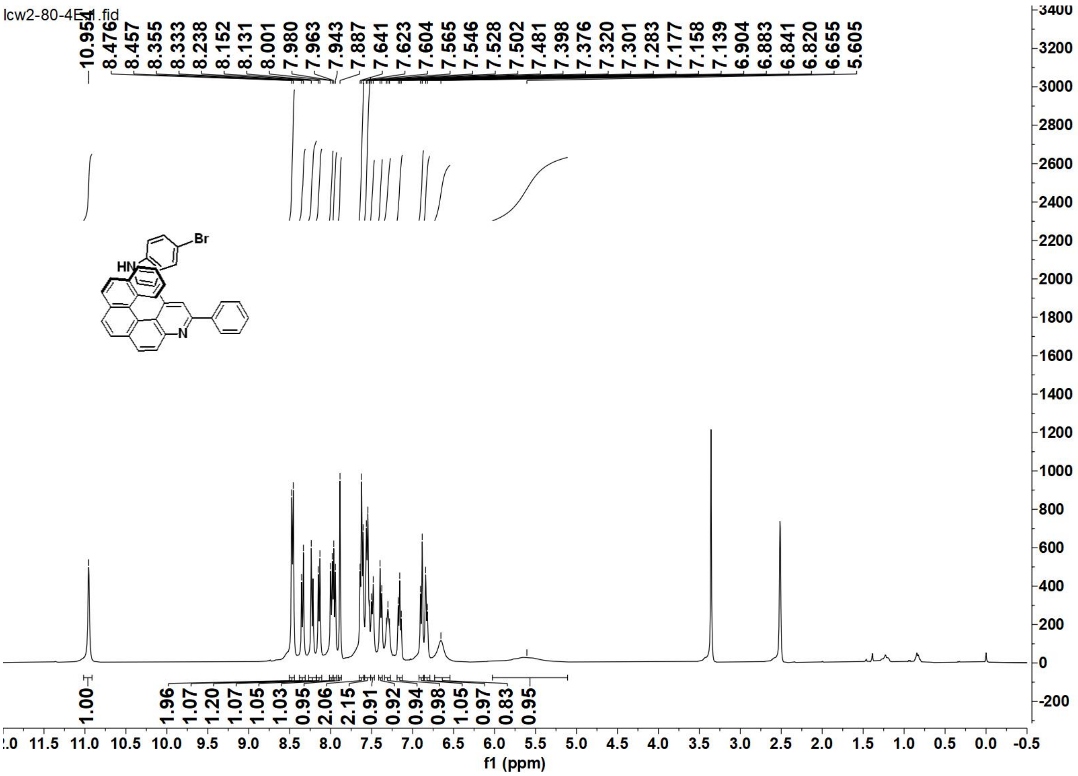

^{13}C NMR (101 MHz, DMSO-d_6) of **5v**

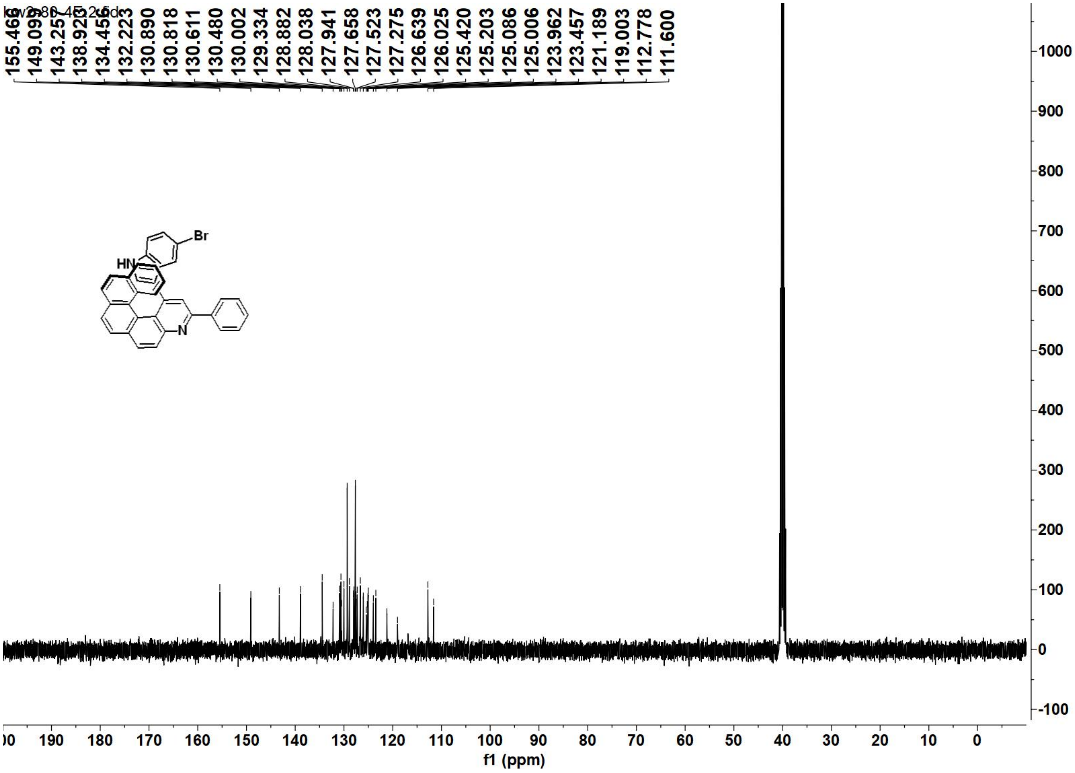

^{1}H NMR (400 MHz, DMSO-d_6) of **5w**

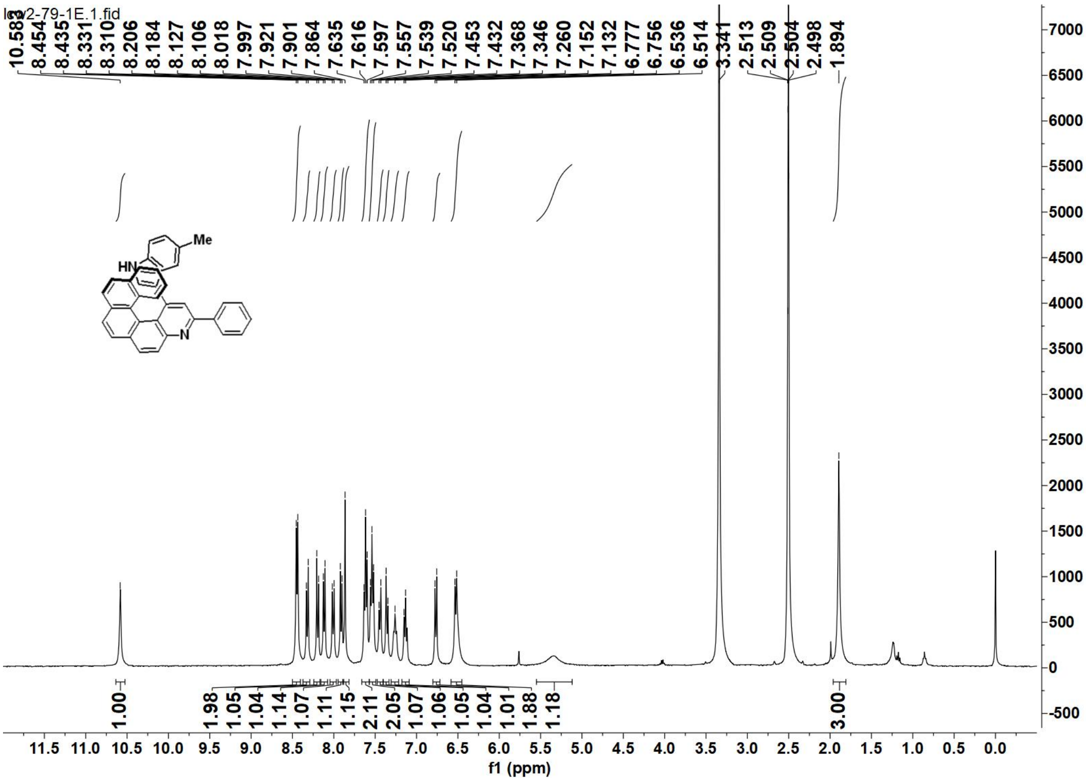

^{13}C NMR (101 MHz, DMSO-d_6) of **5w**

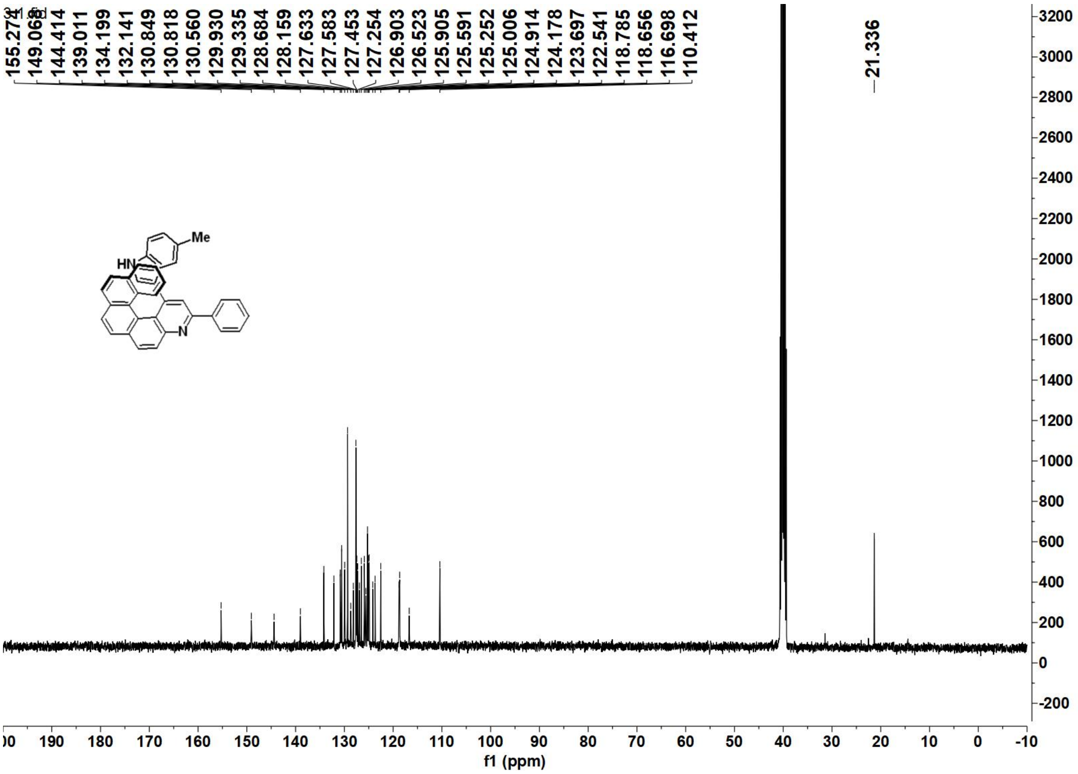

^{1}H NMR (400 MHz, DMSO-d_6) of **5ab**

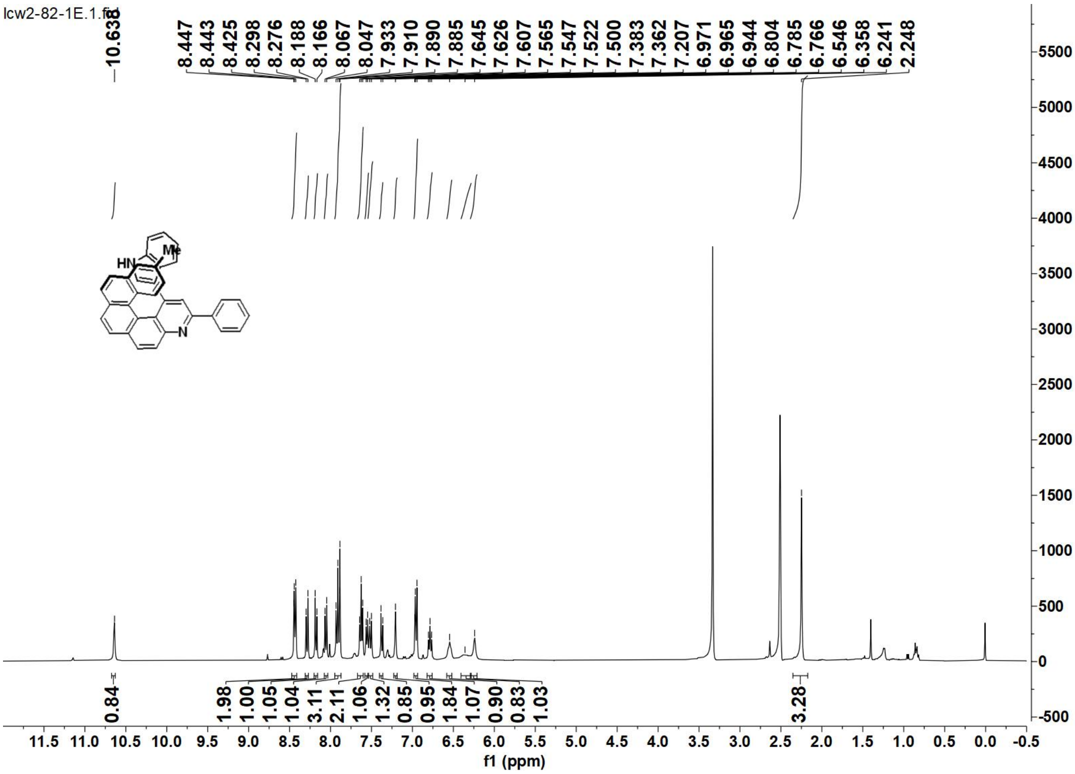

^{13}C NMR (101 MHz, DMSO-d_6) of **5ab**

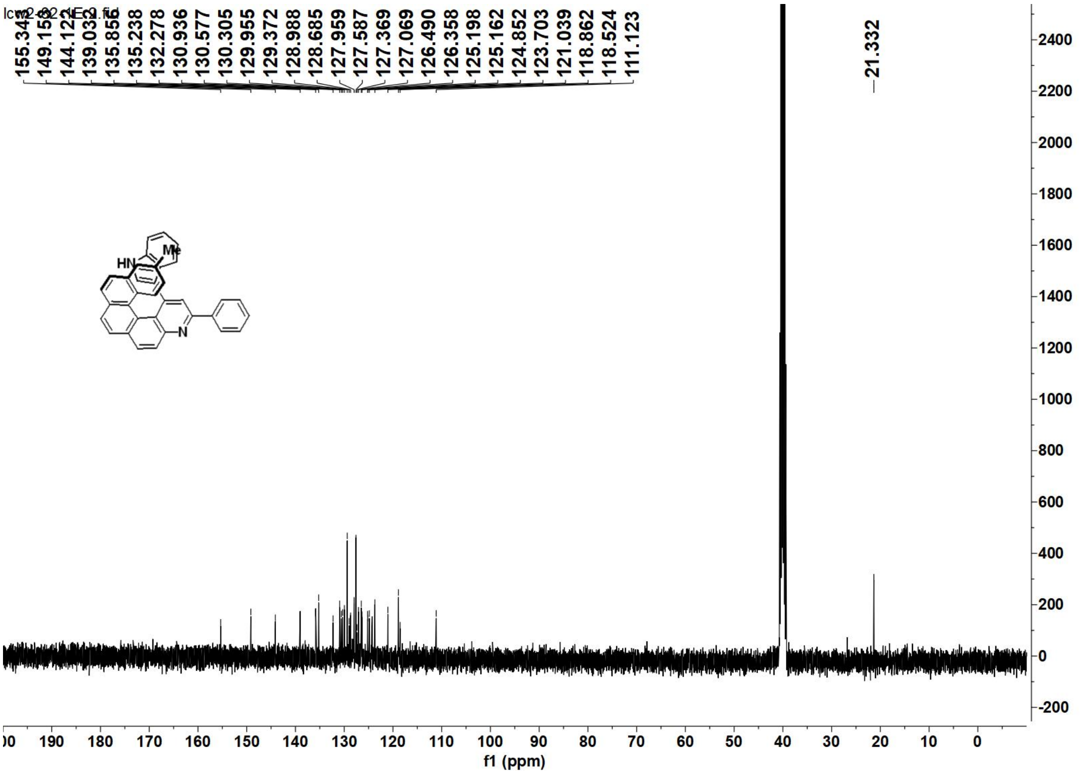

^{1}H NMR (400 MHz, DMSO-d_6) of **5ac**

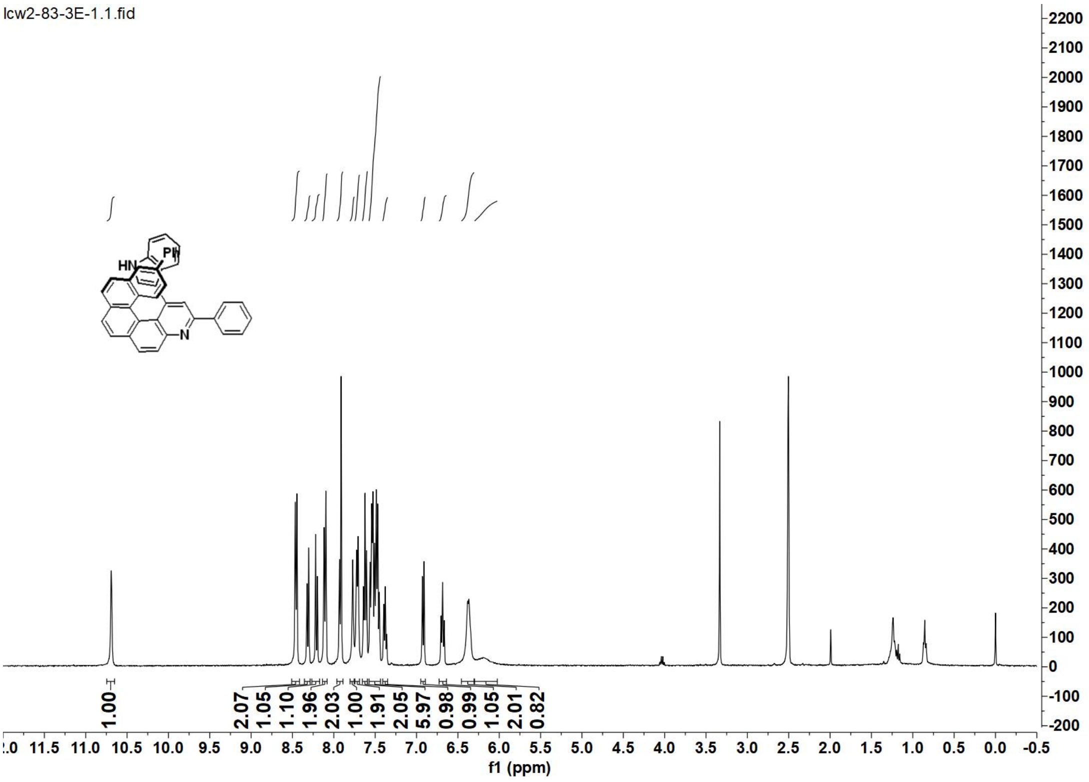

^{13}C NMR (101 MHz, DMSO-d_6) of **5ac**

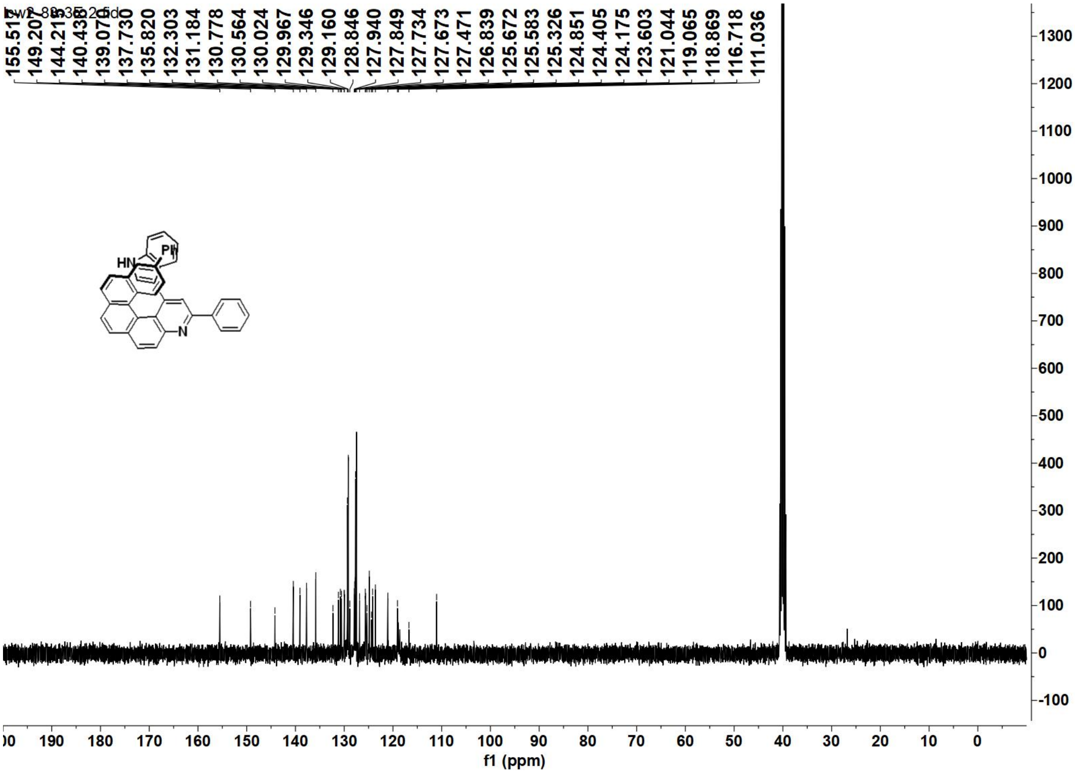

^{1}H NMR (400 MHz, DMSO-d_6) of **5ad**

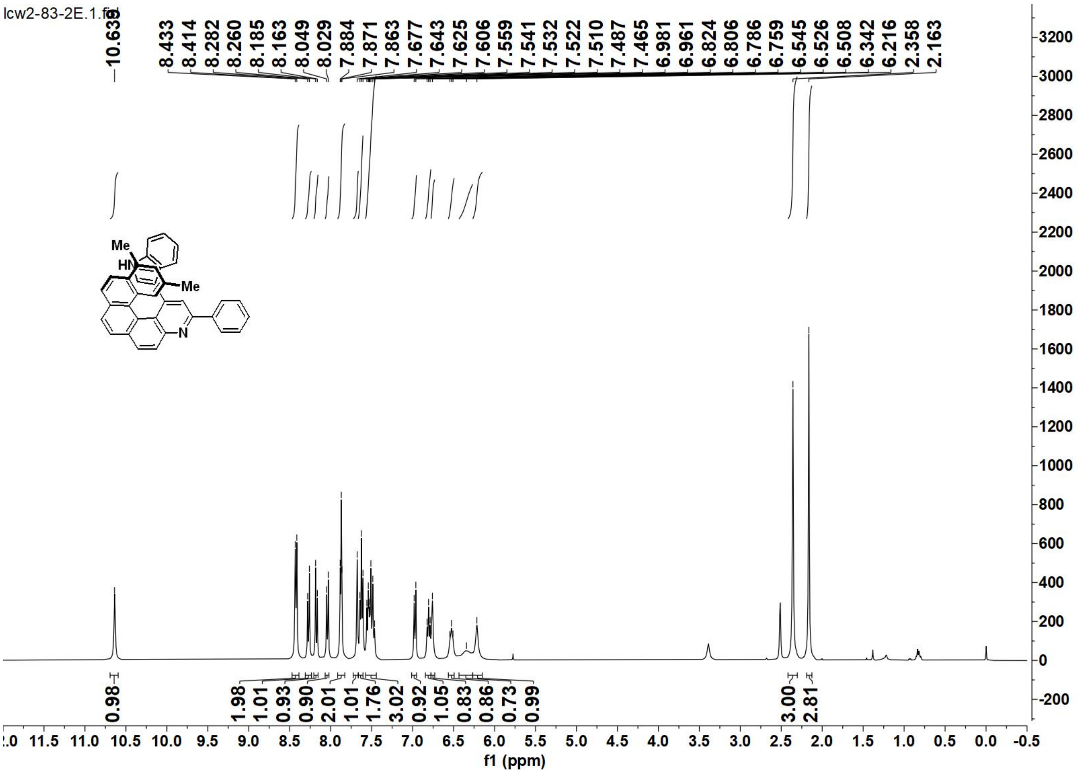

^{13}C NMR (101 MHz, DMSO-d_6) of **5ad**

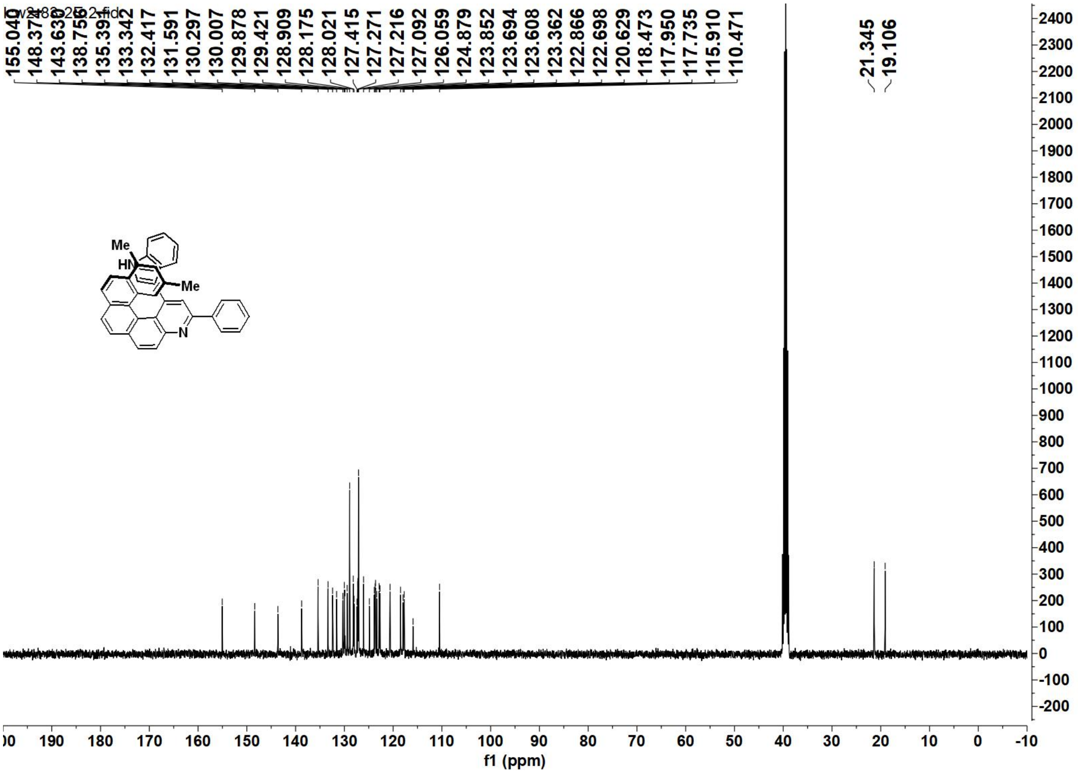

^{1}H NMR (400 MHz, DMSO-d_6) of **5ae**

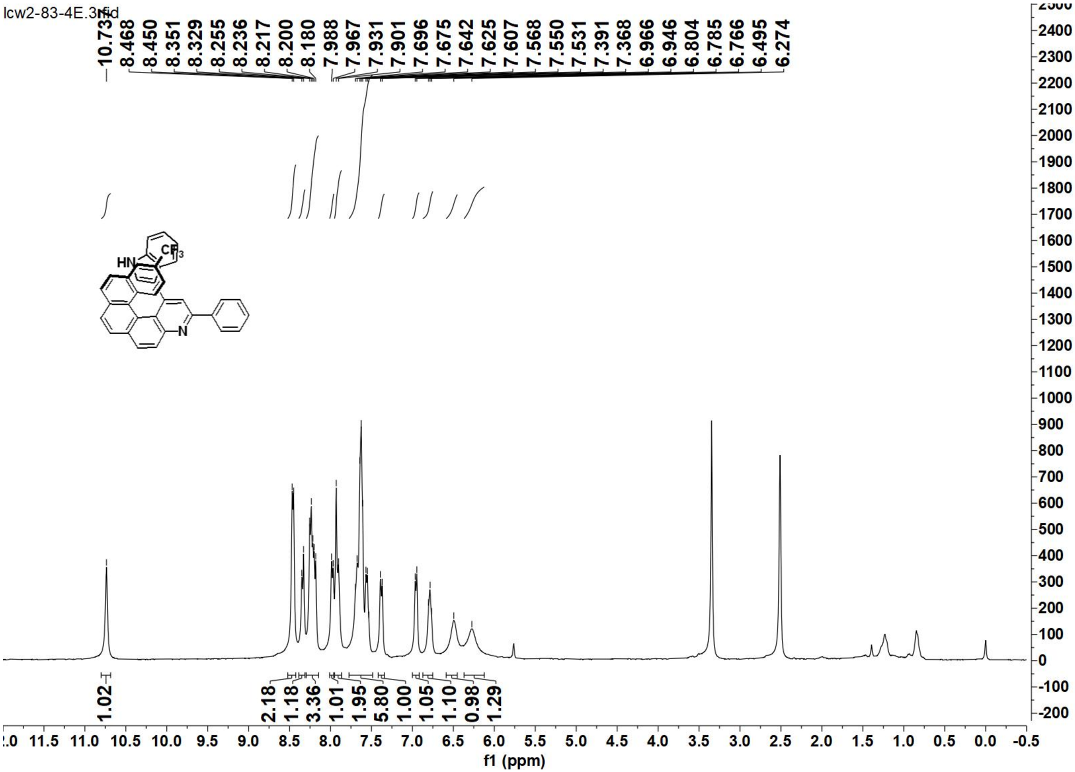

^{13}C NMR (101 MHz, DMSO-d_6) of **5ae**

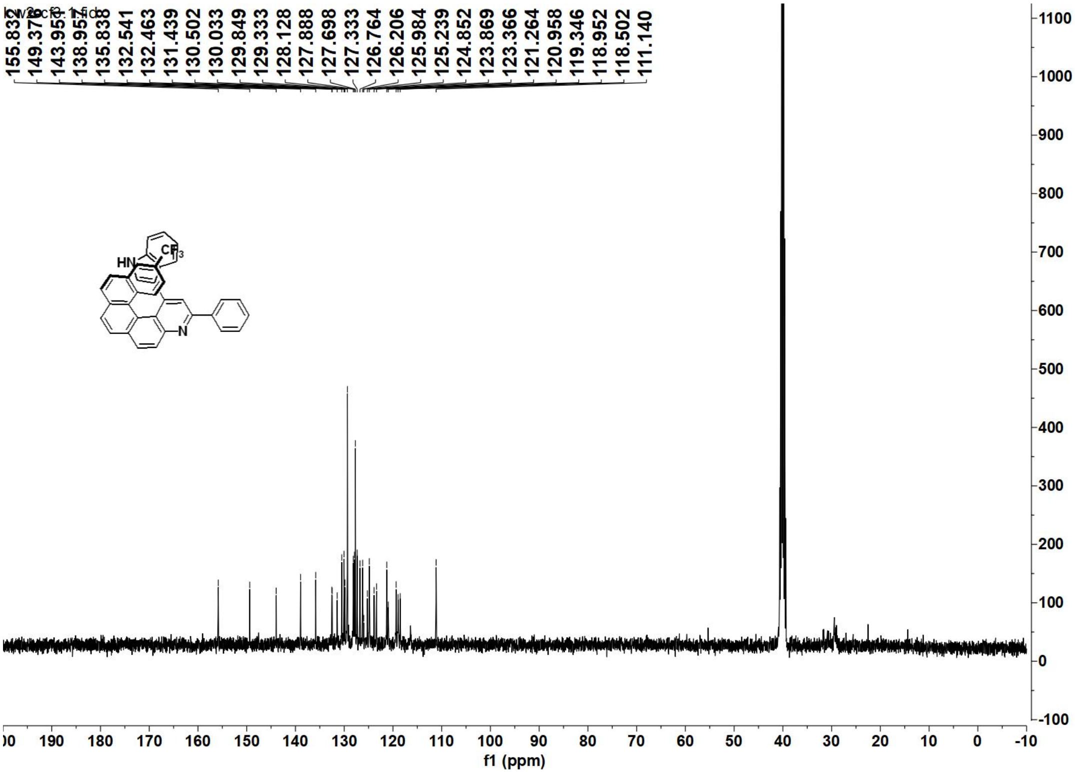

^{19}F NMR (376 MHz, DMSO-d_6) of **5ae**

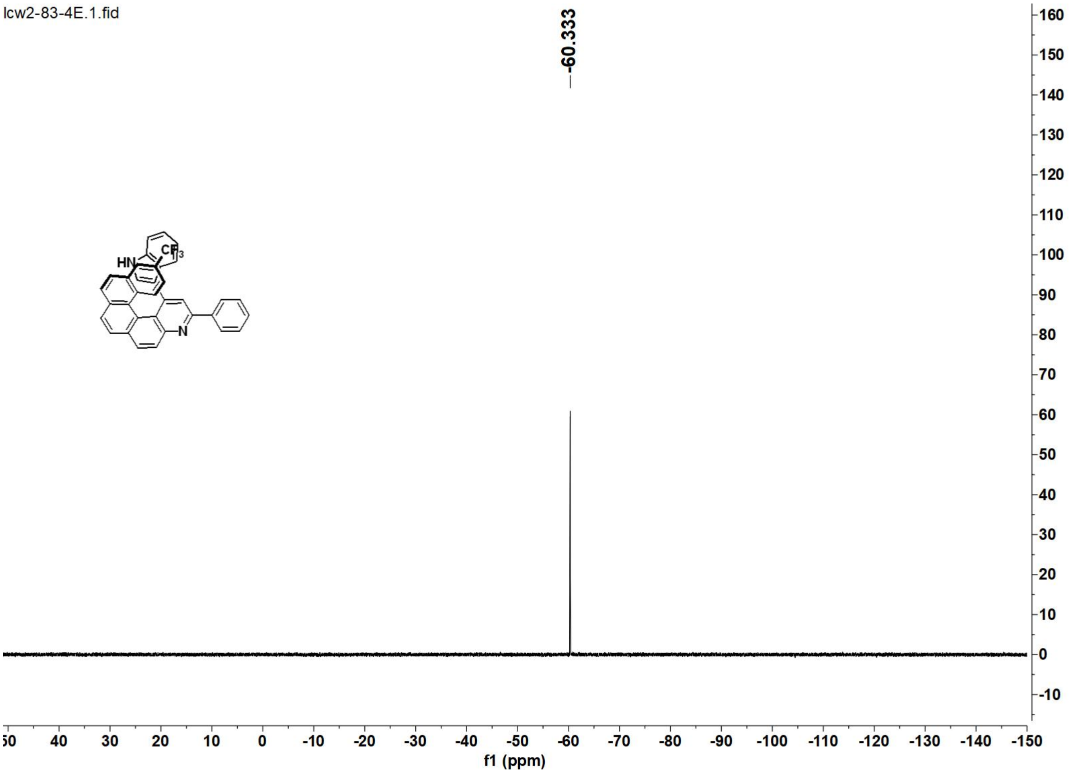

^{1}H NMR (400 MHz, DMSO-d_6) of **5af**

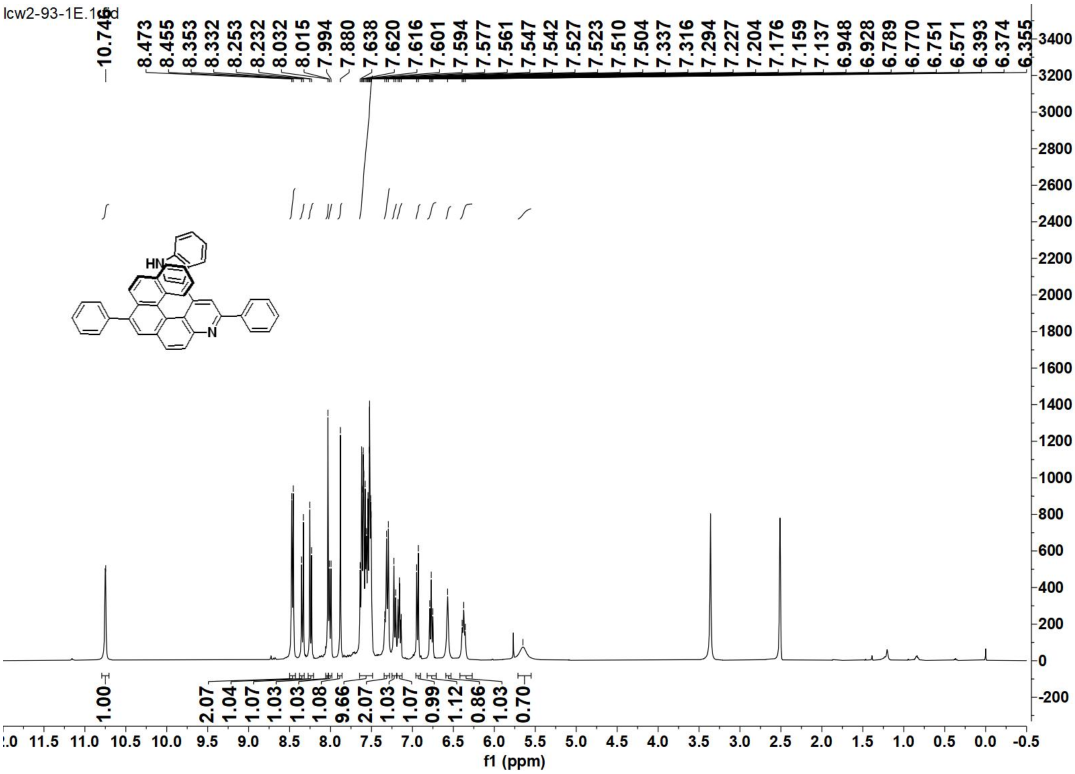

^{13}C NMR (101 MHz, DMSO-d_6) of **5af**

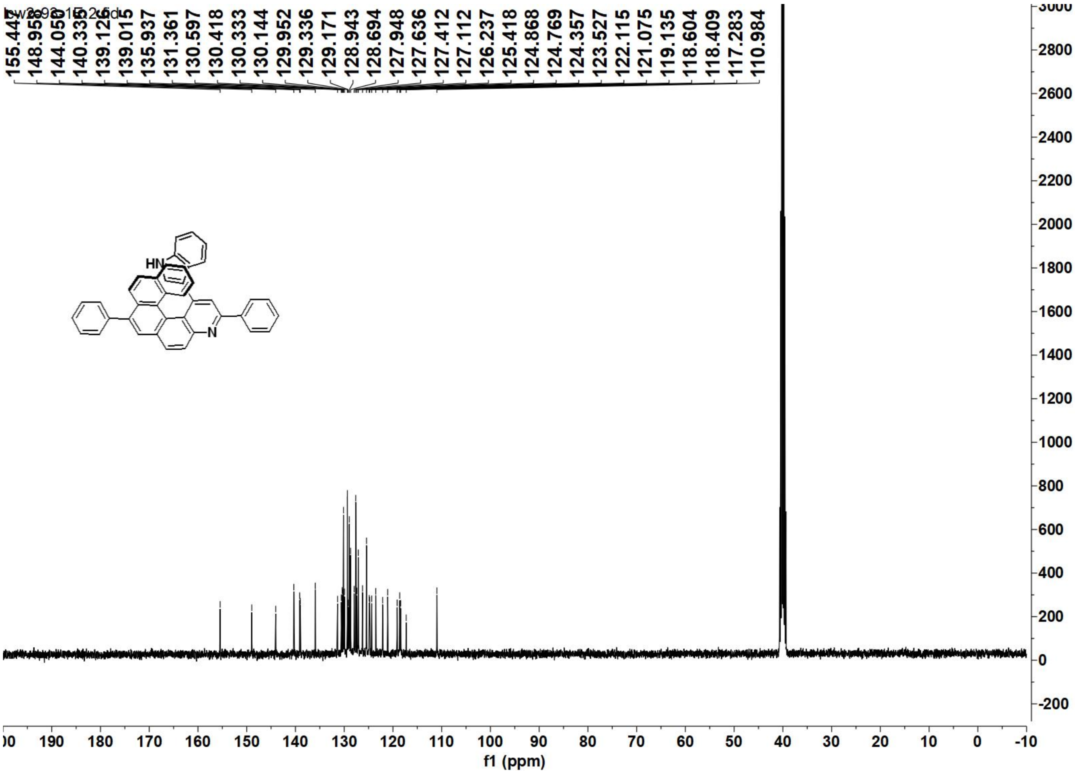

^{1}H NMR (400 MHz, DMSO-d_6) of **5ag**

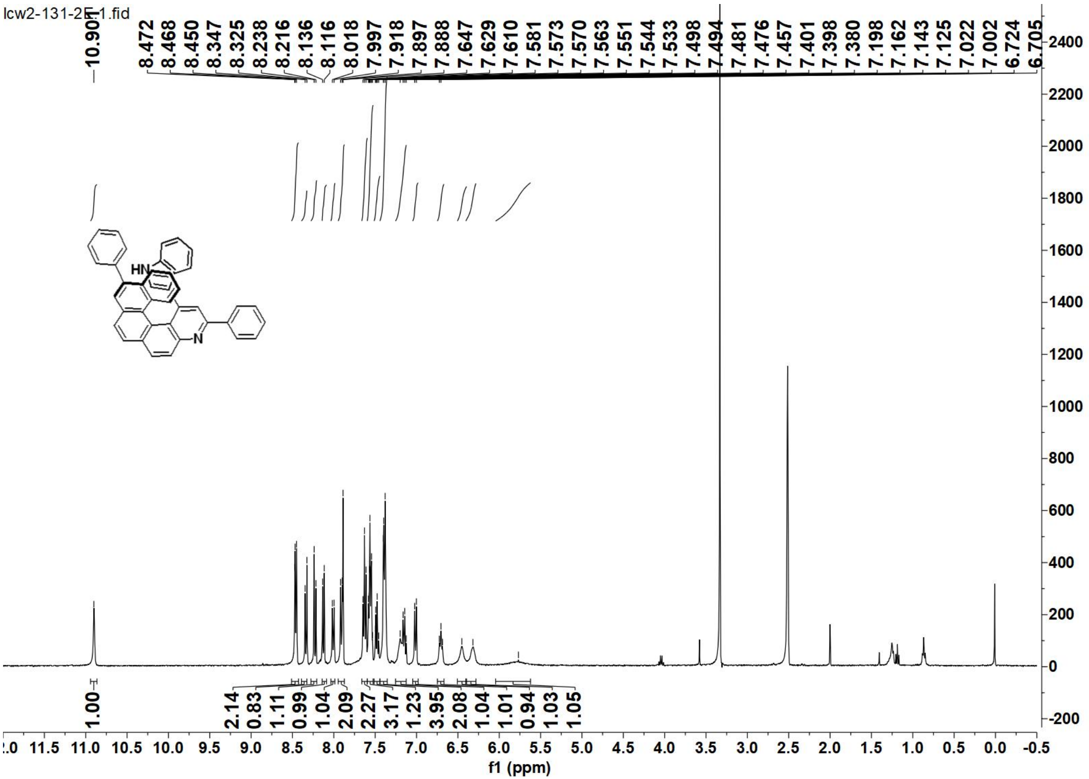

^{13}C NMR (101 MHz, DMSO-d_6) of **5ag**

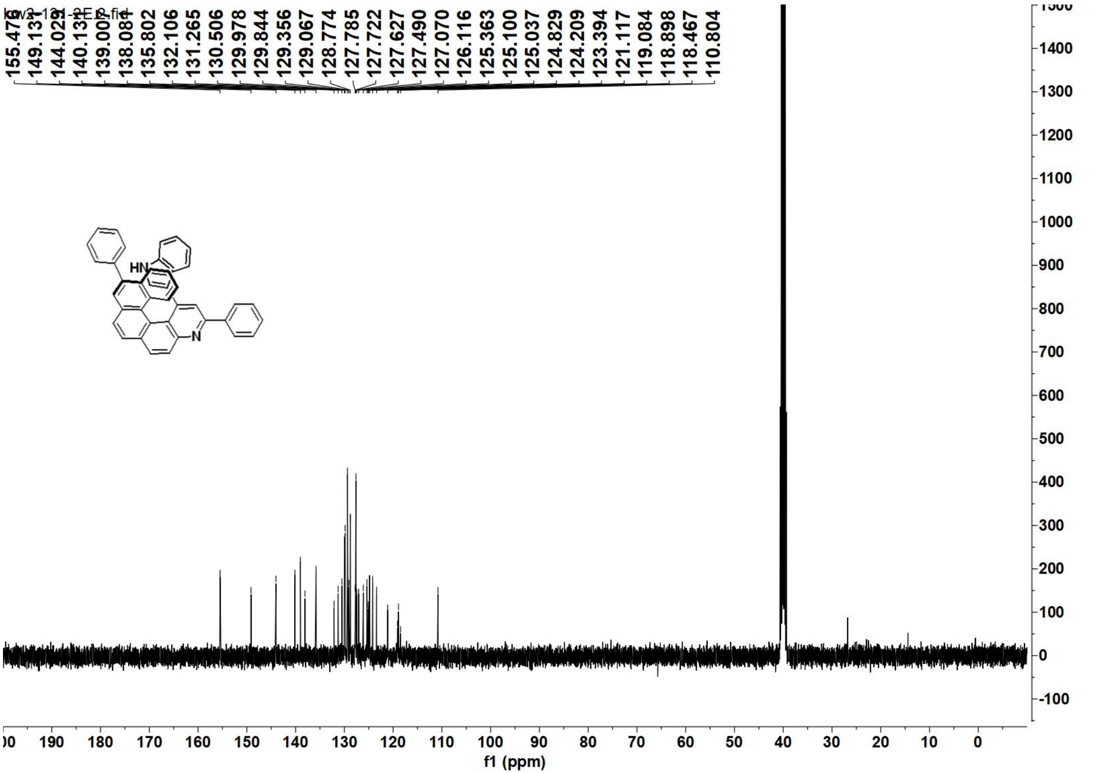

^1H NMR (400 MHz, DMSO-d_6) of **5ah**

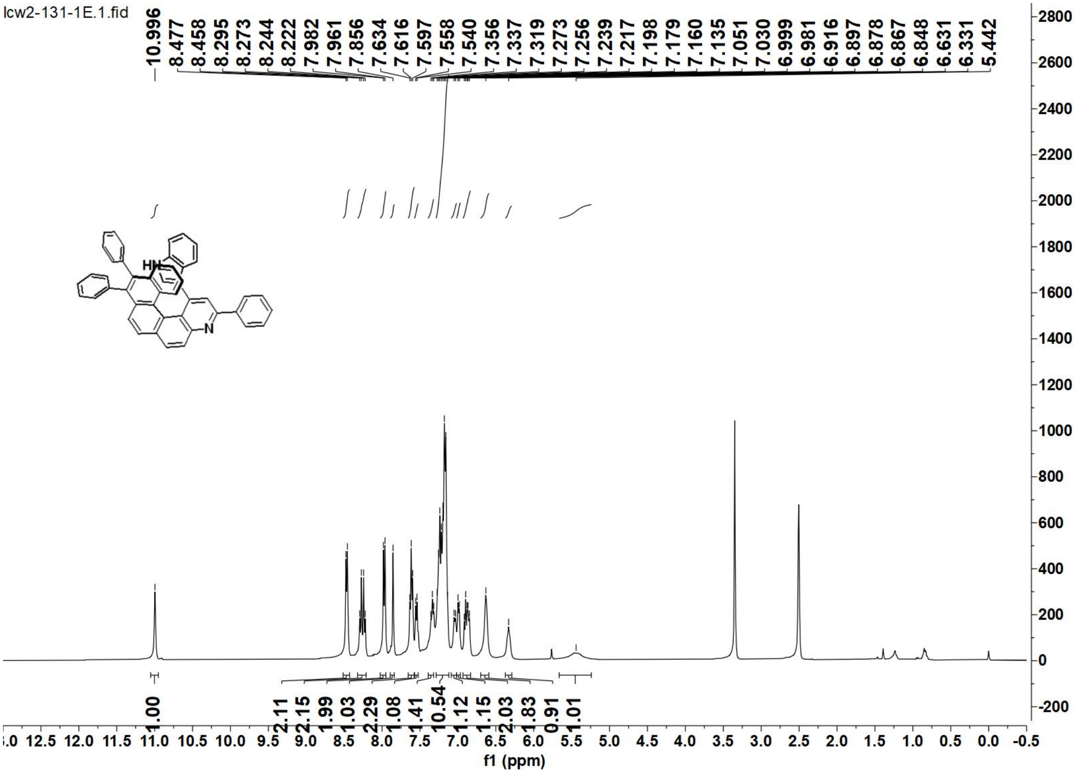

^{13}C NMR (101 MHz, DMSO-d_6) of **5ah**

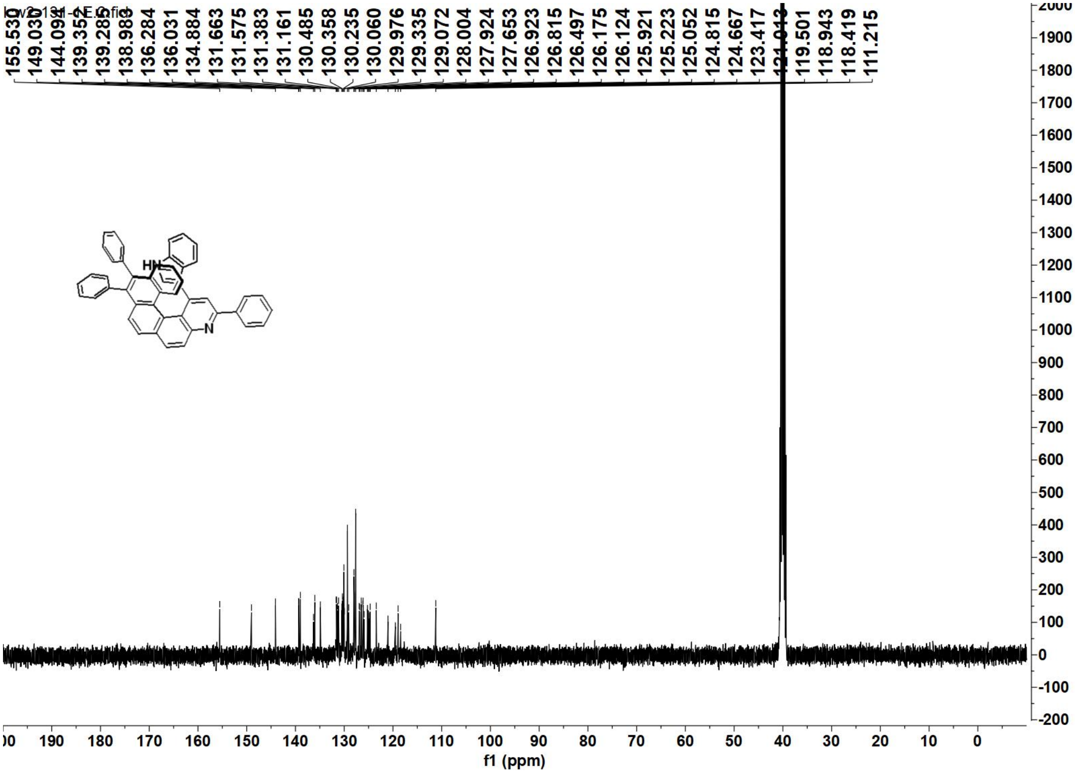

^{1}H NMR (400 MHz, DMSO-d_6) of **5ai**

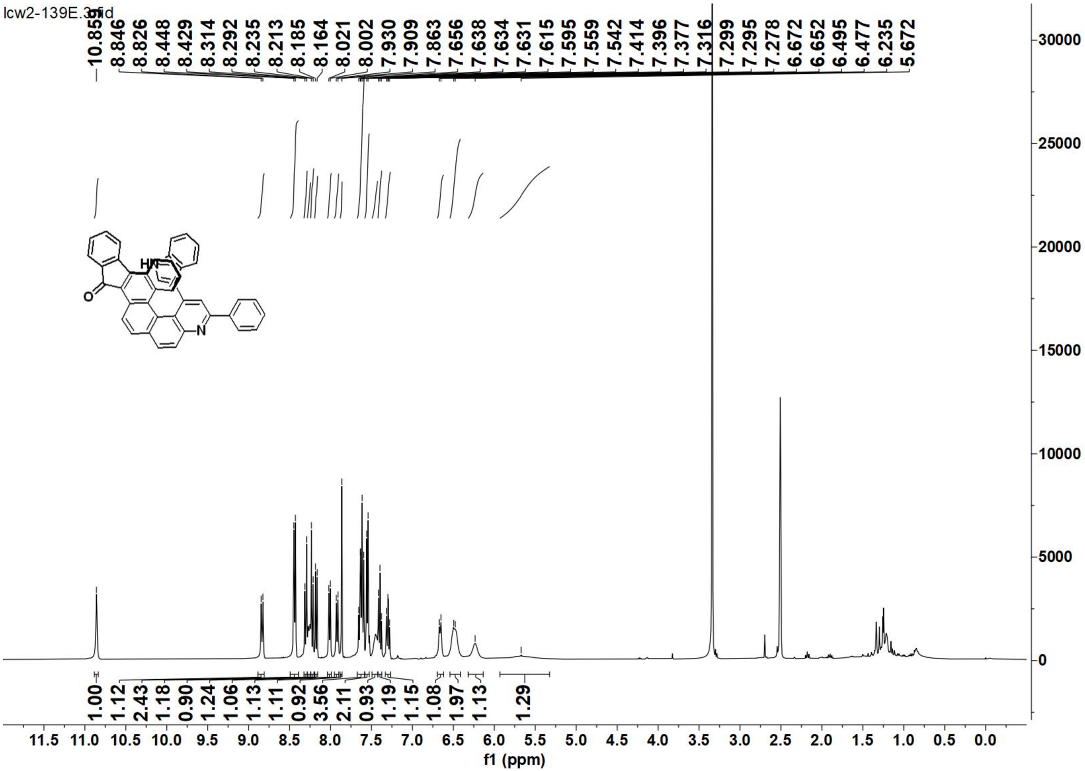

^{13}C NMR (101 MHz, DMSO-d_6) of **5ai**

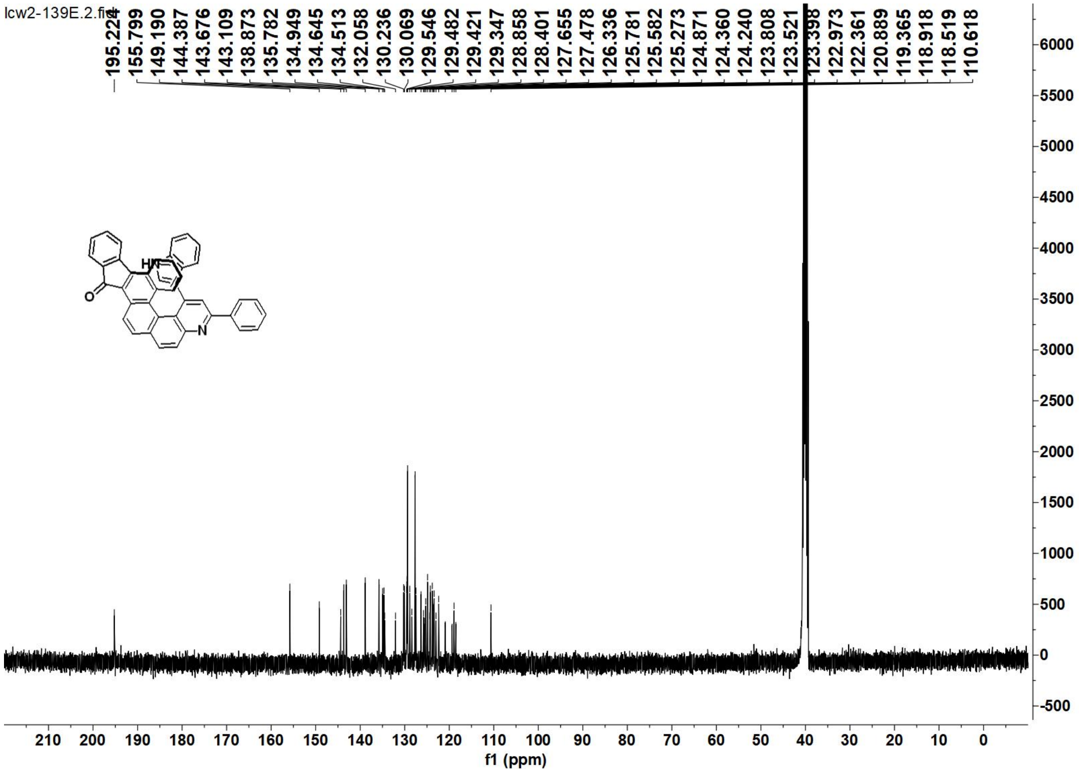

^{1}H NMR (400 MHz, DMSO-d_6) of **5aj**

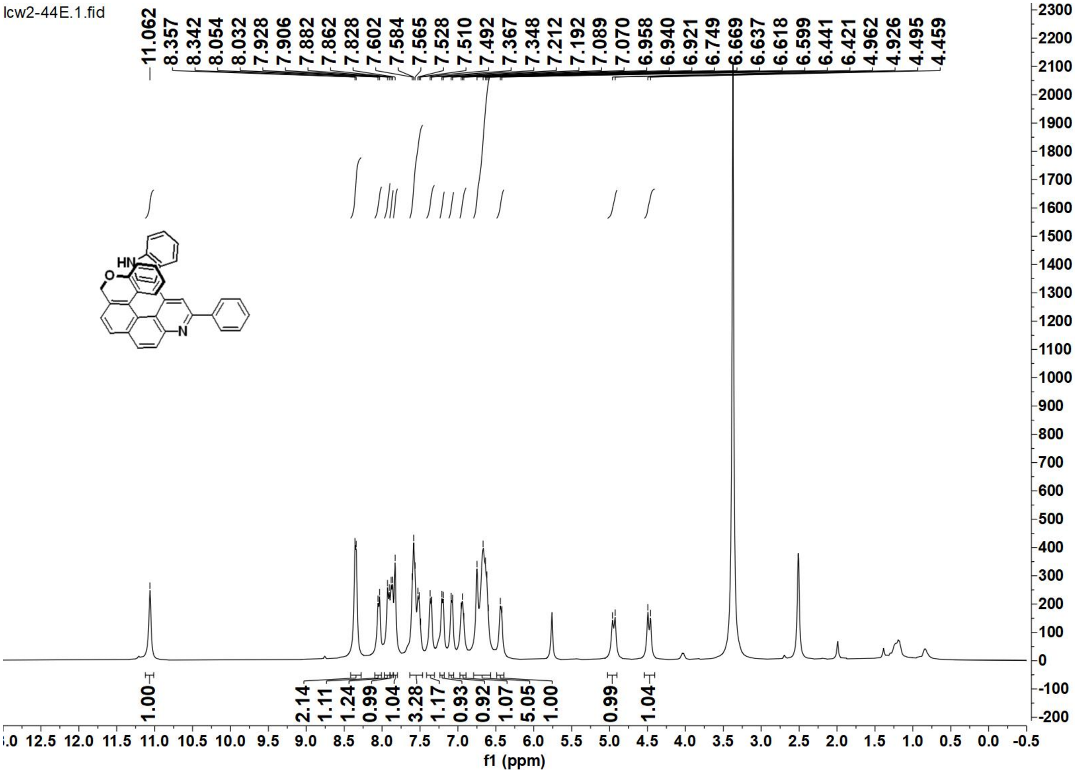

^{13}C NMR (101 MHz, DMSO-d_6) of **5aj**

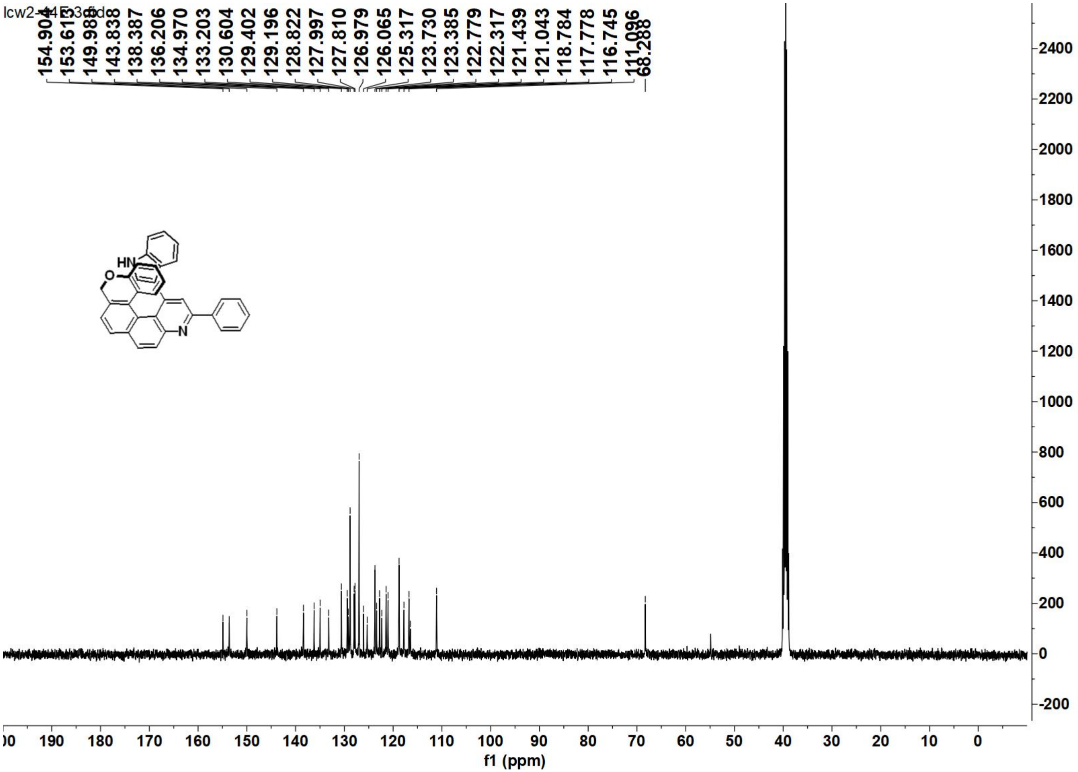

^{1}H NMR (400 MHz, DMSO-d_6) of **5ak**

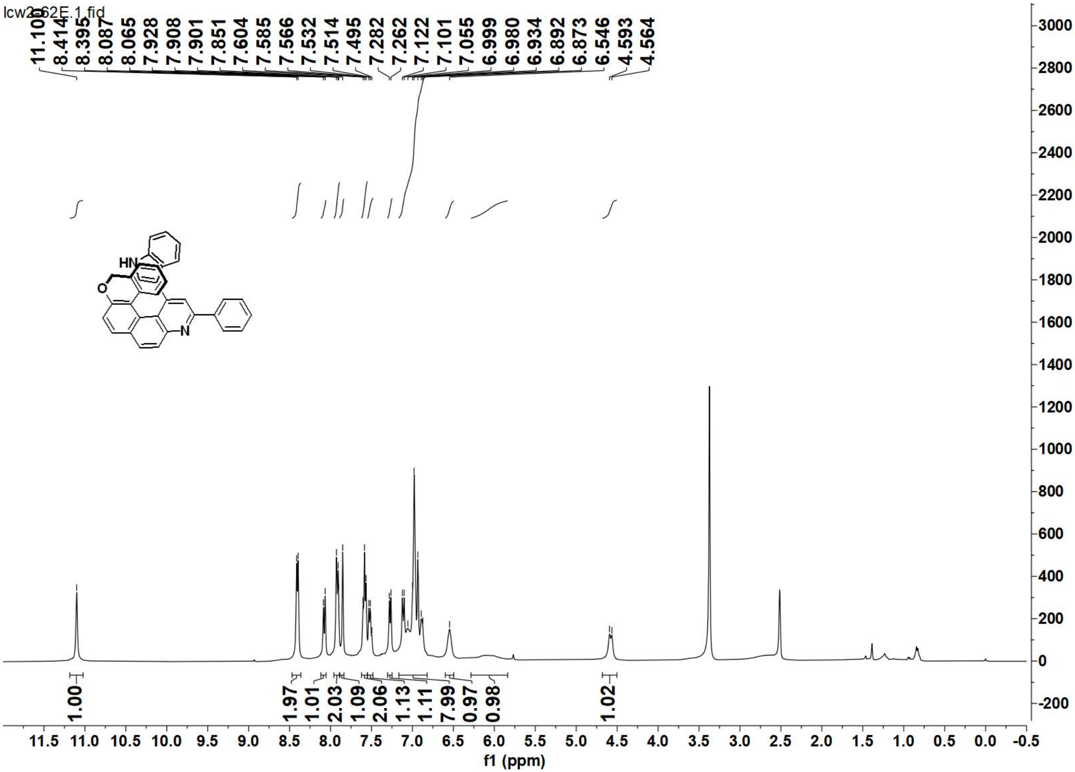

^{13}C NMR (101 MHz, DMSO-d_6) of **5ak**

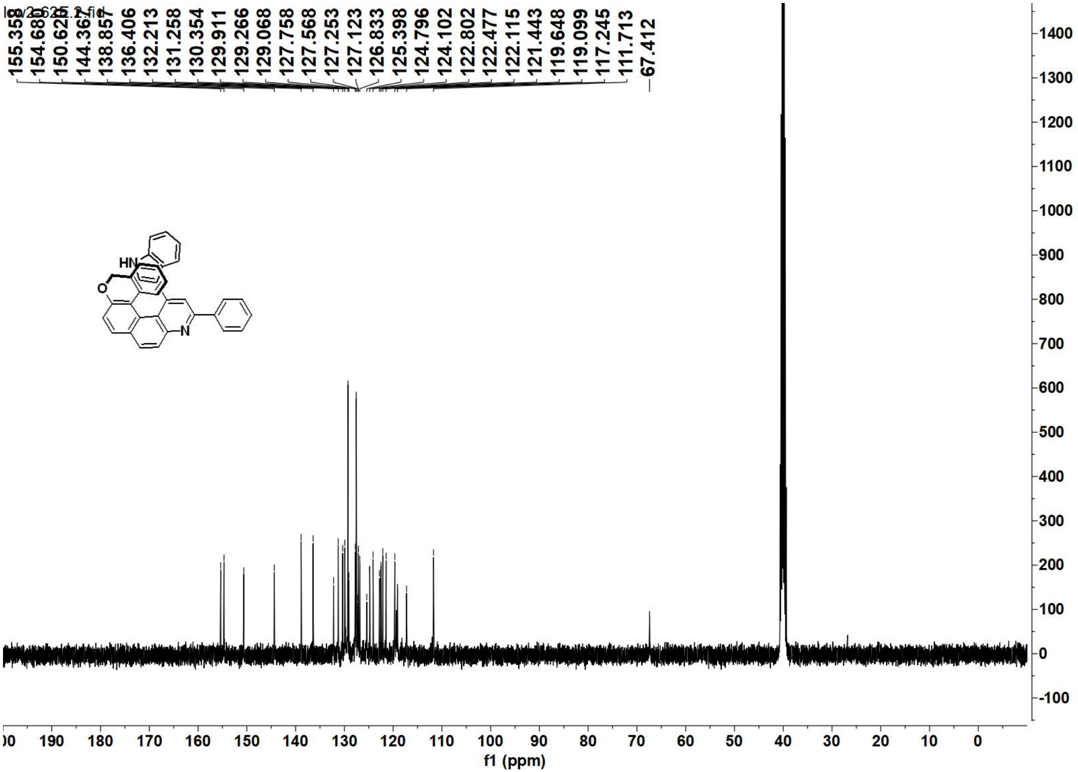

^{1}H NMR (400 MHz, DMSO-d_6) of **5al**

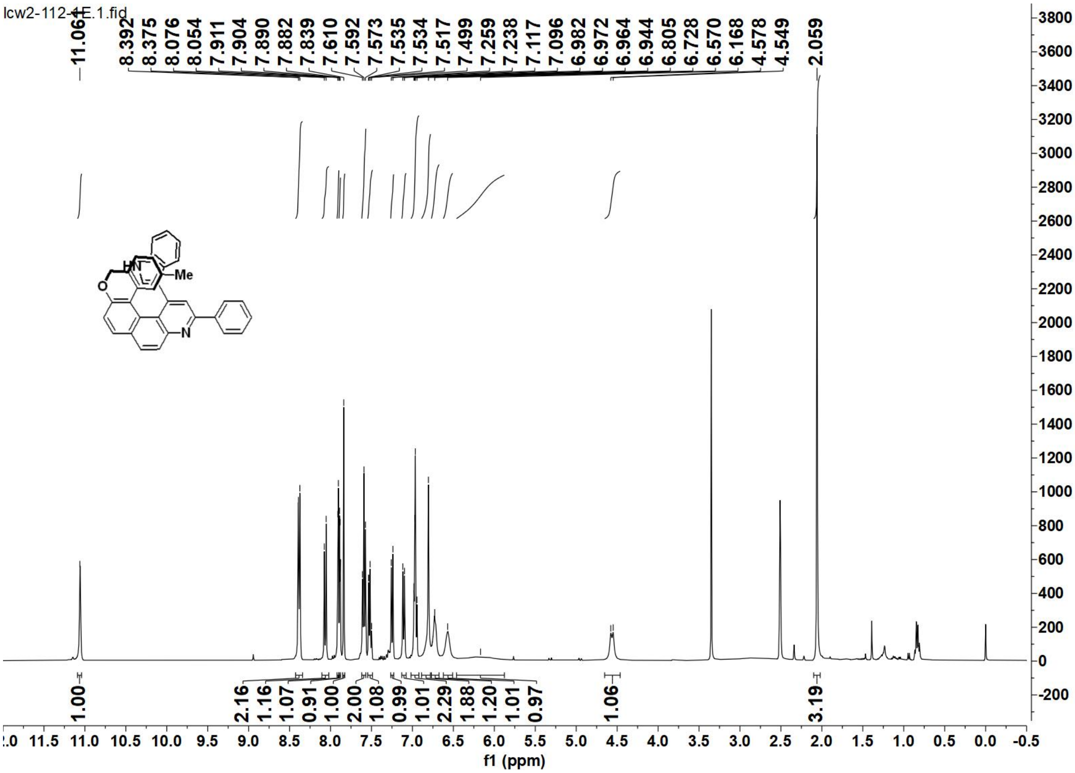

^{13}C NMR (101 MHz, DMSO-d_6) of **5al**

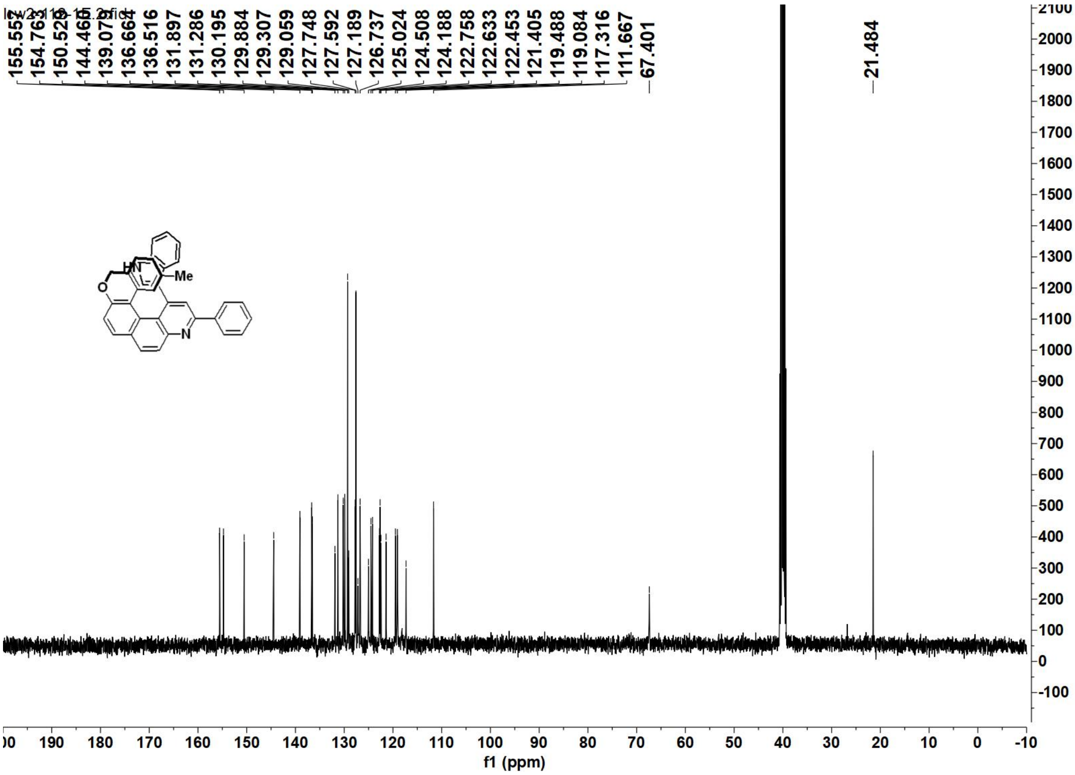

^{1}H NMR (400 MHz, DMSO-d_6) of **5am**

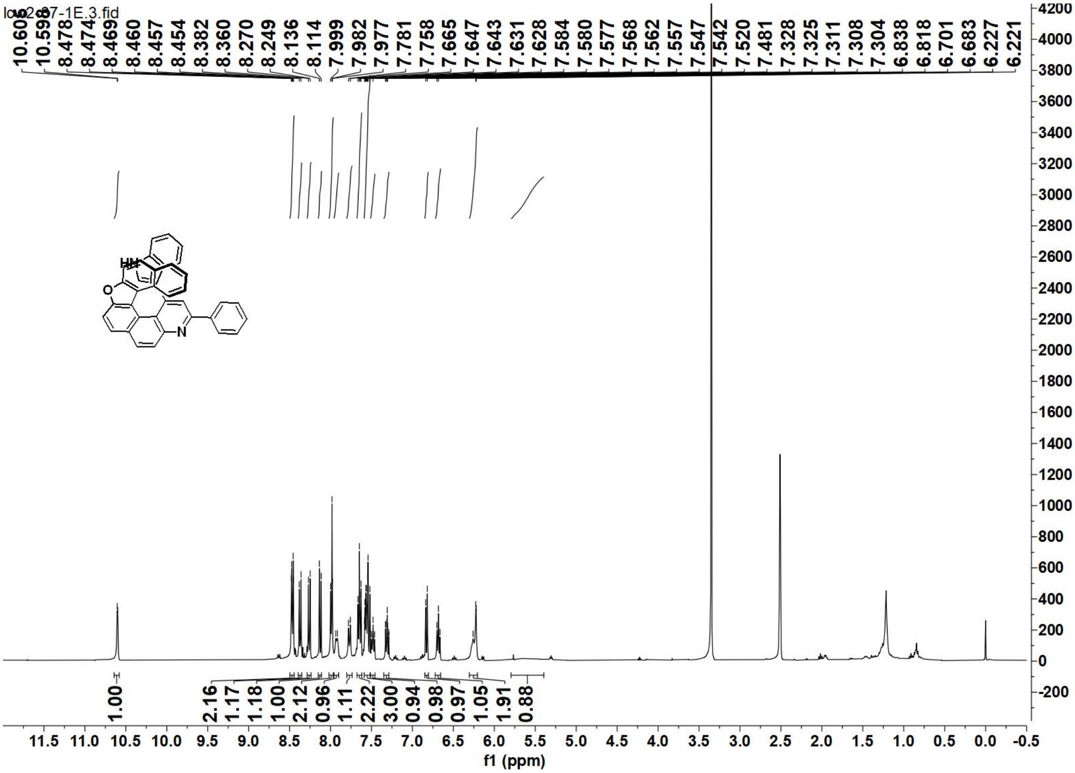

^{13}C NMR (101 MHz, DMSO-d_6) of **5am**

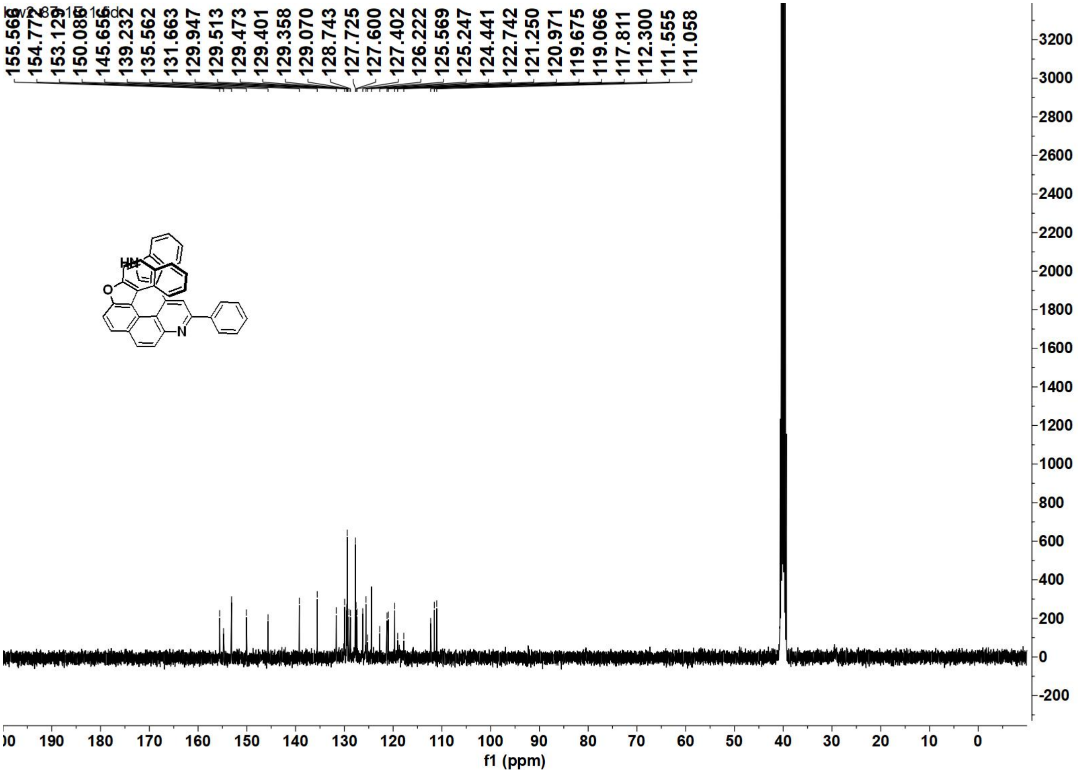

^{1}H NMR (400 MHz, DMSO-d_6) of **6a**

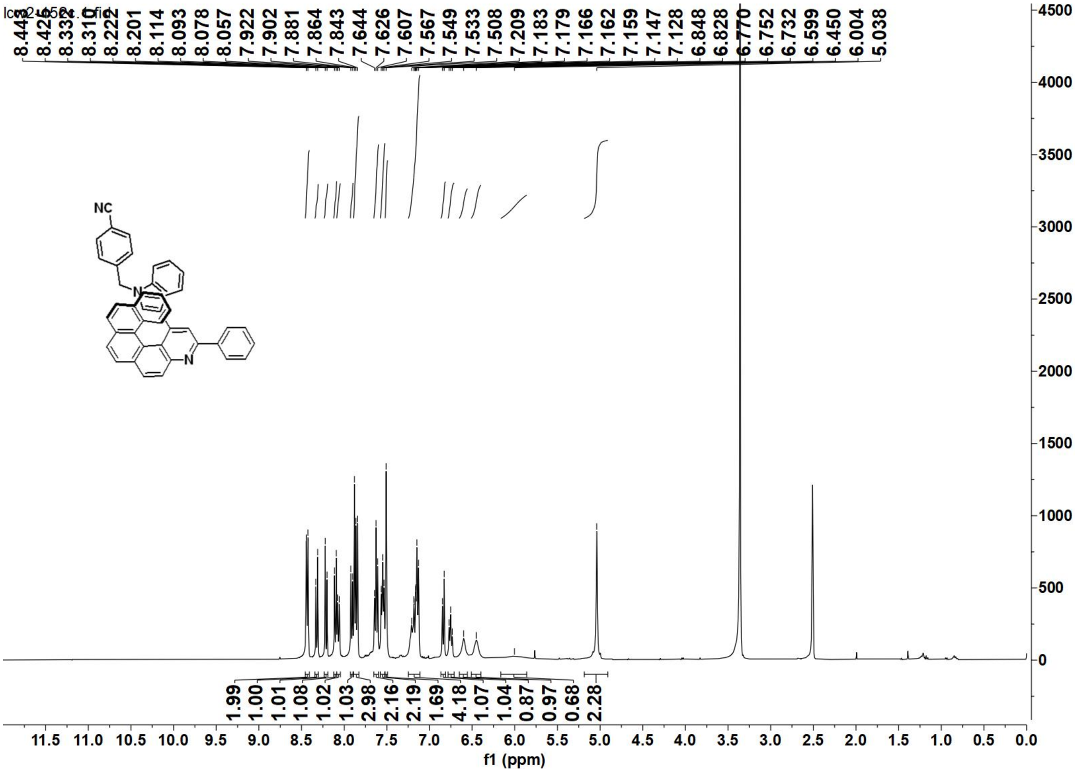

^{13}C NMR (400 MHz, DMSO-d_6) of **6a**

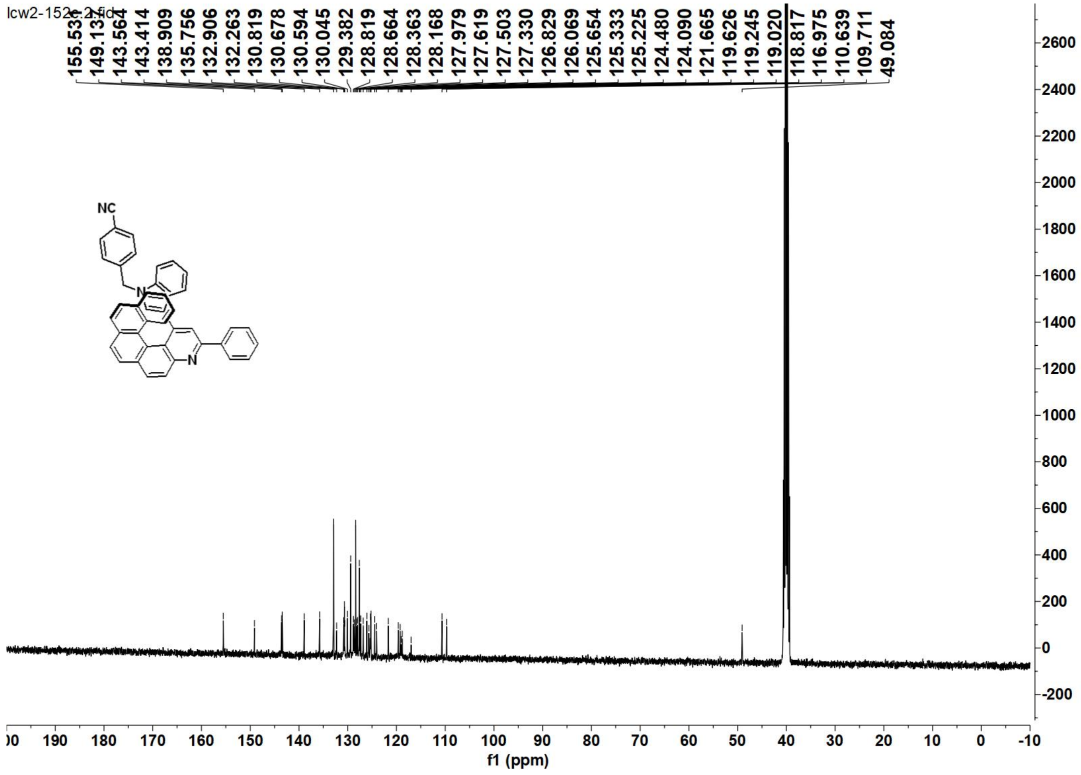

^{1}H NMR (400 MHz, DMSO-d_6) of **6b**

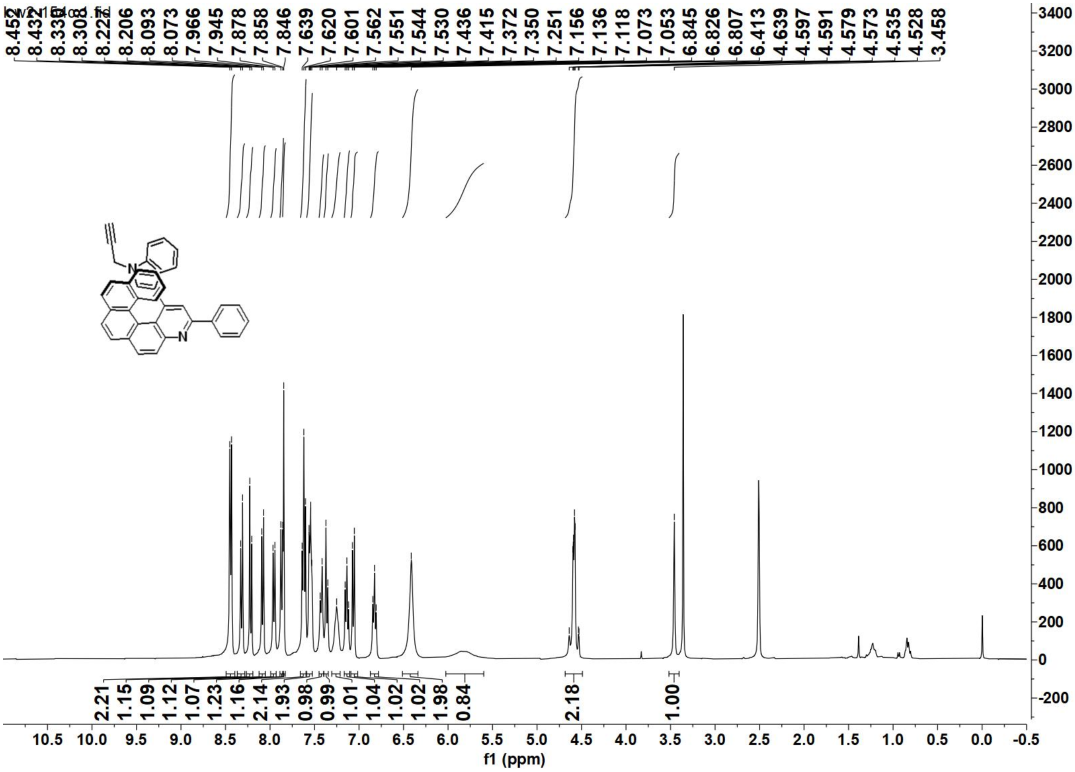

^{13}C NMR (400 MHz, DMSO-d_6) of **6b**

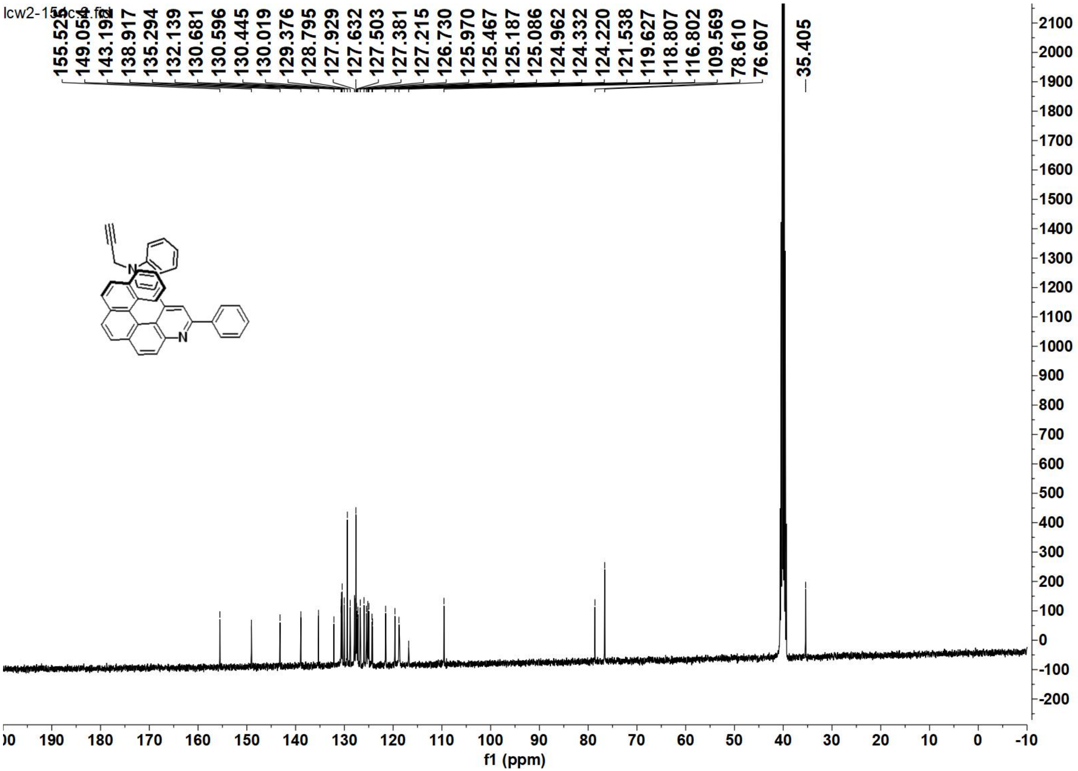

2. Supplementary References

1. Sako, M., et al. fficient Enantioselective Synthesis of Oxahelicenes Using Redox Acid Cooperative Catalysts. *J. Am. Chem. Soc.* **138**. 11481−11484 (2016).
2. Wu, J., Yu, J., Wang, Y. & Zhang, P. Direct Amination of Phenols under Metal-Free Conditions. *Synlett* **24**. 1448-1454 (2013).
3. Yu, J., Zhang, P., Wu, J. & Shang, Z. Metal-free C–N bond-forming reaction: straightforward synthesis of anilines, through cleavage of aryl C–O bond and amide C–N bond. *Tetrahedron Lett.* **54**. 3167-3170 (2013).
4. Frigoli, M., et al. P-Type Photochromism of New Helical Naphthopyrans: Synthesis and Photochemical, Photophysical and Theoretical Study. *Chemphyschem* **16**. 2447-2458 (2015).
5. Yao, T., Campo, M. A. & Larock, R. C. Synthesis of Polycyclic Aromatic Iodides via ICl-Induced Intramolecular Cyclization. *Org. Lett.* **6**. 2677-2680 (2004).
6. Campo, M. A. & Larock, R. C. Synthesis of Fluoren-9-ones via Palladium-Catalyzed Cyclocarbonylation of o-Halobiaryls. *Org. Lett.* **2**. 3675-3677 (2000).
7. Campo, M. A. & Larock, R. C. Synthesis of Fluoren-9-ones by the Palladium-Catalyzed Cyclocarbonylation of o-Halobiaryls. *J. Org. Chem.* **67**. 5616-5620 (2002).
8. Liu, P., et al. Simultaneous Control of Central and Helical Chiralities: Expedient Helicoselective Synthesis of Dioxa[6]helicenes. *J. Am. Chem. Soc.* **142**. 16199-16204 (2020).
9. You, J., Song, C., Yan, T., Sun, Z., Li, Y. & Suo, Y. An improved reagent for determination of aliphatic amines with fluorescence and online atmospheric chemical ionization-mass spectrometry identification. *AnalChimActa.* **658**. 98-105 (2010).

10. Cadart, T., et al. Rhodium-Catalyzed Enantioselective Synthesis of Highly Fluorescent and CPL-Active Dispiroindeno[2,1-c]fluorenes. *Chem. Eur. J.* **27**. 11279-11284 (2021).

11. M. J. Frisch, G. W. Trucks, H. B. Schlegel, G. E. Scuseria, M. A. Robb, J. R. Cheeseman, G. Scalmani, V. Barone, G. A. Petersson, H. Nakatsuji, X. Li, M. Caricato, A. V. Marenich, J. Bloino, B. G. Janesko, R. Gomperts, B. Mennucci, H. P. Hratchian, J. V. Ortiz, A. F. Izmaylov, J. L. Sonnenberg, Williams, F. Ding, F. Lipparini, F. Egidi, J. Goings, B. Peng, A. Petrone, T. Henderson, D. Ranasinghe, V. G. Zakrzewski, J. Gao, N. Rega, G. Zheng, W. Liang, M. Hada, M. Ehara, K. Toyota, R. Fukuda, J. Hasegawa, M. Ishida, T. Nakajima, Y. Honda, O. Kitao, H. Nakai, T. Vreven, K. Throssell, J. A. Montgomery Jr., J. E. Peralta, F. Ogliaro, M. J. Bearpark, J. J. Heyd, E. N. Brothers, K. N. Kudin, V. N. Staroverov, T. A. Keith, R. Kobayashi, J. Normand, K. Raghavachari, A. P. Rendell, J. C. Burant, S. S. Iyengar, J. Tomasi, M. Cossi, J. M. Millam, M. Klene, C. Adamo, R. Cammi, J. W. Ochterski, R. L. Martin, K. Morokuma, O. Farkas, J. B. Foresman, D. J. Fox, Gaussian 16 Rev. A.03, Wallingford, CT, USA. 2016. *Tetrahedron*.

12. Lee, C., Yang, W. & Parr, R. G. Development of the Colic-Salvetti correlation-energy formula into a functional of the electron density. *Phys. Rev. B.* **37**. 785-789 (1988).

13. Stephens, P. J., Devlin, F. J., Chabalowski, C. F. & Frisch, M. J. Ab Initio Calculation of Vibrational Absorption and Circular Dichroism Spectra Using Density Functional Force Fields. *J. Phy. Chem.* **98**. 11623-11627 (1994).

14. Vosko, S. H., Wilk, L. & Nusair, M. Accurate spin-dependent electron liquid correlation energies for local spin density calculations: a critical analysis. *Can. J. Phys.* **58**. 1200-1211 (1980).

15. Grimme, S., Antony, J., Ehrlich, S. & Krieg, H. A consistent and accurate ab initio parametrization of density functional dispersion correction (DFT-D) for the 94 elements H-Pu. *J. Chem. Phys.* **132**. 154104 (2010).

16. S. Grimme, S. Ehrlich, L. Goerigk, J. Comput. *Chem.* **32**, 1456-1465 (2011).
17. Weigend, F. & Ahlrichs, R. Balanced basis sets of split valence, triple zeta valence and quadruple zeta valence quality for H to Rn: Design and assessment of accuracy. *Chem. Phys.* **7**. 3297-3305 (2005).
18. Zhao, Y. & Truhlar, D. G. Density Functionals with Broad Applicability in Chemistry. *Acc. Chem. Res.* **41**. 157-167 (2008).
19. Zhao, Y. & Truhlar, D. G. The M06 suite of density functionals for main group thermochemistry, thermochemical kinetics, noncovalent interactions, excited states, and transition elements: two new functionals and systematic testing of four M06-class functionals and 12 other functionals. *Theor. Chem. Acc.* **120**. 215-241 (2008).
20. A. V. Marenich, C. J. Cramer, D. G. Truhlar, *J. Phys. Chem. B*. **113**, 6378-6396(2009).
21. Lemos, B. C., Venturini Filho, E., Fiorot, R. G., Medici, F., Greco, S. J. & Benaglia, M. Enantioselective Povarov Reactions: An Update of a Powerful Catalytic Synthetic Methodology. *Eur. J. Org. Chem.* **e202101171** (2022).
22. Clerigue, J., Ramos, M. T. & Menendez, J. C. Enantioselective catalytic Povarov reactions. *Org. Biomol. Chem.* **20**. 1550-1581 (2022).
23. Bergonzini, G., Gramigna, L., Mazzanti, A., Fochi, M., Bernardi, L. & Ricci, A. Organocatalytic asymmetric Povarov reactions with 2- and 3-vinylindoles. *Chem. Commun.* **46**. 327-329 (2010).
24. Dai, W., Jiang, X.-L., Tao, J.-Y. & Shi, F. Application of 3-Methyl-2-vinylindoles in Catalytic Asymmetric Povarov Reaction: Diastereo- and Enantioselective Synthesis of Indole-Derived Tetrahydroquinolines. *J. Org. Chem.* **81**. 185-192 (2016).
25. Bisag, G. D., et al. Central-to-Axial Chirality Conversion Approach Designed on Organocatalytic Enantioselective Povarov Cycloadditions: First Access to Configurationally Stable

Indole-Quinoline Atropisomers. *Chem. Eur. J.* **25**. 15694-15701 (2019).
